# Copper(I)-catalyzed asymmetric decarboxylative Mannich reaction enabled by acidic activation of 2*H*-azirines

**DOI:** 10.1038/s41467-019-09750-5

**Published:** 2019-04-12

**Authors:** Hai-Jun Zhang, Yan-Cheng Xie, Liang Yin

**Affiliations:** CAS Key Laboratory of Synthetic Chemistry of Natural Substances, Center for Excellence in Molecular Synthesis, Shanghai Institute of Organic Chemistry, University of Chinese Academy of Sciences, Chinese Academy of Sciences, 345 Lingling Road, Shanghai, 200032 China

## Abstract

Chiral aziridines are structure units found in many biologically active compounds and are important building blocks in organic synthesis. Herein, by merging nucleophilic generation through copper(I)-catalyzed decarboxylation and activation of poorly electrophilic 2*H*-azirines through protonation with carboxylic acids, an asymmetric decarboxylative Mannich reaction between α,α-disubstituted cyanoacetic acids and 2*H*-azirines is uncovered, which leads to generation of chiral aziridines containing vicinal tetrasubstituted and acyclic quaternary stereogenic carbon centers in good to excellent diastereo- and enantioselectivities. At last, transformations of the produced chiral aziridine are successfully carried out to deliver synthetically useful compounds.

## Abstract (web summary)

Due to their poor electrophilicity, 2*H*-azirines do not easily react with nucleophiles. Here, the authors show an acidic activation of 2*H*-azirines by cyanoacetic acid coupling partners affording chiral aziridines containing vicinal tetrasubstituted and acyclic quaternary stereogenic carbon centers.

## Introduction

Nature uses a mild but powerful strategy to generate a nucleophile through decarboxylation of a malonic acid half thioester in the presence of an enzyme, which is typically employed in the biosynthesis of fatty acids and polyketides^1^. Inspired by this fantastic method in nature, Shair and colleagues^2^ developed a Cu(II)-catalyzed asymmetric thioester aldol reaction, which was compatible with a broad range of protic functional groups and enolizable aldehydes due to the mild acidic reaction conditions^2^. Unfortunately, their following research uncovered that their reaction occurred by a different mechanism from the nature’s reaction (the nucleophile was actually not generated through decarboxylation)^3^. In 2009, Shibasaki and Kanai group^4^ mimicked the method in nature by using α-methyl-α-phenyl cyanoacetic acid as a pronucleophile and accomplished a decarboxylative Mannich-type reaction of *N*-diphenyl-phosphinoyl aldimines and a decarboxylative aldol reaction of aldehydes under copper(I) catalysis, which generated contiguous trisubstituted and acyclic quaternary stereocenters in moderate to high diastereo- and enantioselectivities^4,5^.

**Fig. 1:**
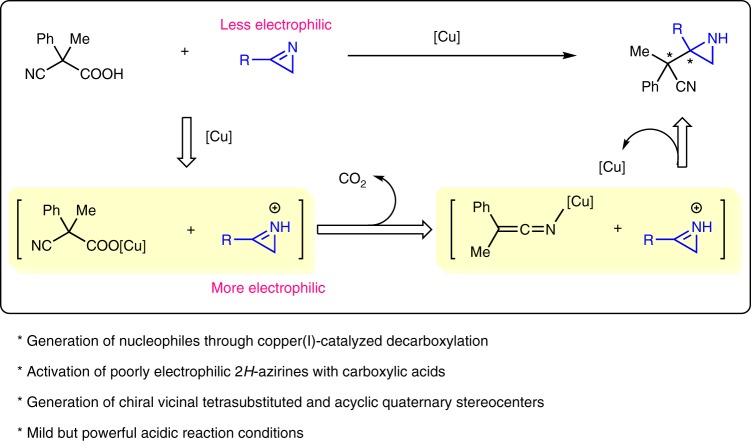
Initial design. Our working hypothesis for the catalytic asymmetric decarboxylative Mannich reaction of 2*H*-azirines

However, in the above two catalytic asymmetric decarboxylative reactions, cyanoacetic acid was only employed as the precursor for decarboxylative nucleophile generation^6,7^ by anion exchange with CuOAc–bisphosphine complex (decarboxylative protonation was a side reaction^8^). However, the acidic nature of cyanoacetic acid was not fully utilized. Considering the basicity and the poor electrophilicity of 2*H*-azirines, we propose a strategy that basic 2*H*-azirines might be activated by a cyanoacetic acid through protonation to afford more electrophilic iminium species (Fig. 1)^9,10^. Meanwhile, the anion exchange leads to the formation of copper(I) cyanoacetate, which affords the nucleophilic copper(I) ketenimide through extrusion of CO_2_. Then, asymmetric addition of the nucleophilic copper(I) ketenimide to the electrophilic iminium species would allow the easy formation of a series of chiral aziridines in good yields, which contain chiral adjacent tetrasubstituted and acyclic quaternary stereocenters. These chiral aziridines would serve as intermediates to furnish synthetically useful compounds through the transformations of the aziridine functional group.

It is well known that chiral aziridines are important intermediates in organic synthesis, as they can react with nucleophiles to generate chiral amines through ring-opening reactions^11–16^. Moreover, chiral aziridines are structure units found in many natural products and man-made molecules, which exhibit a broad range of biological properties, such as antitumor and antibacterial activities^17–19^. Therefore, the asymmetric synthesis of chiral aziridines has received significant attention from the chemical community. One of the straightforward methods is the catalytic asymmetric addition of nucleophiles to 2*H*-azirines^20,21^. However, the lower reactivity of 2*H*-azirines has led to much less developments in this area^22–28^. Furthermore, it is well known that the asymmetric construction of vicinal chiral tetrasubstituted (including quaternary) and acyclic quaternary carbon stereocenters^29–31^ was very challenging and achieved much less success largely due to the significantly increased steric hindrance and the difficulty to control the asymmetric induction^32–35^.

Here, by activation of the poorly electrophilic 2*H*-azirines with carboxylic acids, we disclose a copper(I)-catalyzed decarboxylative Mannich reaction of 2*H*-azirines, which affords a series of chiral aziridines in good to high yields, diastereoselectivity, and enantioselectivity. More importantly, adjacent chiral tetrasubstituted and acyclic quaternary stereocenters are generated efficiently.

## Results

### Optimization of reaction conditions

The reaction between α-methyl-α-phenyl cyanoacetic acid (**1a**) and 2*H*-azirine **2a** was studied for the optimization of reaction conditions as shown in Table 1. The decarboxylative Mannich reaction proceeded smoothly in the presence of 5 mol % of CuOAc and 5 mol % of (*R*)-BINAP at 0 ^o^C in tetrahydrofuran (THF), which delivered product **3a** in 81% yield with 2.0/1 dr and 8% ee for the major diastereoisomer (Table 1, entry 1). Screening of the commercially available bisphosphine ligands, including (*R*)-TOL-BINAP, (*R*)-SEGPHOS, (*R*)-DIFLUORPHOS, (*R*,*R*)-QUINOXP*, (*R*,*R*)-Ph-BPE, (*R*,*R*_*P*_)-TANIAPHOS, (*R*)-DTBM-SEGPHOS, and (*R*)-DIPA-MeO-BIPHEP, identified (*R*)-DTBM-SEGPHOS and (*R*)-DIPA-MeO-BIPHEP as the suitable ligands (Table 1, entries 2–9). With (*R*)-DTBM-SEGPHOS as the ligand, product **3a** was generated in 88% yield with 4.0/1 dr and 93% ee for the major diastereoisomer (Table 1, entry 8). In the case of (*R*)-DIPA-MeO-BIPHEP, product **3a** was obtained in 62% yield with 5.7/1 dr and 91% ee for the major diastereoisomer (Table 1, entry 9).

**Table 1:**
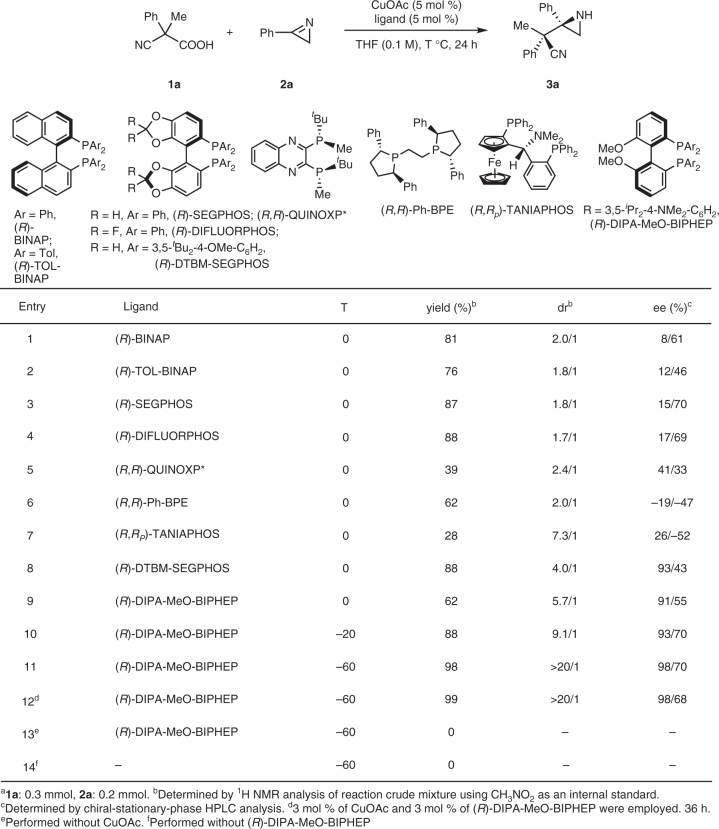
Optimization of the reaction conditions^a^

**Fig. 2:**
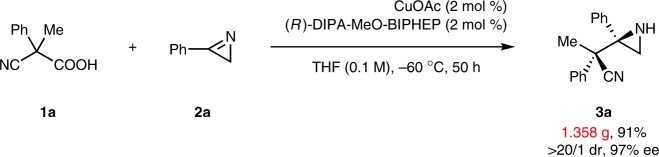
Gram-scale reaction. Conditions: **1a** (9 mmol, 1.577 g), **2a** (6 mmol, 0.703 g), CuOAc (2 mol %), (*R*)-DIPA-MeO-BIPHEP (2 mol %), and THF (60 mL), −60 ^o^C, 50 h

With (*R*)-DTBM-SEGPHOS as the ligand, the studies of copper(I) source, reaction solvent, and temperature effect were not fruitful (see Supplementary Table 1-3 for details). Fortunately, decreasing the reaction temperature to −20 ^o^C resulted in improved yield, diastereo-, and enantioselectivities in the case of (*R*)-DIPA-MeO-BIPHEP (Table 1, entry 10). By further lowering the reaction temperature to −60 ^o^C, product **3a** was generated in 98% yield with >20/1 dr and 98% ee (Table 1, entry 11). Moreover, the loading of CuOAc-(*R*)-DIPA-MeO-BIPHEP complex was successfully reduced to 3 mol % by prolonging the reaction time from 24 h to 36 h (Table 1, entry 12). It was verified that both CuOAc and ligand ((*R*)-DIPA-MeO-BIPHEP) were indispensable, as no product **3a** was observed in the absence of either (Table 1, entries 13, 14). The catalyst loading was further reduced to 2 mol % with the success of a gram-scale reaction as shown in Fig. 2. Remarkably, 1.358 g of aziridine **3a** was isolated in 91% yield with >20/1 dr and 97% ee.

### Substrate scope

The study of the substrate scope of aromatic 2*H*-azirines (**2**) with 3 mol % of CuOAc-(*R*)-DIPA-MeO-BIPHEP complex was described in Table 2. Both electron-donating groups and electron-withdrawing groups were accepted at the *para*-positon of the phenyl group (**3a**–**3j**). Although the diastereoselectivity was moderate in some cases, both the yield and the enantioselectivity were high to excellent. Several aromatic 2*H*-azirines with a substituent at the *meta*-position served as appropriate substrates to afford the aziridines in good to excellent stereoselectivities (**3k**–**3n**). Unfortunately, a substituent was not well tolerated at the *ortho*-position largely due to the increased steric hindrance. 2*H*-Azirine with a 2-naphthyl group was also a suitable substrate as excellent results were obtained (**3o**). Moreover, both 3-thienyl and benzo[*b*]thiophen-2-yl groups did not have a negative effect on the reaction results (**3p** and **3q**).

**Table 2:**
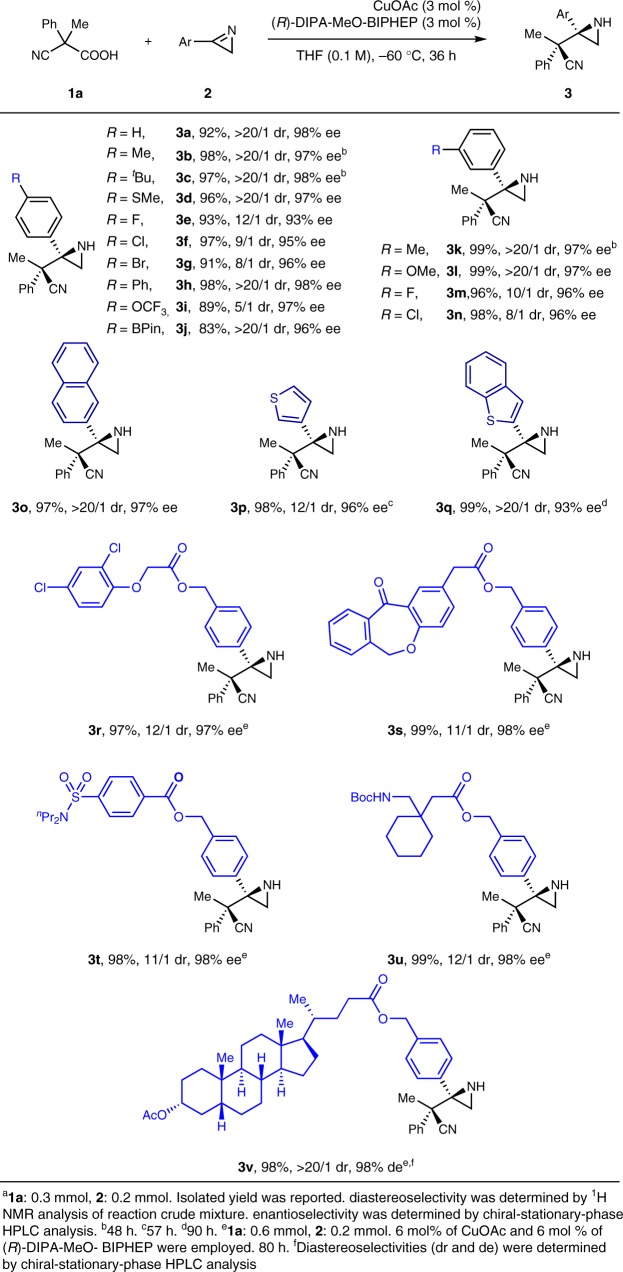
Substrate scope of aromatic 2*H*-azirines^a^

Then, complex molecules containing an aromatic 2*H*-azirine moiety were investigated. 2,4-Dichlorophenoxyacetic acid, a systemic herbicide, was successfully attached to the 2*H*-azirine group through an ester linker (**2r**). **2r** underwent the copper(I)-catalyzed decarboxylative Mannich reaction smoothly to afford product **3r** in excellent both yield and stereoselectivity. By using the same linker, isoxepac acid (an advanced intermediate toward the synthesis of olopatadine, which is sold as a prescription eye drop), probenecid (a medication that is primarily used in treating gout and hyperuricemia), and gabapentin (a medication that is used primarily to treat seizures and neuropathic pain) were attached with the 2*H*-azirine moiety to generate **2s**, **2t**, and **2u**. These three 2*H*-azirines reacted with **1a** nicely to furnish the aziridines (**3s**, **3t**, and **3u**) in uniformly excellent yield, high diastereoselectivity, and excellent enantioselectivity. It was noted that a ketone unit, a tertiary sulfonate amide, and a secondary carbamate did not disturb both the reactivity and the stereoselectivity. Moreover, the 2*H*-azirine moiety was introduced to lithocholic acid (a natural steroid molecule) through the same linker to generate a complex 2*H*-azirine (**2v**), which also served as a competent substrate to furnish product **3v** in excellent both yield and diastereoselectivities.

As (*R*)-DTBM-SEGPHOS led to better control of the diastereoselectivity than (*R*)-DIPA-MeO-BIPHEP (6/1 dr vs. 4/1 dr) in the reaction of aliphatic 2*H*-azirine **4a**, (*R*)-DTBM-SEGPHOS was employed for the study of aliphatic 2*H*-azirines (Table 3). Although the diastereoselectivity was not satisfactory in the cases of **5a** and **5b**, both the yields and the enantioselectivity were excellent. The same situation was also observed in the reaction of α,β-unsaturated 2*H*-azirine **4c**. The aryl group in α,α-disubstituted cyanoacetic acid (**1**) was successfully extended to *para*-methyl-phenyl (**1b**), *para*-chloro-phenyl (**1c**), and 2-thienyl groups (**1d**) with high to excellent stereoselectivities (**5d**, **5e**, and **5f**). It was noted that cyanoacetic acid **1c** with a *para*-chloro-phenyl led to moderate yield. Moreover, the alkyl group in **1** was successfully changed from methyl (**1a**) to ethyl and allyl groups (**1e** and **1f**). Unfortunately, the yields were moderate and the diastereoselectivity decreased significantly, possibly due to the extenuated difference of the steric hindrance between phenyl and ethyl or allyl groups (**5g** and **5h**). Furthermore, the 2*H*-azirine moiety was tethered with indometacin (a nonsteroidal anti-inflammatory drug), dehydroepiandrosterone (an endogenous steroid hormone), and a protected glucose to give three complex aliphatic 2*H*-azirines (**4i**, **4j**, and **4k**). Their decarboxylative Mannich reactions with **1a** proceeded smoothly to give the aziridines (**5i**, **5j**, and **5k**) in high yields with high to excellent stereoselectivities.

**Table 3:**
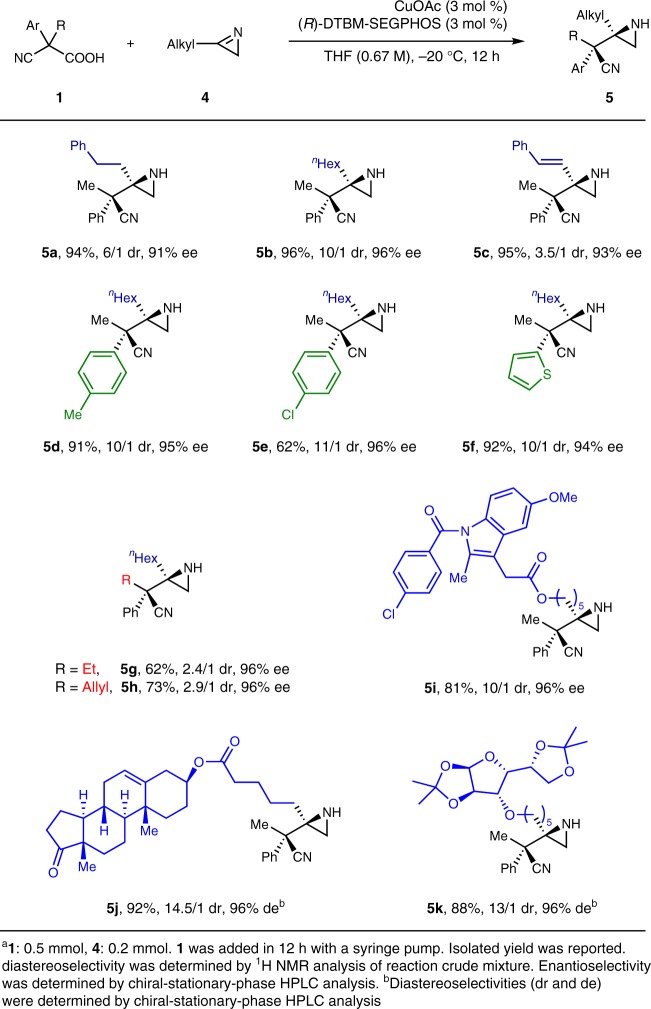
Substrate scope of aliphatic 2*H*-azirines^a^

### Demonstration of the strategy

**Fig. 3:**
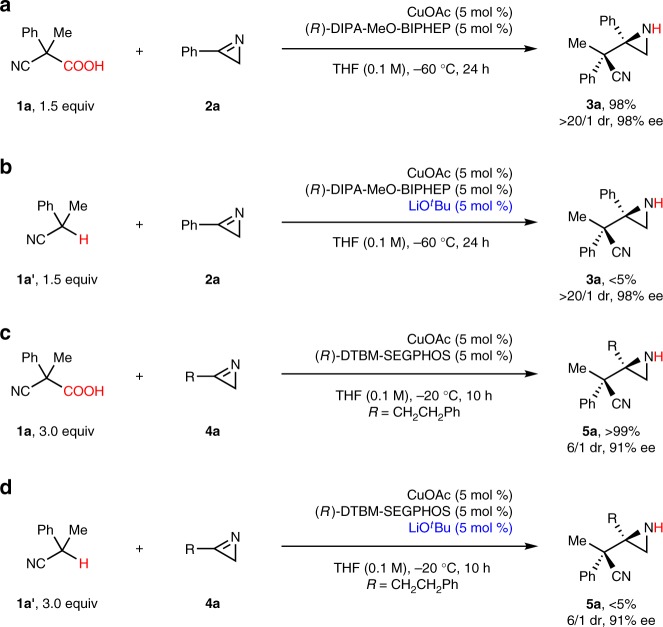
Demonstration of the strategy―one substrate activation by the other. **a** Decarboxylative Mannich reaction of aromatic 2*H*-azirine. **b** Proton-transfer Mannich reaction of aromatic 2*H*-azirine. **c** Decarboxylative Mannich reaction of aliphatic 2*H*-azirine. **d** Proton-transfer Mannich reaction of aliphatic 2*H*-azirine

The present decarboxylative Mannich reaction exhibited an impressive advantage over the classical proton-transfer version. As shown in Fig. 3, the proton-transfer Mannich reaction of **1a’** and **2a** proceeded in <5% yield in the presence of 5 mol % of CuOAc-(*R*)-DIPA-MeO-BIPHEP complex (Fig. 3b), while the decarboxylative version occurred in 98% yield (Fig. 3a). Moreover, the retro-Mannich reaction of **3a** in the presence of 5 mol % of CuO^*t*^Bu and 5 mol % of (*R*)-DIPA-MeO-BIPHEP at −60 ^o^C did not proceed at all, indicating that the very low yield of the proton-transfer Mannich reaction between **1a’** and **2a** was not attributed to the retro-Mannich reaction of **3a** under basic conditions. The same diastereo- and enantioselectivities were observed both in the decarboxylative version and in the proton-transfer version, indicating that the same copper(I) intermediate was generated. It is reasonable that the proton-transfer version led to very low yield as 2*H*-azirine **2a** is a poor electrophile as described in literature^25,28^. As 2*H*-azirine **2a** is an imine base, it is also reasonable that the protonation of **2a** by **1a** might occur smoothly to give an iminium cation, which is a highly electrophilic species. Moreover, it was observed that the copper(I)-catalyzed decarboxylation protonation of **1a** proceeded very slowly in the absence of **2a** at −60 ^o^C, which indicated the interaction between **1a** and **2a** (the activation of **2a** by **1a** through a hydrogen-bonding effect can not be excluded completely at present). The difference of the electrophiles in the Mannich reaction resulted in different yields. The same tendency was also observed in the Mannich reaction of aliphatic 2*H*-azirine **4a** with (*R*)-DTBM-SEGPHOS as the ligand (Fig. 3c, d). It is obvious that the present acidic reaction conditions are superior to the classical basic proton-transfer reaction conditions in the construction of contiguous tetrasubstituted and quaternary stereocenters.

### Transformations

**Fig. 4:**
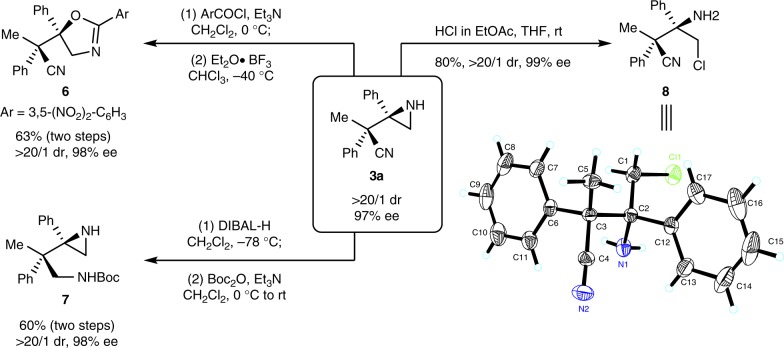
Transformations of the product **3a**. Boc, tert-butoxycarbonyl; DIBAL-H, diisobutylaluminium hydride

Transformations of **3a** were shown in Fig. 4. After protection of **3a** with an acid chloride, the rearrangement of the aziridine moiety in **3a** to an oxazoline group produced **6** in 63% yield with maintained enantioselectivity^25^.The reduction of cyano group by DIBAL-H afforded carbamate **7** in 60% yield after protection of the free amine with Boc_2_O. Moreover, the acidic opening of the aziridine group in **3a** with HCl proceeded smoothly to furnish amine **8** in 80% yield. Obviously, the primary alkyl chloride moiety in **8** allows further structure elaboration. At last, the absolute configurations of the stereocenters in **8** were determined by the X-ray analysis of its single crystals as depicted in Fig. 4, which led to the determination of the exact stereochemistry of **3a**. Then, the absolute configurations in **3** and **5** were assigned tentatively by analogy (see Supplementary Fig. 40 and Supplementary Table 4 for details).

## Discussion

In conclusion, a copper(I)-catalyzed decarboxylative Mannich reaction between α,α-disubstituted cyanoacetic acids and various 2*H*-azirines was disclosed, which efficiently constructed chiral aziridines bearing vicinal tetrasubstituted and acyclic quaternary stereogenic carbon centers. The present reaction enjoyed the advantages of mild reaction conditions, easy reaction protocol, broad substrate scope, and high to excellent stereoselectivity. The activation of basic 2*H*-azirines by cyanoacetic acids, which might generate highly electrophilic iminium species, was proposed to be the key for the success of the present challenging reaction. At last, several transformations of the product were successfully carried out by means of the synthetically versatile cyano and aziridine groups. Expansion of both the present activation strategy and the mild nucleophile generation strategy through copper(I)-catalyzed decarboxylation in asymmetric catalysis is currently in progress in our laboratory.

## Methods

### General procedure for the synthesis of **3a**

A dried 25 mL Schlenk tube equipped with a magnetic stirring bar was charged with CuOAc (2.94 mg, 0.024 mmol) and (*R*)-DIPA-MeO-BIPHEP (26.2 mg, 0.024 mmol) in a glove box under Ar atmosphere. Anhydrous THF (4 mL) was added via a syringe. The mixture was stirred for 15 min to give a clear catalyst solution. Another dried 25 mL Schlenk tube equipped with a magnetic stirring bar was charged with aromatic 2*H*-azirines **2a** (0.2 mmol, 1.0 equiv, 23.4 mg). The catalyst solution (1.0 mL) containing copper(I) complex (0.006 mmol, 0.03 equiv) was added via a syringe. The reaction mixture was then cooled to −60 °C and cyanoacetic acid **1a** (0.3 M in THF, 1.0 mL, 0.3 mmol, 1.5 equiv, 52.6 mg) was added dropwise over 2 min. The resulting reaction mixture was stirred at −60 °C for 36 h. The diastereomeric ratio of the crude reaction mixture was determined by ^1^H NMR spectroscopy. Then, the residue was purified by silica gel column chromatography (petroleum ether/ethyl acetate = 5/1) to give **3a** in 92% yield as pale yellow solid.

## Supplementary information

Supplementary Information

## Data Availability

The X-ray crystallographic coordinates for structures reported in this study have been deposited at the Cambridge Crystallographic Data Centre (CCDC), under deposition number CCDC 1891666 (**8**). These data can be obtained free of charge from The Cambridge Crystallographic Data Center via www.ccdc.cam.ac.uk/data_request/cif. The data supporting the findings of this study are available within the article and its Supplementary Information file. Any further relevant data are available from the authors on request.
