# Supplementary material for: Copper(I)-catalyzed asymmetric decarboxylative Mannich reaction enabled by acidic activation of 2H-azirines
Source: Nat Commun. 2019 Apr 12;10:1699. doi: 10.1038/s41467-019-09750-5 (PMC6461707; doi:10.1038/s41467-019-09750-5)
Supplement: Supplementary file 1 — Supplementary Information [file 41467_2019_9750_MOESM1_ESM.pdf]

Copper(I)-catalyzed asymmetric decarboxylative Mannich reaction enabled by acidic activation of 2*H*-azirines

Zhang et al.

Supplementary Methods

General information

Nuclear Magnetic Resonance (NMR) spectra were acquired on an Agilent 400 or Agilent 500 or Bruker 400 spectrometer. For ^{1}H NMR, chemical shifts were reported in δ ppm referenced to an internal $SiMe_4$ standard. For ^{19}F NMR, $CFCl_3$ was used as the reference with chemical shift at 0 ppm. For ^{13}C NMR, chemical shifts were reported in the scale relative to NMR solvent ($CDCl_3$: δ 77.0 ppm; DMSO-d_6: δ 39.52 ppm) as an internal reference. Multiplicities are reported using the following abbreviations: br = broad, s = singlet, d = doublet, t = triplet, q = quartet, m = multiplet. High-resolution mass spectra (ESI) were measured on Agilent 6200 Series TOF/6500 Series. High-resolution mass spectra (DART) were measured on Thermo Fisher Scienticfic LTQ FTICR-MS. Infrared (IR) spectra were recorded on Thermo Scientific Nicolet iS5 FT-IR. Optical rotation was measured on an Anton Paar Mcp 5500 polarimeter. HPLC analysis was conducted on a Shimadzu HPLC system equipped with Daicel chiral-stationary-phase columns (ϕ 4.6 mm × 250 mm).

Cyanoacetic acids were synthesized according to the literature method.[1] All other reagents were obtained commercially unless otherwise noted.

Preparation of 2H-azirines

Preparation of 2a-2q

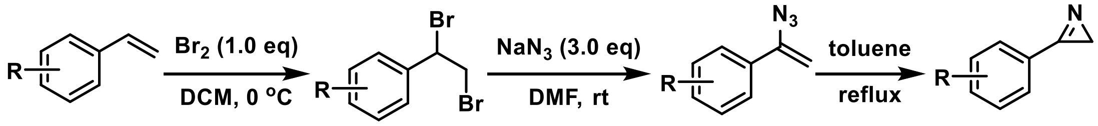

To a solution of alkene (5 mmol) in DCM (10 mL) cooled to 0 °C was added bromine (1.0 M DCM solution) dropwise until the solution turned orange. The resulting solution was stirred at room temperature for 5 minutes. Upon completion as indicated by TLC, the reaction was quenched with saturated aqueous Na_2SO_3 and stirred vigorously until the orange color disappeared. The organic phase was separated and the aqueous phase was extracted with DCM (20 mL × 2). The organic extracts were dried over anhydrous Na_2SO_4 and the solvent was removed under reduced pressure to give the crude product which was used in next step without further purification.

To a solution of dibromide in DMF (10 mL) was added NaN_3 (3.0 equiv). The mixture was stirred overnight at room temperature, then diluted with water and extracted with diethyl ether (20 mL × 3). The combined organic layers were washed for three times with water, and dried over anhydrous Na_2SO_4. The solvent was removed under reduced pressure to give the crude product which was used in next step without further purification.

The crude vinyl azide was refluxed in toluene (0.1 M) for 2 hours. The reaction mixture was cooled to room temperature and concentrated under reduced pressure to give the crude product, which was purified by silica gel column chromatography (petroleum ether /ethyl acetate) to afford 2*H*-azirine.

Most of aromatic 2*H*-azirines were known compounds.[2,3] Several new 2*H*-azirines were shown below.

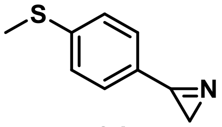

2d

^{1}H NMR (400 MHz, $CDCl_3$) δ 7.79 (d, *J* = 7.5 Hz, 2H), 7.36 (d, *J* = 7.6 Hz, 2H), 2.54 (s, 3H), 1.76 (s, 2H) ppm.
^{13}C NMR (100 MHz, $CDCl_3$) δ 164.82, 145.62, 129.70, 125.48, 121.50, 19.50, 14.77 ppm.
HRMS (DART) m/z [M+H]$^+$: calcd. 164.0528, found 164.0529.
IR (film): 3040, 2920, 1735, 1592, 820 cm^{-1}.

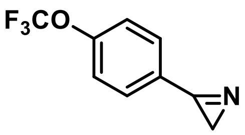

2i

^{1}H NMR (400 MHz, CDCl$_3$) δ 7.98-7.95 (m, 2H), 7.41 (d, *J* = 8.0 Hz, 2H), 1.83 (s, 2H) ppm.
^{13}C NMR (100 MHz, CDCl$_3$) δ 164.93, 152.32 (q, *J* = 1.6 Hz), 131.27, 123.98, 121.13 (q, *J* = 1.0 Hz), 120.26 (q, *J* = 259.0 Hz), 19.96 ppm.
^{19}F NMR (376 MHz, CDCl$_3$) δ -57.74 (s) ppm.
HRMS (DART) m/z [M+H]$^+$: calcd. 202.0474, found 202.0475.
IR (film): 3051, 2985, 2104, 1746, 1605, 773, 681 cm^{-1}.

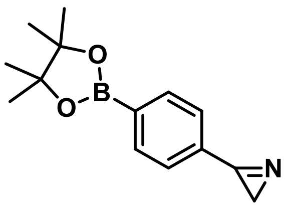

2j

^{1}H NMR (400 MHz, CDCl$_3$) δ 7.99 (d, *J* = 8.0 Hz, 2H), 7.89 (d, *J* = 8.0 Hz, 2H), 1.80 (s, 2H), 1.37 (s, 12H) ppm.
^{13}C NMR (100 MHz, CDCl$_3$) δ 165.96, 135.19, 128.55, 127.49, 126.12, 84.23, 24.84, 19.78 ppm.
HRMS (DART) m/z [M+H]$^+$: calcd. for $[C_{14}H_{19}O_2N^{10}B]^+$ 243.1540, found 243.1540.
IR (film): 2979, 1361, 1270, 1145, 857, 654 cm^{-1}.

Preparation of 2r-2v

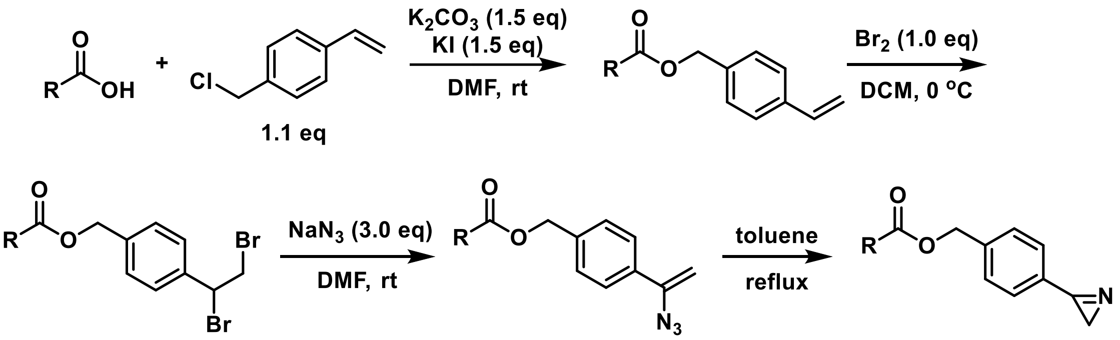

To a stirring suspension of acid (10 mmol), K_2CO_3 (1.5 equiv) and KI (1.5 equiv) in DMF (20 mL) was added 4-vinylbenzyl chloride (1.1 equiv). The mixture was stirred for 24 h at room temperature, then diluted with water and extracted with diethyl ether (20 mL × 3). The combined organic layers were washed for three times with water, and dried over anhydrous Na_2SO_4. The solvent was removed under reduced pressure to give the crude product, which was purified by silica gel column chromatography (petroleum ether /ethyl acetate) to afford alkene.

To a solution of alkene (5 mmol) in DCM (10 mL) cooled to 0 °C was added bromine (1.0 M DCM solution) dropwise until the solution turned orange. The resulting solution was stirred at room

temperature for 5 minutes. Upon completion as indicated by TLC, the reaction was quenched with saturated aqueous Na_2SO_3 and stirred vigorously until the orange color disappeared. The organic phase was separated and the aqueous phase was extracted with DCM (20 mL × 2). The organic extracts were dried over anhydrous Na_2SO_4 and the solvent was removed under reduced pressure to give the crude product which was used in next step without further purification.

To a solution of dibromide in DMF (10 mL) was added NaN_3 (3.0 equiv). The mixture was stirred overnight at room temperature, then diluted with water and extracted with diethyl ether (20 mL × 3). The combined organic layers were washed for three times with water, and dried over anhydrous Na_2SO_4. The solvent was removed under reduced pressure to give the crude product which was used in next step without further purification.

The crude vinyl azide was refluxed in toluene (0.1 M) for 2 hours. The reaction mixture was cooled to room temperature and concentrated under reduced pressure to give the crude product, which was purified by silica gel column chromatography (petroleum ether /ethyl acetate) to afford 2*H*-azirine.

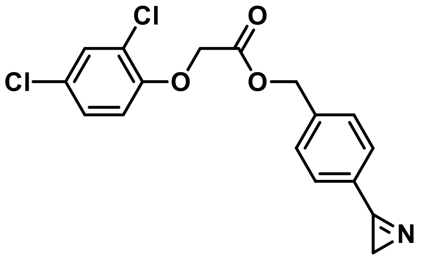

2r

^{1}H NMR (400 MHz, $CDCl_3$) δ 7.90 (d, *J* = 7.9 Hz, 2H), 7.52 (d, *J* = 7.9 Hz, 2H), 7.40-7.39 (m, 1H), 7.13 (dd, *J* = 8.7, 2.0 Hz, 1H), 6.77 (d, *J* = 8.8 Hz, 1H), 5.32 (s, 2H), 4.77 (s, 2H), 1.81 (s, 2H) ppm.
^{13}C NMR (100 MHz, $CDCl_3$) δ 167.74, 165.43, 152.12, 139.83, 130.29, 129.74, 128.56, 127.47, 127.14, 125.49, 124.15, 114.57, 66.26, 66.22, 19.80 ppm.
HRMS (ESI) m/z [M+H]$^+$: calcd. 350.0345, found 350.0345.
IR (film): 1763, 1489, 1232, 1085, 990, 819, 807 cm^{-1}.

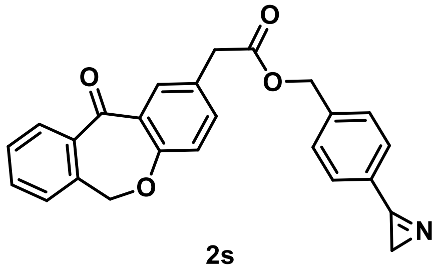

2s

^{1}H NMR (400 MHz, $CDCl_3$) δ 8.15 (s, 1H), 7.89 (d, *J* = 8.0 Hz, 3H), 7.59-7.41 (m, 5H), 7.37 (d, *J* = 7.3 Hz, 1H), 7.03 (d, *J* = 8.4 Hz, 1H), 5.23 (s, 2H), 5.19 (s, 2H), 3.73 (s, 2H), 1.79 (s, 2H) ppm.
^{13}C NMR (100 MHz, $CDCl_3$) δ 190.71, 170.97, 165.40, 160.45, 140.75, 140.28, 136.22, 135.38, 132.75, 132.42, 129.71, 129.35, 129.21, 128.32, 127.76, 127.28, 125.15, 125.04, 121.06, 73.52,

65.85, 40.04, 19.70 ppm.

HRMS (ESI) m/z [M+H]$^+$: calcd. 398.1387, found 398.1387.

IR (film): 3052, 2978, 1739, 1599, 1139, 762, 699 cm^{-1}.

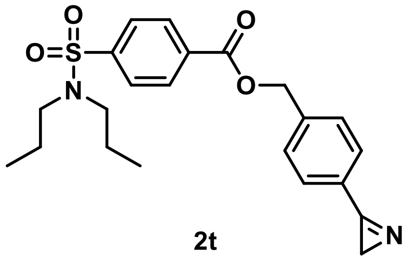

2t

^{1}H NMR (400 MHz, CDCl$_3$) δ 8.22-8.19 (m, 2H), 7.96-7.88 (m, 4H), 7.66-7.63 (m, 2H), 5.48-5.47 (m, 2H), 3.12-3.07 (m, 4H), 1.82-1.79 (m, 2H), 1.60-1.48 (m, 4H), 0.90-0.82 (m, 6H) ppm.

^{13}C NMR (100 MHz, CDCl$_3$) δ 165.46, 164.85, 144.50, 140.49, 132.91, 130.31, 129.84, 128.55, 127.02, 125.46, 66.47, 49.84, 21.86, 19.79, 11.10 ppm.

HRMS (ESI) m/z [M+H]$^+$: calcd. 415.1686, found 415.1686.

IR (film): 2968, 2935, 2876, 1727, 1271, 740, 694 cm^{-1}.

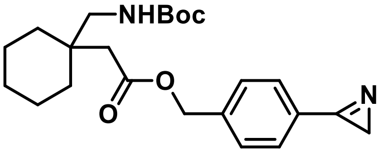

2u

^{1}H NMR (400 MHz, CDCl$_3$) δ 7.91 (d, *J* = 8.0 Hz, 2H), 7.56 (d, *J* = 7.9 Hz, 2H), 5.20 (s, 2H), 5.01 (s, 1H), 3.14 (d, *J* = 6.7 Hz, 2H), 2.38 (s, 2H), 1.80 (s, 2H), 1.60-1.35 (m, 19H) ppm.

^{13}C NMR (100 MHz, CDCl$_3$) δ 172.01, 165.33, 156.17, 140.83, 129.66, 128.44, 125.14, 78.82, 65.34, 46.95, 40.72, 37.89, 33.79, 28.26, 25.74, 21.29, 19.65 ppm.

HRMS (ESI) m/z [M+H]$^+$: calcd. 401.2435, found 401.2437.

IR (film): 3359, 3046, 2977, 2930, 1716, 1612, 764 cm^{-1}.

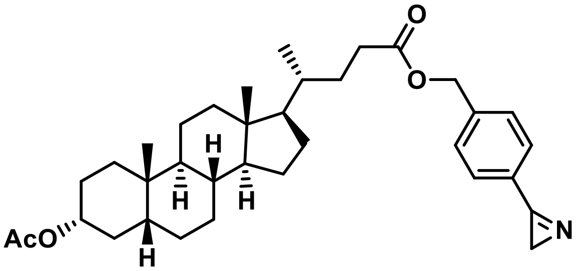

2v

^{1}H NMR (400 MHz, CDCl$_3$) δ 7.91 (d, *J* = 8.0 Hz, 2H), 7.55 (d, *J* = 7.9 Hz, 2H), 5.20 (s, 2H), 4.76-4.68 (m, 1H), 2.48-2.41 (m, 1H), 2.35-2.28 (m, 1H), 2.03 (s, 3H), 1.97-0.91 (m, 34H), 0.63 (s, 3H) ppm.

^{13}C NMR (100 MHz, CDCl$_3$) δ 173.85, 170.58, 165.43, 141.25, 129.72, 128.37, 125.11, 74.32,

65.25, 56.40, 55.89, 42.67, 41.79, 40.31, 40.05, 35.70, 35.26, 34.96, 34.51, 32.17, 31.13, 30.89, 28.14, 26.94, 26.56, 26.25, 24.11, 23.28, 21.45, 20.75, 19.73, 18.20, 11.97 ppm.
HRMS (ESI) m/z [M+H]$^+$: calcd. 548.3734, found 548.3736.
IR (film): 2939, 2867, 1737, 1243, 1160, 824 cm^{-1}.
Optical rotation: $[\alpha]_D^{25}$ = +33.63 (*c* = 1.115, CH_2Cl_2).

Preparation of 4a-4c and 4i-4k

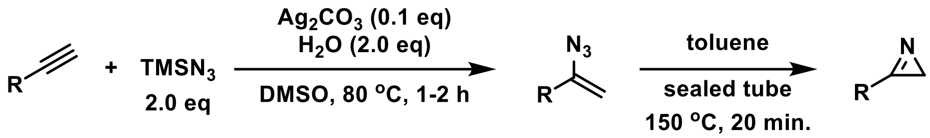

To a solution of alkyne (2.0 mmol), $TMSN_3$ (2.0 equiv) and H_2O (2.0 equiv) in DMSO (4 mL) at 80 ^oC, Ag_2CO_3 (0.1 equiv) was added. The mixture was stirred for 1-2 h. Upon completion as indicated by TLC, the reaction was quenched with water and extracted with diethyl ether (10 mL × 3). The combined organic layers were washed for three times with water, dried over anhydrous Na_2SO_4. The solvent was removed under reduced pressure to give the crude product which was used in next step without further purification.[4]

The crude vinyl azide was heated in toluene (0.1 M) in sealed tube at 150 ^oC for 20 minutes. The reaction mixture was cooled to room temperature and concentrated under reduced pressure to give the crude product, which was purified by flash silica gel column chromatography (petroleum ether /ethyl acetate) to afford 2*H*-azirine.

4a and **4c** were known compounds.[2,3,5] Several new 2*H*-azirines were shown below.

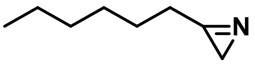

4b

^{1}H NMR (400 MHz, $CDCl_3$) δ 2.79 (t, *J* = 7.3 Hz, 2H), 1.79-1.67 (m, 2H), 1.44-1.28 (m, 8H), 0.89 (t, *J* = 5.7 Hz, 3H) ppm.
^{13}C NMR (100 MHz, $CDCl_3$) δ 169.77, 31.37, 28.85, 28.38, 24.03, 22.42, 18.76, 13.97 ppm.
HRMS (ESI) m/z [M+H]$^+$: calcd. 126.1277, found 126.1277.
IR (film): 3039, 2932, 2860, 1766, 1466, 1421, 986, 726 cm^{-1}.

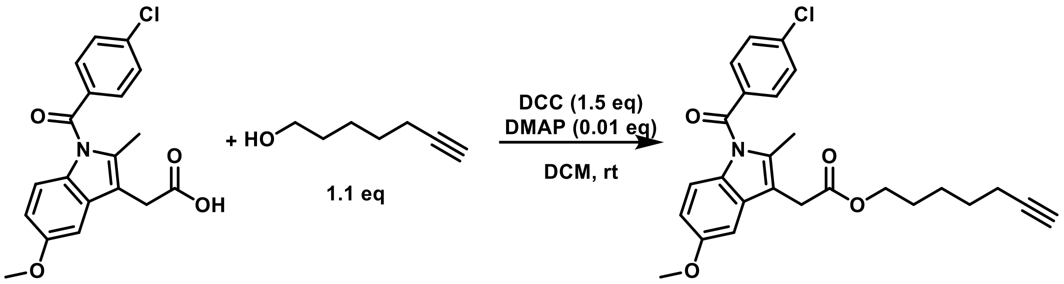

To a stirred solution of indometacin (10 mmol, 1.0 equiv) and hept-6-yn-1-ol (1.1 equiv) in DCM (20 mL) were added DCC (1.5 equiv) and DMAP (0.01 equiv) at room temperature. The reaction mixture was stirred overnight. The precipitate was filtered off and washed with CH_2Cl_2. After removal of solvent under reduced pressure, the residue was purified by silica gel column chromatography (petroleum ether/ethyl acetate) to afford the alkyne. Following the above method, alkyne can be transformed to corresponding 2*H*-azirine.

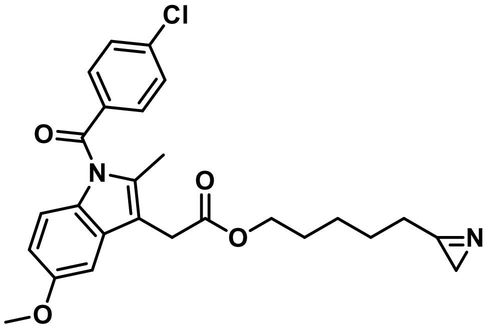

4i

^{1}H NMR (400 MHz, CDCl$_3$) δ 7.66 (d, *J* = 8.3 Hz, 2H), 7.47 (d, *J* = 8.3 Hz, 2H), 6.96 (d, *J* = 2.0 Hz, 1H), 6.87 (d, *J* = 9.0 Hz, 1H), 6.66 (dd, *J* = 9.0, 2.2 Hz, 1H), 4.11 (t, *J* = 6.5 Hz, 2H), 3.83 (s, 3H), 3.66 (s, 2H), 2.73 (t, *J* = 7.2 Hz, 2H), 2.38 (s, 3H), 1.79-1.62 (m, 4H), 1.47-1.32 (m, 4H) ppm.
^{13}C NMR (100 MHz, CDCl$_3$) δ 170.78, 169.42, 168.16, 155.87, 139.13, 135.80, 133.76, 131.06, 130.67, 130.53, 129.01, 114.84, 112.52, 111.39, 101.26, 64.57, 55.58, 30.30, 28.17, 25.43, 23.61, 18.79, 13.28 ppm.
HRMS (ESI) m/z [M+NH$_4$]$^+$: calcd. 484.1998, found 484.1998.
IR (film): 2938, 1733, 1683, 1592, 1166, 1088, 755 cm^{-1}.

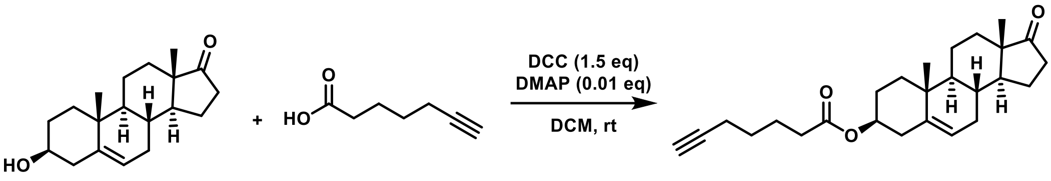

To a stirred solution of hept-6-ynoic acid (10 mmol, 1.0 equiv) and dehydroepiandrosterone (1.1 equiv) in DCM (20 mL) were added DCC (1.5 equiv) and DMAP (0.01 equiv) at room temperature. The reaction mixture was stirred overnight. The precipitate was filtered off and washed with CH_2Cl_2. After removal of solvent under reduced pressure, the residue was purified by silica gel column chromatography (petroleum ether/ethyl acetate) to afford the alkyne. Following the above method, alkyne can be transformed to corresponding 2*H*-azirine.

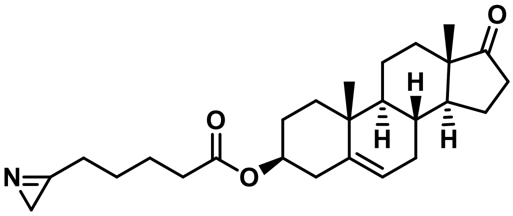

4j

^{1}H NMR (400 MHz, $CDCl_3$) δ 5.34 (s, 1H), 4.61-4.50 (m, 1H), 2.76 (t, *J* = 5.4 Hz, 2H), 2.39 (dd, *J* = 19.2, 8.8 Hz, 1H), 2.32-2.19 (m, 4H), 2.07-1.98 (m, 2H), 1.95-0.91 (m, 23H), 0.82 (s, 3H) ppm.
^{13}C NMR (100 MHz, $CDCl_3$) δ 220.89, 172.43, 169.38, 139.75, 121.81, 73.60, 51.58, 50.01, 47.41, 37.99, 36.81, 36.62, 35.74, 34.04, 31.35, 31.29, 30.67, 28.08, 27.62, 24.41, 23.53, 21.78, 20.22, 19.25, 18.84, 13.45 ppm.
HRMS (ESI) m/z [M+H]$^+$: calcd. 412.2846, found 412.2852.
IR (film): 2944, 1738, 1374, 1174, 990 cm^{-1}.
Optical rotation: $[\alpha]_D^{25}$ = +4.06 (*c* = 1.040, CH_2Cl_2).

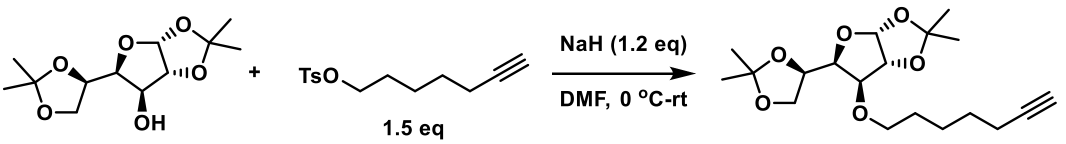

To a stirred solution of diacetone-*D*-glucose (15 mmol, 1.0 equiv) in DMF (20 mL) at 0 °C was added NaH (1.2 equiv, 60% in mineral oil). After 30 minutes, hept-6-yn-1-yl 4-methylbenzenesulfonate (1.5 equiv) was added and the mixture was stirred overnight. The reaction was quenched with water and extracted with ethyl acetate (20 mL × 3). The combined organic layers were washed for three times with water, and dried over anhydrous Na_2SO_4. After removal of solvent under reduced pressure, the residue was purified by silica gel column chromatography (petroleum ether/acetone) to afford the alkyne. Following the above method, alkyne can be transformed to corresponding 2*H*-azirine.

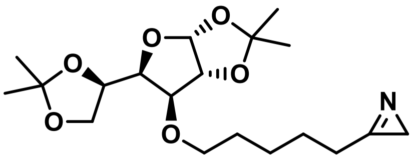

4k

^{1}H NMR (400 MHz, $CDCl_3$) δ 5.87 (d, *J* = 3.5 Hz, 1H), 4.53 (d, *J* = 3.6 Hz, 1H), 4.29 (dd, *J* = 13.4, 6.2 Hz, 1H), 4.14-4.05 (m, 2H), 3.98 (dd, *J* = 8.3, 6.0 Hz, 1H), 3.85 (d, *J* = 2.7 Hz, 1H), 3.65-3.51 (m, 2H), 2.80 (t, *J* = 7.2 Hz, 2H), 1.83-1.73 (m, 2H), 1.67-1.58 (m, 2H), 1.52-1.46 (m, 5H), 1.42 (s, 3H), 1.37 (s, 2H), 1.34 (s, 3H), 1.32 (s, 3H) ppm.
^{13}C NMR (100 MHz, $CDCl_3$) δ 169.55, 111.65, 108.83, 105.16, 82.38, 82.03, 81.05, 72.36, 70.11, 67.18, 29.26, 28.31, 26.75, 26.72, 26.15, 25.65, 25.33, 23.80, 18.80 ppm.
HRMS (ESI) m/z $[M+NH_4]^+$: calcd. 387.2490, found 387.2495.
IR (film): 2985, 2937, 2868, 1765, 1216, 1075, 849 cm^{-1}.
Optical rotation: $[\alpha]_D^{25}$ = -30.84 (*c* = 0.983, CH_2Cl_2).

Optimization of reaction conditions with (R)-DTBM-SEGPHOS

Screening of solvent

Supplementary Table 1. Screening of solvent[a]

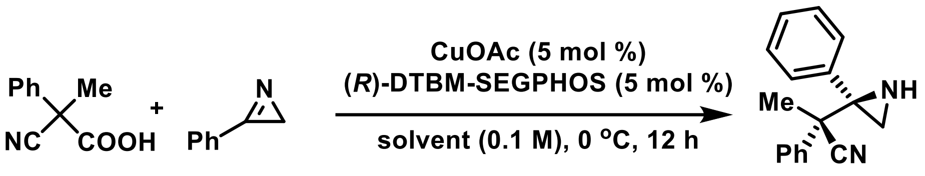

1a 2a 3a

entry	solvent	yield (%)[b]	dr[b]	ee (%)[c]
1	THF	88	4.0/1	93/43
2	2-Me-THF	89	3.0/1	92/50
3[d]	CyH	94	2.2/1	88/52
4	DCE	<5	-	-
5[d]	1,4-dioxane	61	3.0/1	91/44
6	CPME	88	2.7/1	91/50
7	DME	60	3.2/1	90/47
8	MTBE	67	2.2/1	90/53
9	toluene	73	2.4/1	91/57
9	DCM	<5	-	-

[a]**1a**: 0.3 mmol, **2a**: 0.2 mmol. [b]Determined by ^{1}H NMR analysis of reaction crude mixture using CH_3NO_2 as an internal standard. [c]Determined by chiral-stationary-phase HPLC analysis. [d]0 °C to rt.

Screening of temperature

Supplementary Table 2. Screening of temperature[a]

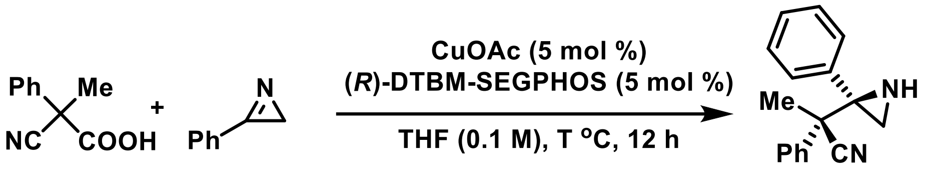

entry	T	yield (%)[b]	dr[b]	ee (%)[c]
**1**	**0**	**88**	**4.0/1**	**93/43**
**2**	**-20**	**quant.**	**5.0/1**	**95/44**
**3**	**-40**	**85**	**6.2/1**	**97/47**

[a]**1a**: 0.3 mmol, **2a**: 0.2 mmol. [b]Determined by ^{1}H NMR analysis of reaction crude mixture using CH_3NO_2 as an internal standard. [c]Determined by chiral-stationary-phase HPLC analysis.

Screening of copper(I) source

Supplementary Table 3. Screening of copper(I) source[a]

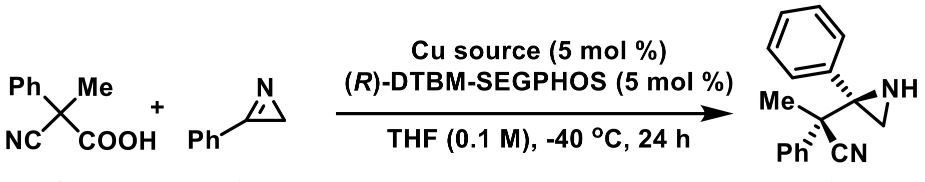

entry	Cu source	yield(%)[b]	dr[b]	ee (%)[c]
**1**	**CuOAc**	**85**	**6.2/1**	**97/47**
**2**	**$Cu(CH_3CN)_4PF_6$**	**95**	**5.6/1**	**96/57**
**3**	**CuCl**	**<5**	**-**	**-**
**4**	**CuOTf**	**96**	**6.3/1**	**96/48**
**5**	**mesitylcopper**	**78**	**6.3/1**	**97/60**

[a]**1a**: 0.3 mmol, **2a**: 0.2 mmol. [b]Determined by ^{1}H NMR analysis of reaction crude mixture using CH_3NO_2 as an internal standard. [c]Determined by chiral-stationary-phase HPLC analysis.

Comparison of the reaction performance between (R)-DTBM-SEGPHOS and (R)-DIPA-MeO-BIPHEP

Comparison of the reaction performance between (R)-DTBM-SEGPHOS and (R)-DIPA-MeO-BIPHEP in the decarboxylative Mannich reaction of aromatic 2H-azirine 2a and 1a

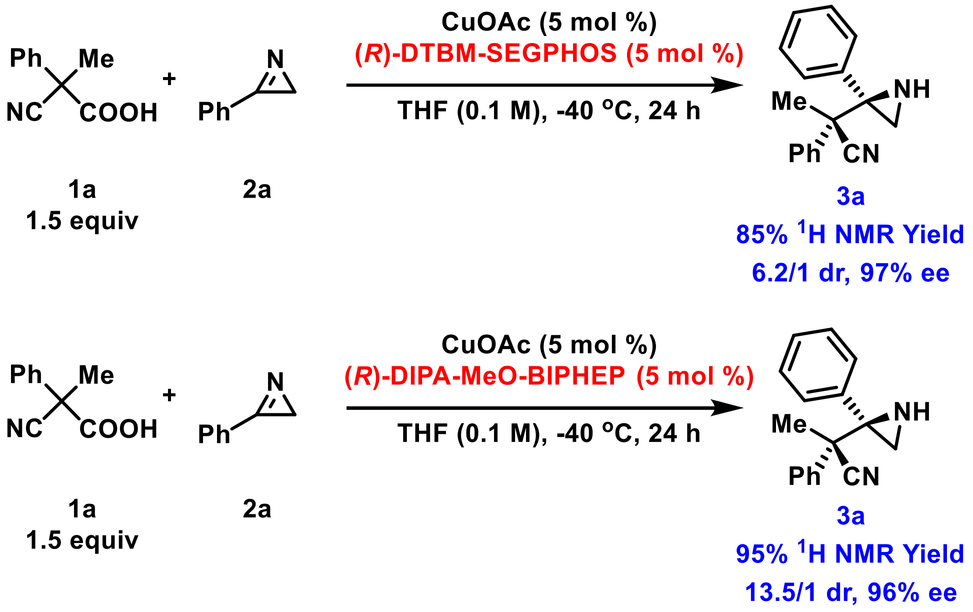

HPLC: DAICEL CHIRALPAK IC, hexane/*i*-PrOH = 4/1, flow rate: 0.5 mL/min, λ = 254 nm.

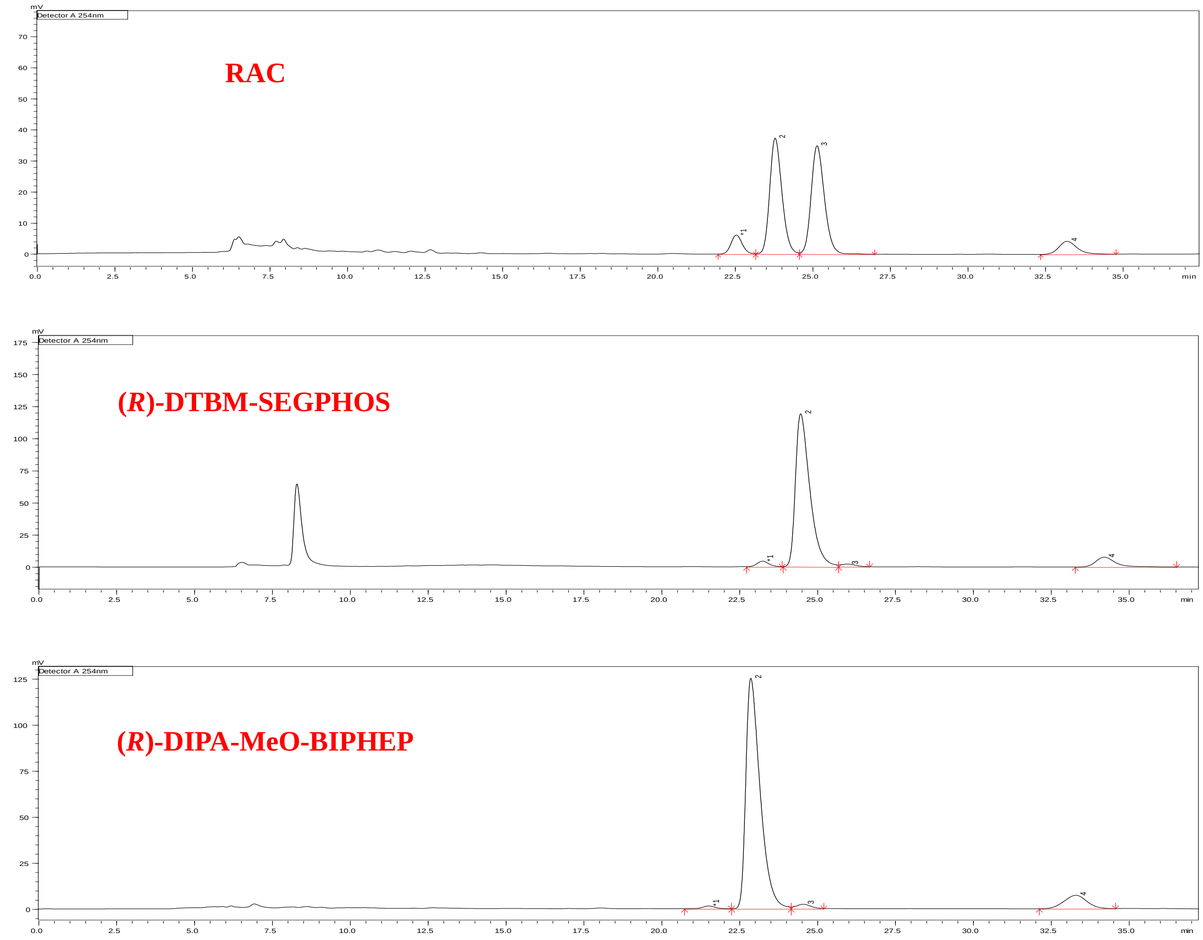

Peak#	Ret. Time	Area%
1	22.543	6.592
2	23.794	43.405
3	25.145	43.393
4	33.205	6.610

Peak#	Ret. Time	Area%
1	23.243	2.795
2	24.467	87.838
3	25.975	1.570
4	34.211	7.797

Peak#	Ret. Time	Area%
1	21.514	0.960
2	22.867	88.942
3	24.554	1.824
4	33.300	8.274

Supplementary Figure 1. HPLC chromatogram for compound **3a**

According to the HPLC spectra, the absolute configurations of **3a** with (*R*)-DTBM-SEGPHOS and (*R*)-DIPA-MeO-BIPHEP were identical.

Comparison of the reaction performance between (R)-DTBM-SEGPHOS and (R)-DIPA-MeO-BIPHEP in the decarboxylative Mannich reaction of aliphatic 2H-azirine 4a and 1a

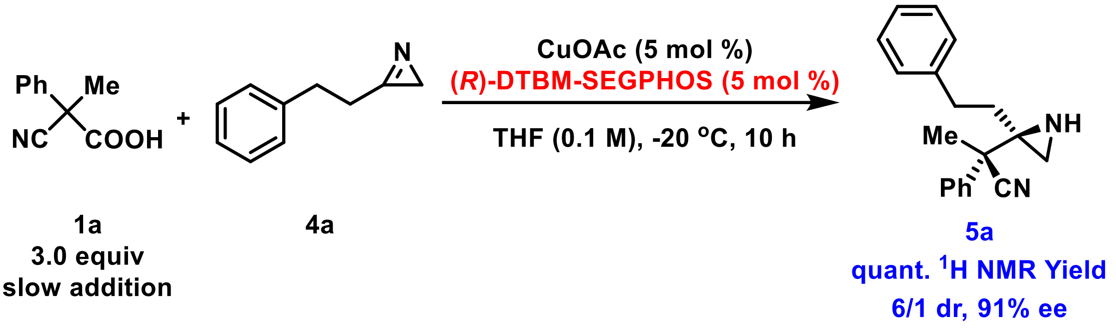

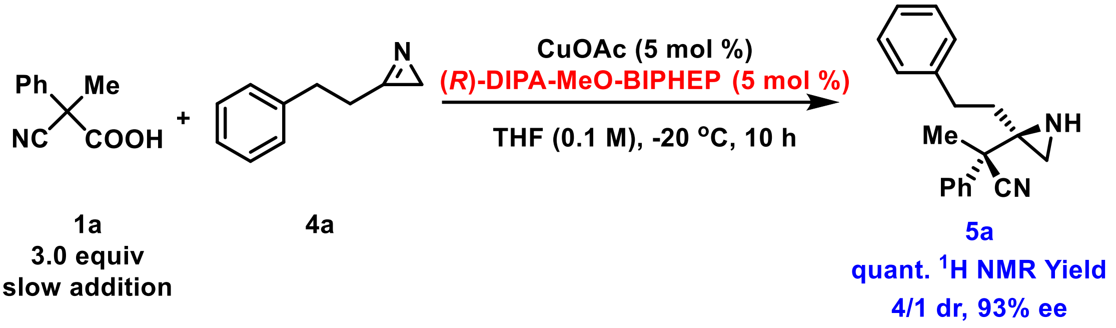

HPLC: DAICEL CHIRALPAK IA connected with DAICEL CHIRALPAK ODH in series, hexane/*i*-PrOH = 4/1, flow rate: 0.5 mL/min, λ = 207 nm.

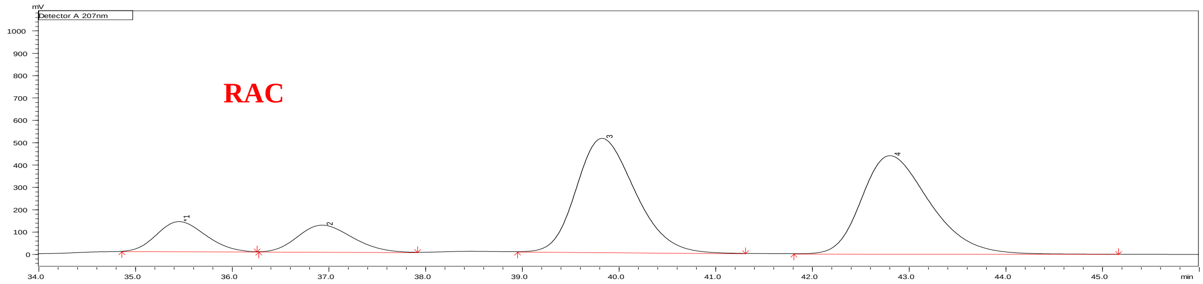

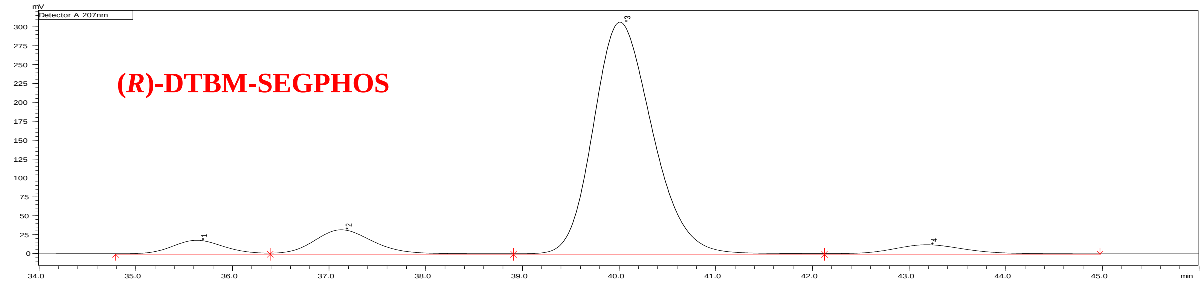

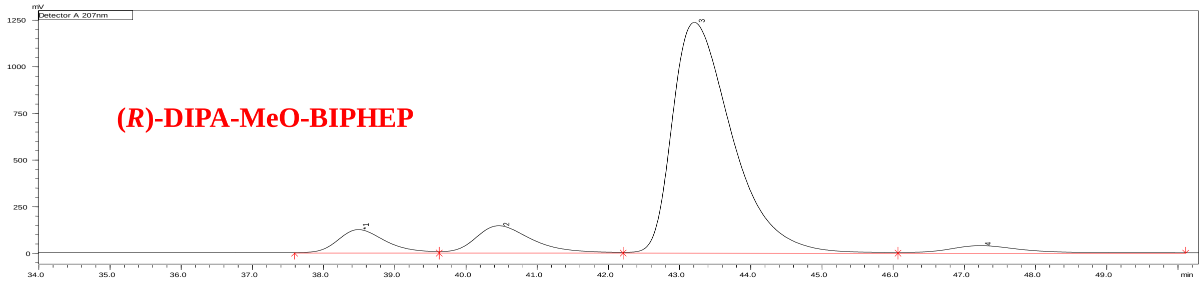

Peak#	Ret. Time	Area%
1	35.454	8.906
2	36.935	8.915
3	39.829	41.128
4	42.806	41.052

Peak#	Ret. Time	Area%
1	35.635	4.236
2	37.132	8.521
3	40.013	83.337
4	43.187	3.907

Peak#	Ret. Time	Area%
1	38.496	6.510
2	40.467	8.948
3	43.214	81.666
4	47.232	2.875

Supplementary Figure 2. HPLC chromatogram for compound **5a**

According to the HPLC spectra, the absolute configurations of **5a** with (*R*)-DTBM-SEGPHOS and (*R*)-DIPA-MeO-BIPHEP were identical.

Copper(I)-catalyzed asymmetric decarboxylative Mannich reaction of 2H-azirines and cyanoacetic acids

General procedure for Copper(I)-catalyzed asymmetric decarboxylative Mannich reaction of aromatic 2H-azirines and cyanoacetic acid 1a

Procedure A:

Preparation of catalyst solution: A dried 25 mL Schlenk tube equipped with a magnetic stirring bar was charged with CuOAc (2.94 mg, 0.024 mmol) and (*R*)-DIPA-MeO-BIPHEP (26.2 mg, 0.024 mmol) in a glove box under Ar atmosphere. Anhydrous THF (4 mL) was added via a syringe. The mixture was stirred for 15 minutes to give a clear catalyst solution.

A dried 25 mL Schlenk tube equipped with a magnetic stirring bar was charged with aromatic 2*H*-azirines **2** (0.2 mmol, 1.0 equiv). The catalyst solution (1.0 mL) containing copper(I) complex (0.006 mmol, 0.03 equiv) was added via a syringe. The reaction mixture was then cooled to -60 °C and cyanoacetic acid **1a** (0.3 M in THF, 1.0 mL, 0.3 mmol, 1.5 equiv) was added dropwise over 2 minutes. The resulting reaction mixture was stirred at -60 °C for indicated time (36-90 h). The reaction mixture was purified by silica gel column chromatography (petroleum ether/ethyl acetate) to give the product.

Procedure B:

A dried 25 mL Schlenk tube equipped with a magnetic stirring bar was charged with CuOAc (1.5 mg, 0.012 mmol, 0.06 equiv) and (*R*)-DIPA-MeO-BIPHEP (13.1 mg, 0.012 mmol, 0.06 equiv) in a glove box under Ar atmosphere. Anhydrous THF (1 mL) was added via a syringe. The mixture was stirred for 15 minutes to give a clear catalyst solution. Then aromatic 2*H*-azirines **2** (0.2 mmol, 1.0 equiv) was added. The reaction mixture was then cooled to -60 °C and cyanoacetic acid **1a** (0.6 M in THF, 1.0 mL, 0.6 mmol, 3.0 equiv) was added dropwise over 2 minutes. The resulting reaction mixture was stirred at -60 °C for 80 h. The reaction mixture was purified by silica gel column chromatography (petroleum ether/ethyl acetate) to give the product.

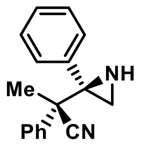

3a

3a: Procedure A, 45.7 mg, pale yellow solid, 92% yield, >20/1 dr.
^{1}H NMR (400 MHz, $CDCl_3$) δ 7.33-7.26 (m, 5H), 7.25-7.21 (m, 1H), 7.14 (t, J = 7.3 Hz, 2H), 6.98 (d, J = 7.4 Hz, 2H), 2.36 (brs, 1H), 2.03 (brs, 1H), 1.79 (s, 3H), 1.12 (brs, 1H) ppm.
^{13}C NMR (100 MHz, $CDCl_3$) δ 138.25, 137.51, 130.07, 128.25, 128.17, 128.11, 127.59, 126.99, 122.17, 47.16, 29.61, 21.87 ppm.
^{1}H NMR (500 MHz, DMSO-d_6-75 ºC) δ 7.32 (s, 5H), 7.20-7.17 (m, 1H), 7.13 (t, J = 7.3 Hz, 2H), 6.99 (d, J = 7.1 Hz, 2H), 2.18 (s, 1H), 1.88 (brs, 2H), 1.69 (s, 3H) ppm.
^{13}C NMR (126 MHz, DMSO-d_6-75 ºC) δ 138.11, 137.76, 129.67, 127.76, 127.53, 127.22, 126.77, 126.41, 122.06, 46.42, 46.19, 28.93, 21.50 ppm.
HRMS (ESI) m/z [M+H]$^{+}$: calcd. 249.1386, found 249.1387.
IR (film): 3298, 3060, 2963, 2236, 1601, 853, 761, 700 cm^{-1}.
Optical rotation: $[\alpha]_D^{25}$ = -7.20 (c = 1.050, CH_2Cl_2, 98% ee).
HPLC: DAICEL CHIRALPAK IC, hexane/i-PrOH = 4/1, flow rate: 0.5 mL/min, λ = 254 nm, t_R(major) = 25.1 min, t_R(minor) = 26.7 min, ee =98 %.

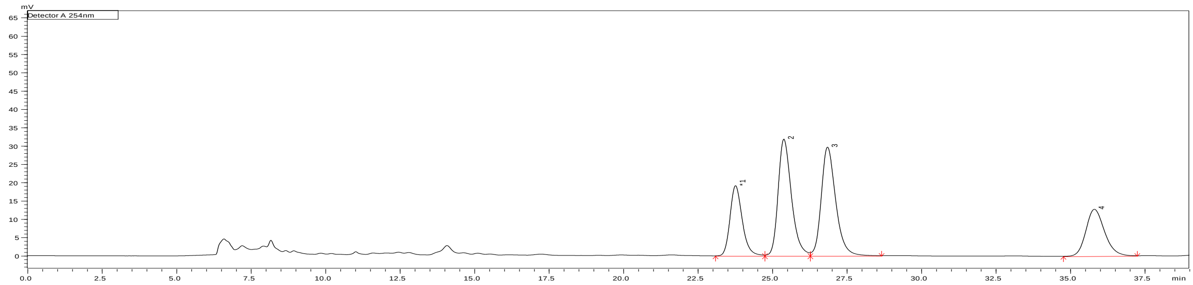

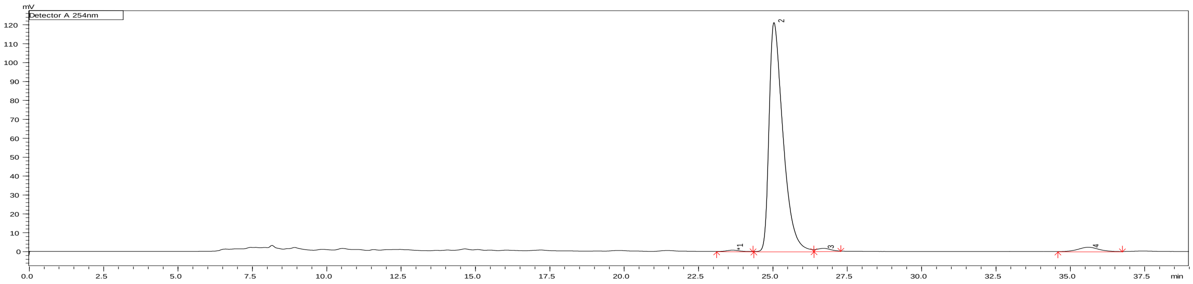

Peak#	Ret. Time	Area%
1	23.767	17.705
2	25.389	32.080
3	26.855	32.318
4	35.811	17.898

Peak#	Ret. Time	Area%
1	23.658	0.449
2	25.050	95.835
3	26.711	1.156
4	35.617	2.559

Supplementary Figure 3. HPLC chromatogram for compound **3a**

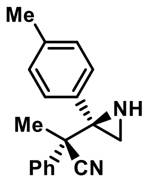

3b

3b: Procedure A, 51.3 mg, pale yellow solid, 98% yield, >20/1 dr.
^{1}H NMR (400 MHz, $CDCl_3$) δ 7.32-7.31 (m, 5H), 6.95 (d, *J* = 7.7 Hz, 2H), 6.85 (d, *J* = 7.4 Hz, 2H), 2.32 (brs, 1H), 2.26 (s, 3H), 1.99 (brs, 1H), 1.77 (s, 3H), 0.92 (brs, 1H) ppm.
^{13}C NMR (100 MHz, $CDCl_3$) δ 137.91, 137.64, 135.33, 129.91, 128.26, 128.21, 128.10, 127.02, 122.27, 47.13, 46.87, 29.83, 21.87, 21.04 ppm.
HRMS (ESI) m/z $[M+H]^+$: calcd. 263.1543, found 263.1548.
IR (film): 3298, 3060, 2923, 2237, 1601, 819, 762, 698 cm^{-1}.
Optical rotation: $[\alpha]_D^{25}$ = -5.07 (*c* = 1.020, CH_2Cl_2, 97% ee).
HPLC: DAICEL CHIRALPAK IA, hexane/*i*-PrOH = 24/1, flow rate: 0.5 mL/min, λ = 207 nm, t_R(major) = 18.6 min, t_R(minor) = 20.2 min, ee = 97%.

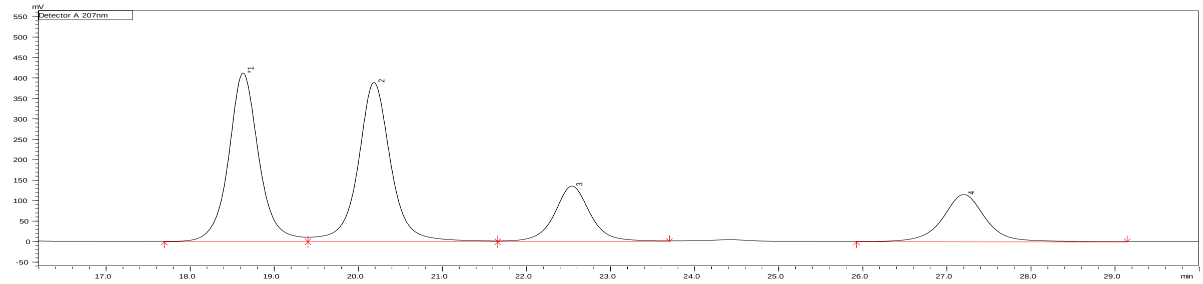

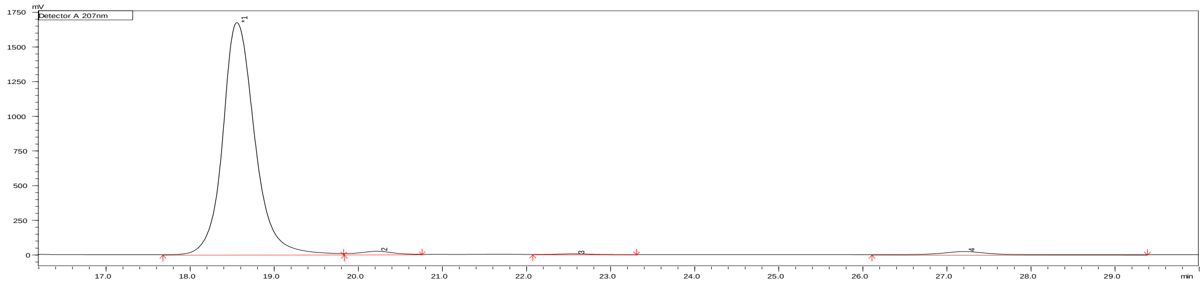

Peak#	Ret. Time	Area%
1	18.634	35.767
2	20.190	36.437
3	22.545	13.851
4	27.205	13.944

Peak#	Ret. Time	Area%
1	18.561	96.602
2	20.224	1.378
3	22.569	0.291
4	27.210	1.729

Supplementary Figure 4. HPLC chromatogram for compound **3b**

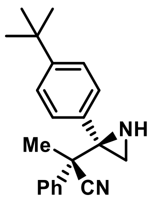

3c

3c: Procedure A, 59.1 mg, pale yellow oil, 97% yield, >20/1 dr.

^{1}H NMR (400 MHz, CDCl$_3$) δ 7.35-7.26 (m, 5H), 7.16 (d, *J* = 8.2 Hz, 2H), 6.92 (d, *J* = 7.4 Hz, 2H), 2.26 (brs, 1H), 1.99 (brs, 1H), 1.78 (s, 3H), 1.25 (s, 9H), 0.92 (brs, 1H) ppm.

^{13}C NMR (100 MHz, CDCl$_3$) δ 151.09, 137.58, 135.31, 129.69, 128.14, 127.09, 124.48, 122.32, 47.11, 46.80, 34.42, 31.14, 29.61, 21.98 ppm.

HRMS (ESI) m/z [M+H]$^+$: calcd. 305.2012, found 305.2013.

IR (film): 3298, 3061, 3031, 2963, 2237, 1601, 833, 744, 698 cm^{-1}.

Optical rotation: $[\alpha]_D^{25}$ = -2.64 (*c* = 1.144, CH_2Cl_2, 98% ee).

HPLC: DAICEL CHIRALPAK IA, hexane/*i*-PrOH = 24/1, flow rate: 0.5 mL/min, λ = 207 nm, t_R(major) = 14.7 min, t_R(minor) = 16.0 min, ee = 98%.

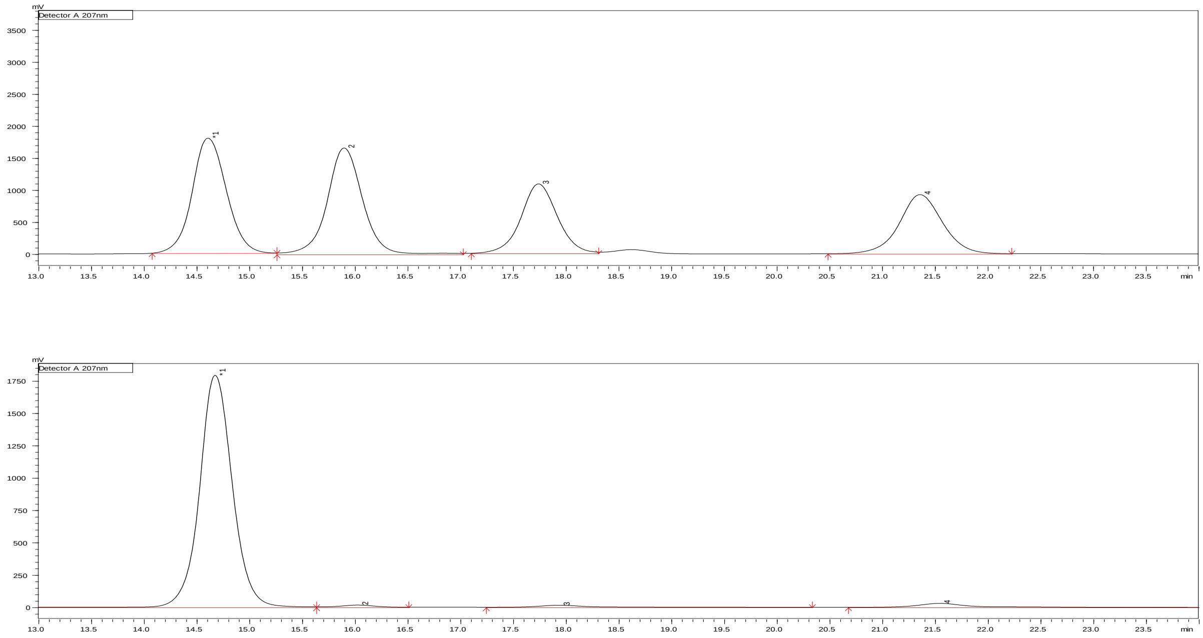

Peak#	Ret. Time	Area%
1	14.608	30.737
2	15.898	29.559
3	17.741	19.778
4	21.359	19.925

Peak#	Ret. Time	Area%
1	14.675	95.005
2	16.028	0.990
3	17.936	1.378
4	21.545	2.627

Supplementary Figure 5. HPLC chromatogram for compound **3c**

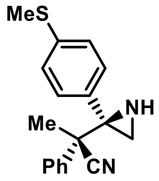

3d

3d: Procedure A, 56.8 mg, pale yellow solid, 96% yield, >20/1 dr.
^{1}H NMR (400 MHz, $CDCl_3$) δ 7.32-7.31 (m, 5H), 6.99 (d, *J* = 7.8 Hz, 2H), 6.87 (d, *J* = 7.8 Hz, 2H), 2.40 (s+brs, 4H), 1.99 (s, 1H), 1.77 (s, 3H), 1.00 (brs, 1H) ppm.
^{13}C NMR (100 MHz, $CDCl_3$) δ 138.73, 137.36, 134.67, 130.36, 128.24, 128.14, 126.91, 125.01, 122.06, 47.13, 46.68, 29.73, 21.74, 15.18 ppm.
HRMS (ESI) m/z [M+H]$^+$: calcd. 295.1263, found 295.1265.
IR (film): 3295, 3060, 2962, 2854, 2236, 1598, 1261, 821, 749, 699 cm^{-1}.
Optical rotation: $[\alpha]_D^{25}$ = +5.13 (*c* = 1.102, CH_2Cl_2, 97% ee).
HPLC: DAICEL CHIRALPAK IA, hexane/*i*-PrOH = 24/1, flow rate: 0.5 mL/min, λ = 207 nm, t_R(major) = 27.5 min, t_R(minor) = 30.2 min, ee = 97%.

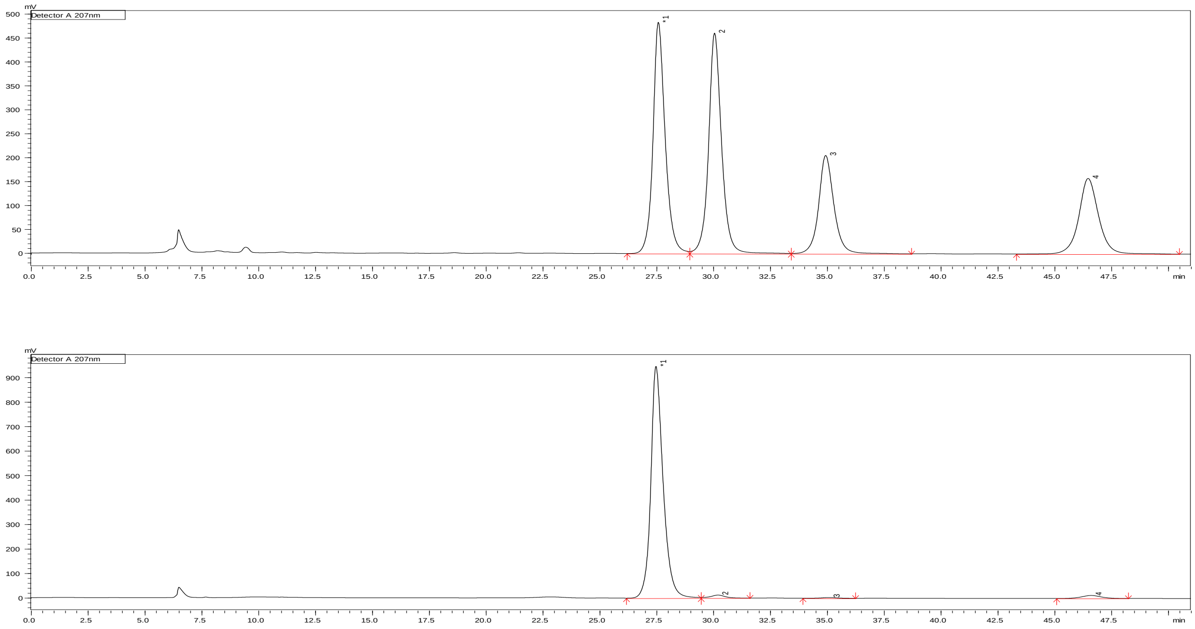

Peak#	Ret. Time	Area%
1	27.585	32.391
2	30.056	32.965
3	34.941	17.320
4	46.480	17.323

Peak#	Ret. Time	Area%
1	27.481	96.144
2	30.206	1.495
3	35.099	0.390
4	46.617	1.971

Supplementary Figure 6. HPLC chromatogram for compound **3d**

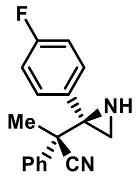

3e

3e: Procedure A, 49.7 mg, pale yellow oil, 93% yield, 12/1 dr.

^{1}H NMR (400 MHz, CDCl$_3$) δ 7.32 (s, 5H), 6.94 (t, J = 5.0 Hz, 2H), 6.83 (t, J = 8.5 Hz, 2H), 2.38 (brs, 1H), 2.00 (brs, 1H), 1.77 (s, 3H), 0.94 (brs, 1H) ppm.

^{13}C NMR (100 MHz, CDCl$_3$) δ 162.18 (d, J = 247.9 Hz), 137.33, 134.05, 131.78 (d, J = 8.2 Hz), 128.36, 128.30, 126.90, 122.02, 114.53 (d, J = 21.6 Hz), 47.08, 46.57, 29.87, 21.71 ppm.

^{19}F NMR (376 MHz, CDCl$_3$) δ -112.88 (s) ppm.

HRMS (ESI) m/z [M+H]$^+$: calcd. 267.1292, found 267.1295.

IR (film): 3297, 3064, 2927, 2237, 1605, 836, 764, 699 cm^{-1}.

Optical rotation: $[\alpha]_D^{25}$ = -15.10 (c = 1.163, CH_2Cl_2, 93% ee).

HPLC: DAICEL CHIRALPAK IA, hexane/*i*-PrOH = 24/1, flow rate: 0.5 mL/min, λ = 207 nm, t_R(major) = 20.4 min, t_R(minor) = 23.0 min, ee = 93%.

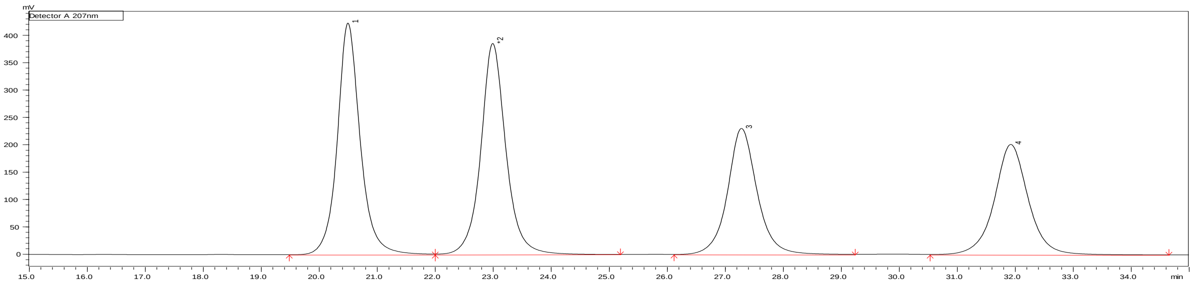

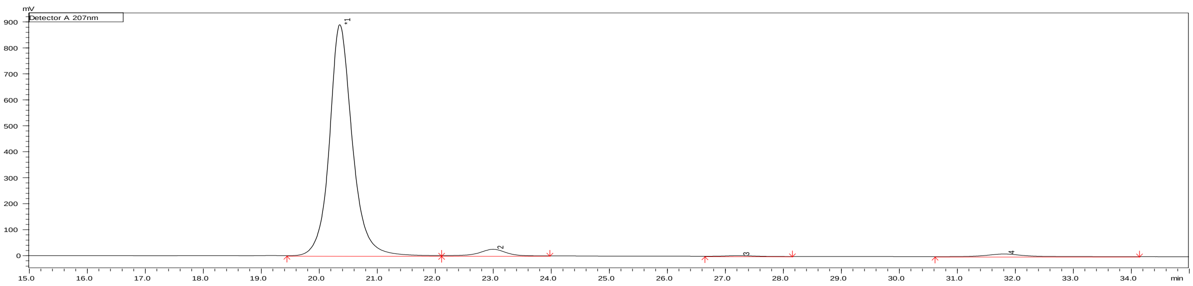

Peak#	Ret. Time	Area%
1	20.500	29.322
2	22.997	29.314
3	27.287	20.666
4	31.930	20.697

Peak#	Ret. Time	Area%
1	20.360	94.717
2	23.003	3.194
3	27.241	0.326
4	31.814	1.763

Supplementary Figure 7. HPLC chromatogram for compound **3e**

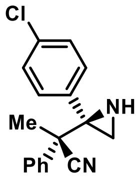

3f

3f: Procedure A, 54.7 mg, white solid, 97% yield, 9/1 dr.

^{1}H NMR (400 MHz, $CDCl_3$) δ 7.32 (s, 5H), 7.12 (d, J = 8.3 Hz, 2H), 6.89 (d, J = 7.4 Hz, 2H), 2.38 (brs, 1H), 2.00 (brs, 1H), 1.77 (s, 3H), 0.91 (brs, 1H) ppm.

^{13}C NMR (100 MHz, $CDCl_3$) δ 137.30, 134.13, 131.39, 128.44, 127.81, 126.92, 121.95, 46.70, 29.85, 21.73 ppm.

HRMS (ESI) m/z [M+H]$^+$: calcd. 283.0997, found 283.0997.

IR (film): 3296, 3063, 2962, 2237, 1598, 828, 749, 699 cm^{-1}.

Optical rotation: $[\alpha]_D^{25}$ = -1.81 (c = 1.007, CH_2Cl_2, 95% ee).

HPLC: DAICEL CHIRALPAK IA, hexane/i-PrOH = 24/1, flow rate: 0.5 mL/min, λ = 207 nm, t_R(major) = 21.4 min, t_R(minor) = 24.2 min, ee = 95%.

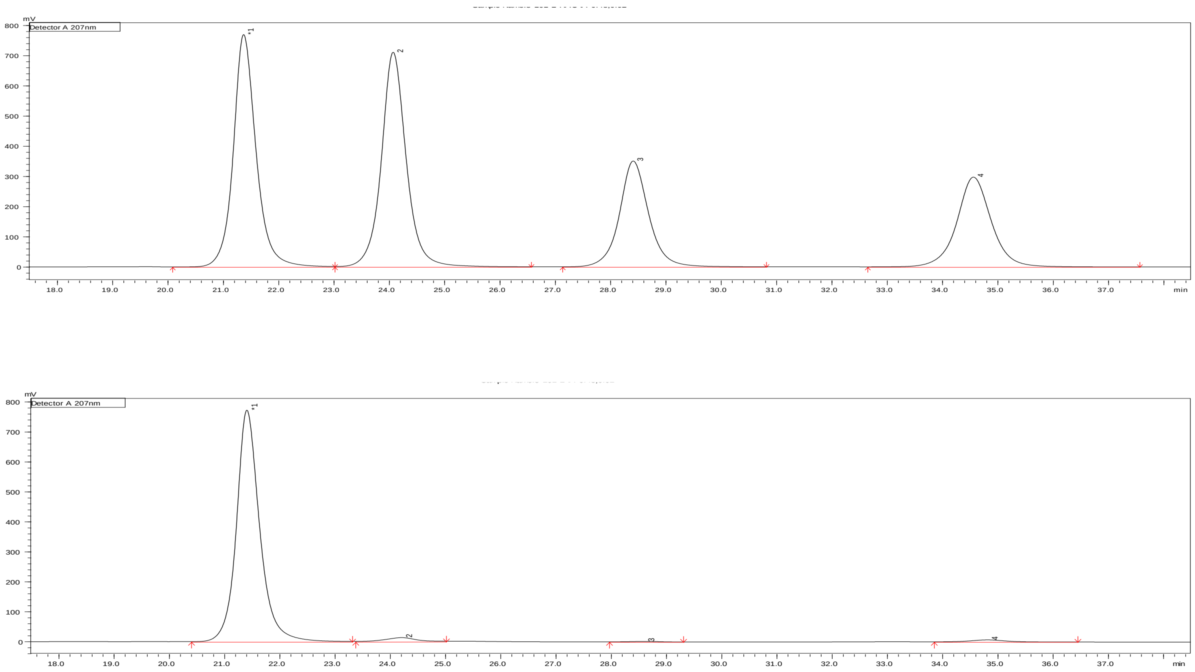

Peak#	Ret. Time	Area%
1	21.373	31.471
2	24.075	31.717
3	28.415	18.375
4	34.565	18.438

Peak#	Ret. Time	Area%
1	21.410	96.141
2	24.211	2.394
3	28.597	0.222
4	34.817	1.242

Supplementary Figure 8. HPLC chromatogram for compound **3f**

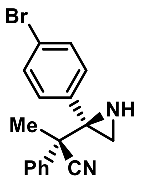

3g

3g: Procedure A, 59.2 mg, pale yellow oil, 91% yield, 8/1 dr.
^{1}H NMR (400 MHz, $CDCl_3$) δ 7.32 (s, 5H), 7.27 (d, *J* = 8.0 Hz, 2H), 6.83 (d, *J* = 7.2 Hz, 2H), 2.38 (brs, 1H), 2.00 (brs, 1H), 1.77 (s, 3H), 0.90 (brs, 1H) ppm.
^{13}C NMR (100 MHz, $CDCl_3$) δ 137.26, 131.70, 130.76, 128.44, 126.91, 122.36, 121.91, 46.75, 29.77, 21.71 ppm.
HRMS (ESI) m/z [M+H]$^+$: calcd. 327.0491, found 327.0490.
IR (film): 3297, 3062, 2926, 2237, 1591, 825, 746, 699 cm^{-1}.
Optical rotation: $[\alpha]_D^{25}$ = +1.62 (*c* = 1.198, CH_2Cl_2, 96% ee).
HPLC: DAICEL CHIRALPAK IC, hexane/*i*-PrOH = 4/1, flow rate: 0.5 mL/min, λ = 207 nm, t_R(major) = 25.0 min, t_R(minor) = 21.9 min, ee = 96%.

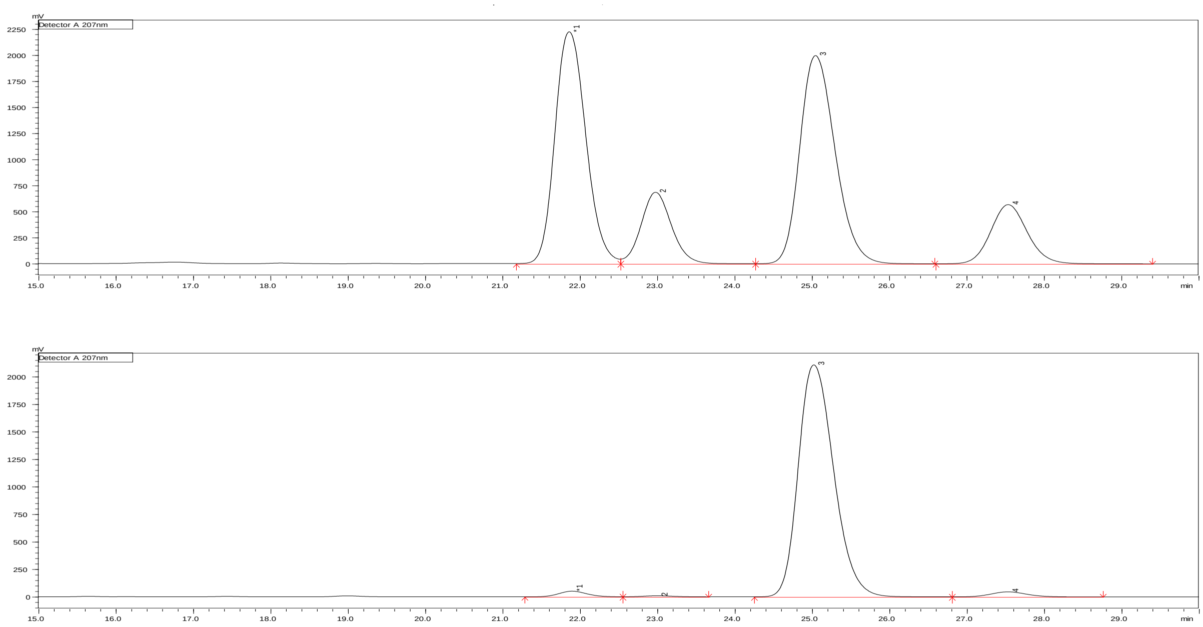

Peak#	Ret. Time	Area%
1	21.862	37.978
2	22.979	11.539
3	25.047	38.992
4	27.538	11.490

Peak#	Ret. Time	Area%
1	21.898	1.870
2	22.997	0.375
3	25.023	95.613
4	27.531	2.142

Supplementary Figure 9. HPLC chromatogram for compound **3g**

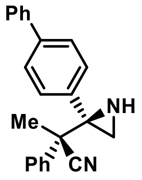

3h

3h: Procedure A, 63.5 mg, white solid, 98% yield, >20/1 dr.

^{1}H NMR (400 MHz, $CDCl_3$) δ 7.50 (d, *J* = 7.2 Hz, 2H), 7.41-7.29 (m, 10H), 7.03 (d, *J* = 7.9 Hz, 2H), 2.37 (brs, 1H), 2.02 (brs, 1H), 1.80 (s, 3H), 1.04 (brs, 1H) ppm.

^{13}C NMR (100 MHz, $CDCl_3$) δ 140.79, 140.05, 137.42, 137.09, 130.44, 128.67, 128.26, 128.18, 127.43, 127.00, 126.87, 126.15, 122.16, 47.09, 46.91, 29.85, 21.83 ppm.

HRMS (ESI) m/z [M+H]$^+$: calcd. 325.1699, found 325.1700.

IR (film): 3299, 3059, 2998, 2237, 1599, 838, 735, 698 cm^{-1}.

Optical rotation: $[\alpha]_D^{25}$ = +15.77 (*c* = 1.052, CH_2Cl_2, 98% ee).

HPLC: DAICEL CHIRALPAK IC, hexane/*i*-PrOH = 4/1, flow rate: 0.5 mL/min, λ = 254 nm, t_R(major) = 34.6 min, t_R(minor) = 30.9 min, ee = 98%.

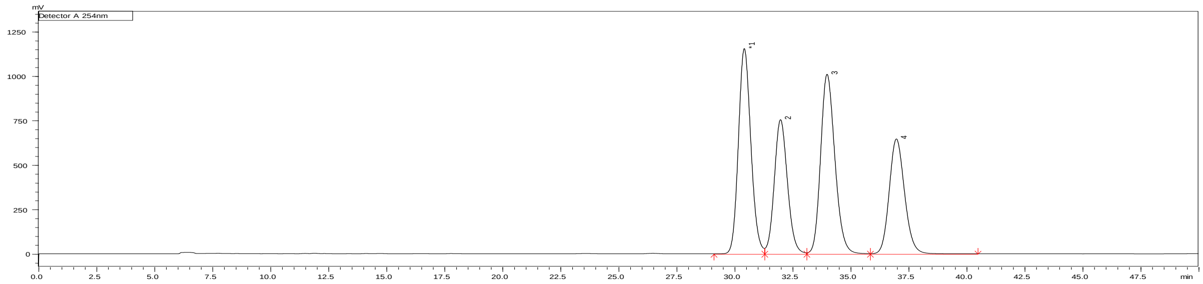

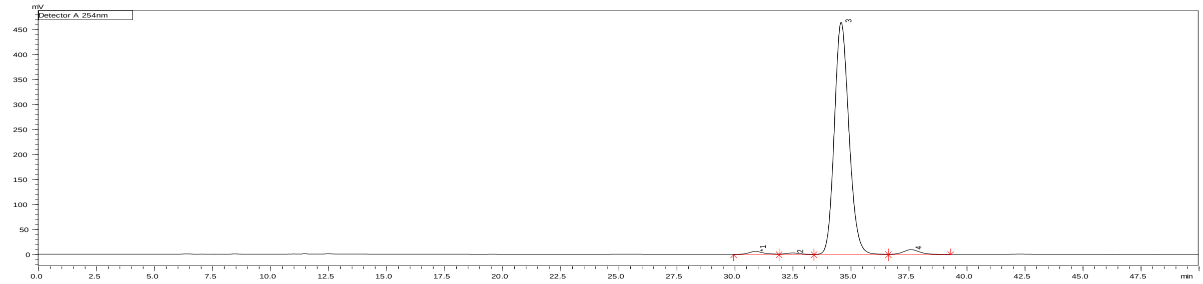

Peak#	Ret. Time	Area%
1	30.421	29.571
2	31.983	20.376
3	33.989	29.675
4	36.977	20.377

Peak#	Ret. Time	Area%
1	30.907	1.186
2	32.507	0.524
3	34.593	96.168
4	37.609	2.122

Supplementary Figure 10. HPLC chromatogram for compound **3h**

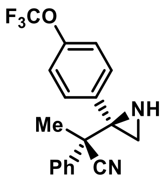

3i

3i: Procedure A, 59.0 mg, colorless oil, 89% yield, 5/1 dr.

^{1}H NMR (400 MHz, $CDCl_3$) δ 7.33 (s, 5H), 7.00 (s, 4H), 2.39 (brs, 1H), 2.03 (brs, 1H), 1.78 (s, 3H), 0.98 (brs, 1H) ppm.

^{13}C NMR (100 MHz, $CDCl_3$) δ 148.80 (q, *J* = 1.8 Hz), 137.14, 131.59, 128.46, 126.93, 121.94, 120.25 (q, *J* = 257.6 Hz), 119.94, 47.00, 46.66, 29.82, 21.74 ppm.

^{19}F NMR (376 MHz, $CDCl_3$) δ -57.92 (s) ppm.

HRMS (ESI) m/z [M+H]$^+$: calcd. 333.1209, found 333.1209.

IR (film): 3297, 3065, 2999, 2927, 2238, 1602, 846, 768, 699 cm^{-1}.

Optical rotation: $[\alpha]_D^{25}$ = -6.00 (*c* = 1.138, CH_2Cl_2, 97% ee).

HPLC: DAICEL CHIRALPAK IA, hexane/*i*-PrOH = 24/1, flow rate: 0.5 mL/min, λ = 207 nm, t_R(major) = 17.4 min, t_R(minor) = 20.2 min, ee = 97%.

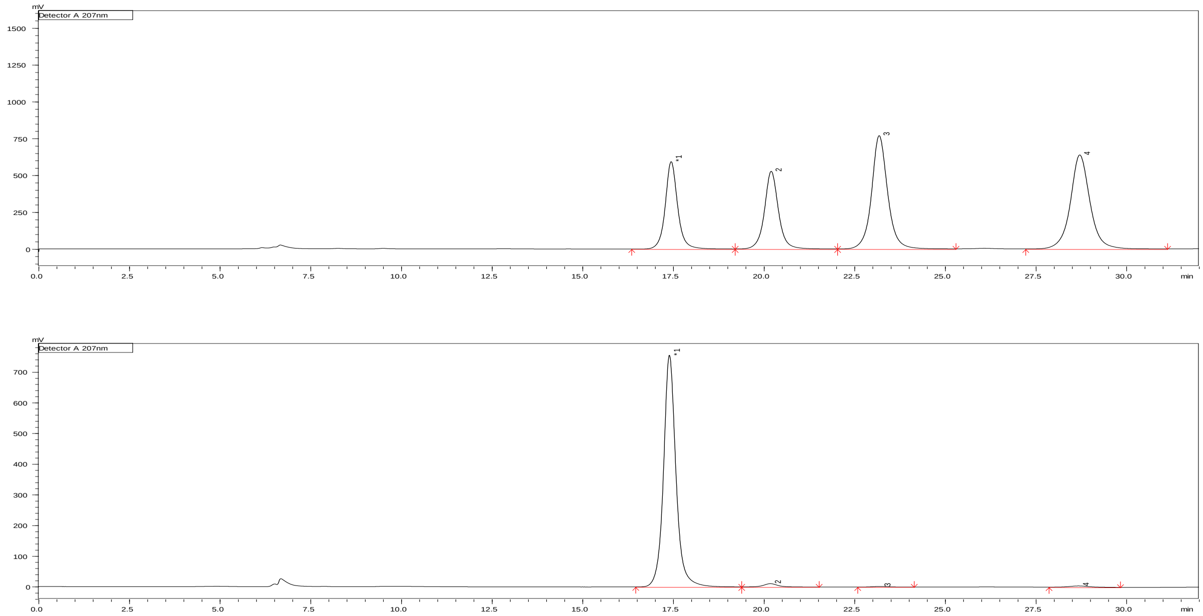

Peak#	Ret. Time	Area%
1	17.447	18.514
2	20.207	18.617
3	23.183	31.286
4	28.715	31.582

Peak#	Ret. Time	Area%
1	17.399	97.174
2	20.185	1.666
3	23.207	0.316
4	28.682	0.845

Supplementary Figure 11. HPLC chromatogram for compound **3i**

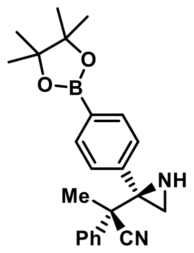

3j

3j: Procedure A, 61.8 mg, white solid, 83% yield, >20/1 dr.
^{1}H NMR (400 MHz, CDCl$_3$) δ 7.58 (d, *J* = 7.5 Hz, 2H), 7.30 (s, 5H), 6.96 (d, *J* = 7.5 Hz, 2H), 2.39 (brs, 1H), 2.01 (brs, 1H), 1.78 (s, 3H), 1.31 (s, 12H), 1.02 (brs, 1H) ppm.
^{13}C NMR (100 MHz, CDCl$_3$) δ 140.97, 137.38, 133.93, 129.39, 128.30, 128.17, 126.94, 122.05, 83.82, 47.25, 47.08, 29.88, 24.75, 21.77 ppm.
HRMS (ESI) m/z [M+H]$^+$: calcd. for $[C_{23}H_{28}O_2N_2{}^{10}B]^+$ 374.2275 , found 374.2274.
IR (film): 3296, 2979, 2931, 2237, 1612, 1362, 1143, 836, 752, 699 cm^{-1}.
Optical rotation: $[\alpha]_D^{25}$ = +11.20 (*c* = 1.073, CH_2Cl_2, 96% ee).
HPLC: DAICEL CHIRALPAK IA, hexane/*i*-PrOH = 24/1, flow rate: 0.5 mL/min, λ = 207 nm, t_R(major) = 17.7 min, t_R(minor) = 20.2 min, ee = 96%.

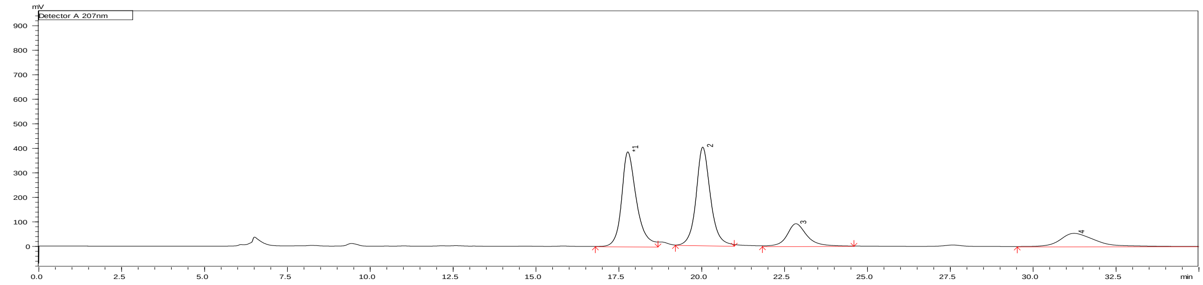

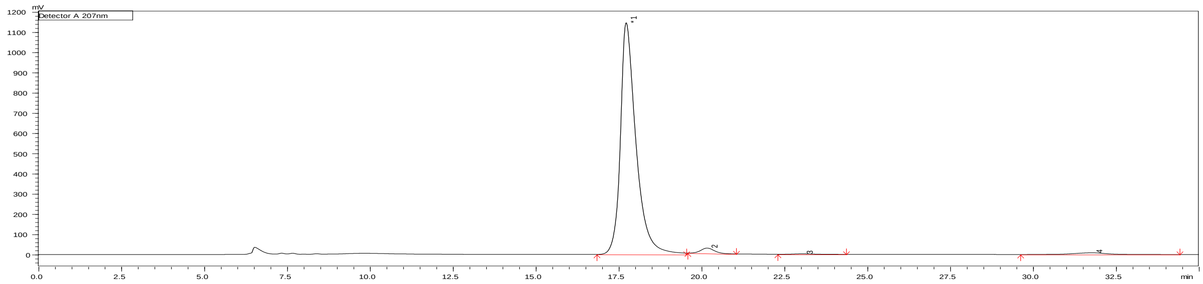

Peak#	Ret. Time	Area%
1	17.782	37.962
2	20.042	37.955
3	22.854	12.019
4	31.245	12.063

Peak#	Ret. Time	Area%
1	17.728	96.265
2	20.168	1.948
3	23.039	0.253
4	31.777	1.534

Supplementary Figure 12. HPLC chromatogram for compound **3j**

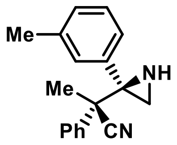

3k

3k: Procedure A, 52.2 mg, colorless oil, 99% yield, >20/1 dr.
^{1}H NMR (400 MHz, $CDCl_3$) δ 7.31 (s, 5H), 7.05-7.02 (m, 2H), 6.76 (s, 2H), 2.35 (brs, 1H), 2.19 (s, 3H), 2.03 (brs, 1H), 1.78 (s, 3H), 1.07 (brs, 1H) ppm.
^{13}C NMR (100 MHz, $CDCl_3$) δ 138.03, 137.56, 137.18, 130.85, 128.78, 128.15, 128.12, 127.41, 127.08, 127.03, 122.20, 47.14, 29.79, 21.81, 21.14 ppm.
HRMS (ESI) m/z [M+H]$^+$: calcd. 263.1543, found 263.1546.
IR (film): 3298, 3060, 2961, 2237, 1603, 790, 761, 699 cm^{-1}.
Optical rotation: $[\alpha]_D^{25}$ = -3.93 (c = 1.020, CH_2Cl_2, 97% ee).
HPLC: DAICEL CHIRALPAK IA, hexane/*i*-PrOH = 24/1, flow rate: 0.5 mL/min, λ = 207 nm, t_R(major) = 16.7 min, t_R(minor) = 19.5 min, ee = 97%.

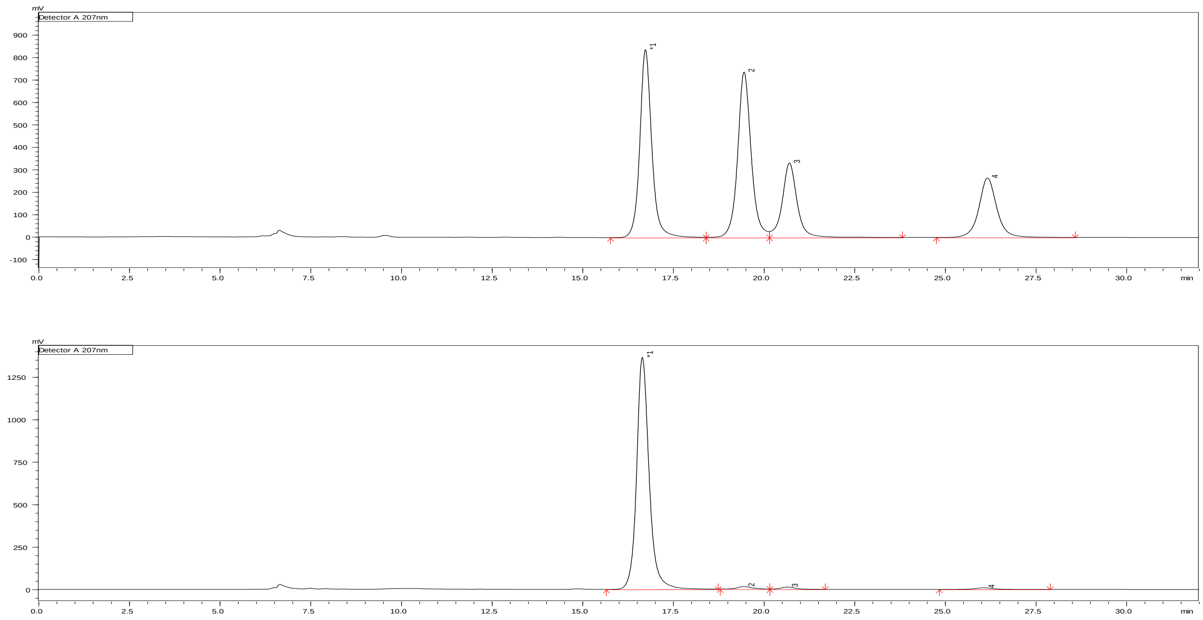

Peak#	Ret. Time	Area%
1	16.737	33.827
2	19.458	33.643
3	20.708	16.571
4	26.171	15.959

Peak#	Ret. Time	Area%
1	16.652	96.564
2	19.454	1.307
3	20.660	1.056
4	26.071	1.072

Supplementary Figure 13. HPLC chromatogram for compound **3k**

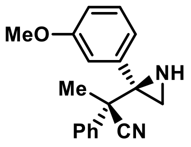

3l

3l: Procedure A, 55.0 mg, pale yellow oil, 99% yield, >20/1 dr.

^{1}H NMR (400 MHz, CDCl$_3$) δ 7.36-7.30 (m, 5H), 7.06 (t, J = 7.8 Hz, 1H), 6.74 (d, J = 8.2 Hz, 1H), 6.63 (d, J = 7.2 Hz, 1H), 6.41 (s, 1H), 3.58 (s, 3H), 2.37 (brs, 1H), 2.03 (brs, 1H), 1.79 (s, 3H), 1.12 (brs, 1H) ppm.

^{13}C NMR (100 MHz, CDCl$_3$) δ 158.52, 139.52, 137.59, 128.56, 128.23, 128.12, 126.98, 122.21, 122.14, 115.27, 114.20, 54.95, 47.14, 29.85, 21.75 ppm.

HRMS (ESI) m/z [M+H]$^+$: calcd. 279.1492, found 279.1491.

IR (film): 3299, 3061, 2942, 2237, 1600, 1138, 761, 700 cm^{-1}.

Optical rotation: $[\alpha]_D^{25}$ = -9.34 (c = 1.072, CH_2Cl_2, 97% ee).

HPLC: DAICEL CHIRALPAK ODH connected with DAICEL CHIRALPAK IA in series, hexane/*i*-PrOH = 4/1, flow rate: 0.5 mL/min, λ = 207 nm, t_R(major) = 28.9 min, t_R(minor) = 30.1 min, ee = 97%.

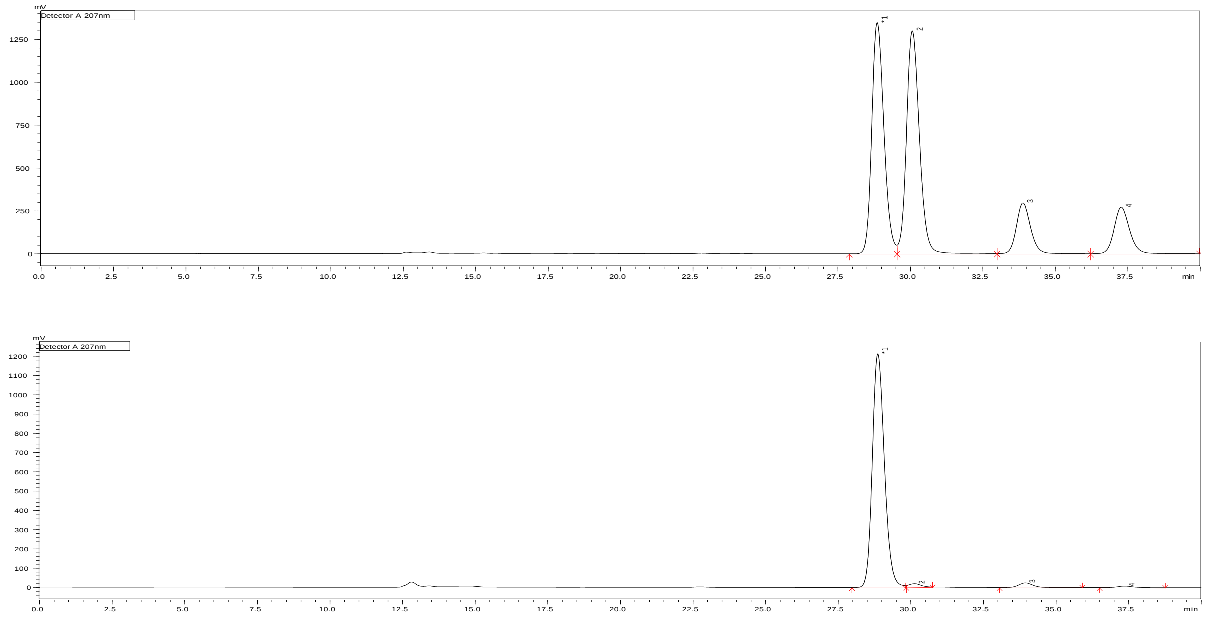

Peak#	Ret. Time	Area%
1	28.866	39.377
2	30.077	40.520
3	33.895	10.061
4	37.287	10.042

Peak#	Ret. Time	Area%
1	28.875	95.477
2	30.140	1.459
3	33.958	2.287
4	37.376	0.777

Supplementary Figure 14. HPLC chromatogram for compound **3l**

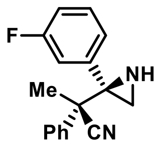

3m

3m: Procedure A, 53.0 mg, pale yellow oil, 96% yield, 10/1 dr.

^{1}H NMR (400 MHz, $CDCl_3$) δ 7.33 (s, 5H), 7.12 (dd, *J* = 14.0, 7.7 Hz, 1H), 6.91 (td, *J* = 8.2, 1.5 Hz, 1H), 6.80 (d, *J* = 7.5 Hz, 1H), 6.65 (d, *J* = 9.0 Hz, 1H), 2.38 (brs, 1H), 2.04 (brs, 1H), 1.79 (s, 3H), 1.14 (brs, 1H) ppm.

^{13}C NMR (100 MHz, $CDCl_3$) δ 161.66 (d, *J* = 246.9 Hz), 140.54, 137.16, 129.16 (d, *J* = 8.2 Hz), 128.42, 127.02, 126.89, 125.76, 121.92, 117.17 (d, *J* = 22.0 Hz), 115.21 (d, *J* = 20.9 Hz), 46.98, 46.89, 29.92, 21.79 ppm.

^{19}F NMR (376 MHz, $CDCl_3$) δ -112.88 (s) ppm.

HRMS (ESI) m/z $[M+H]^+$: calcd. 267.1292, found 267.1292.

IR (film): 3297, 3064, 2962, 2237, 1613, 791, 733, 698 cm^{-1}.

Optical rotation: $[\alpha]_D^{25}$ = -2.03 (*c* = 0.988, CH_2Cl_2, 96% ee).

HPLC: DAICEL CHIRALPAK IA, hexane/*i*-PrOH = 24/1, flow rate: 0.5 mL/min, λ = 207 nm, t_R(major) = 20.5 min, t_R(minor) = 22.5 min, ee = 96%.

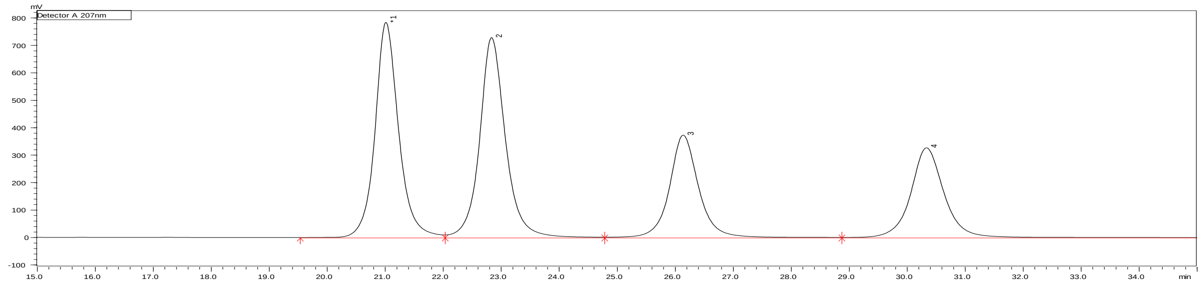

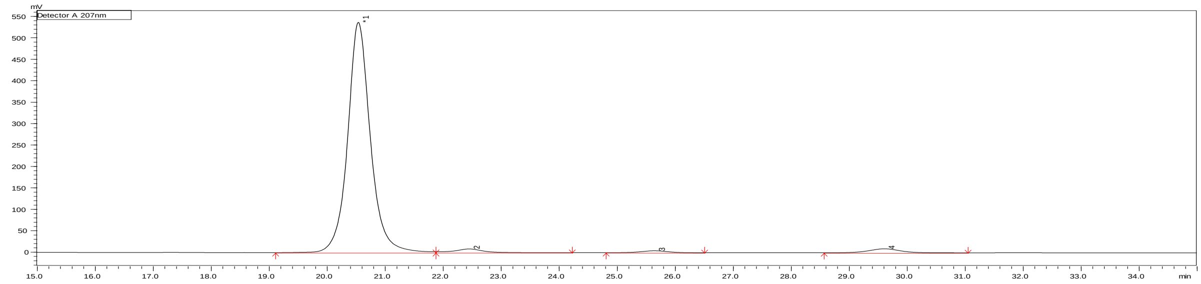

Peak#	Ret. Time	Area%
1	21.018	31.317
2	22.840	31.832
3	26.144	18.438
4	30.339	18.413

Peak#	Ret. Time	Area%
1	20.542	94.819
2	22.456	1.941
3	25.648	0.954
4	29.610	2.287

Supplementary Figure 15. HPLC chromatogram for compound **3m**

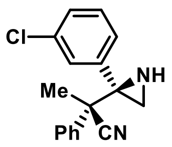

3n

3n: Procedure A, 55.7 mg, pale yellow oil, 98% yield, 8/1 dr.

^{1}H NMR (400 MHz, $CDCl_3$) δ 7.35-7.33 (m, 5H), 7.19 (d, *J* = 7.7 Hz, 1H), 7.08 (t, *J* = 7.8 Hz, 1H), 6.92-6.85 (m, 2H), 2.39 (brs, 1H), 2.02 (brs, 1H), 1.77 (s, *J* = 9.4 Hz, 3H), 1.11 (brs, 1H) ppm.

^{13}C NMR (100 MHz, $CDCl_3$) δ 139.97, 137.04, 133.34, 130.23, 128.85, 128.41, 128.34, 128.23, 126.88, 121.82, 46.89, 29.83, 21.68 ppm.

HRMS (ESI) m/z [M+H]$^+$: calcd. 283.0997, found 283.1000.

IR (film): 3297, 3063, 2927, 2238, 1596, 786, 737, 707 cm^{-1}.

Optical rotation: $[\alpha]_D^{25}$ = -2.38 (*c* = 1.062, CH_2Cl_2, 96% ee).

HPLC: DAICEL CHIRALPAK IA, hexane/*i*-PrOH = 24/1, flow rate: 0.5 mL/min, λ = 207 nm, t_R(major) = 19.1 min, t_R(minor) = 21.8 min, ee = 96%.

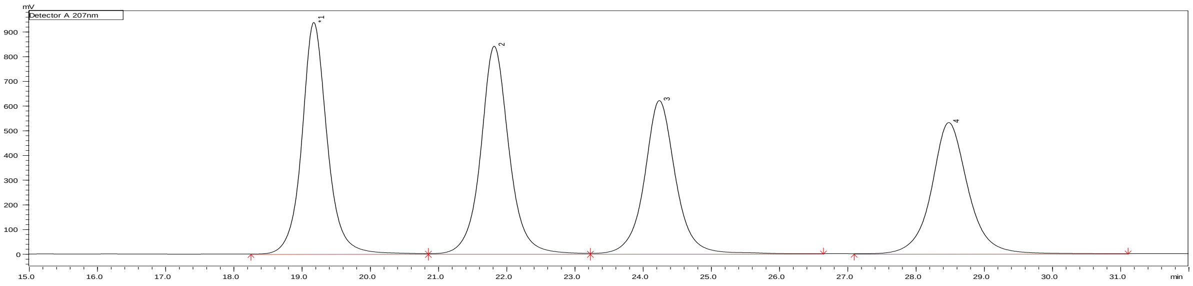

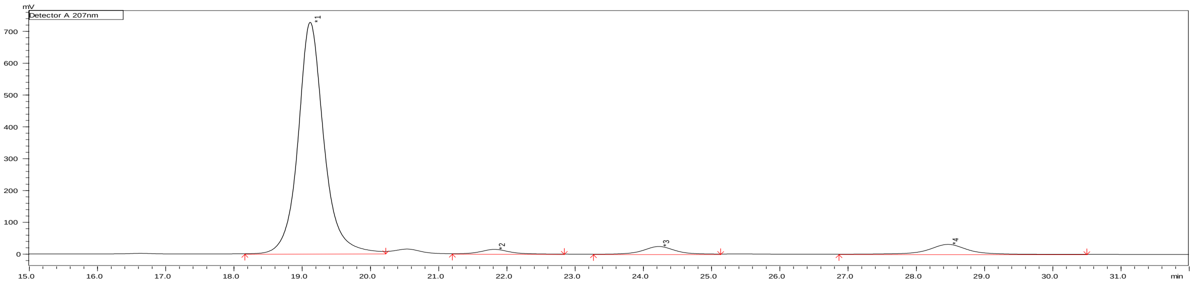

Peak#	Ret. Time	Area%
1	19.176	27.524
2	21.821	27.629
3	24.242	22.430
4	28.485	22.417

Peak#	Ret. Time	Area%
1	19.122	88.980
2	21.823	1.813
3	24.232	3.563
4	28.469	5.645

Supplementary Figure 16. HPLC chromatogram for compound **3n**

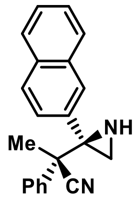

3o

3o: Procedure A, 57.9 mg, white solid, 97% yield, >20/1 dr.
^{1}H NMR (400 MHz, CDCl$_3$) δ 7.75-7.71 (m, 1H), 7.68-7.64 (m, 1H), 7.57 (d, *J* = 8.3 Hz, 1H), 7.49-7.37 (m, 3H), 7.36-7.24 (m, 5H), 6.98 (d, *J* = 8.0 Hz, 1H), 2.44 (brs, 1H), 2.09 (brs, 1H), 1.82 (s, 3H), 1.13 (brs, 1H) ppm.
^{13}C NMR (100 MHz, CDCl$_3$) δ 137.47, 135.73, 132.64, 132.26, 129.54, 128.27, 128.21, 127.86, 127.40, 127.09, 127.02, 126.41, 126.20, 122.13, 47.23, 29.88, 21.85 ppm.
HRMS (ESI) m/z [M+H]$^+$: calcd. 299.1543, found 299.1542.
IR (film): 3291, 3057, 2962, 2233, 1596, 821, 751, 695 cm^{-1}.
Optical rotation: $[\alpha]_D^{25}$ = +12.34 (*c* = 1.063, CH_2Cl_2, 97% ee).
HPLC: DAICEL CHIRALPAK IA, hexane/*i*-PrOH = 24/1, flow rate: 0.5 mL/min, λ = 207 nm, t_R(major) = 25.2 min, t_R(minor) = 28.6 min, ee = 97%.

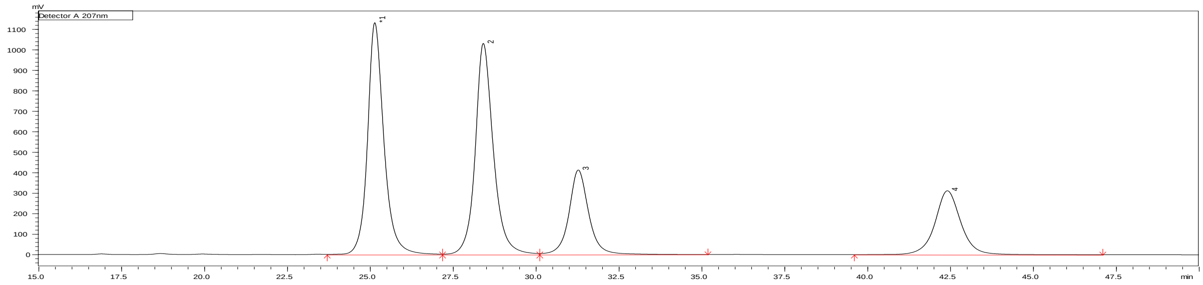

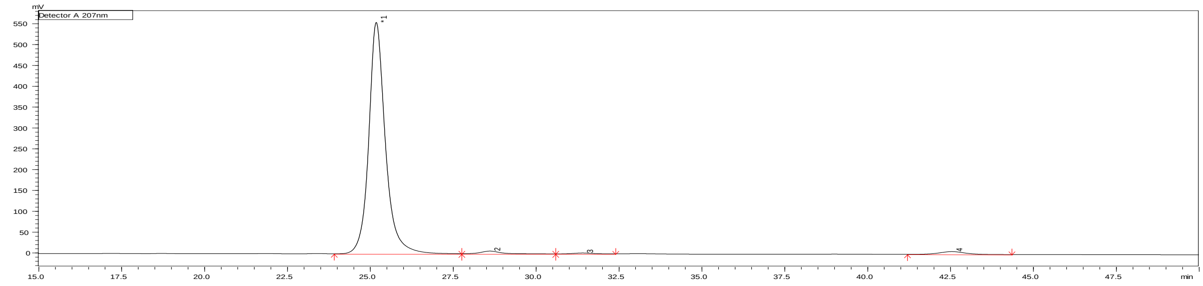

Peak#	Ret. Time	Area%
1	25.145	34.732
2	28.418	34.924
3	31.283	15.255
4	42.415	15.090

Peak#	Ret. Time	Area%
1	25.189	96.136
2	28.617	1.488
3	31.414	0.534
4	42.550	1.842

Supplementary Figure 17. HPLC chromatogram for compound **3o**

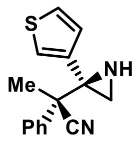

3p

3p: Procedure A, 50.0 mg, colorless solid, 98% yield, 12/1 dr.

^{1}H NMR (400 MHz, $CDCl_3$) δ 7.43-7.29 (m, 5H), 7.06 (d, *J* = 1.8 Hz, 1H), 6.90 (s, 1H), 6.59 (d, *J* = 4.5 Hz, 1H), 2.34 (brs, 1H), 2.07 (brs, 1H), 1.77 (s, 3H), 1.08 (brs, 1H) ppm.

^{13}C NMR (100 MHz, $CDCl_3$) δ 139.34, 137.85, 128.35, 128.31, 128.17, 126.79, 125.34, 124.78, 122.16, 46.84, 43.17, 29.93, 21.79 ppm.

HRMS (ESI) m/z [M+H]$^+$: calcd. 255.0950, found 255.0953.

IR (film): 3299, 3103, 3063, 2926, 2237, 1600, 788, 699 cm^{-1}.

Optical rotation: $[\alpha]_D^{25}$ = -11.61 (*c* = 0.990, CH_2Cl_2, 96% ee).

HPLC: DAICEL CHIRALPAK IA, hexane/*i*-PrOH = 24/1, flow rate: 0.5 mL/min, λ = 207 nm, t_R(major) = 22.7 min, t_R(minor) = 24.3 min, ee = 96%.

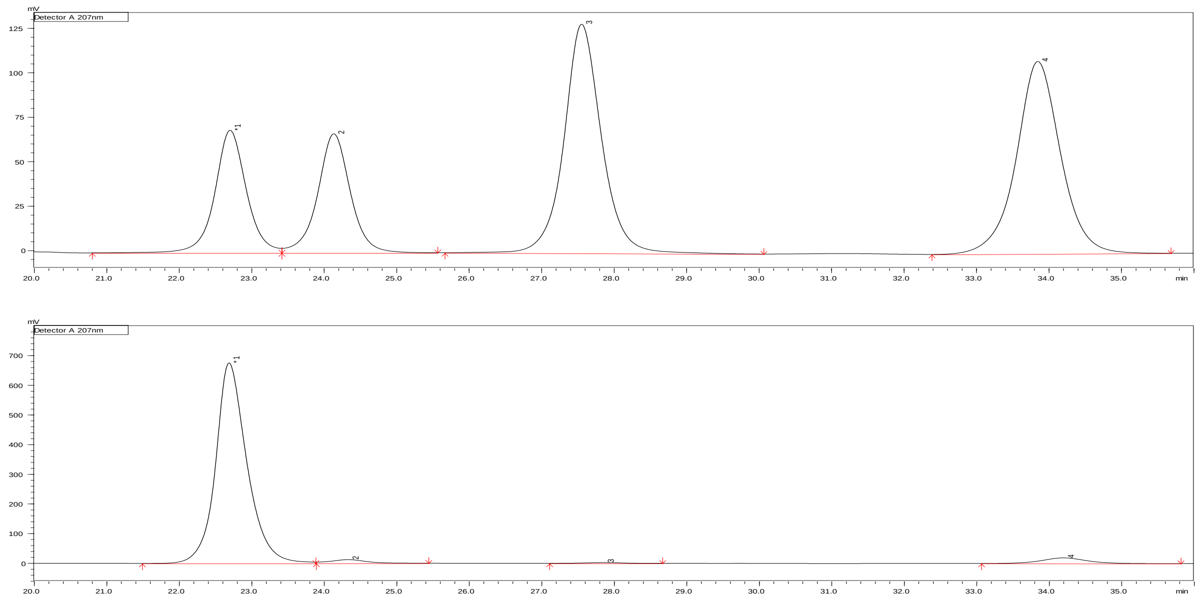

Peak#	Ret. Time	Area%
1	22.707	15.531
2	24.137	15.557
3	27.557	34.623
4	33.850	34.290

Peak#	Ret. Time	Area%
1	22.692	93.908
2	24.334	1.909
3	27.854	0.388
4	34.201	3.796

Supplementary Figure 18. HPLC chromatogram for compound **3p**

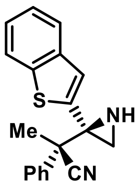

3q

3q: Procedure A, 60.4 mg, pale yellow solid, 99% yield, >20/1 dr.
^{1}H NMR (400 MHz, $CDCl_3$) δ 7.66-7.57 (m, 2H), 7.53-7.47 (m, 2H), 7.39-7.21 (m, 5H), 7.00 (s, 1H), 2.43 (brs, 1H), 2.29 (brs, 1H), 1.85 (s, 3H), 1.17 (brs, 1H) ppm.
^{13}C NMR (100 MHz, $CDCl_3$) δ 142.05, 139.40, 138.31, 137.61, 128.58, 127.10, 126.96, 125.20, 124.71, 124.33, 123.62, 121.90, 46.74, 43.20, 31.75, 22.16 ppm.
HRMS (ESI) m/z [M+H]$^+$: calcd. 305.1107, found 305.1110.
IR (film): 3295, 3059, 2962, 2926, 2237, 1600, 749, 726, 698 cm^{-1}.
Optical rotation: $[\alpha]_D^{25}$ = +5.97 (*c* = 1.036, CH_2Cl_2, 93% ee).
HPLC: DAICEL CHIRALPAK ODH, hexane/*i*-PrOH = 4/1, flow rate: 0.5 mL/min, λ = 207 nm, t_R(major) = 25.6 min, t_R(minor) = 32.6 min, ee = 93%.

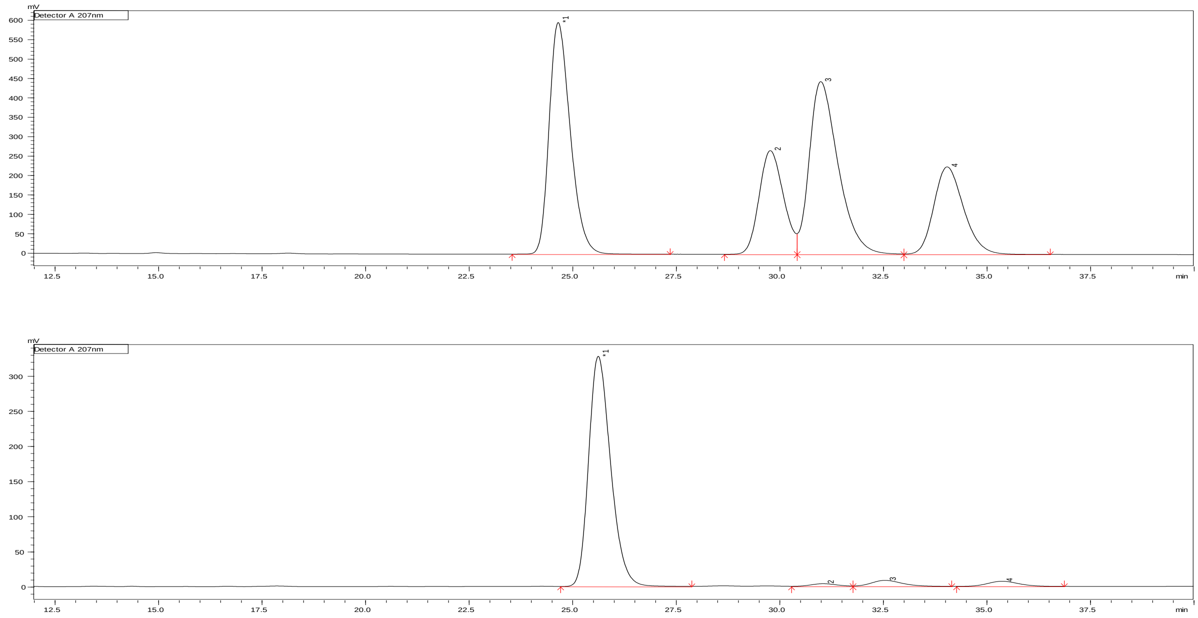

Peak#	Ret. Time	Area%
1	24.655	32.665
2	29.777	16.811
3	30.994	33.201
4	34.045	17.323

Peak#	Ret. Time	Area%
1	25.625	92.497
2	31.058	1.276
3	32.554	3.350
4	35.376	2.878

Supplementary Figure 19. HPLC chromatogram for compound **3q**

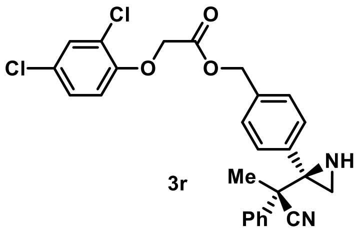

3r

3r: Procedure B, 93.5 mg, white solid, 97% yield, 12/1 dr.

^{1}H NMR (400 MHz, CDCl$_3$) δ 7.37-7.28 (m, 6H), 7.14-7.08 (m, 3H), 6.98 (d, *J* = 7.9 Hz, 2H), 6.73 (d, *J* = 8.8 Hz, 1H), 5.13 (s, 2H), 4.69 (s, 2H), 2.34 (brs, 1H), 1.98 (brs, 1H), 1.77 (s, 3H), 1.06 (brs, 1H) ppm.

^{13}C NMR (100 MHz, CDCl$_3$) δ 167.65, 152.10, 138.58, 137.20, 134.81, 130.29, 130.14, 128.27, 128.23, 127.40, 127.38, 126.89, 124.02, 121.99, 114.56, 66.35, 66.14, 46.84, 29.57, 21.79 ppm.

HRMS (ESI) m/z [M+H]$^+$: calcd. 481.1080, found 481.1080.

IR (film): 3305, 3062, 2962, 2237, 1760, 1600, 1192, 875, 804, 736, 699 cm^{-1}.

Optical rotation: $[\alpha]_D^{25}$ = -1.80 (*c* = 1.030, CH_2Cl_2, 97% ee).

HPLC: DAICEL CHIRALPAK IA connected with DAICEL CHIRALPAK ODH in series,, hexane/*i*-PrOH = 2/1, flow rate: 0.6 mL/min, λ = 207 nm, t_R(major) = 57.5 min, t_R(minor) = 51.3 min, ee = 97%.

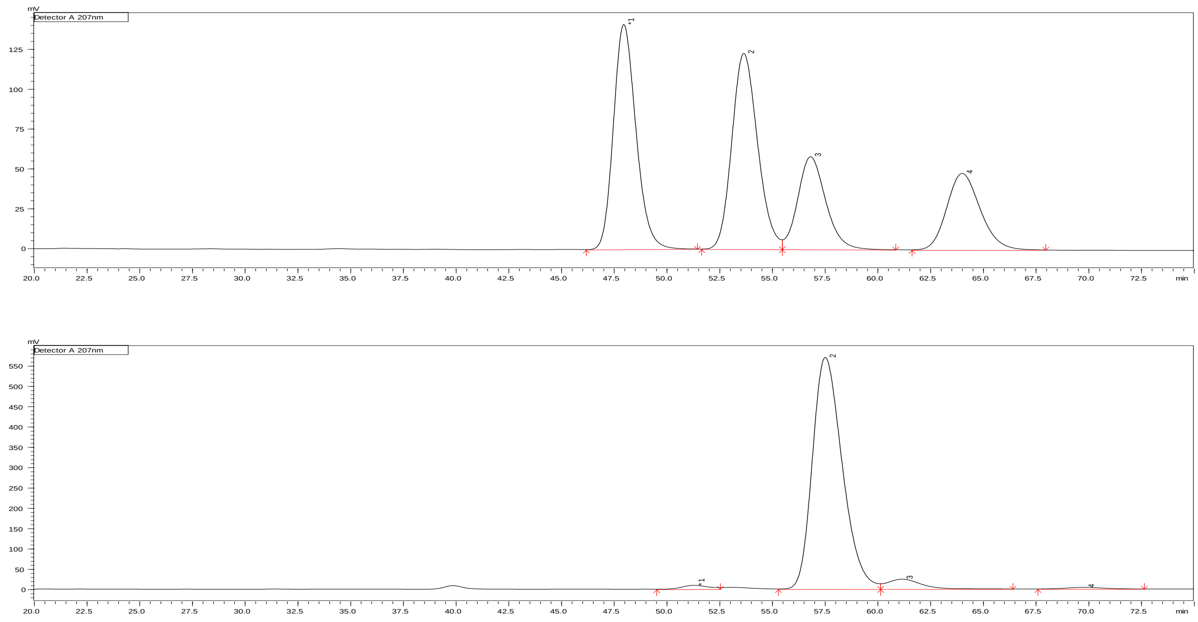

Peak#	Ret. Time	Area%
1	47.966	33.249
2	53.658	33.052
3	56.822	16.984
4	64.011	16.716

Peak#	Ret. Time	Area%
1	51.306	1.385
2	57.522	93.325
3	61.167	4.486
4	69.776	0.804

Supplementary Figure 20. HPLC chromatogram for compound **3r**

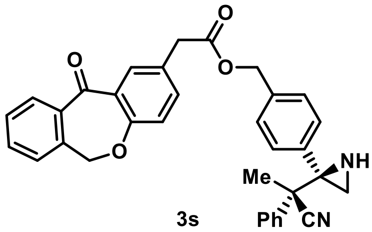

3s

3s: Procedure B, 104.5 mg, white solid, 99% yield, 11/1 dr.

^{1}H NMR (400 MHz, CDCl$_3$) δ 8.09 (d, J = 2.2 Hz, 1H), 7.86 (d, J = 7.6 Hz, 1H), 7.54 (td, J = 7.4, 1.0 Hz, 1H), 7.45 (td, J = 11.0, 4.0 Hz, 1H), 7.41-7.27 (m, 7H), 7.10 (d, J = 7.7 Hz, 2H), 6.98 (m, 3H), 5.15 (s, 2H), 5.05 (s, 2H), 3.65 (s, 2H), 2.33 (brs, 1H), 1.97 (brs, 1H), 1.76 (s, 3H), 1.13 (brs, 1H) ppm.

^{13}C NMR (100 MHz, CDCl$_3$) δ 190.53, 170.90, 160.29, 140.16, 138.05, 137.24, 136.14, 135.59, 135.33, 132.65, 132.26, 130.20, 129.24, 129.08, 128.21, 128.15, 127.68, 127.35, 127.15, 126.87, 124.91, 122.02, 120.90, 73.40, 65.89, 46.85, 39.92, 29.68, 21.73 ppm.

HRMS (ESI) m/z [M+H]$^+$: calcd. 529.2122, found 529.2122.

IR (film): 3301, 3059, 2925, 2237, 1737, 1648, 1611, 1121, 881, 762, 699 cm^{-1}.

Optical rotation: $[\alpha]_D^{25}$ = +0.09 (c = 1.064, CH_2Cl_2, 98% ee).

HPLC: DAICEL CHIRALPAK IBN-3, hexane/*i*-PrOH = 1/1, flow rate: 0.6 mL/min, λ = 207 nm, t_R(major) = 43.4 min, t_R(minor) = 69.6 min, ee = 98%.

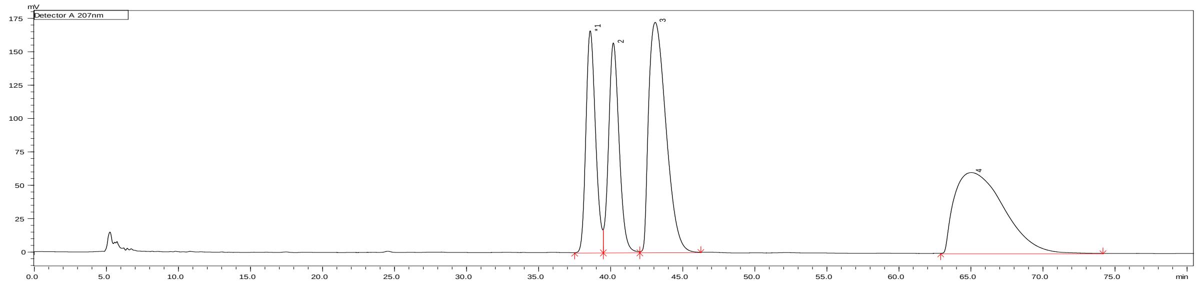

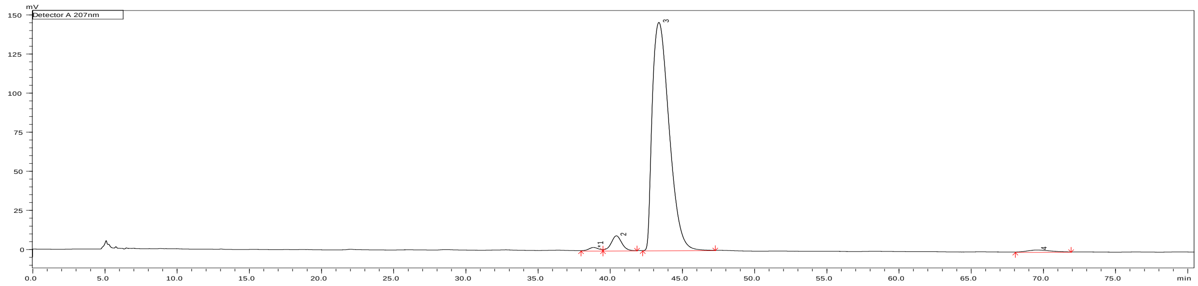

Peak#	Ret. Time	Area%
1	38.606	17.319
2	40.209	17.849
3	43.116	32.333
4	65.053	32.499

Peak#	Ret. Time	Area%
1	38.857	0.738
2	40.449	3.793
3	43.394	94.418
4	69.578	1.051

Supplementary Figure 21. HPLC chromatogram for compound **3s**

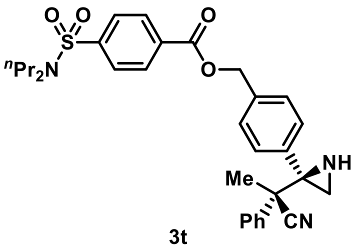

3t

3t: Procedure B, 107.0 mg, white solid, 98% yield, 11/1 dr.

^{1}H NMR (400 MHz, CDCl$_3$) δ 8.16 (d, J = 8.4 Hz, 2H), 7.87 (d, J = 8.4 Hz, 2H), 7.39-7.30 (m, 5H), 7.24 (d, J = 7.9 Hz, 2H), 7.04 (d, J = 7.7 Hz, 2H), 5.31 (s, 2H), 3.12-3.07 (m, 4H), 2.34 (brs, 1H), 2.00 (brs, 1H), 1.79 (s, 3H), 1.60-1.48 (m, 4H), 1.02 (brs, 1H), 0.86 (t, J = 7.4 Hz, 6H) ppm.

^{13}C NMR (100 MHz, CDCl$_3$) δ 164.81, 144.27, 137.23, 135.40, 133.07, 130.38, 130.18, 128.28, 128.25, 127.35, 126.95, 126.88, 122.06, 66.52, 49.78, 46.88, 29.52, 21.90, 21.79, 11.03 ppm.

HRMS (ESI) m/z [M+H]$^+$: calcd. 546.2421, found 546.2426.

IR (film): 3304, 3061, 2966, 2237, 1724, 1600, 1343, 1158, 826, 764, 699 cm^{-1}.

Optical rotation: $[\alpha]_D^{25}$ = -2.04 (c = 1.006, CH_2Cl_2, 98% ee).

HPLC: DAICEL CHIRALPAK IG-3, hexane/i-PrOH = 1/1, flow rate: 0.5 mL/min, λ = 254 nm, t_R(major) = 80.8 min, t_R(minor) = 114.8 min, ee = 98%.

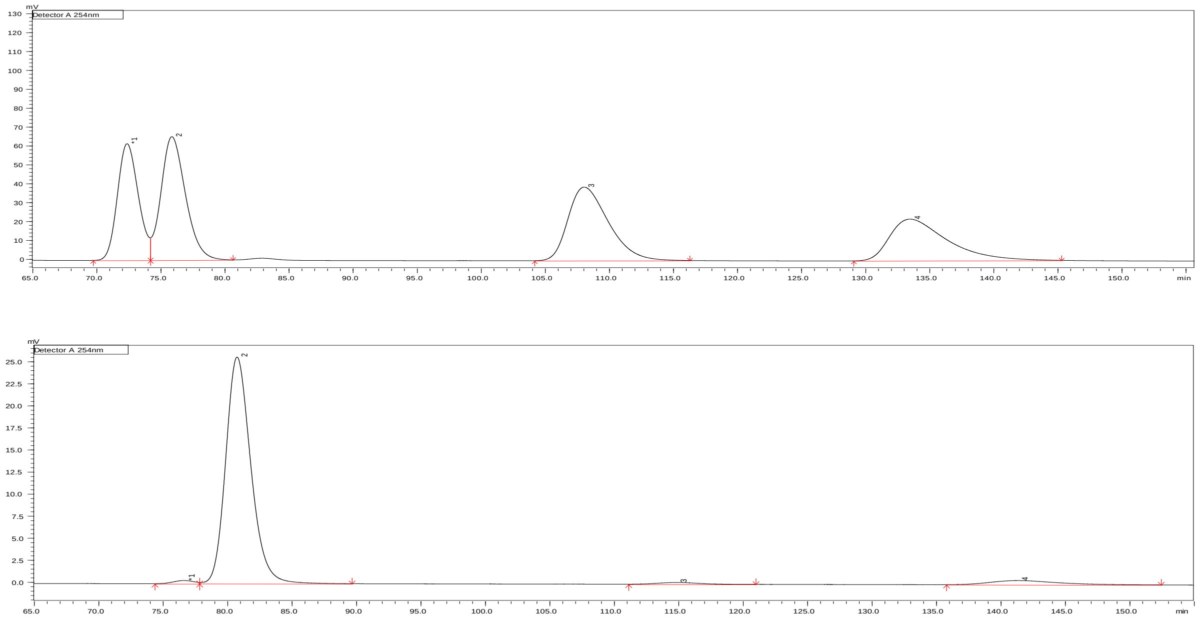

Peak#	Ret. Time	Area%
1	72.388	22.606
2	75.883	28.001
3	108.052	27.339
4	133.516	22.055

Peak#	Ret. Time	Area%
1	76.668	1.046
2	80.759	93.314
3	114.835	1.122
4	141.317	4.517

Supplementary Figure 22. HPLC chromatogram for compound **3t**

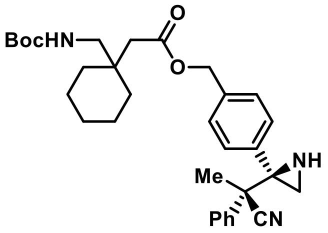

3u

3u: Procedure B, 105.3 mg, white solid, 99% yield, 12/1 dr.

^{1}H NMR (400 MHz, $CDCl_3$) δ 7.32 (s, 5H), 7.14 (d, *J* = 7.8 Hz, 2H), 6.97 (d, *J* = 7.8 Hz, 2H), 5.05-4.96 (m, 3H), 3.09 (d, *J* = 6.7 Hz, 2H), 2.36 (brs, 1H), 2.31 (s, 2H), 1.99 (brs, 1H), 1.78 (s, 3H), 1.54-1.29 (m, 20H) ppm.

^{13}C NMR (100 MHz, $CDCl_3$) δ 164.81, 144.27, 138.25, 137.23, 135.40, 133.07, 130.38, 130.18, 128.28, 128.25, 127.35, 126.95, 126.88, 122.06, 66.52, 49.78, 46.88, 29.52, 21.90, 21.79, 11.03 ppm.

HRMS (ESI) m/z [M+H]$^+$: calcd. 532.3170, found 532.3185.

IR (film): 3442, 3301, 3060, 2929, 2863, 2237, 1716, 820, 764, 699 cm^{-1}.

Optical rotation: $[\alpha]_D^{25}$ = +1.65 (*c* = 1.023, CH_2Cl_2, 98% ee).

HPLC: DAICEL CHIRALPAK IA, hexane/*i*-PrOH = 9/1, flow rate: 0.5 mL/min, λ = 207 nm, t_R(major) = 29.9 min, t_R(minor) = 32.6 min, ee = 98%.

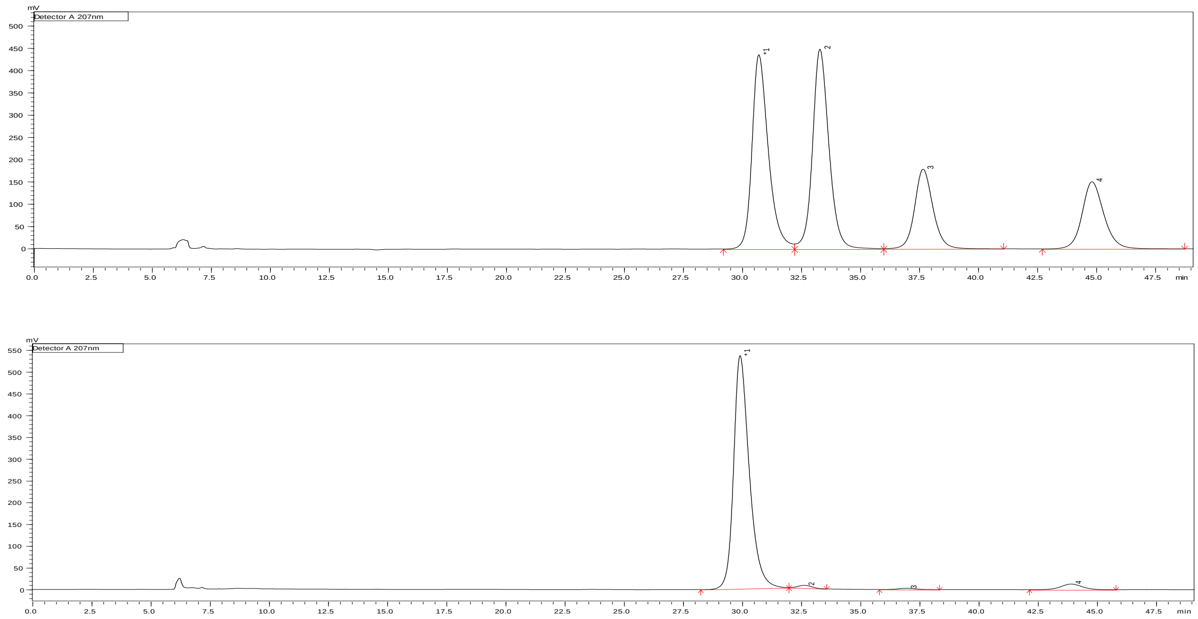

Peak#	Ret. Time	Area%
1	30.708	33.887
2	33.295	34.931
3	37.665	15.627
4	44.821	15.555

Peak#	Ret. Time	Area%
1	29.914	95.264
2	32.628	0.989
3	36.955	0.577
4	43.914	3.169

Supplementary Figure 23. HPLC chromatogram for compound **3u**

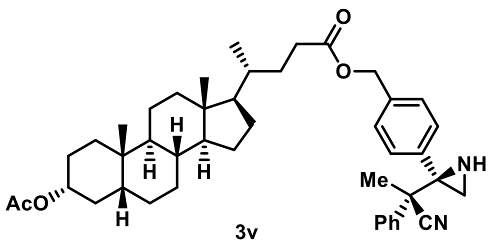

3v

3v: Procedure B, 133.1 mg, white solid, 98% yield, >20/1 dr.
^{1}H NMR (400 MHz, CDCl$_3$) δ 7.32 (s, 5H), 7.13 (d, *J* = 7.9 Hz, 2H), 6.98 (d, *J* = 7.8 Hz, 2H), 5.02 (s, 2H), 4.75-4.68 (m, 1H), 2.46-2.16 (m, 3H), 2.10-0.75 (m, 40H), 0.62 (s, 3H) ppm.
^{13}C NMR (100 MHz, CDCl$_3$) δ 173.81, 170.47, 137.38, 136.09, 130.26, 128.26, 128.20, 127.19, 126.96, 122.05, 74.24, 65.30, 56.35, 55.84, 46.90, 42.60, 41.75, 40.27, 40.01, 35.65, 35.20, 34.91, 34.45, 32.12, 31.08, 30.81, 28.06, 26.89, 26.51, 26.20, 24.06, 23.23, 21.93, 21.37, 20.70, 18.15, 11.93 ppm.
HRMS (ESI) m/z [M+H]$^+$: calcd. 679.4469, found 679.4479.
IR (film): 3301, 3059, 2939, 2867, 2237, 1735, 822, 737, 699 cm^{-1}.
Optical rotation: $[\alpha]_D^{25}$ = +29.18 (*c* = 1.083, CH_2Cl_2, 98% de).
HPLC: DAICEL CHIRALPAK IG-3 connected with DAICEL CHIRALPAK IBN-3 in series, hexane/*i*-PrOH = 3/1, flow rate: 0.5 mL/min, λ = 207 nm, t_R(major) = 63.5 min, t_R(minor) = 68.2 min, de = 98%. Diastereoselectivity determined by chiral-stationary-phase HPLC analysis.

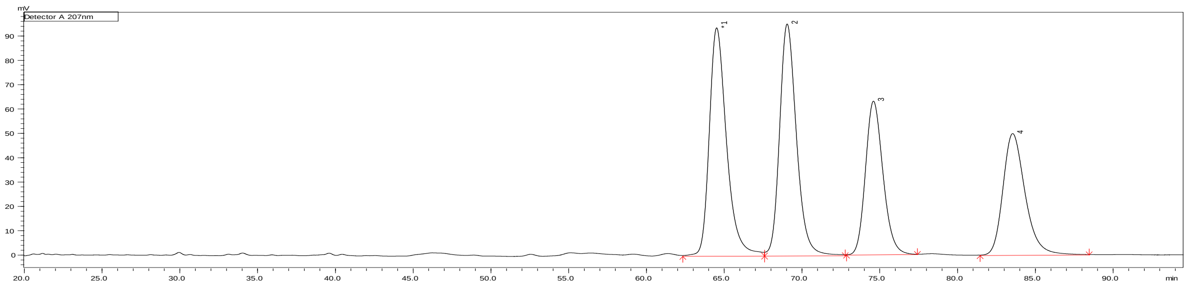

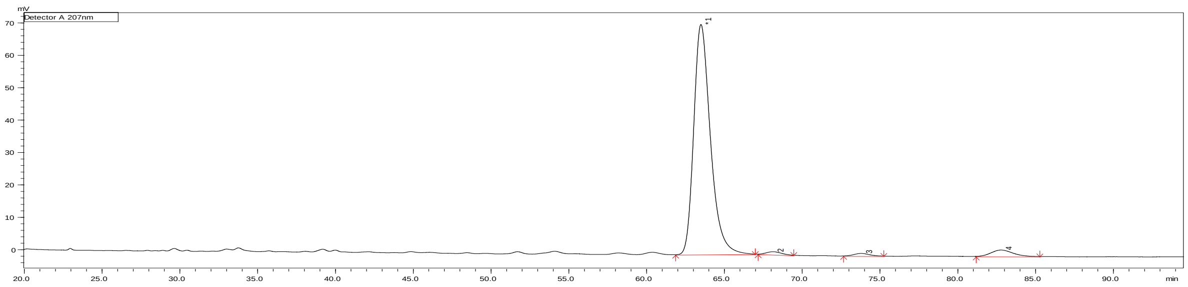

Peak#	Ret. Time	Area%
1	64.537	29.843
2	69.068	29.537
3	74.618	20.308
4	83.569	20.312

Peak#	Ret. Time	Area%
1	63.516	94.546
2	68.150	1.046
3	73.840	0.965
4	82.832	3.443

Supplementary Figure 24. HPLC chromatogram for compound **3v**

General procedure for Copper(I)-catalyzed asymmetric decarboxylative Mannich reaction of aliphatic 2H-azirines and cyanoacetic acids

Procedure C:

Preparation of catalyst solution: A dried 25 mL Schlenk tube equipped with a magnetic stirring bar was charged with CuOAc (1.5 mg, 0.012 mmol) and (*R*)-DTBM-SEGPHOS (14.2 mg, 0.012 mmol) in a glove box under Ar atmosphere. Anhydrous THF (2 mL) was added via a syringe. The mixture was stirred for 15 minutes to give a clear catalyst solution.

A dried 25 mL Schlenk tube equipped with a magnetic stirring bar was charged with aliphatic 2*H*-azirines **4** (0.2 mmol, 1.0 equiv). The catalyst solution (1.0 mL) containing copper(I) complex (0.006 mmol, 0.03 equiv) was added via a syringe. The reaction mixture was then cooled to -20 °C and cyanoacetic acid **1a** (0.25 M in THF, 2.0 mL, 0.5 mmol, 2.5 equiv) was added over 12 h with a syringe pump. The reaction mixture was purified by silica gel column chromatography (petroleum ether/ethyl acetate) to give the product.

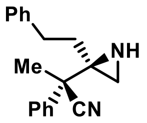

5a

5a: Procedure C, 52.2 mg, colorless solid, 94% yield, 6/1 dr.

^{1}H NMR (400 MHz, $CDCl_3$) δ 7.53 (d, J = 7.2 Hz, 2H), 7.40-7.31 (m, 3H), 7.22-7.11 (m, 3H), 6.98 (d, J = 7.1 Hz, 2H), 2.39-2.30 (t, 2H), 2.04-1.83 (m, 4H), 1.67 (s, 3H), 0.61 (brs, 1H) ppm.

^{13}C NMR (100 MHz, $CDCl_3$) δ 140.43, 138.11, 128.72, 128.33, 128.13, 127.89, 126.47, 126.00, 122.35, 47.16, 41.79, 33.48, 30.75, 27.52, 22.96 ppm.

HRMS (ESI) m/z [M+H]$^+$: calcd. 277.1699, found 277.1700.

IR (film): 3303, 3061, 2942, 2862, 2235, 1602, 757, 699 cm^{-1}.

Optical rotation: $[\alpha]_D^{25}$ = +0.64 (c = 1.090, CH_2Cl_2, 91% ee).

HPLC: DAICEL CHIRALPAK IA connected with DAICEL CHIRALPAK ODH in series, hexane/*i*-PrOH = 4/1, flow rate: 0.5 mL/min, λ = 207 nm, t_R(major) = 40.0 min, t_R(minor) = 43.2 min, ee = 91%.

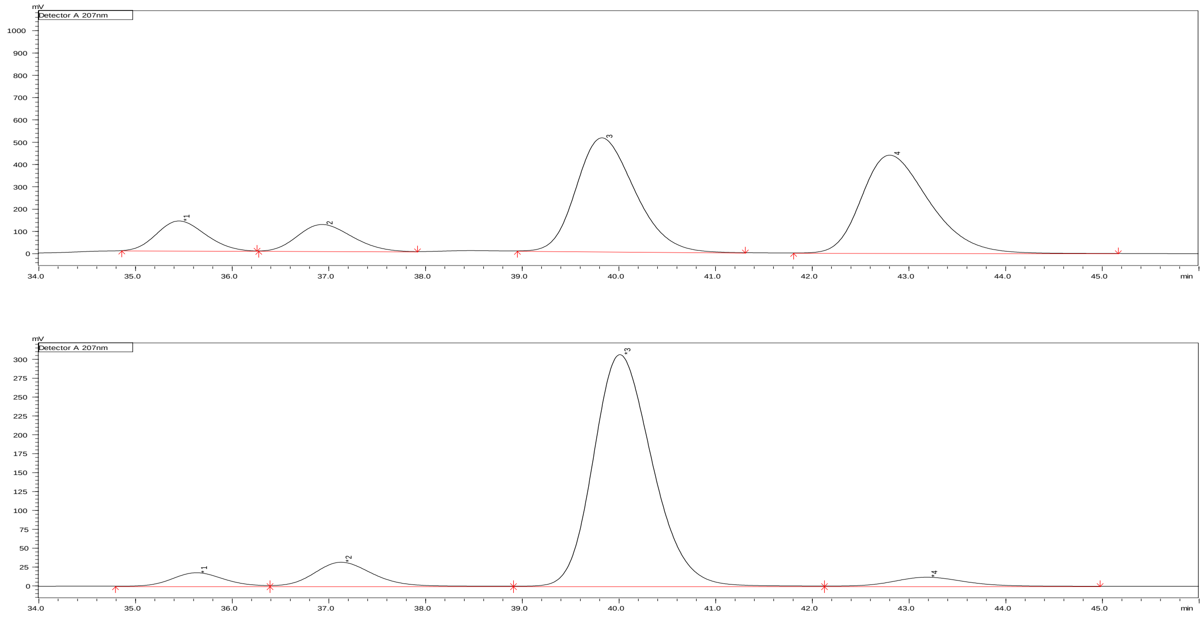

Peak#	Ret. Time	Area%
1	35.454	8.906
2	36.935	8.915
3	39.829	41.128
4	42.806	41.052

Peak#	Ret. Time	Area%
1	35.635	4.236
2	37.132	8.521
3	40.013	83.337
4	43.187	3.907

Supplementary Figure 25. HPLC chromatogram for compound **5a**

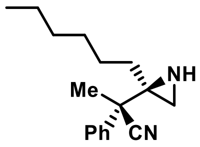

5b

5b: Procedure C, 49.0 mg, colorless oil, 96% yield, 10/1 dr.
^{1}H NMR (400 MHz, $CDCl_3$) δ 7.51 (d, *J* = 7.3 Hz, 2H), 7.42-7.30 (m, 3H), 1.92 (brs, 1H), 1.85 (brs, 1H), 1.67 (s, 3H), 1.60-1.54 (m, 2H), 1.26-1.10 (m, 6H), 1.02-0.99 (m, 2H), 0.82 (t, *J* = 7.0 Hz, 3H), 0.60 (brs, 1H) ppm.
^{13}C NMR (100 MHz, $CDCl_3$) δ 138.44, 128.62, 127.96, 126.49, 122.50, 47.30, 41.70, 31.42, 31.24, 29.07, 27.17, 24.11, 22.99, 22.33, 13.86 ppm.
HRMS (ESI) m/z [M+H]$^+$: calcd. 257.2012, found 257.2013.
IR (film): 3304, 3062, 2930, 2858, 2236, 1601, 759, 700 cm^{-1}.
Optical rotation: $[\alpha]_D^{25}$ = -4.29 (*c* = 1.065, CH_2Cl_2, 96% ee).
HPLC: DAICEL CHIRALPAK IA, hexane/*i*-PrOH = 24/1, flow rate: 0.5 mL/min, λ = 207 nm, t_R(major) = 17.4 min, t_R(minor) = 16.5 min, ee = 96%.

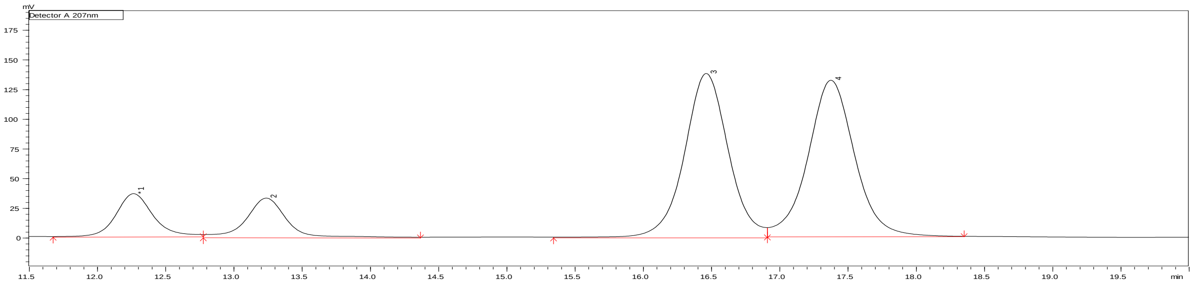

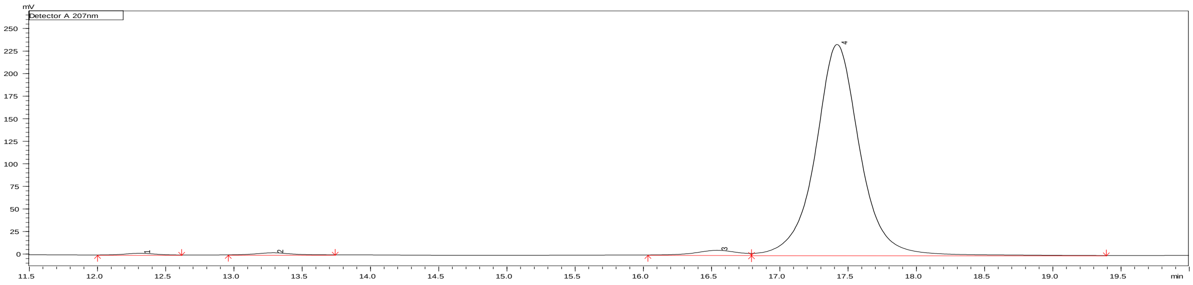

Peak#	Ret. Time	Area%
1	12.267	9.031
2	13.239	8.873
3	16.463	40.855
4	17.377	41.241

Peak#	Ret. Time	Area%
1	12.312	0.513
2	13.288	0.705
3	16.543	1.964
4	17.422	96.819

Supplementary Figure 26. HPLC chromatogram for compound **5b**

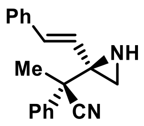

5c

5c: Procedure C, 52.2 mg, pale yellow oil, 95% yield, 3.5/1 dr.
^{1}H NMR (400 MHz, $CDCl_3$) δ 7.56 (d, *J* = 7.4 Hz, 2H), 7.42-7.33 (m, 3H), 7.30-7.19 (m, 5H), 6.46 (brs, 1H), 6.26 (d, *J* = 15.6 Hz, 1H), 2.15 (brs, 2H), 1.77 (s, 3H), 0.80 (brs, 1H) ppm.
^{13}C NMR (100 MHz, $CDCl_3$) δ 135.67, 132.76, 128.65, 128.56, 128.29, 128.16, 126.84, 126.43, 125.88, 122.07, 46.34, 44.14, 30.49, 22.86 ppm.
HRMS (ESI) m/z [M+H]$^+$: calcd. 275.1543, found 275.1545.
IR (film): 3298, 3060, 3027, 2926, 2236, 1600, 967, 750, 697 cm^{-1}.
Optical rotation: $[\alpha]_D^{25}$ = +172.50 (*c* = 0.800, CH_2Cl_2, 93% ee).
HPLC: DAICEL CHIRALPAK ADH, hexane/*i*-PrOH = 24/1, flow rate: 0.5 mL/min, λ = 207 nm, t_R(major) = 47.1 min, t_R(minor) = 52.2 min, ee = 93%.

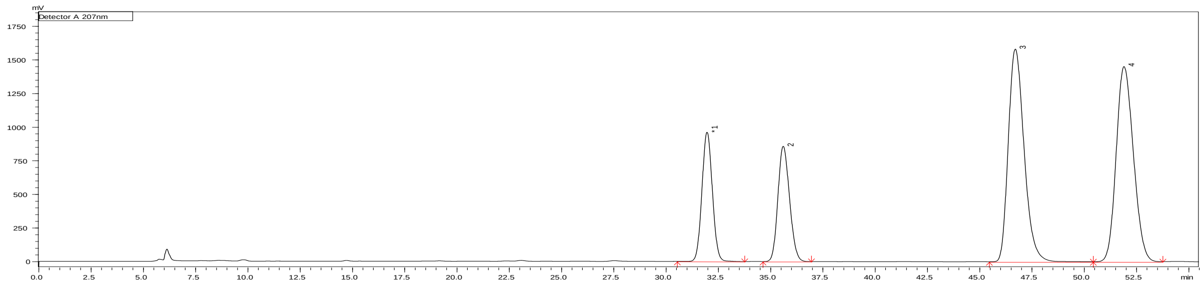

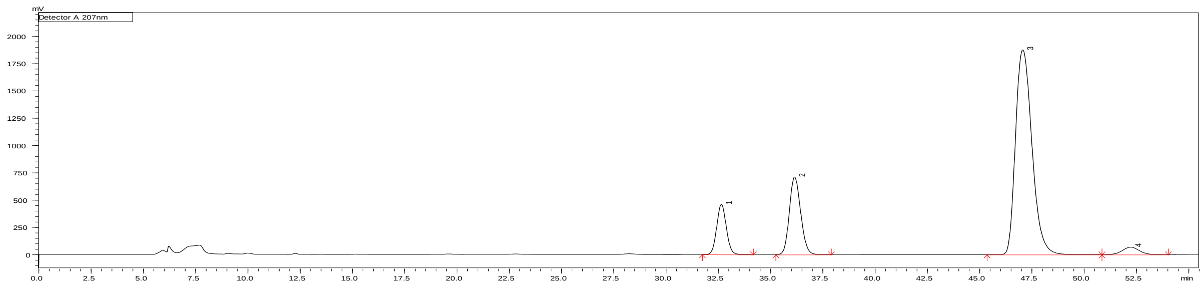

Peak#	Ret. Time	Area%
1	31.976	14.332
2	35.620	14.238
3	46.721	35.704
4	51.918	35.726

Peak#	Ret. Time	Area%
1	32.666	9.924
2	36.166	18.080
3	47.078	69.495
4	52.242	2.501

Supplementary Figure 27. HPLC chromatogram for compound **5c**

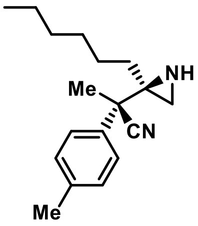

5d

5d: Procedure C, 49.2 mg, pale yellow oil, 91% yield, 10/1 dr.

^{1}H NMR (400 MHz, CDCl$_3$) δ 7.38 (d, *J* = 8.1 Hz, 2H), 7.19 (d, *J* = 7.9 Hz, 2H), 2.36 (s, 3H), 1.86 (brs, 2H), 1.65 (s+brs, 5H), 1.26-1.12 (m, 6H), 1.02 (brs, 2H), 0.83 (t, *J* = 6.9 Hz, 3H), 0.67 (brs, 1H) ppm.

^{13}C NMR (100 MHz, CDCl$_3$) δ 137.79, 135.35, 129.31, 129.25, 126.40, 47.30, 41.62, 31.48, 29.14, 24.07, 23.01, 22.37, 20.91, 13.90 ppm.

HRMS (ESI) m/z [M+H]$^+$: calcd. 271.2169, found 271.2170.

IR (film): 3304, 3059, 2929, 2858, 2235, 841, 817 cm^{-1}.

Optical rotation: $[\alpha]_D^{25}$ = -5.96 (*c* = 1.103, CH_2Cl_2, 95% ee).

HPLC: DAICEL CHIRALPAK ADH, hexane/*i*-PrOH = 47/3, flow rate: 0.5 mL/min, λ = 207 nm, t_R(major) = 16.3 min, t_R(minor) = 14.9 min, ee = 95%.

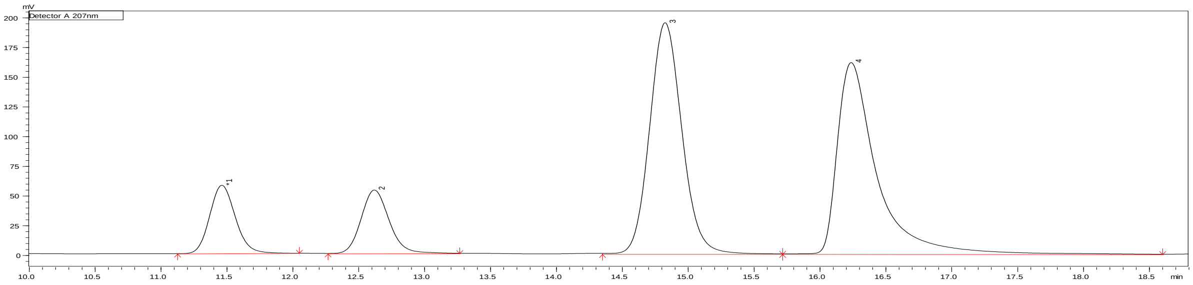

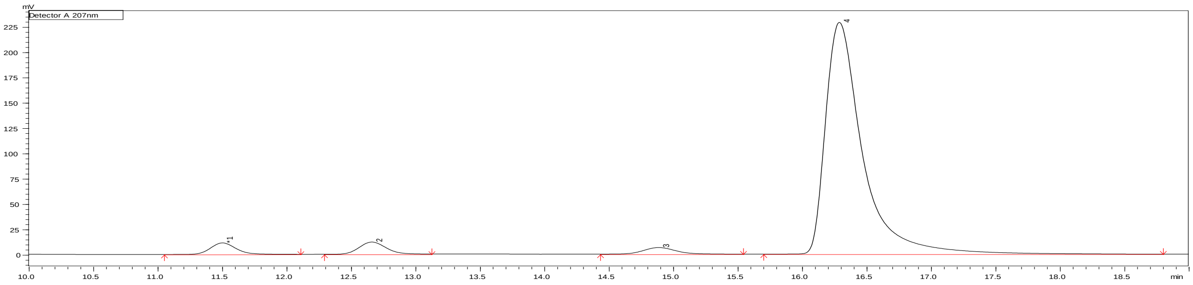

Peak#	Ret. Time	Area%
1	11.464	9.402
2	12.621	9.552
3	14.827	40.359
4	16.239	40.687

Peak#	Ret. Time	Area%
1	11.503	3.156
2	12.662	3.604
3	14.887	2.294
4	16.289	90.946

Supplementary Figure 28. HPLC chromatogram for compound **5d**

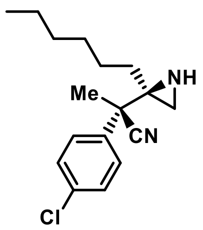

5e

5e: Procedure C, 36.0 mg, pale yellow oil, 62% yield, 11/1 dr.

^{1}H NMR (400 MHz, $CDCl_3$) δ 7.47 (d, *J* = 8.6 Hz, 2H), 7.36 (d, *J* = 8.6 Hz, 2H), 1.89 (brs, 1H), 1.86 (brs, 1H), 1.66 (s, 3H), 1.62-1.48 (m, 2H), 1.36-1.10 (m, 6H), 1.05-0.96 (m, 2H), 0.83 (t, *J* = 7.0 Hz, 3H), 0.54 (brs, 1H) ppm.

^{13}C NMR (100 MHz, $CDCl_3$) δ 137.08, 134.04, 128.81, 128.03, 122.16, 46.98, 41.67, 31.47, 30.99, 29.09, 27.12, 24.11, 22.90, 22.38, 13.90 ppm.

HRMS (ESI) m/z [M+H]$^+$: calcd. 291.1623, found 291.1624.

IR (film): 3305, 3066, 2930, 2858, 2236, 828, 802 cm^{-1}.

Optical rotation: $[\alpha]_D^{25}$ = -9.36 (*c* = 1.057, CH_2Cl_2, 96% ee).

HPLC: DAICEL CHIRALPAK ADH, hexane/*i*-PrOH = 47/3, flow rate: 0.5 mL/min, λ = 207 nm, t_R(major) = 17.6 min, t_R(minor) = 15.6 min, ee = 96%.

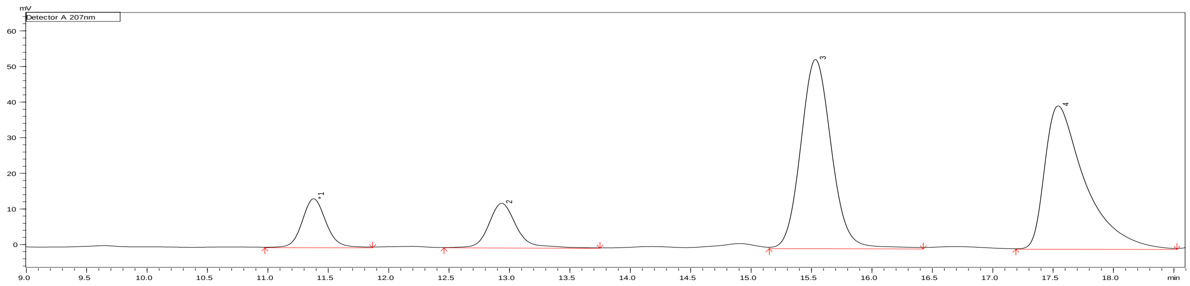

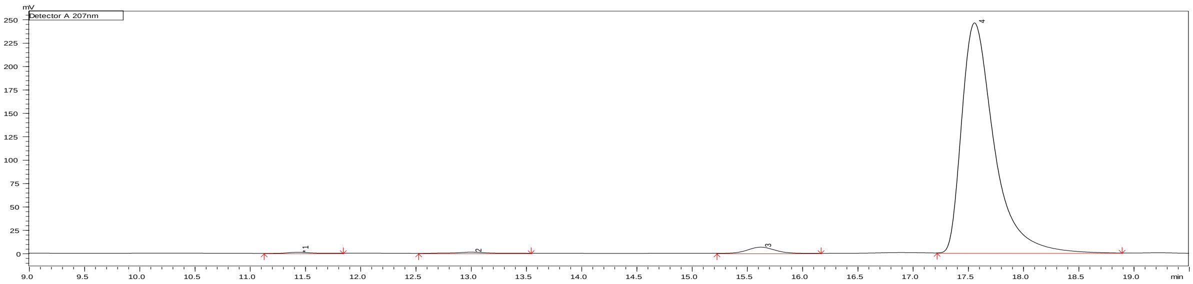

Peak#	Ret. Time	Area%
1	11.382	8.314
2	12.938	8.669
3	15.536	41.699
4	17.545	41.318

Peak#	Ret. Time	Area%
1	11.436	0.369
2	13.002	0.568
3	15.624	2.186
4	17.559	96.877

Supplementary Figure 29. HPLC chromatogram for compound **5e**

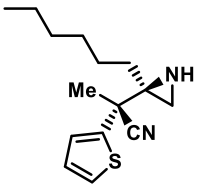

5f

5f: Procedure C, 48.5 mg, pale yellow oil, 92% yield, 10/1 dr.
^{1}H NMR (400 MHz, CDCl$_3$) δ 7.30 (dd, *J* = 5.1, 0.9 Hz, 1H), 7.19 (dd, *J* = 3.5, 0.9 Hz, 1H), 6.98 (dd, *J* = 5.1, 3.6 Hz, 1H), 1.93 (brs, 1H), 1.84 (brs, 1H), 1.78-1.69 (m, 4H), 1.64-1.56 (m, 1H), 1.26-1.18 (m, 6H), 1.05-1.00 (m, 2H), 0.86-0.82 (m, 4H) ppm.
^{13}C NMR (100 MHz, CDCl$_3$) δ 142.13, 126.38, 126.02, 125.78, 121.48, 44.95, 42.33, 31.45, 30.27, 29.12, 27.29, 24.13, 23.97, 22.37, 13.89 ppm.
HRMS (ESI) m/z [M+H]$^+$: calcd. 263.1576, found 263.1573.
IR (film): 3305, 3072, 2929, 2857, 2237, 830, 701 cm^{-1}.
Optical rotation: $[\alpha]_D^{25}$ = -29.16 (*c* = 1.163, CH_2Cl_2, 94% ee).
HPLC: DAICEL CHIRALPAK ADH, hexane/*i*-PrOH = 47/3, flow rate: 0.5 mL/min, λ = 207 nm, t_R(major) = 17.4 min, t_R(minor) = 15.3 min, ee = 94%.

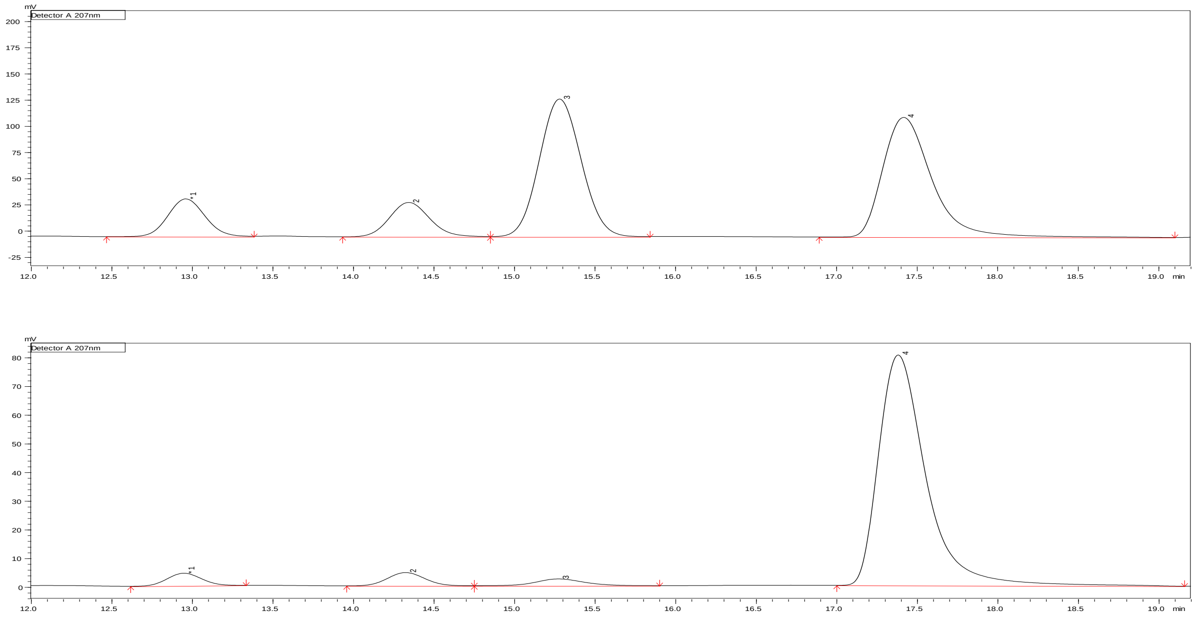

Peak#	Ret. Time	Area%
1	12.962	9.738
2	14.346	9.552
3	15.282	40.059
4	17.418	40.651

Peak#	Ret. Time	Area%
1	12.950	3.570
2	14.326	4.043
3	15.274	2.695
4	17.383	89.692

Supplementary Figure 30. HPLC chromatogram for compound **5f**

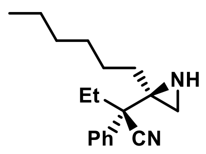

5g

5g: Procedure C, 33.5 mg, pale yellow oil, 62% yield, 2.4/1 dr.

^{1}H NMR (400 MHz, $CDCl_3$) δ 7.47 (d, J = 7.4 Hz, 2H), 7.41-7.31 (m, 3H), 2.18-2.09 (m, 1H), 1.97 (s, 1H), 1.84-1.78 (m, 2H), 1.66-1.51 (m, 2H), 1.25-1.13 (m, 6H), 0.99 (brs, 2H), 0.90 (t, J = 7.3 Hz, 3H), 0.82 (t, J = 7.0 Hz, 3H), 0.68 (brs, 1H) ppm.

^{13}C NMR (100 MHz, $CDCl_3$) δ 136.71, 128.60, 127.92, 127.10, 121.33, 53.73, 41.92, 31.98, 31.50, 29.15, 27.70, 27.59, 24.05, 22.40, 13.93, 9.39 ppm.

HRMS (ESI) m/z [M+H]$^+$: calcd. 271.2169, found 271.2171.

IR (film): 3304, 3062, 2930, 2857, 2235, 1601, 754, 700 cm^{-1}.

Optical rotation: $[\alpha]_D^{25}$ = +8.36 (c = 1.120, CH_2Cl_2, 96% ee).

HPLC: DAICEL CHIRALPAK IA, hexane/i-PrOH = 24/1, flow rate: 0.5 mL/min, λ = 207 nm, t_R(major) = 17.6 min, t_R(minor) = 16.7 min, ee = 96%.

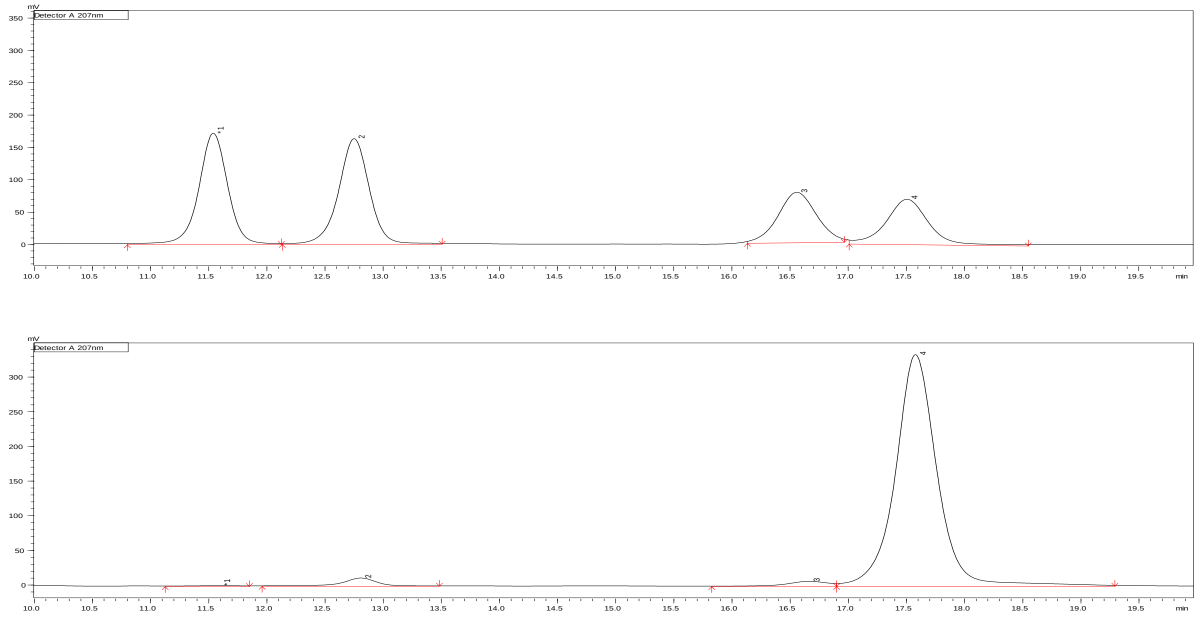

Peak#	Ret. Time	Area%
1	11.543	31.329
2	12.754	31.446
3	16.562	18.771
4	17.509	18.454

Peak#	Ret. Time	Area%
1	11.597	0.254
2	12.812	2.550
3	16.667	1.966
4	17.581	95.229

Supplementary Figure 31. HPLC chromatogram for compound **5g**

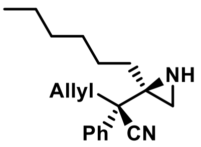

5h

5h: Procedure C, 41.2 mg, pale yellow oil, 73% yield, 2.9/1 dr.
^{1}H NMR (400 MHz, $CDCl_3$) δ 7.47 (d, *J* = 7.4 Hz, 2H), 7.40-7.31 (m, 3H), 5.65-5.55 (m, 1H), 5.11-5.05 (m, 2H), 2.84 (dd, *J* = 13.7, 7.6 Hz, 1H), 2.56 (dd, *J* = 13.6, 6.6 Hz, 1H), 1.98 (s, 1H), 1.87 (s, 1H), 1.68-1.53 (m, 2H), 1.35-1.11 (m, 6H), 1.05-0.95 (m, 2H), 0.82 (t, *J* = 7.0 Hz, 3H), 0.66 (brs, 1H) ppm.
^{13}C NMR (100 MHz, $CDCl_3$) δ 136.53, 131.72, 128.60, 128.01, 127.14, 120.98, 119.81, 52.81, 41.77, 39.05, 31.77, 31.47, 29.10, 27.68, 23.98, 22.38, 13.91 ppm.
HRMS (ESI) m/z [M+H]$^+$: calcd. 283.2169, found 283.2171.
IR (film): 3305, 3064, 2929, 2236, 1642, 1601, 993, 921, 755, 699 cm^{-1}.
Optical rotation: $[\alpha]_D^{25}$ = +20.43 (*c* = 1.385, CH_2Cl_2, 96% ee).
HPLC: DAICEL CHIRALPAK IA, hexane/*i*-PrOH = 24/1, flow rate: 0.5 mL/min, λ = 207 nm, t_R(major) = 16.5 min, t_R(minor) = 18.1 min, ee = 96%.

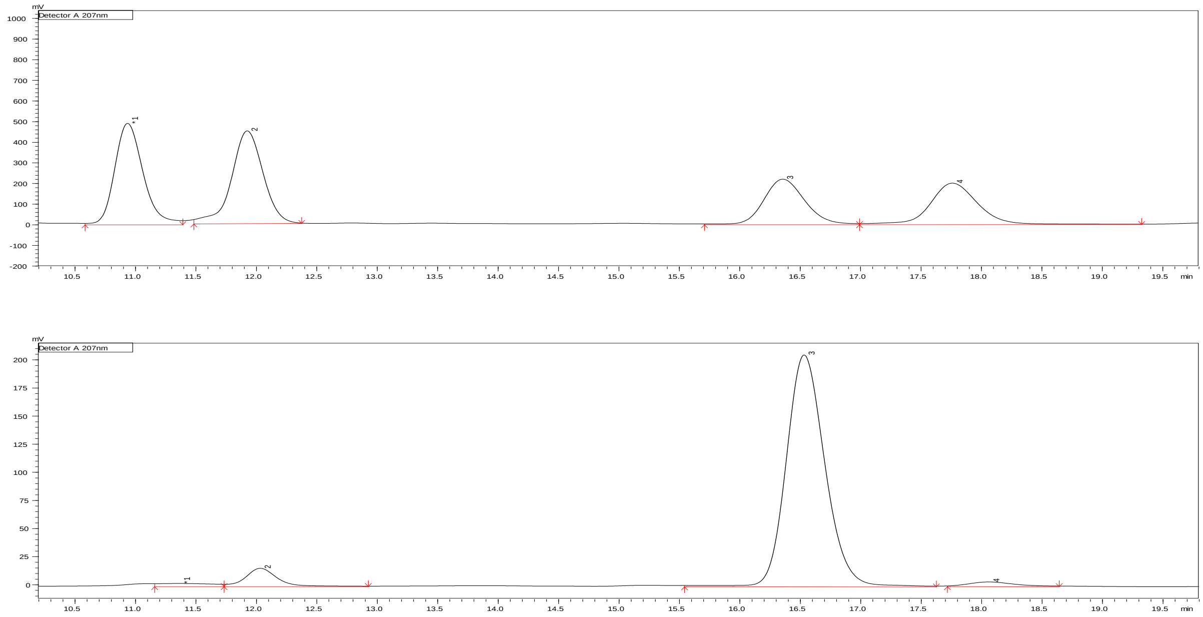

Peak#	Ret. Time	Area%
1	10.937	30.151
2	11.927	30.577
3	16.359	19.183
4	17.763	20.089

Peak#	Ret. Time	Area%
1	11.368	1.525
2	12.035	5.633
3	16.534	90.874
4	18.063	1.968

Supplementary Figure 32. HPLC chromatogram for compound **5h**

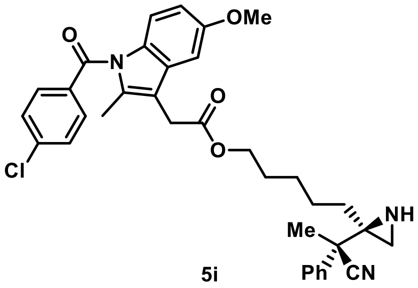

5i

5i: Procedure C, 97.3 mg, yellow oil, 81% yield, 10/1 dr.

^{1}H NMR (400 MHz, $CDCl_3$) δ 7.65 (d, J = 8.5 Hz, 2H), 7.55-7.44 (m, 4H), 7.40-7.29 (m, 3H), 6.94 (d, J = 2.4 Hz, 1H), 6.86 (d, J = 9.0 Hz, 1H), 6.65 (dd, J = 9.0, 2.5 Hz, 1H), 4.00 (t, J = 6.5 Hz, 2H), 3.81 (s, 3H), 3.63 (s, 2H), 2.36 (s, 3H), 1.90 (brs, 1H), 1.78 (brs, 1H), 1.65 (brs, 3H), 1.55-1.45 (m, 4H), 1.17-1.09 (m, 2H), 1.04-0.98 (m, 2H), 0.68 (brs, 1H) ppm.

^{13}C NMR (100 MHz, $CDCl_3$) δ 170.73, 168.15, 155.85, 139.13, 138.30, 135.75, 133.75, 131.06, 130.66, 130.51, 129.01, 128.70, 128.07, 126.47, 122.41, 114.82, 112.53, 111.45, 101.26, 64.55, 55.60, 47.15, 41.59, 31.54, 30.27, 28.28, 27.35, 25.75, 24.00, 23.05, 13.27 ppm.

HRMS (ESI) m/z [M+H]$^{+}$: calcd. 598.2467, found 598.2469.

IR (film): 3304, 3060, 2933, 2236, 1733, 1683, 1592, 835, 755, 701 cm^{-1}.

Optical rotation: $[\alpha]_D^{25}$ = -1.87 (c = 1.038, CH_2Cl_2, 96% ee).

HPLC: Chiral HPLC analysis of the 3,5-dinitrobenzoyl protected aziridine, DAICEL CHIRALPAK IA-3, hexane/*i*-PrOH = 4/2, flow rate: 0.6 mL/min, λ = 254 nm, t_R(major) = 51.7 min, t_R(minor) = 45.0 min, ee = 96%.

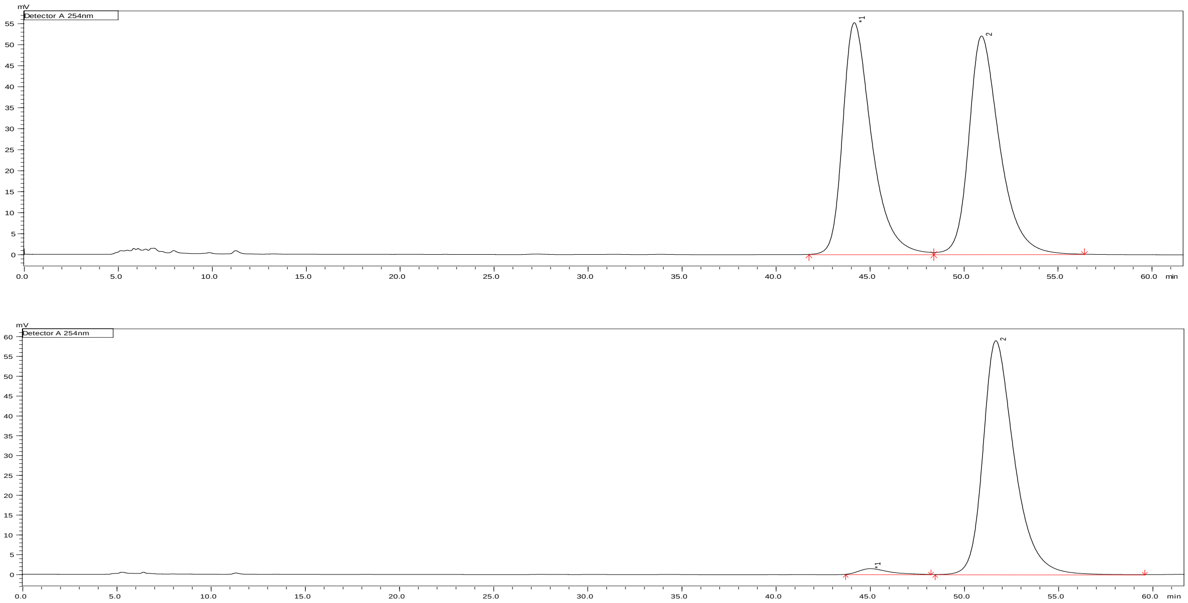

Peak#	Ret. Time	Area%
1	44.180	49.851
2	50.952	50.149

Peak#	Ret. Time	Area%
1	45.033	2.185
2	51.687	97.815

Supplementary Figure 33. HPLC chromatogram for compound **5i**

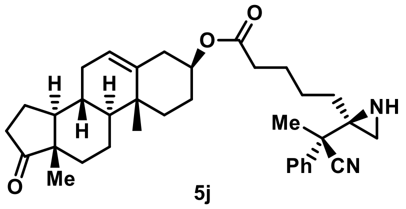

5j

5j: Procedure C, 99.8 mg, white solid, 92% yield, 14.5/1 dr.

^{1}H NMR (400 MHz, CDCl$_3$) δ 7.56-7.50 (m, 2H), 7.41-7.32 (m, 3H), 5.40 (d, J = 4.6 Hz, 1H), 4.64-4.52 (m, 1H), 2.46 (dd, J = 19.2, 8.8 Hz, 1H), 2.36-0.97 (m, 34H), 0.89 (s, 3H), 0.58 (brs, 1H) ppm.

^{13}C NMR (100 MHz, CDCl$_3$) δ 220.84, 172.47, 139.68, 138.26, 128.66, 128.60, 128.01, 126.40, 121.73, 73.45, 51.50, 49.94, 47.34, 41.46, 37.91, 36.73, 36.55, 35.68, 33.98, 31.27, 31.23, 30.60, 27.54, 24.69, 23.64, 22.96, 21.71, 20.15, 19.19, 13.39 ppm.

HRMS (ESI) m/z [M+H]$^+$: calcd. 543.3581, found 543.3583.

IR (film): 3303, 3059, 2944, 2857, 2236, 1736, 737, 701 cm^{-1}.

Optical rotation: $[\alpha]_D^{25}$ = +1.46 (c = 1.043, CH_2Cl_2, 96% de).

HPLC: DAICEL CHIRALPAK ADH connected with DAICEL CHIRALPAK IA in series, hexane/*i*-PrOH = 4/1, flow rate: 0.5 mL/min, λ = 207 nm, t_R(major) = 53.0 min, t_R(minor) = 55.8 min, de = 96%. Diastereoselectivity determined by chiral-stationary-phase HPLC analysis.

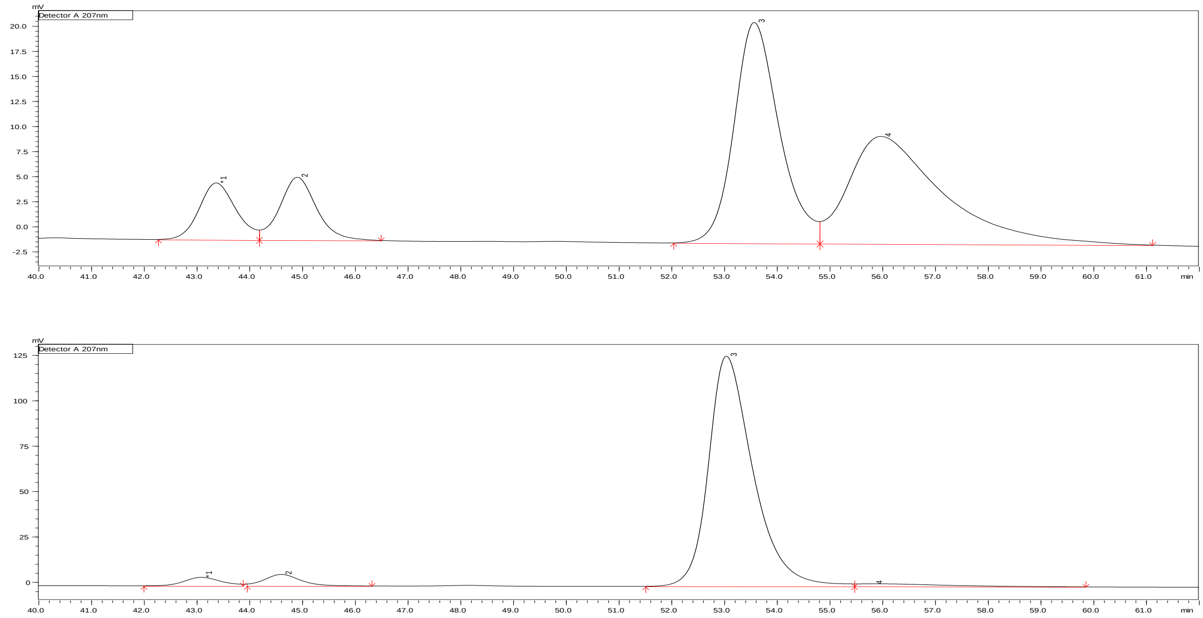

Peak#	Ret. Time	Area%
1	43.367	8.502
2	44.909	9.376
3	53.571	40.813
4	55.967	41.309

Peak#	Ret. Time	Area%
1	43.089	2.779
2	44.602	3.670
3	53.042	91.542
4	55.797	2.009

Supplementary Figure 34. HPLC chromatogram for compound **5j**

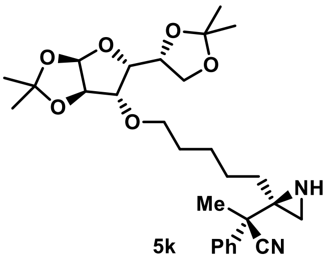

5k

5k: Procedure C, 88.2 mg, white solid, 88% yield, 13/1 dr.

^{1}H NMR (400 MHz, $CDCl_3$) δ 7.53 (d, 7.4 Hz, 2H), 7.41-7.32 (m, 3H), 5.84 (d, *J* = 3.6 Hz, 1H), 4.48 (d, *J* = 3.6 Hz, 1H), 4.24 (dd, *J* = 13.5,6.1 Hz, 1H), 4.10-4.03 (m, 2H), 3.96 (dd, *J* = 8.5, 5.9 Hz, 1H), 3.79 (d, *J* = 2.9 Hz, 1H), 3.55-3.39 (m, 2H), 1.92 (brs, 1H), 1.83 (brs, 1H), 1.66 (s, 3H), 1.61-1.56 (m, 2H), 1.49 (s, 3H), 1.47-1.42 (m, 2H), 1.41 (s, 3H), 1.31 (s, 6H), 1.26-1.17 (m, 2H), 1.10-1.00 (m, 2H), 0.57 (brs, 1H) ppm.

^{13}C NMR (100 MHz, $CDCl_3$) δ 138.34, 128.64, 128.58, 127.99, 126.40, 111.52, 108.71, 105.04, 82.27, 81.89, 80.93, 72.24, 70.08, 67.06, 41.52, 31.44, 29.28, 26.67, 26.64, 26.08, 25.92, 25.29, 23.97, 22.99 ppm.

HRMS (ESI) m/z [M+H]$^+$: calcd. 501.2959, found 501.2965.

IR (film): 3303, 3061, 2936, 2236, 1601, 1075, 760, 701 cm^{-1}.

Optical rotation: $[\alpha]_D^{25}$ = -20.46 (*c* = 1.028, CH_2Cl_2, 96% de).

HPLC: DAICEL CHIRALPAK IBN-3 connected with DAICEL CHIRALPAK IA-3 in series, hexane/*i*-PrOH = 4/1, flow rate: 0.5 mL/min, λ = 207 nm, t_R(major) = 40.0 min, t_R(minor) = 36.7 min, de = 96%. Diastereoselectivity determined by chiral-stationary-phase HPLC analysis.

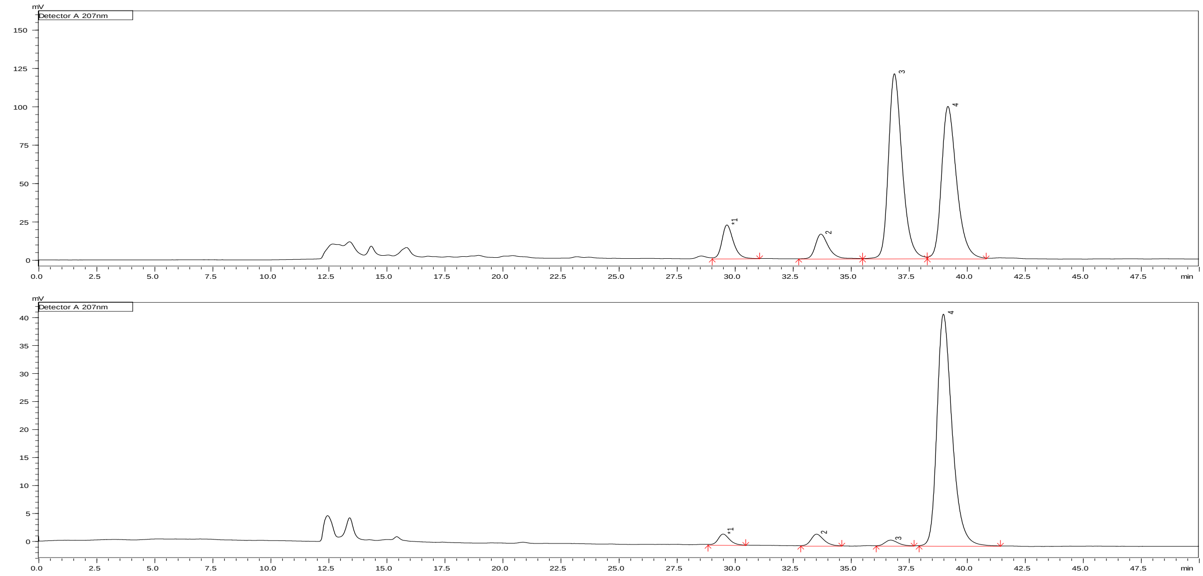

Peak#	Ret. Time	Area%
1	29.665	6.908
2	33.714	5.783
3	36.880	45.742
4	39.187	41.567

Peak#	Ret. Time	Area%
1	29.499	3.187
2	33.529	3.797
3	36.731	1.874
4	38.991	91.142

Supplementary Figure 35. HPLC chromatogram for compound **5k**

Gram-scale reaction and transformations of product 3a

Gram-scale reaction

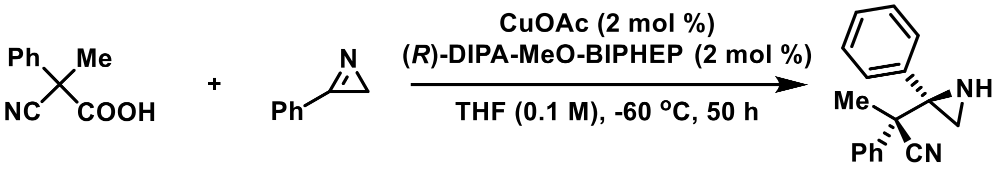

1a
1.5 equiv, 1.577 g

2a
1.0 equiv, 0.703 g

3a
1.358 g, 91%
>20/1 dr, 97% ee

A dried 250 mL round bottom flask equipped with a magnetic stirring bar was charged with CuOAc (14.7 mg, 0.12 mmol, 0.02 equiv) and (*R*)-DIPA-MeO-BIPHEP (131 mg, 0.12 mmol, 0.02 equiv) in a glove box under Ar atmosphere. Anhydrous THF (30 mL) was added via a syringe. The mixture was stirred for 15 minutes to give a clear catalyst solution. Then 2*H*-azirine **2a** (0.703 g, 6.0 mmol, 1.0 equiv) was added. After cooling to -60 °C, cyanoacetic acid **1a** (0.3 M in THF, 30 mL, 9.0 mmol, 1.577 g, 1.5 equiv) was added dropwise over 30 minutes. The resulting reaction mixture was stirred at -60 °C for 50 h. The reaction mixture was purified by silica gel column chromatography (petroleum ether/ethyl acetate = 4/1) to give product **3a** (1.358 g, 91% yield, > 20/1 dr, 97% ee) as a pale yellow solid.

Rearrangement

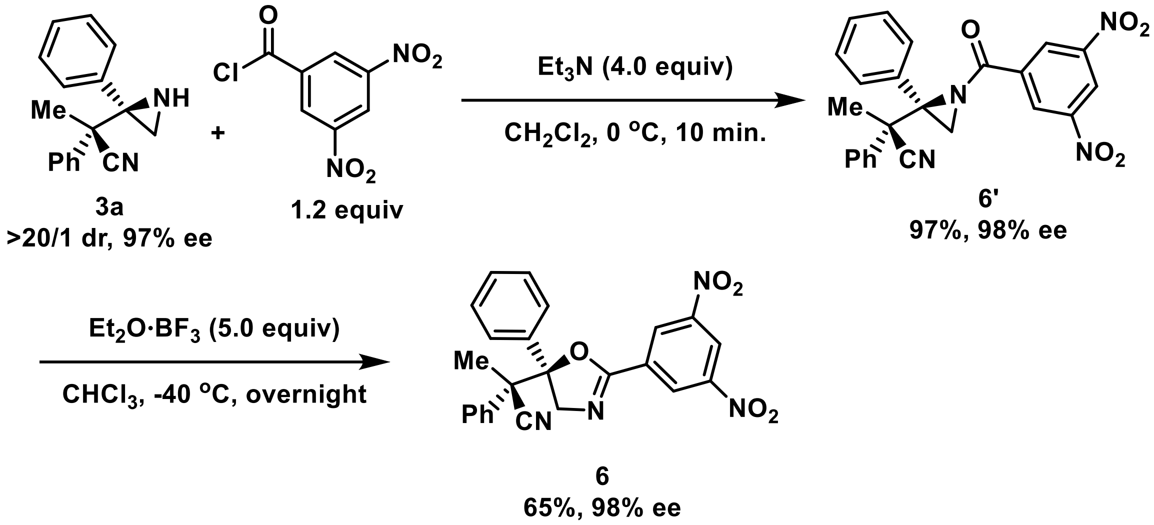

A dried 25 mL round bottom flask equipped with a magnetic stirring bar was charged with **3a** (248.3 mg, 1.0 mmol, 1.0 equiv) under N_2 atmosphere. Anhydrous DCM (10 mL, 0.1 M) was added via a syringe. Et_3N (404.8 mg, 4.0 mmol, 4.0 equiv) and 3,5-dinitrobenzoyl chloride (276.7 mg, 1.2 mmol, 1.2 equiv) were added sequentially at 0 °C. The reaction mixture was stirred for 10 minutes. After removal of solvent under reduced pressure, the crude was purified by silica gel column chromatography (petroleum ether/ethyl acetate = 6/1 to 1/1) to afford **6'** (431.2 mg, 97% yield, >20/1 dr, 98% ee) as a white solid.

A dried 25 mL Schlenk tube equipped with a magnetic stirring bar was charged with **6'** (44.2 mg, 0.1 mmol, 1.0 equiv) under N_2 atmosphere. $CHCl_3$ (2 mL, 0.05 M) was added via a syringe. The solution was then cooled to -40 °C and $Et_2O{\cdot}BF_3$ (62 μL, 0.5 mmol, 5.0 equiv) was added. The reaction mixture was stirred overnight. After removal of solvent under reduced pressure, the crude was purified by silica gel column chromatography (petroleum ether/ethyl acetate = 4/1) to afford **6** (28.6 mg, 65% yield, >20/1 dr, 98% ee) as a white solid.

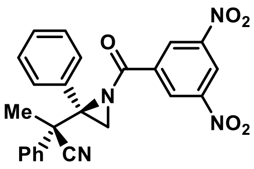

6'

^{1}H NMR (400 MHz, $CDCl_3$) δ 9.23 (s, 1H), 9.11 (s, 2H), 7.39 (s, 5H), 7.31 (t, J = 7.4 Hz, 1H), 7.17 (t, J = 7.6 Hz, 2H), 6.897 (d, J = 7.6 Hz, 2H), 3.12 (s, 1H), 3.08 (s, 1H), 2.01 (s, 3H) ppm.
^{13}C NMR (100 MHz, $CDCl_3$) δ 170.93, 148.52, 137.81, 135.67, 132.02, 130.70, 129.48, 129.10, 128.68, 128.56, 127.85, 127.37, 121.89, 121.39, 56.21, 47.20, 35.34, 22.20 ppm.
HRMS (ESI) m/z [M+H]$^+$: calcd. 443.1350, found 443.1348.
IR (film): 2955, 2924, 2238, 1656, 753, 702 cm^{-1}.
Optical rotation: $[\alpha]_D^{25}$ = +4.30 (c = 1.050, CH_2Cl_2, 98% ee).
HPLC: DAICEL CHIRALPAK ODH, hexane/i-PrOH = 4/1, flow rate: 0.5 mL/min, λ = 207 nm, t_R(major) = 67.8 min, t_R(minor) = 57.4 min, ee = 98%.

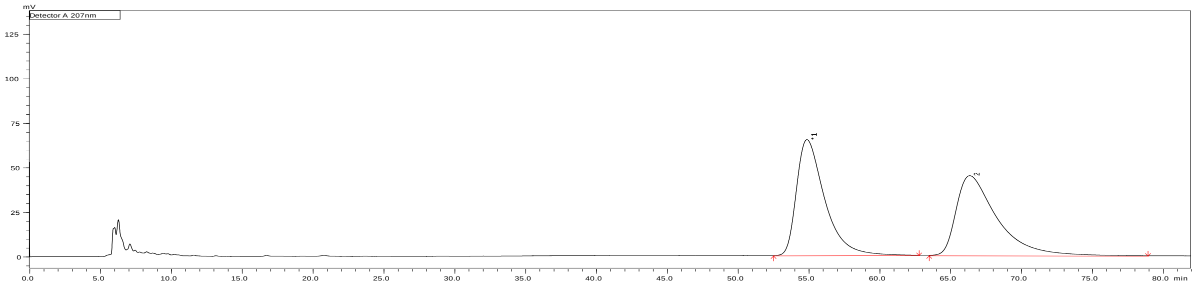

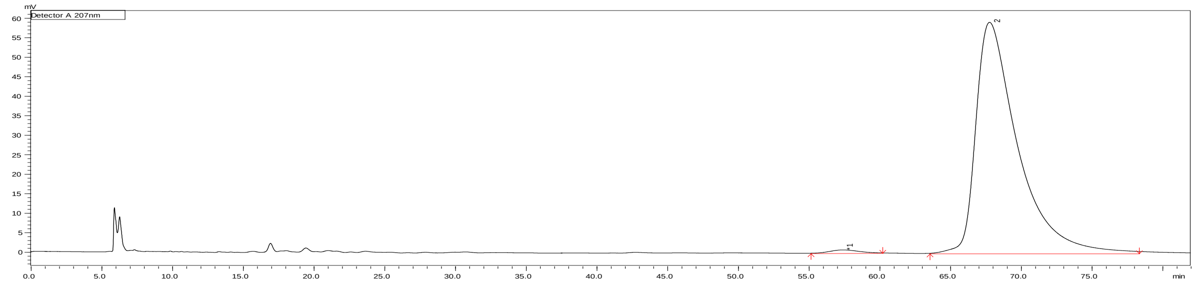

Peak#	Ret. Time	Area%
1	54.879	50.059
2	66.372	49.941

Peak#	Ret. Time	Area%
1	57.365	0.890
2	67.781	99.110

Supplementary Figure 36. HPLC chromatogram for compound **6’**

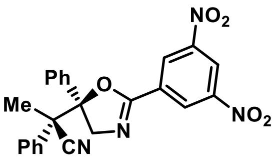

6

^{1}H NMR (400 MHz, $CDCl_3$) δ 9.18 (t, *J* = 2.0 Hz, 1H), 9.11 (d, *J* = 2.1 Hz, 2H), 7.42-7.19 (m, 8H), 7.14 (d, *J* = 6.9 Hz, 2H), 4.87 (d, *J* = 16.2 Hz, 1H), 4.43 (d, *J* = 16.3 Hz, 1H), 1.88 (s, 3H) ppm.
^{13}C NMR (100 MHz, $CDCl_3$) δ 158.86, 148.62, 138.04, 133.87, 130.73, 129.01, 128.94, 128.32, 127.91, 127.85, 127.06, 121.11, 92.87, 65.16, 51.04, 21.02 ppm.
HRMS (ESI) m/z [M+H]$^+$: calcd. 443.1350, found 443.1349.
IR (film): 3099, 2961, 2926, 2238, 1664, 1147, 731, 698 cm^{-1}.
Optical rotation: $[\alpha]_D^{25}$ = +136.96 (*c* = 1.315, CH_2Cl_2, 98% ee).
HPLC: DAICEL CHIRALPAK IA-3, hexane/*i*-PrOH = 4/1, flow rate: 0.5 mL/min, λ = 254 nm, t_R(major) = 20.7 min, t_R(minor) = 25.0 min, ee = 98%.

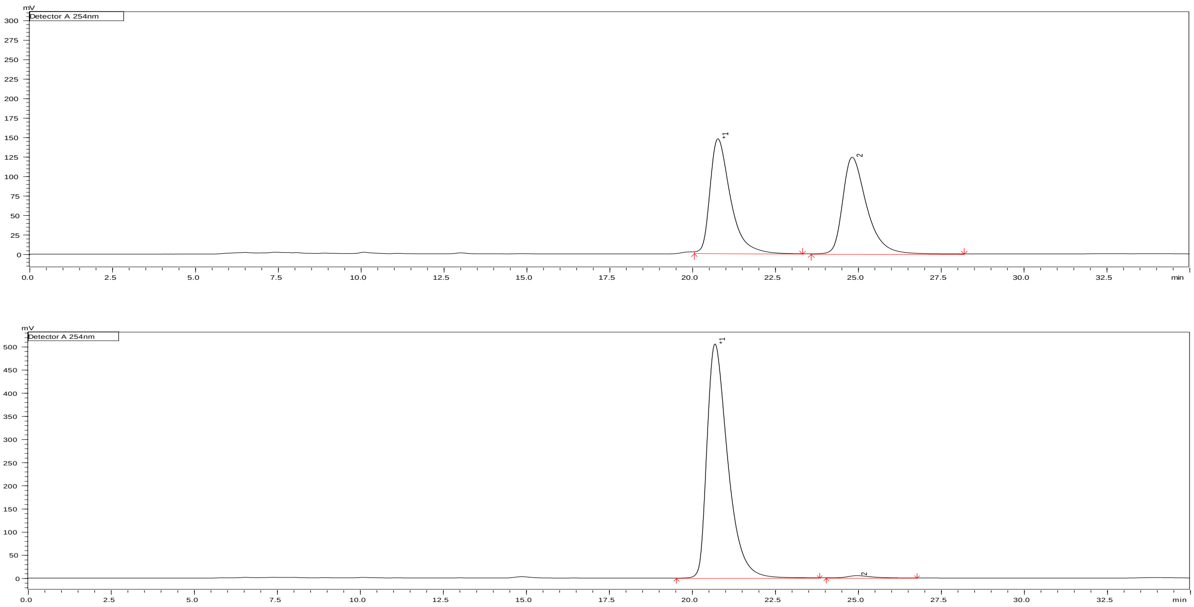

Peak#	Ret. Time	Area%
1	20.774	50.229
2	24.826	49.771

Peak#	Ret. Time	Area%
1	20.693	98.935
2	24.972	1.065

Supplementary Figure 37. HPLC chromatogram for compound **6**

DIBAL-H reduction and protection of the free amine

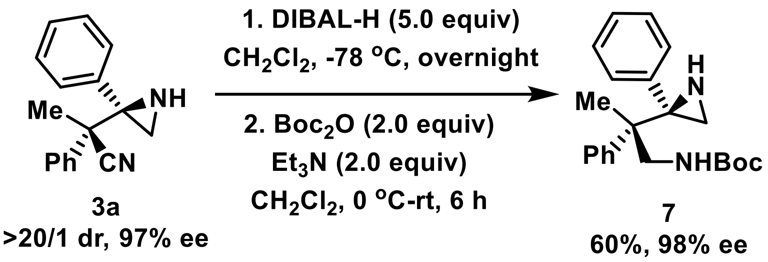

To a stirred solution of **3a** (74.5 mg, 0.3 mmol, 1.0 equiv) in anhydrous DCM (3 mL) was added dropwise DIBAL-H (1.5 M in toluene, 1.0 mL, 1.5 mmol, 5.0 equiv) under N_2 atmosphere at -78 °C. After being stirred overnight at -78 °C, the mixture was allowed to warm up to room temperature and the reaction was quenched by 60 μL water. After addition of 60 μL 15% aqueous solution of sodium hydroxide and 150 μL water, the resulting mixture was stirred for 15 minutes and dried over anhydrous $MgSO_4$. The mixture was filtered through a pack of Celite and washed with DCM. The solvent was removed under reduced pressure to give the crude product which was used in next step without further purification.

The crude was dissolved in 3 mL anhydrous DCM under N_2 atmosphere. Et_3N (60.7 mg, 0.6 mmol, 2.0 equiv) and Boc_2O (131.0 mg, 0.6 mmol, 2.0 equiv) were added sequentially at 0 °C. The reaction mixture was then allowed to warm up to room temperature and stirred for additional 6 h. After removal of solvent under reduced pressure, the crude was purified by silica gel column chromatography (petroleum ether/ethyl acetate = 3.5/1) to afford **7** (63.5 mg, 60% yield, >20/1 dr, 98% ee) as a white foam.

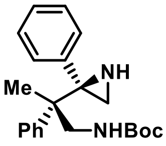

7

^{1}H NMR (400 MHz, $CDCl_3$) δ 7.30-7.23 (m, 5H), 7.16-7.08 (m, 3H), 6.94 (d, J = 6.9 Hz, 2H), 5.30 (brs, 1H), 3.58-3.43 (m, 2H), 2.13 (s, 1H), 1.89 (s, 1H), 1.43 (s, 3H), 1.40 (s, 9H), 0.79 (brs, 1H) ppm.
^{13}C NMR (100 MHz, $CDCl_3$) δ 156.09, 142.73, 141.44, 129.82, 127.97, 127.30, 127.27, 127.17, 126.69, 78.81, 48.56, 47.38, 44.43, 29.32, 28.33, 20.09 ppm.
HRMS (ESI) m/z [M+H]$^+$: calcd. 353.2224, found 353.2224.
IR (film): 3449, 3301, 2976, 1710, 1495, 1365, 1244, 1171, 702 cm^{-1}.
Optical rotation: $[\alpha]_D^{25}$ = +0.97 (c = 1.088, CH_2Cl_2, 98% ee).
HPLC: DAICEL CHIRALPAK ADH, hexane/*i*-PrOH = 47/3, flow rate: 0.5 mL/min, λ = 207 nm, t_R(major) = 20.5 min, t_R(minor) = 23.6 min, ee = 98%.

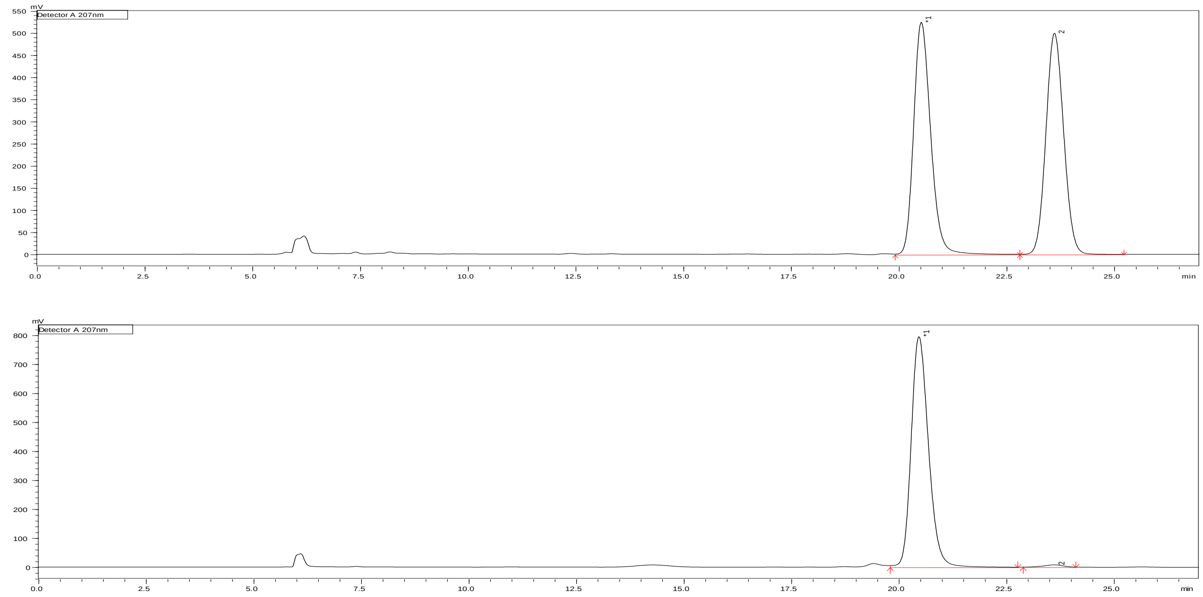

Peak#	Ret. Time	Area%
1	20.522	50.246
2	23.612	49.754

Peak#	Ret. Time	Area%
1	20.468	98.850
2	23.613	1.150

Supplementary Figure 38. HPLC chromatogram for compound **7**

Aziridine-opening with HCl

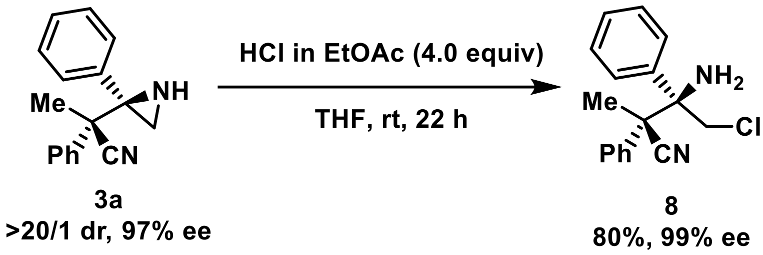

A dried 25 mL round bottom flask equipped with a magnetic stirring bar was charged with **3a** (49.7 mg, 0.2 mmol, 1.0 equiv) under N_2 atmosphere. Anhydrous THF (1.0 mL) was added via a syringe. HCl (0.4 M in EtOAc, 2.0 mL, 0.8 mmol, 4.0 equiv) was added and the reaction mixture was stirred for 22 h at room temperature. Then the reaction was quenched with saturated aqueous $NaHCO_3$ and extracted with EtOAc (10 mL × 3). The organic extracts were dried over anhydrous Na_2SO_4 and the solvent was removed under reduced pressure to give the crude product. The residue was purified by silica gel column chromatography (petroleum ether/ethyl acetate = 9/1) to afford **8** (45.8 mg, 80% yield, >20/1 dr, 99% ee) as a colorless solid.

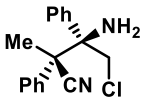

8

^{1}H NMR (400 MHz, CDCl$_3$) δ 7.34-7.24 (m, 6H), 7.14 (d, *J* = 6.2 Hz, 2H), 7.06 (d, *J* = 7.4 Hz, 2H), 4.22 (d, *J* = 11.4 Hz, 1H), 3.96 (d, *J* = 11.4 Hz, 1H), 1.93 (s, 2H), 1.80 (s, 3H) ppm.
^{13}C NMR (100 MHz, CDCl$_3$) δ 136.50, 135.40, 128.57, 128.56, 128.18, 128.13, 127.97, 127.45, 122.88, 63.72, 52.41, 51.41, 22.24 ppm.
HRMS (ESI) m/z [M+H]$^+$: calcd. 285.1153, found 285.1153.
IR (film): 3380, 3318, 3090, 2925, 2234, 1600, 754, 702 cm^{-1}.
Optical rotation: $[\alpha]_D^{25}$ = +19.55 (*c* = 0.950, CH_2Cl_2, 99% ee).
HPLC: DAICEL CHIRALPAK IA, hexane/*i*-PrOH = 24/1, flow rate: 0.5 mL/min, λ = 207 nm, t_R(major) = 13.4 min, t_R(minor) = 15.6 min, ee = 99%.

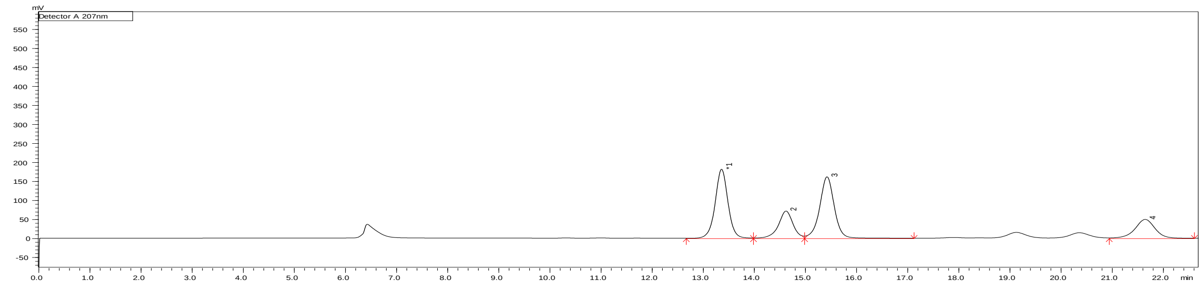

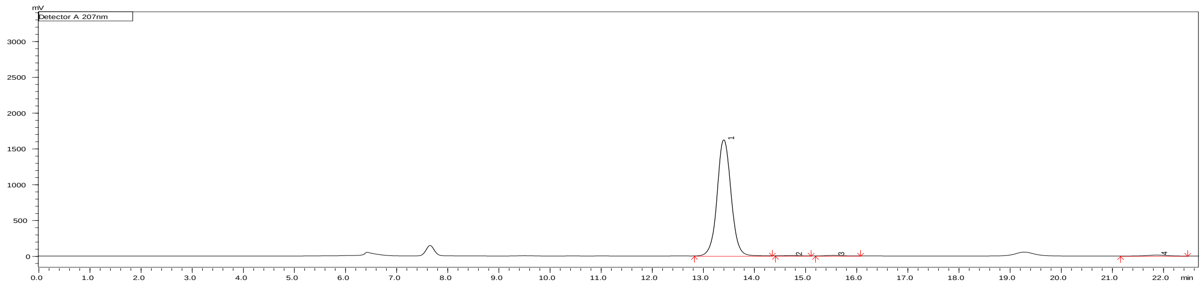

Peak#	Ret. Time	Area%
1	13.365	34.700
2	14.626	15.552
3	15.428	35.098
4	21.653	14.650

Peak#	Ret. Time	Area%
1	13.406	98.114
2	14.728	0.241
3	15.560	0.491
4	21.877	1.155

Supplementary Figure 39. HPLC chromatogram for compound **8**

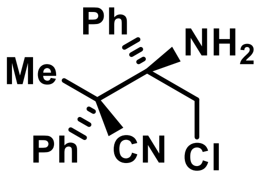

8

A diethyl ether solution of **8** (>20/1 dr, 99% ee) was allowed to grow crystals at room temperature, which led to the determination of the absolute configuration of stereocenters in **8** through X-ray analysis. CCDC number **1891666** contains the supplementary crystallographic data for this report. These data can be obtained free of charge from The Cambridge Crystallographic Data Center via www.ccdc.cam.ac.uk/data_request/cif.

The absolute configurations of the stereocenters in **8** were determined by the X-ray analysis of its single crystals, which led to the determination of the exact stereochemistry of **3a**. According to the section of *"Comparison of the reaction performance between (R)-DTBM-SEGPHOS and (R)-DIPA-MeO-BIPHEP"* **in Supplementary Methods**, the absolute configurations of **3a** and **5a** with (*R*)-DTBM-SEGPHOS and (*R*)-DIPA-MeO-BIPHEP were identical. Then, the absolute configurations in **3** and **5** were assigned tentatively by analogy.

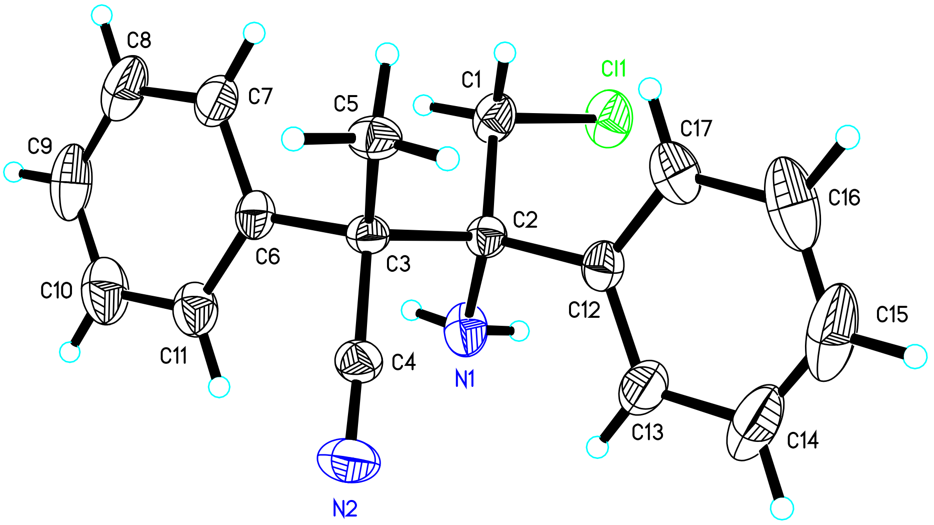

Supplementary Figure 40. The full numbering scheme of CCDC: 1891666

Supplementary Table 4. Crystal data and structure refinement for mo_d8v19066_0m

Identification code	mo_d8v19066_0m	
Empirical formula	C17 H17 Cl N2	
Formula weight	284.77	
Temperature	293(2) K	
Wavelength	0.71073 Å	
Crystal system	Monoclinic	
Space group	P 21	
Unit cell dimensions	a = 6.7694(2) Å	α= 90°.
	b = 10.8521(4) Å	β= 94.2730(10)°.
	c = 10.2350(4) Å	γ = 90°.
Volume	749.80(5) Å^3	
Z	2	
Density (calculated)	1.261 Mg/m^3	
Absorption coefficient	0.246 mm^{-1}	
F(000)	300	
Crystal size	0.200 x 0.160 x 0.130 mm^3	
Theta range for data collection	3.018 to 25.997°.	
Index ranges	-8<=h<=8, -13<=k<=13, -12<=l<=12	
Reflections collected	11335	
Independent reflections	2941 [R(int) = 0.0433]	
Completeness to theta = 25.242°	99.2 %	
Absorption correction	Semi-empirical from equivalents	
Max. and min. transmission	0.7456 and 0.6204	
Refinement method	Full-matrix least-squares on F^2	
Data / restraints / parameters	2941 / 1 / 191	
Goodness-of-fit on F^2	1.017	
Final R indices [I>2sigma(I)]	R1 = 0.0333, wR2 = 0.0866	
R indices (all data)	R1 = 0.0364, wR2 = 0.0899	
Absolute structure parameter	-0.01(3)	
Largest diff. peak and hole	0.125 and -0.134 e.Å^{-3}	

Supplementary Figures of NMR Spectra

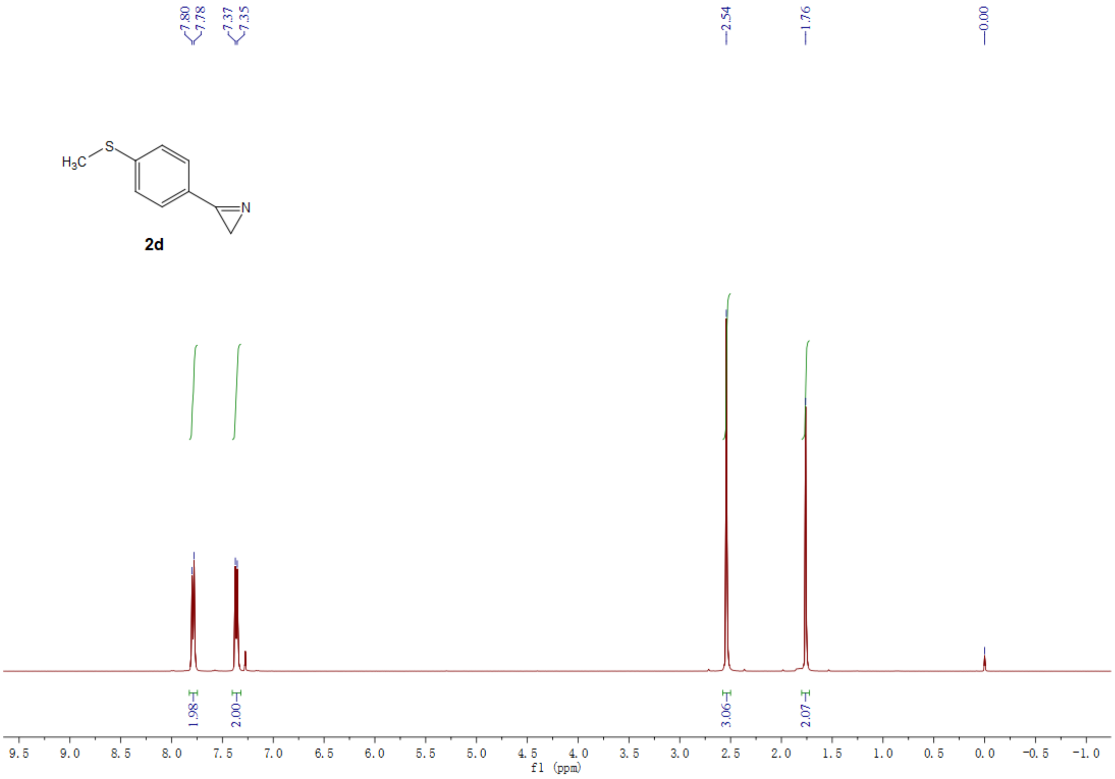

Supplementary Figure 41. ^{1}H NMR spectrum for compound **2d**

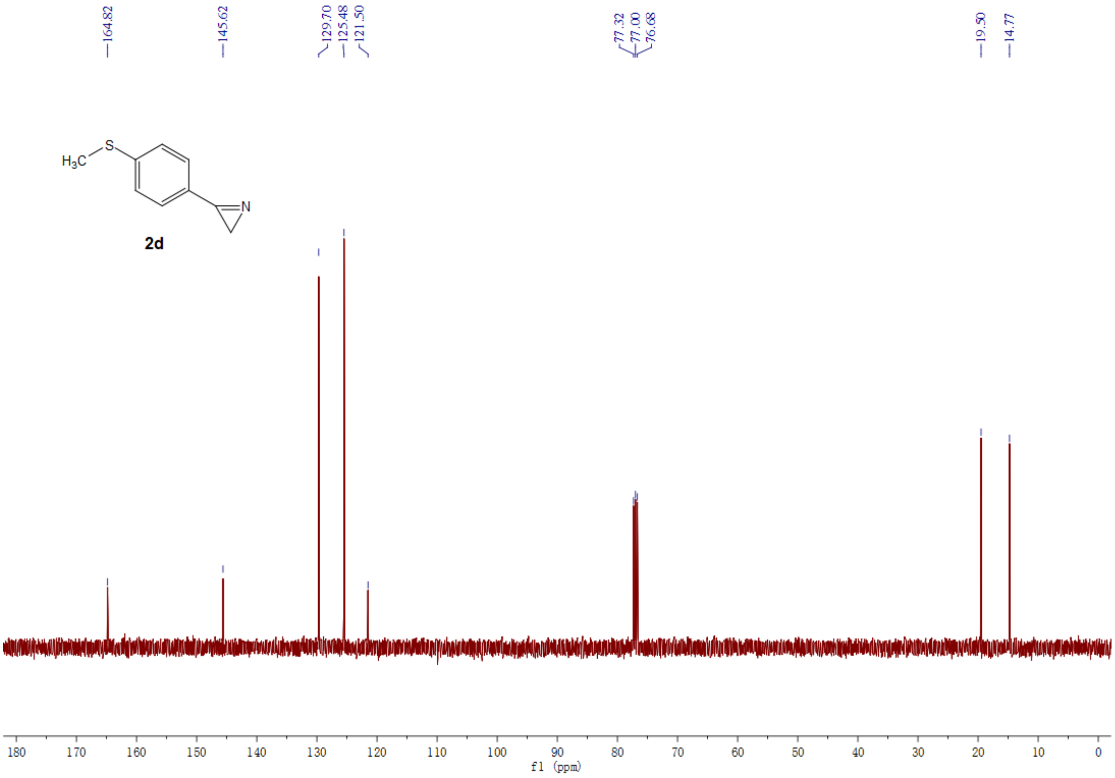

Supplementary Figure 42. ^{13}C NMR spectrum for compound **2d**

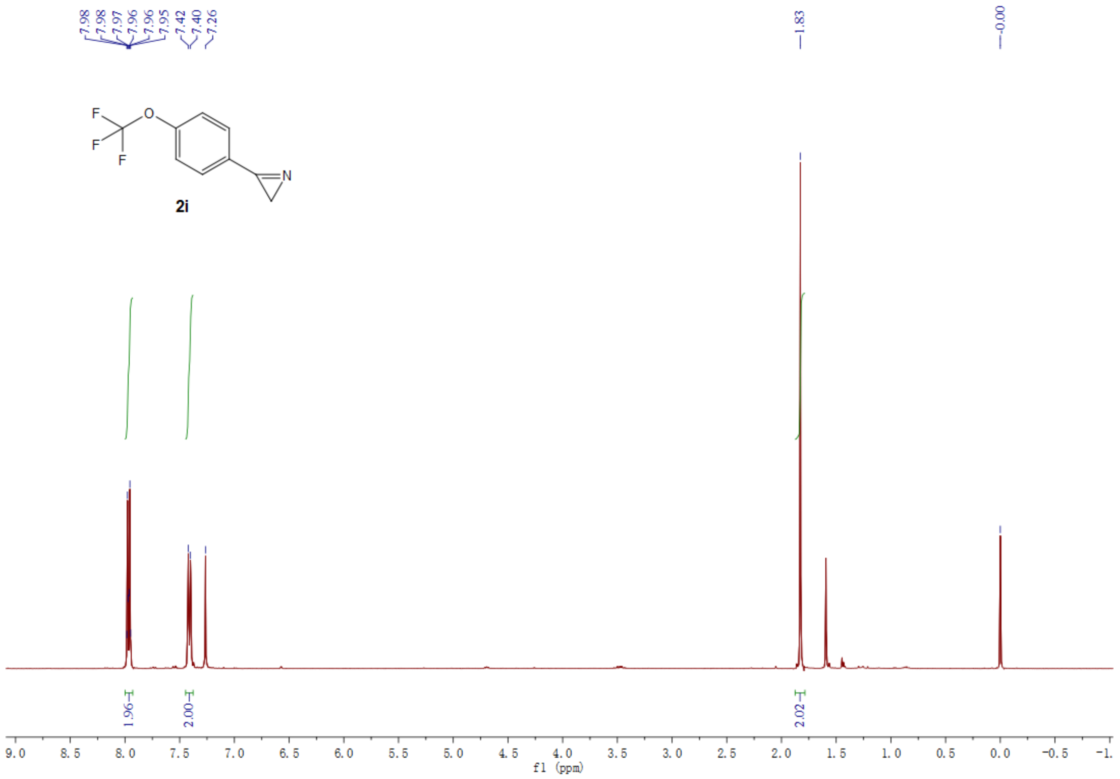

Supplementary Figure 43. ^{1}H NMR spectrum for compound **2i**

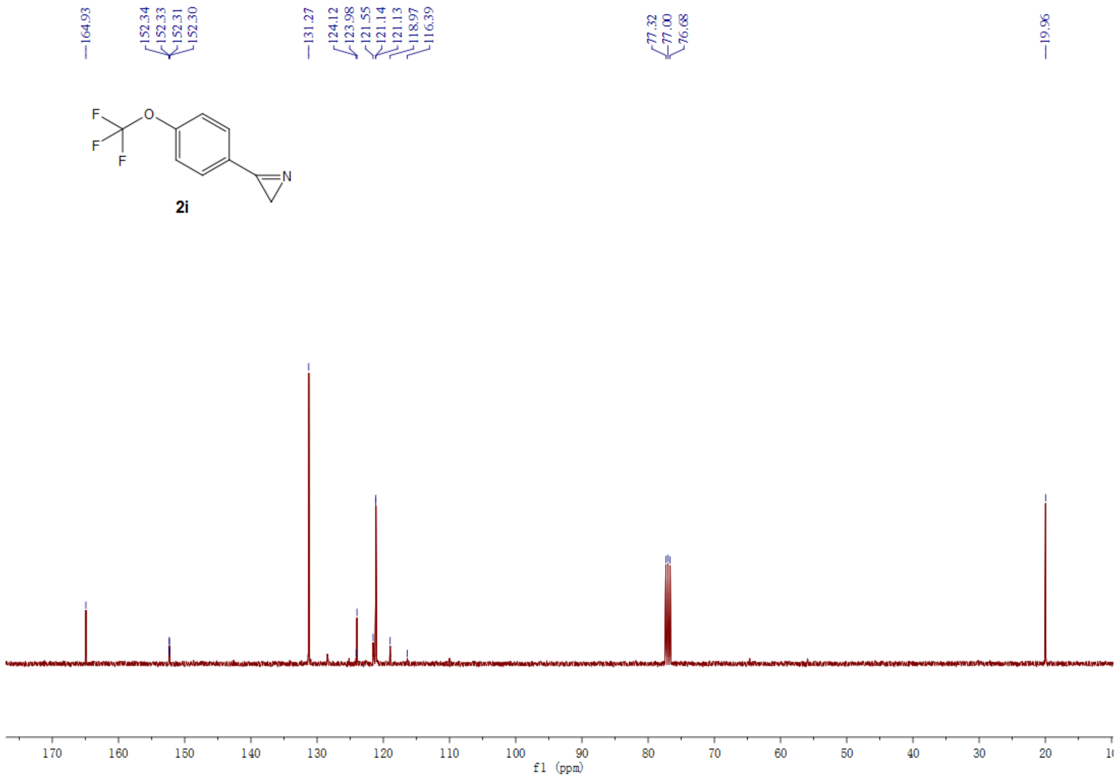

Supplementary Figure 44. ^{13}C NMR spectrum for compound **2i**

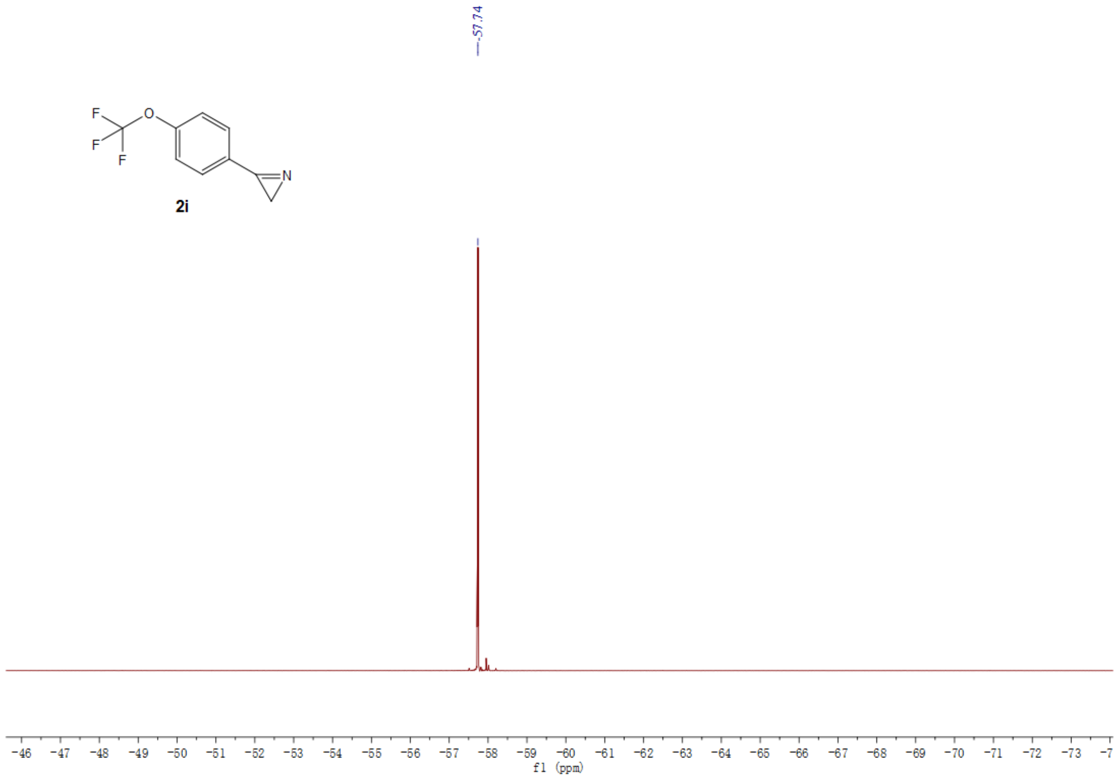

Supplementary Figure 45. ^{19}F NMR spectrum for compound **2i**

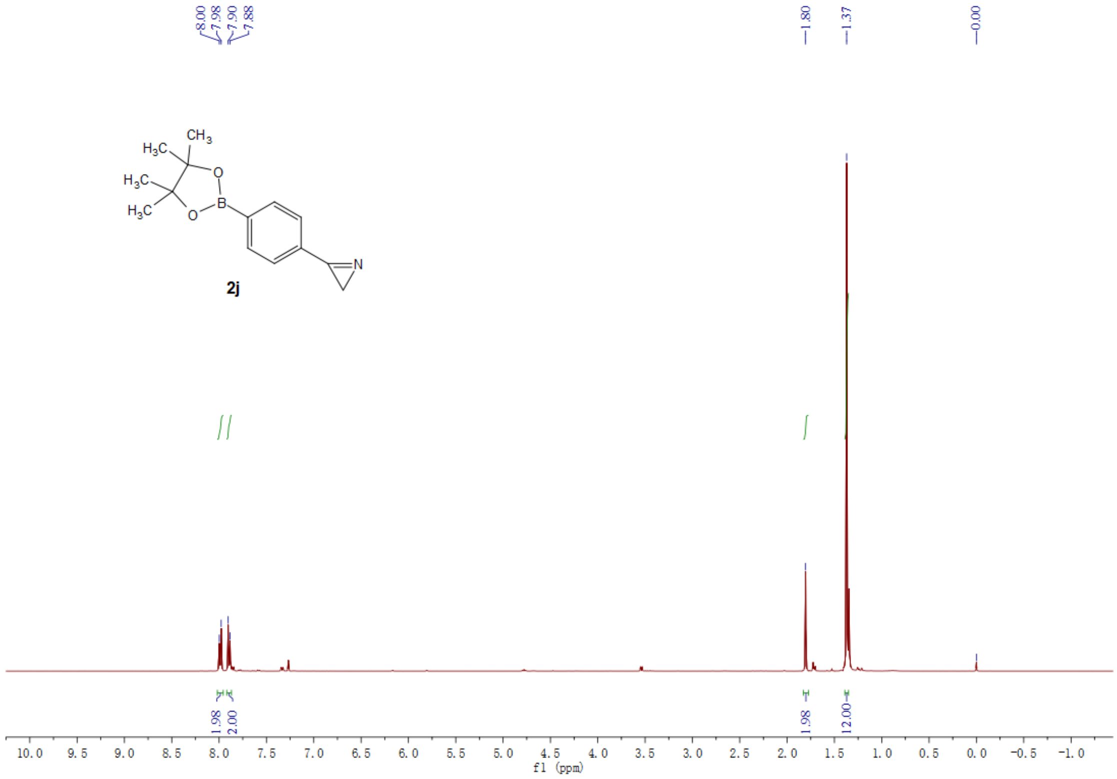

Supplementary Figure 46. ^{1}H NMR spectrum for compound **2j**

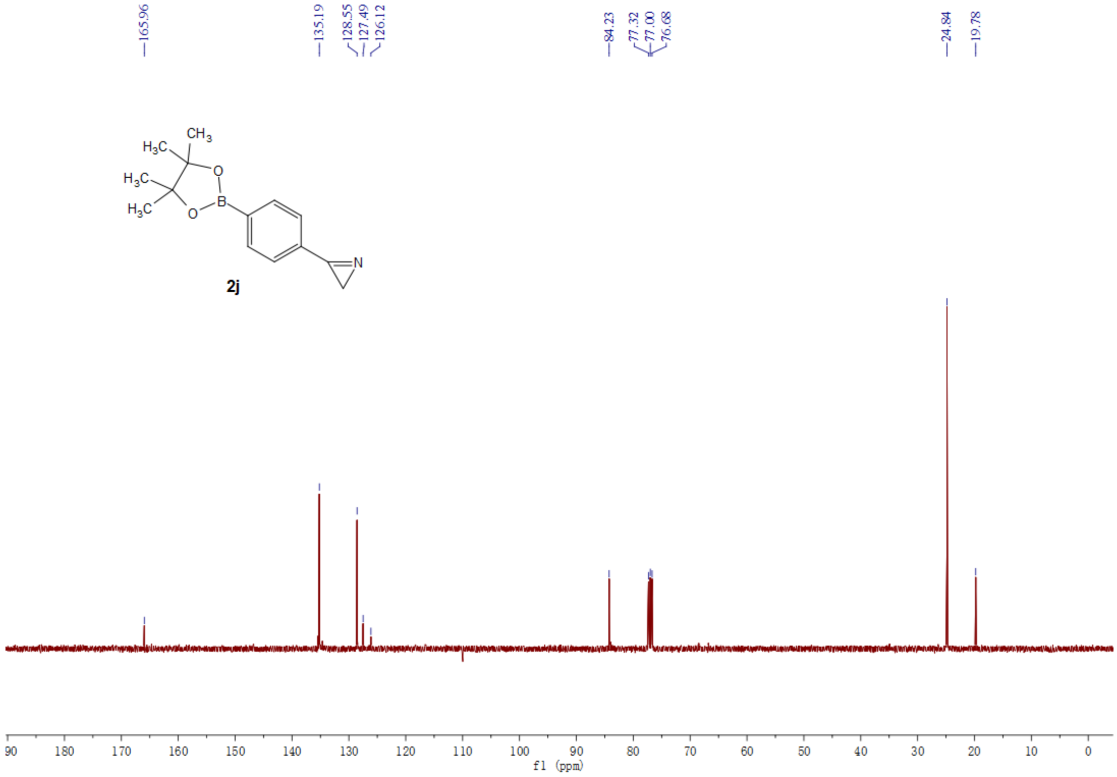

Supplementary Figure 47. ^{13}C NMR spectrum for compound **2j**

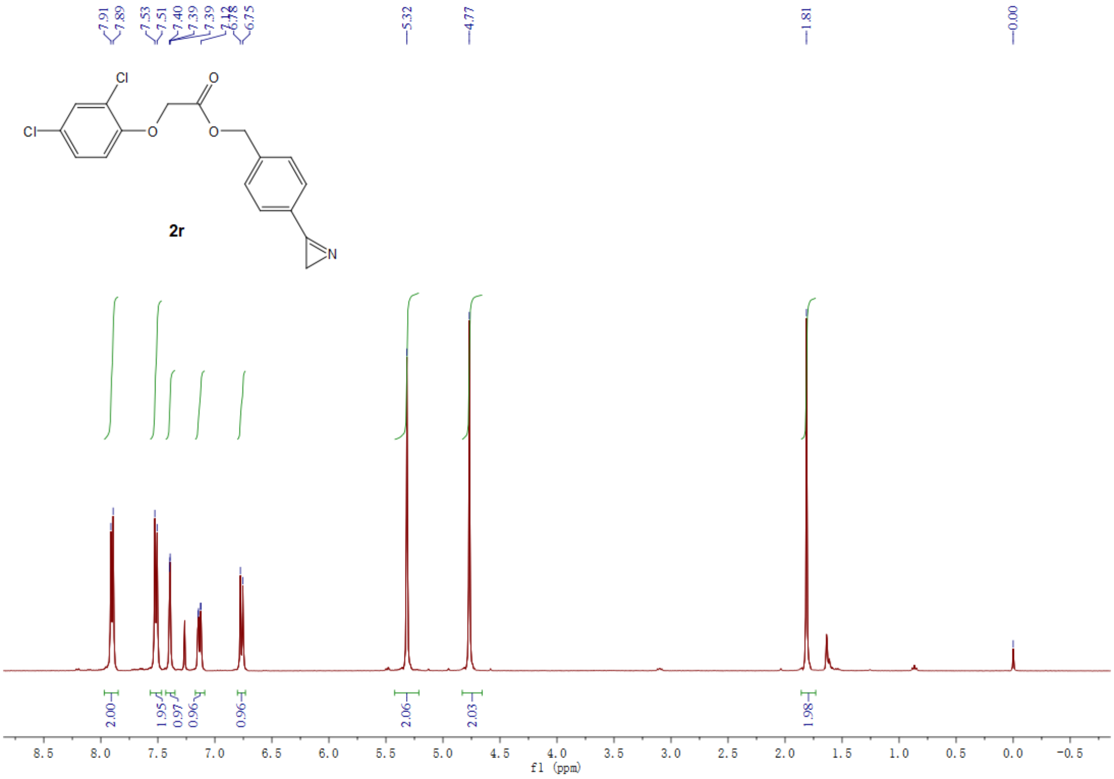

Supplementary Figure 48. ^{1}H NMR spectrum for compound **2r**

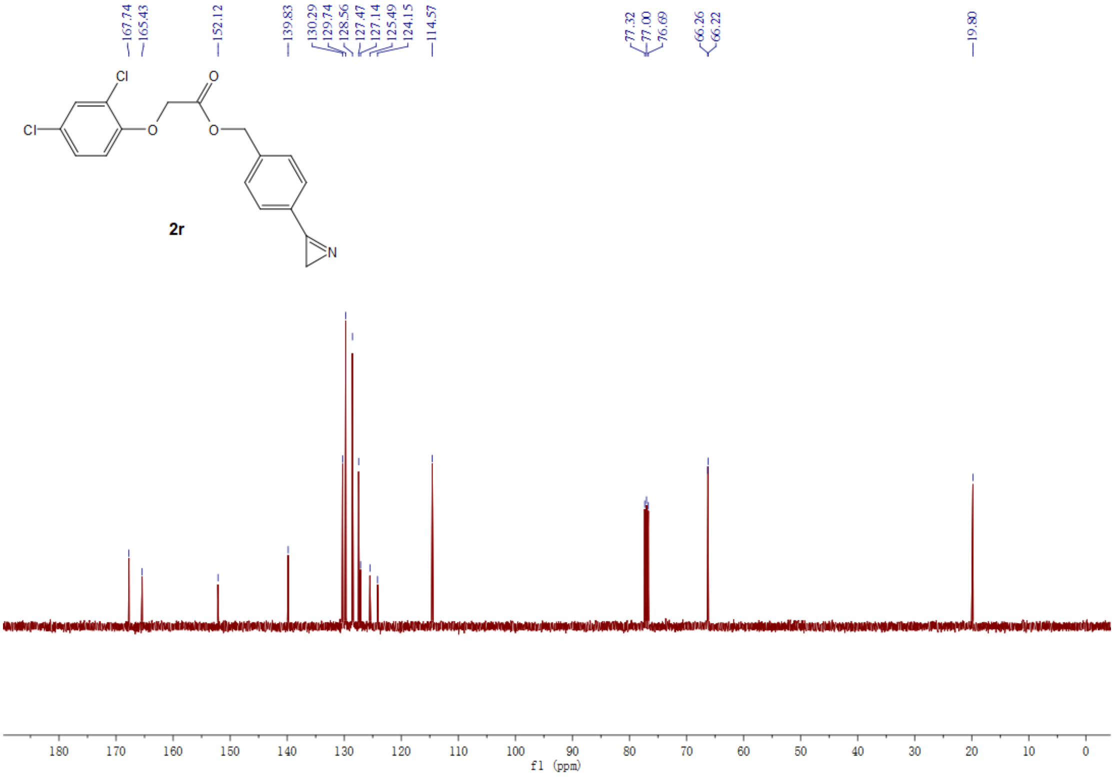

Supplementary Figure 49. ^{13}C NMR spectrum for compound **2r**

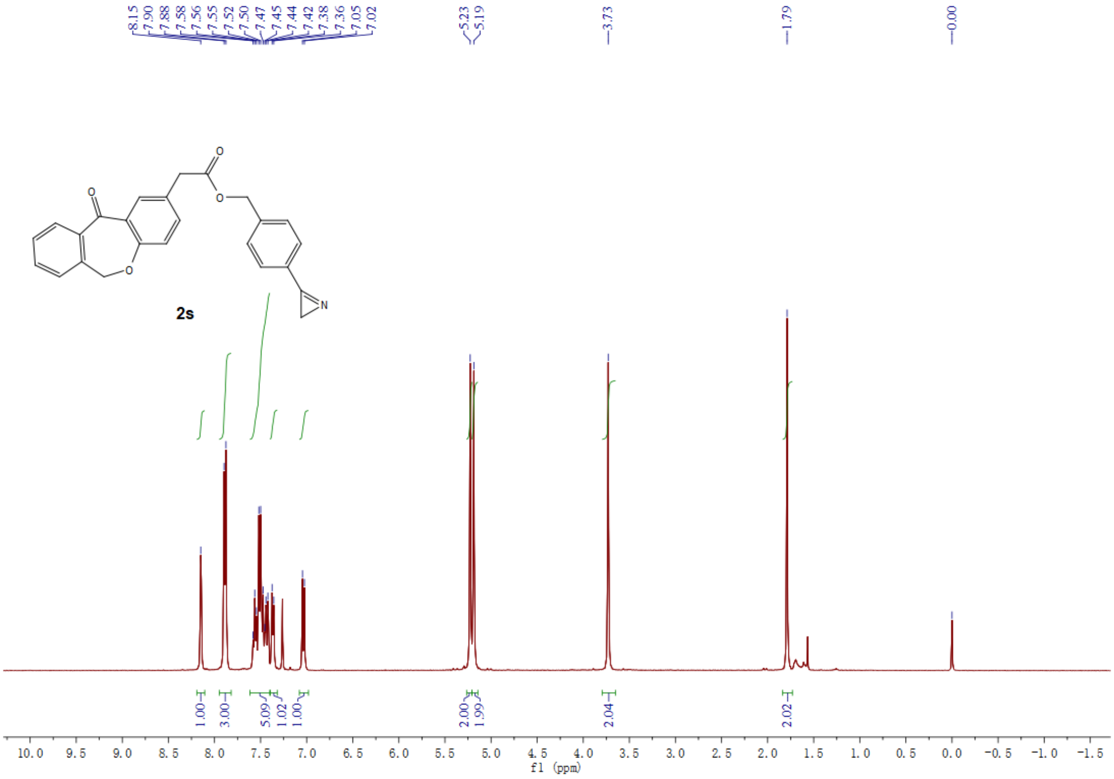

Supplementary Figure 50. ¹H NMR spectrum for compound **2s**

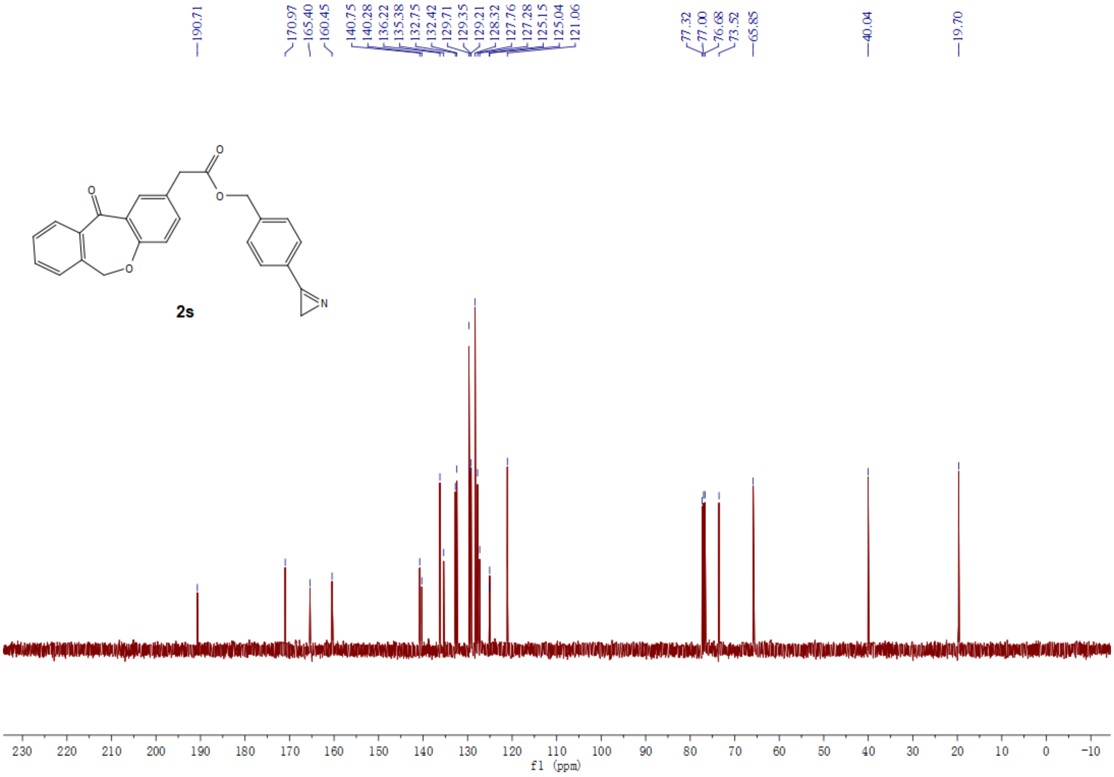

Supplementary Figure 51. ¹³C NMR spectrum for compound **2s**

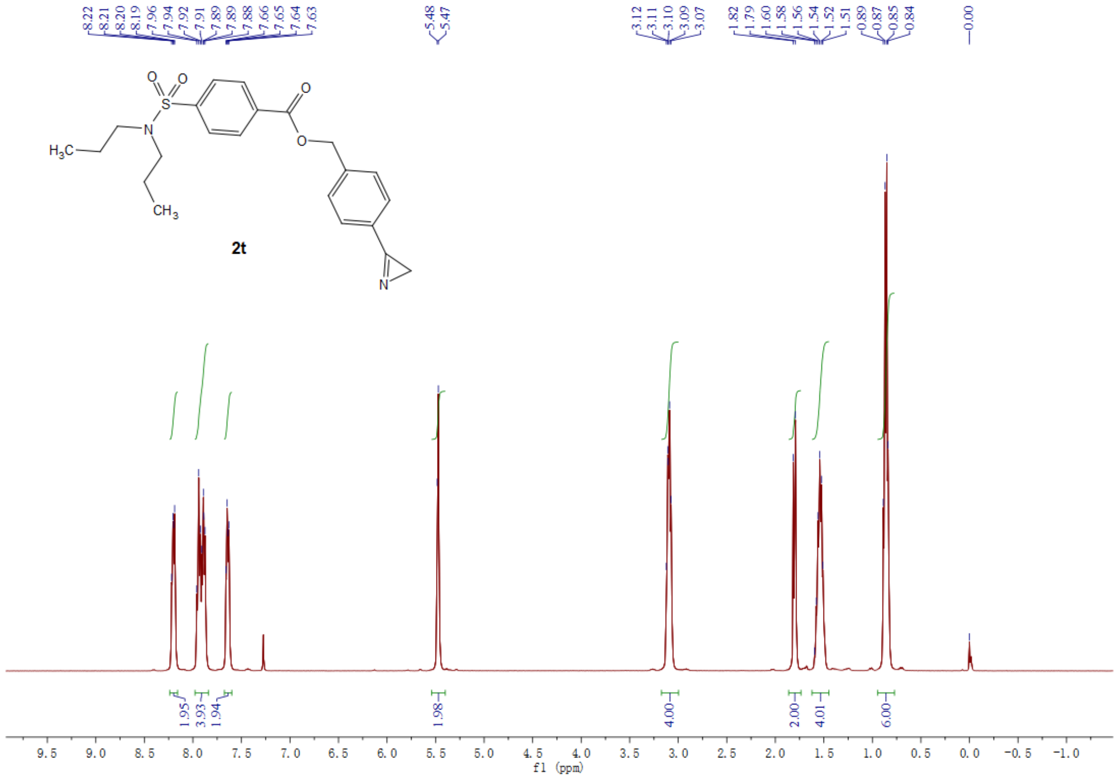

Supplementary Figure 52. ^{1}H NMR spectrum for compound **2t**

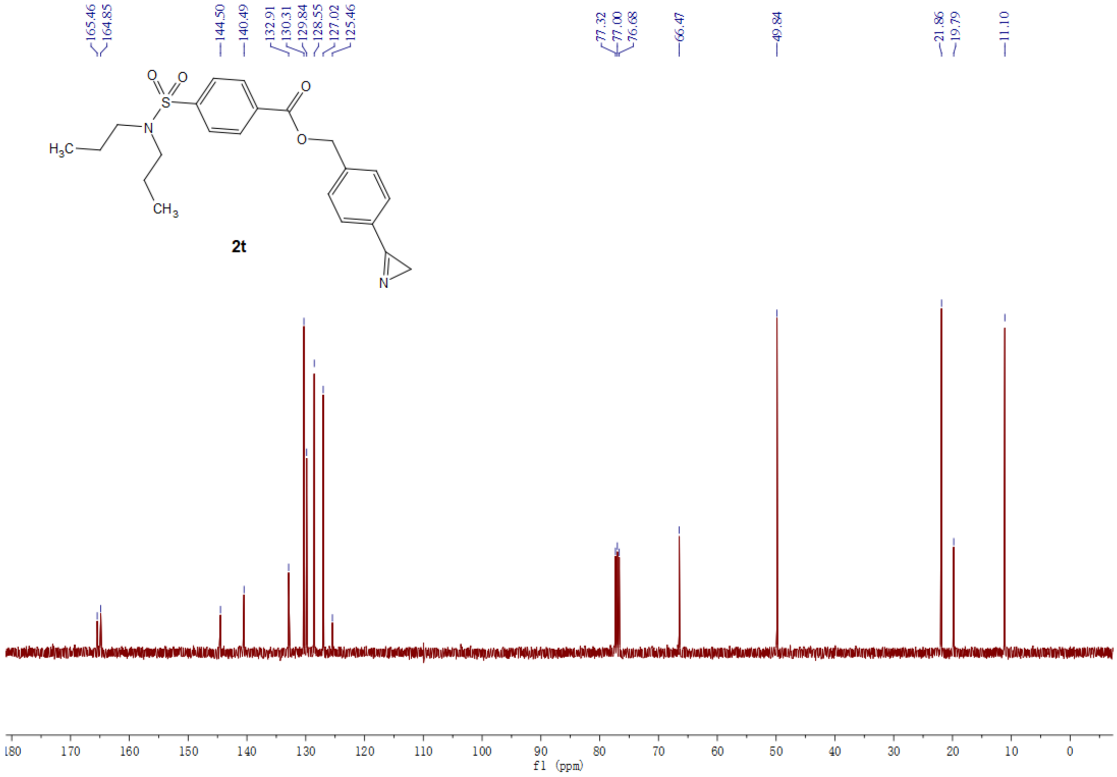

Supplementary Figure 53. ^{13}C NMR spectrum for compound **2t**

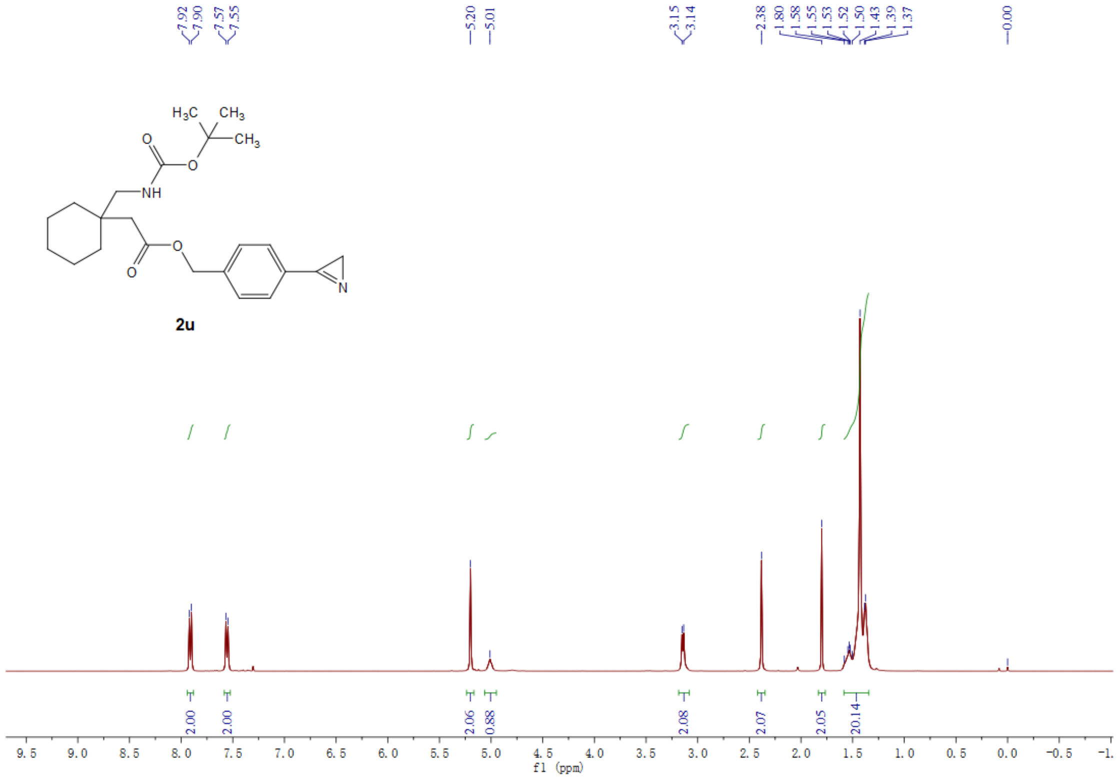

Supplementary Figure 54. ^{1}H NMR spectrum for compound **2u**

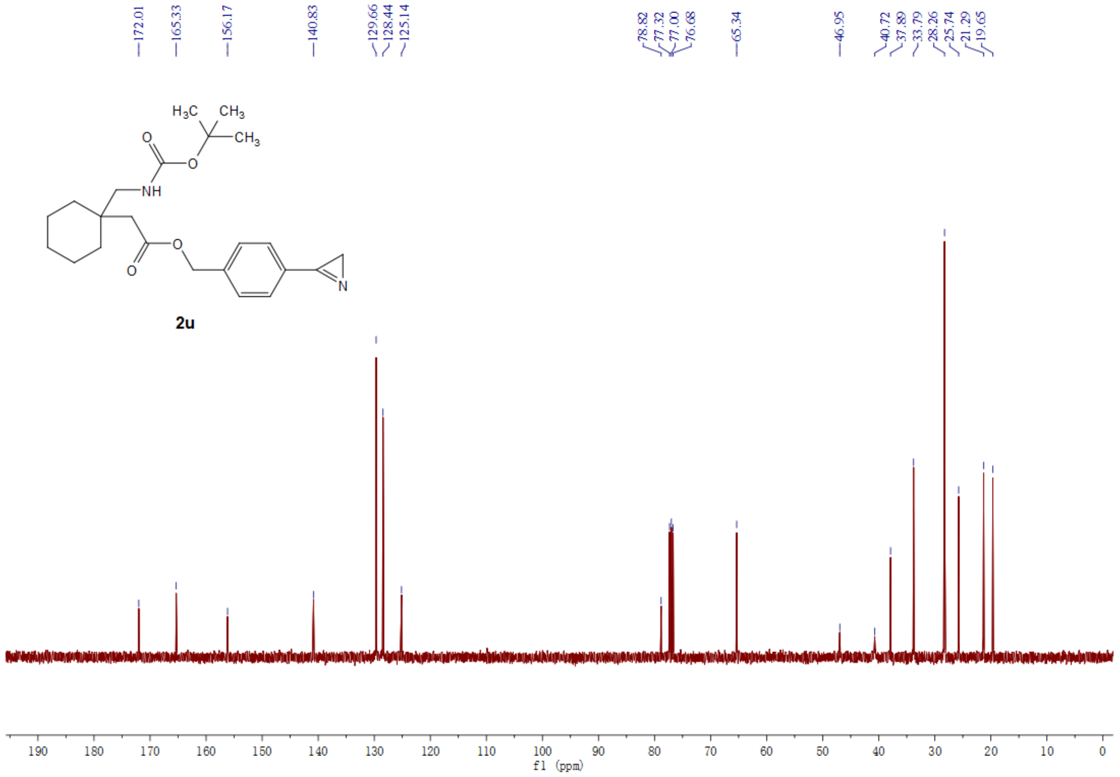

Supplementary Figure 55. ^{13}C NMR spectrum for compound **2u**

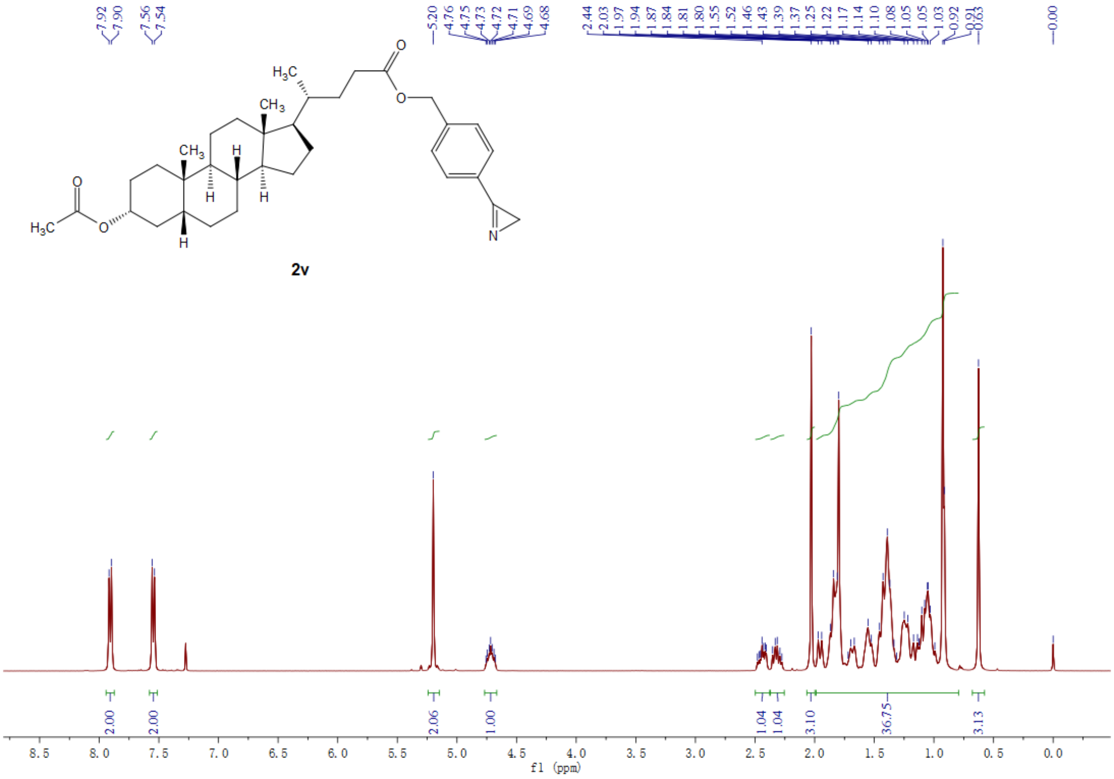

Supplementary Figure 56. ^{1}H NMR spectrum for compound **2v**

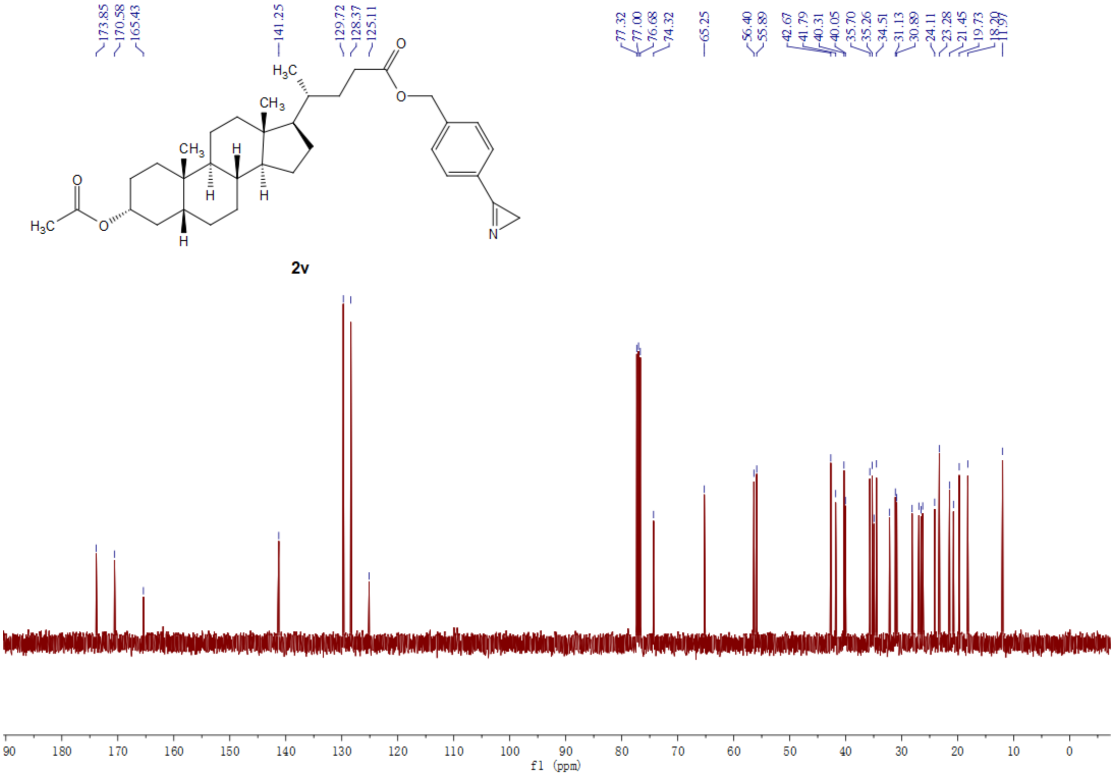

Supplementary Figure 57. ^{13}C NMR spectrum for compound **2v**

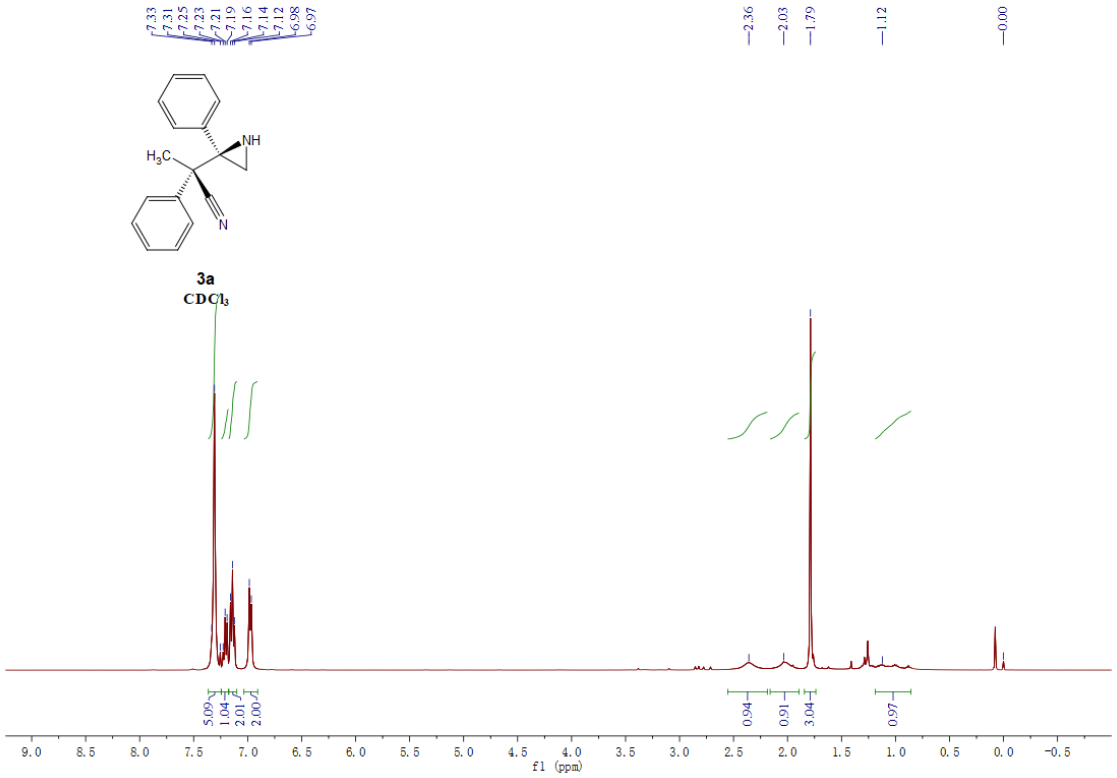

Supplementary Figure 58. ^{1}H NMR spectrum for compound **3a**

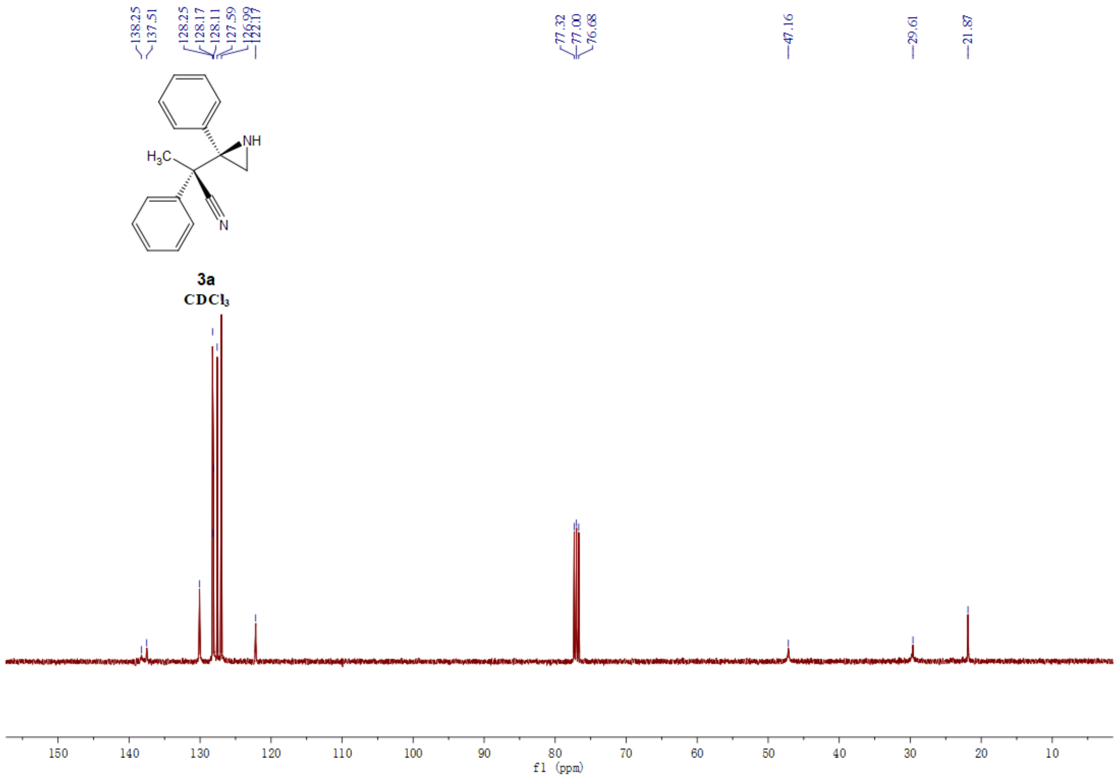

Supplementary Figure 59. ^{13}C NMR spectrum for compound **3a**

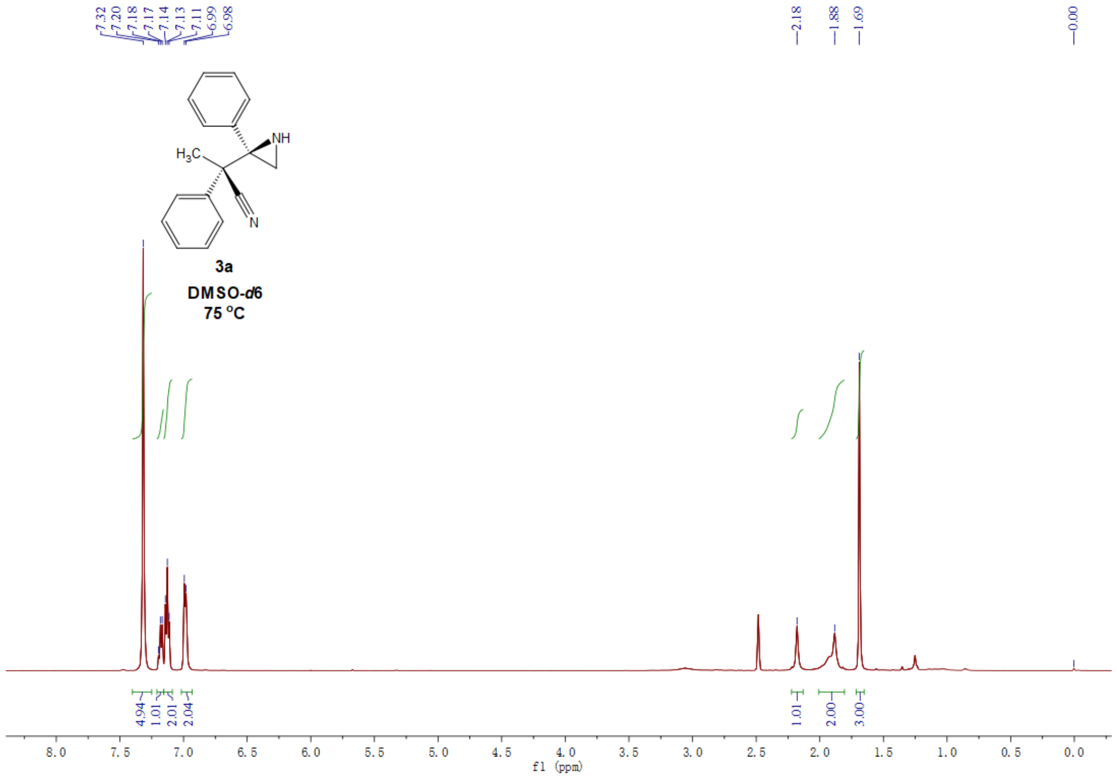

Supplementary Figure 60. ^{1}H NMR spectrum for compound **3a**

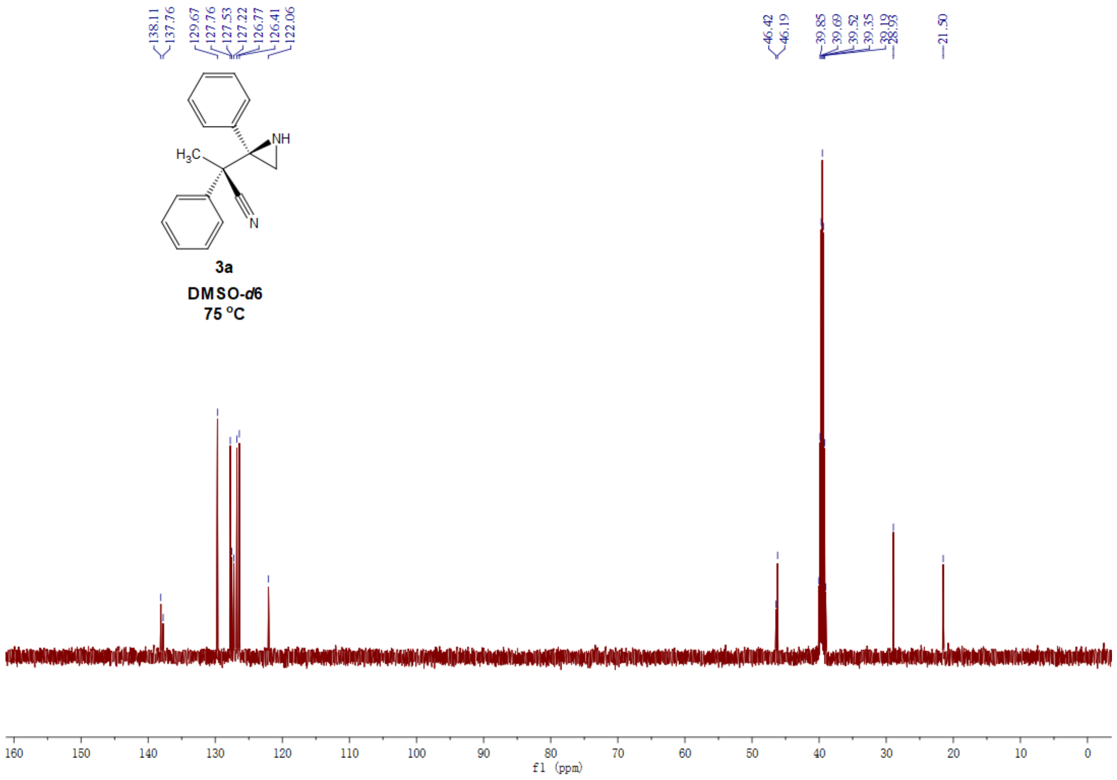

Supplementary Figure 61. ^{13}C NMR spectrum for compound **3a**

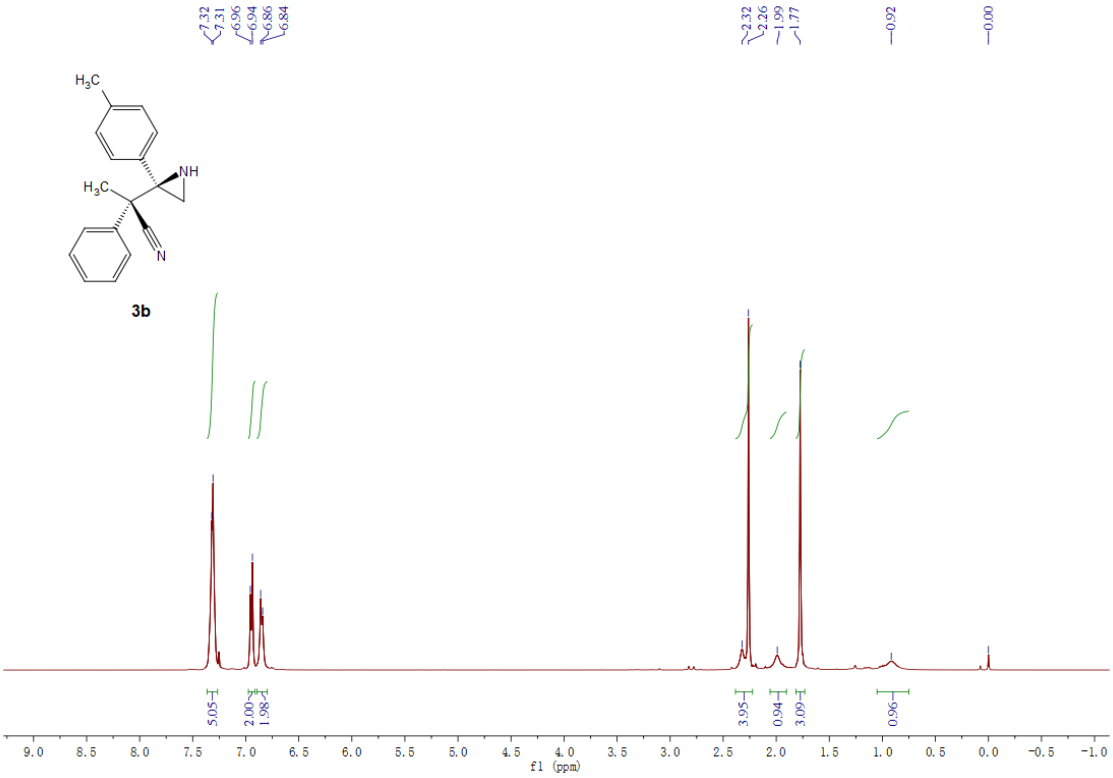

Supplementary Figure 62. ^{1}H NMR spectrum for compound **3b**

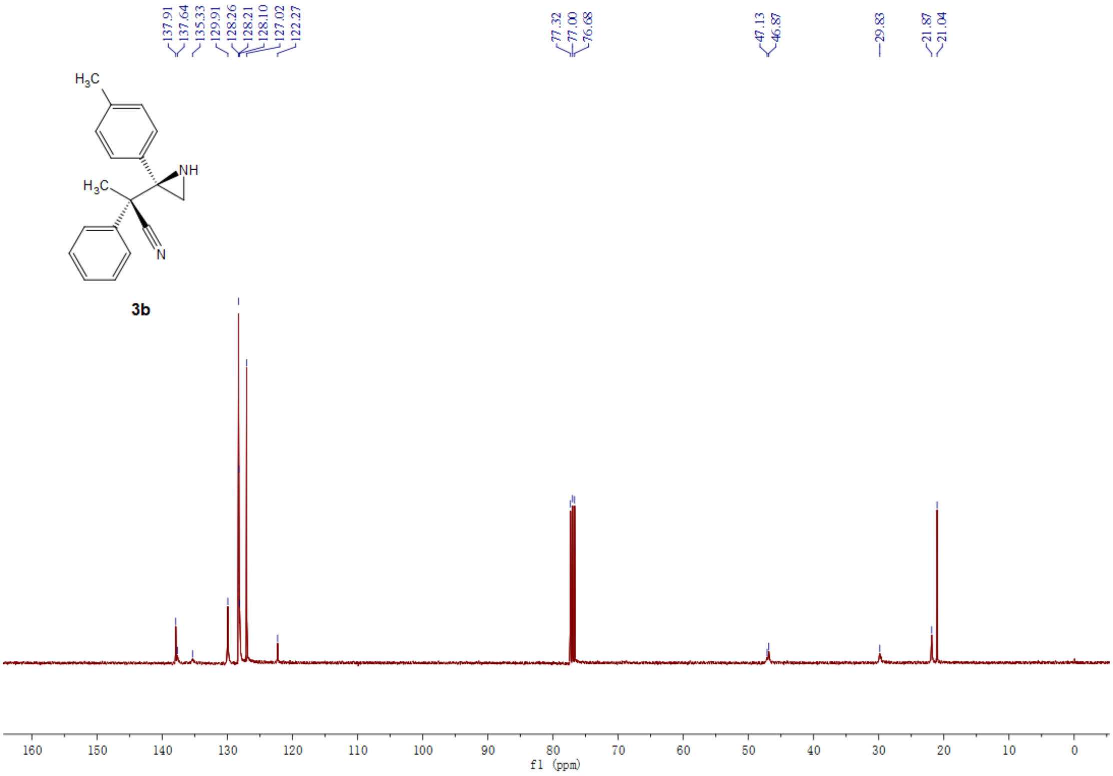

Supplementary Figure 63. ^{13}C NMR spectrum for compound **3b**

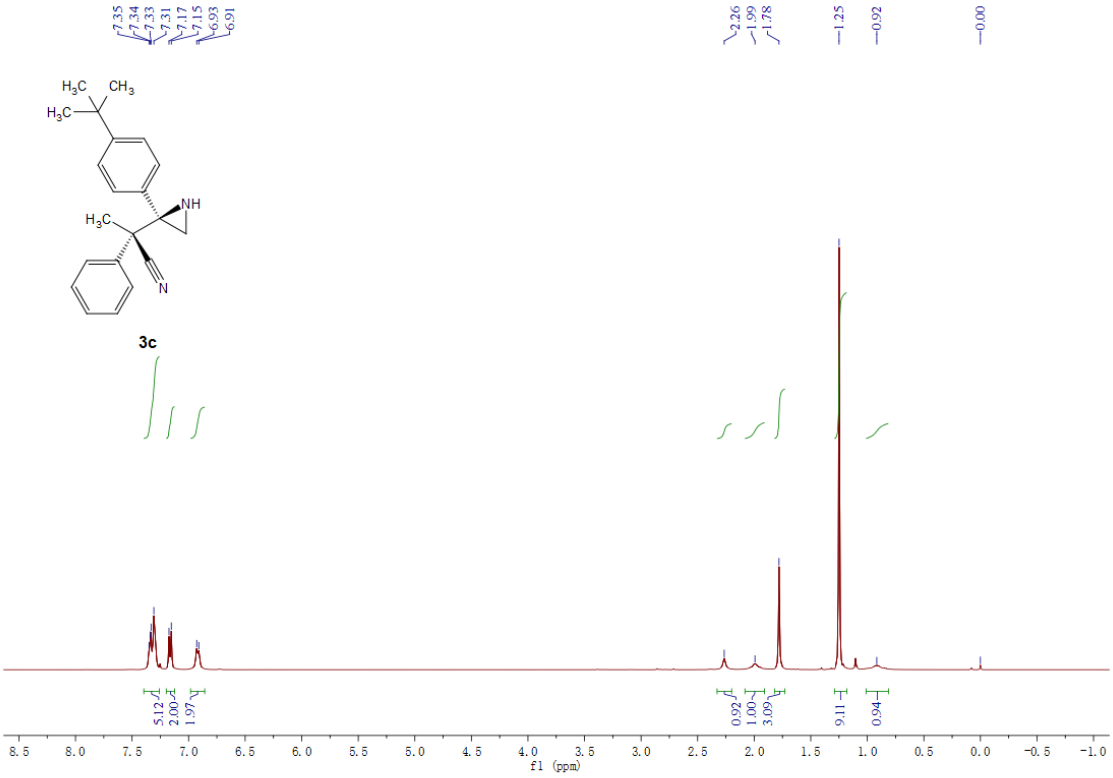

Supplementary Figure 64. ^{1}H NMR spectrum for compound **3c**

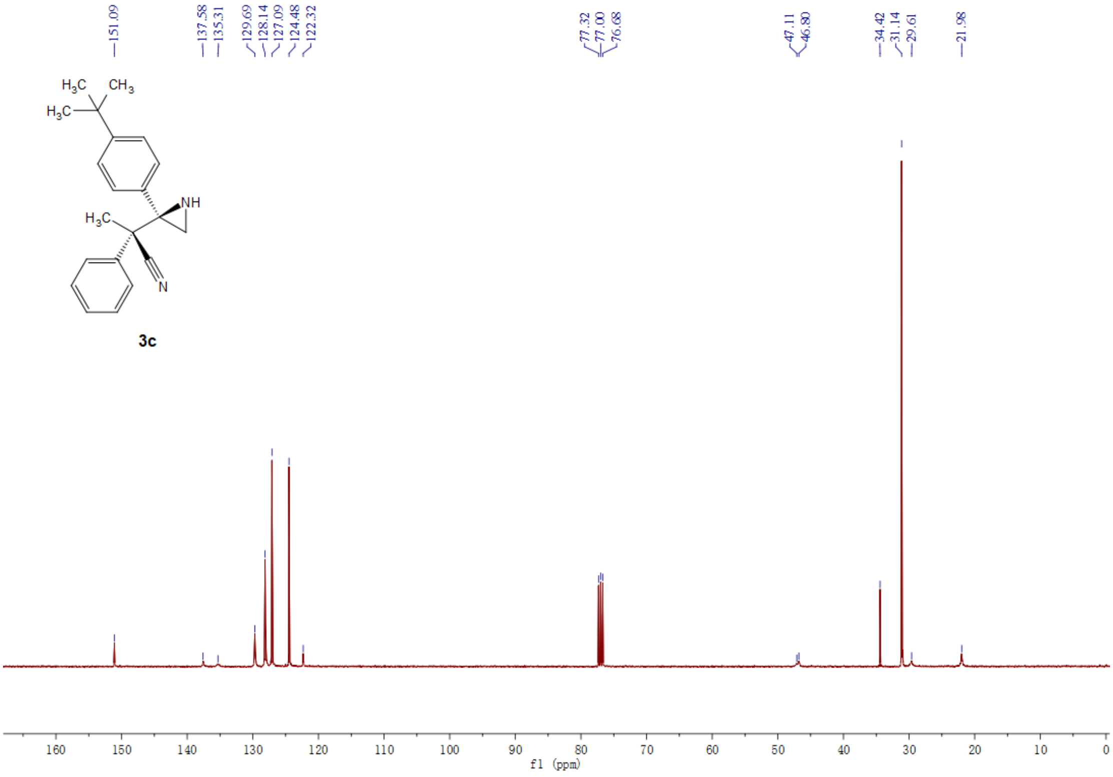

Supplementary Figure 65. ^{13}C NMR spectrum for compound **3c**

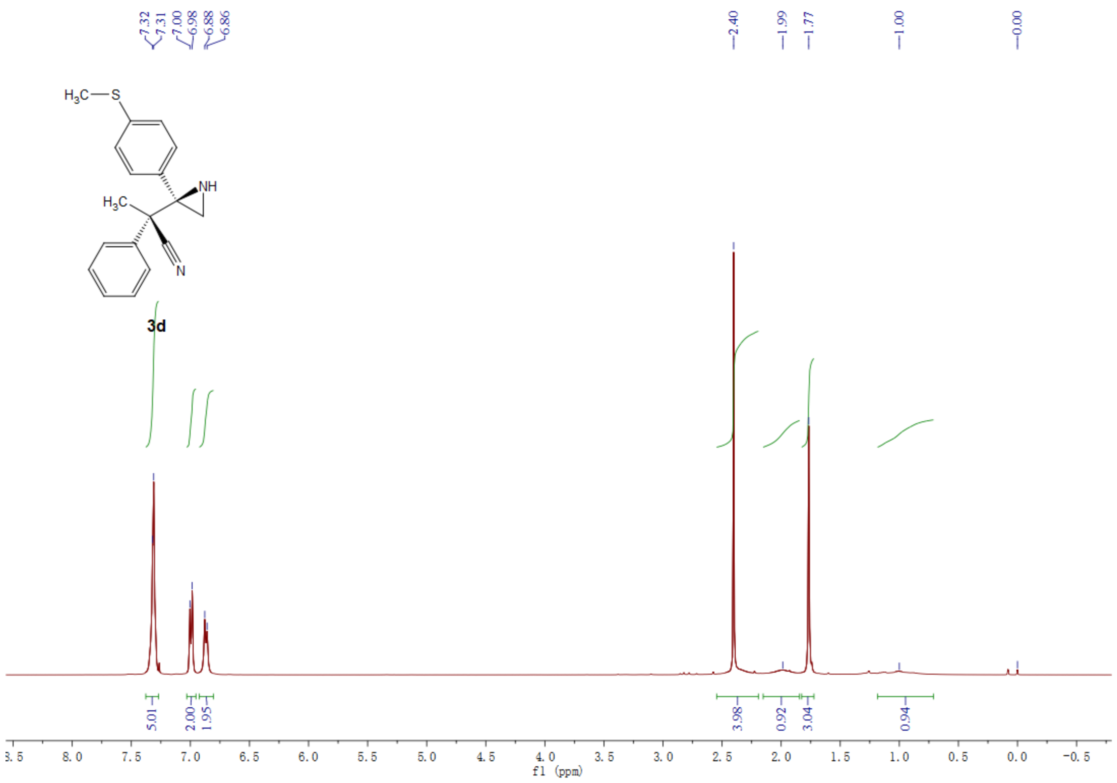

Supplementary Figure 66. ^{1}H NMR spectrum for compound **3d**

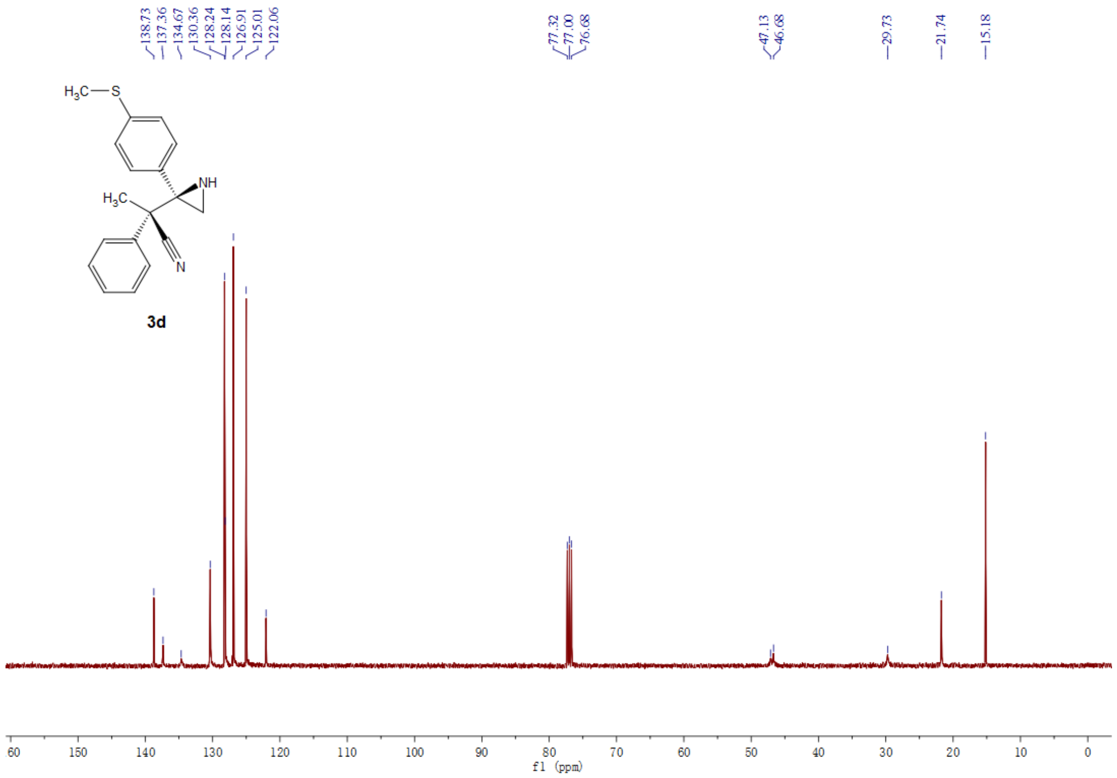

Supplementary Figure 67. ^{13}C NMR spectrum for compound **3d**

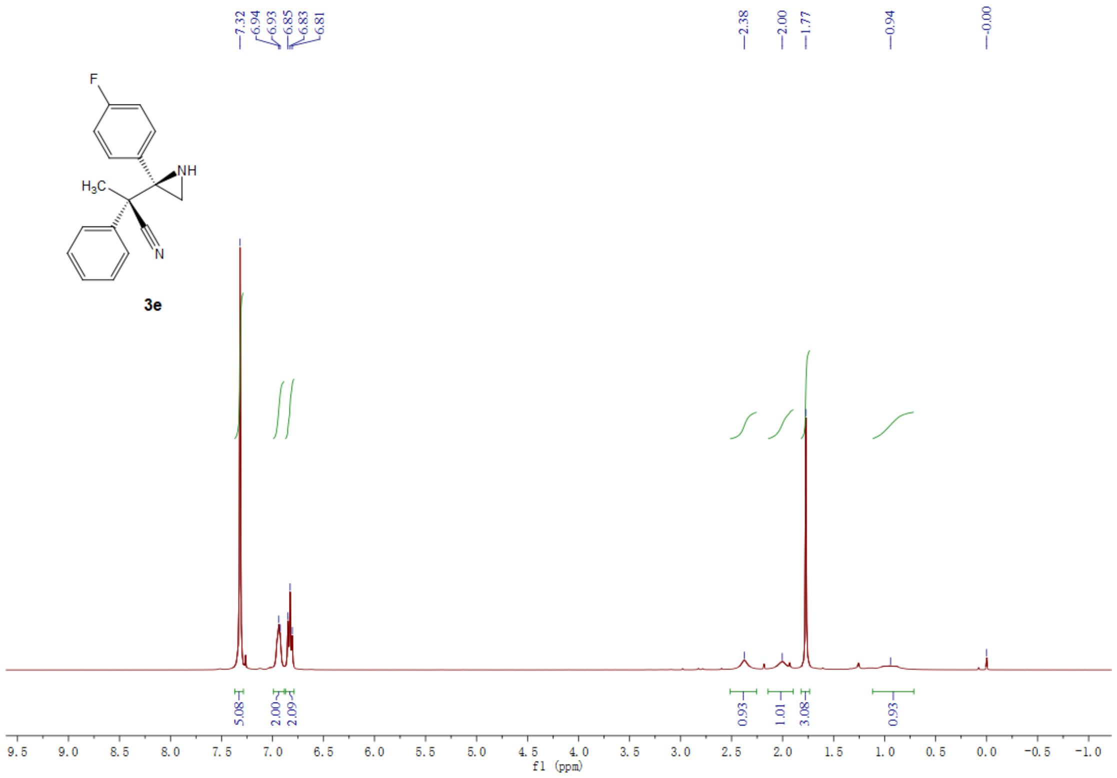

Supplementary Figure 68. ^{1}H NMR spectrum for compound **3e**

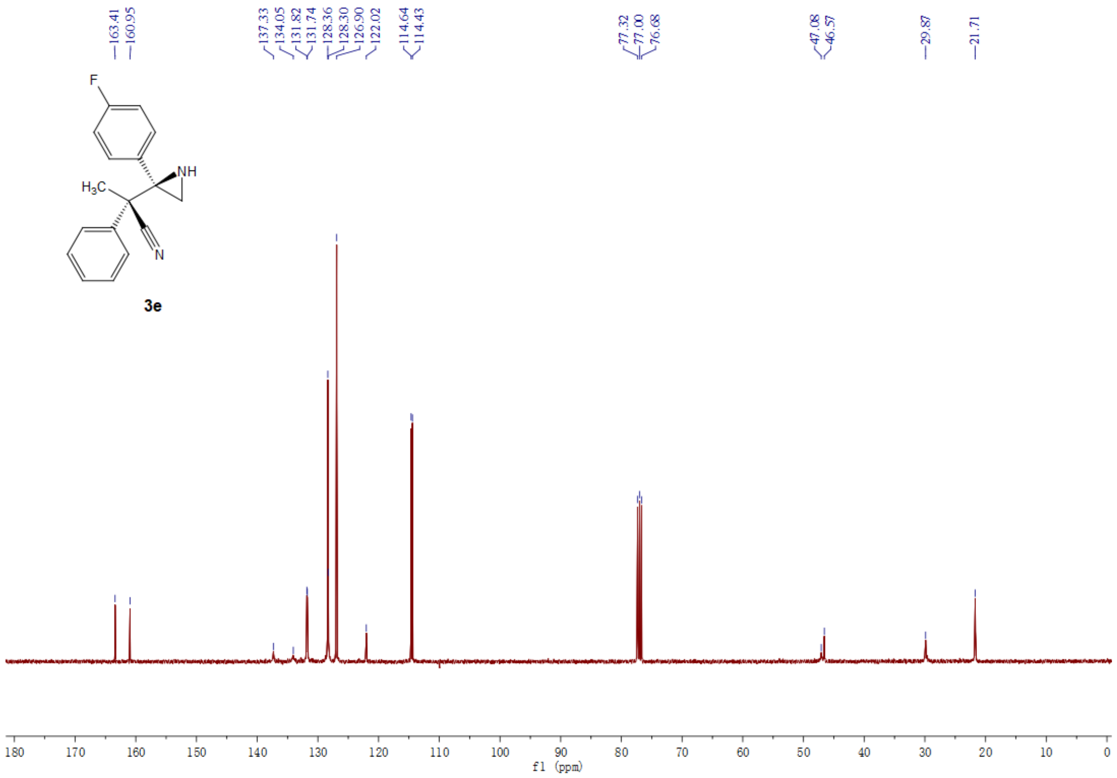

Supplementary Figure 69. ^{13}C NMR spectrum for compound **3e**

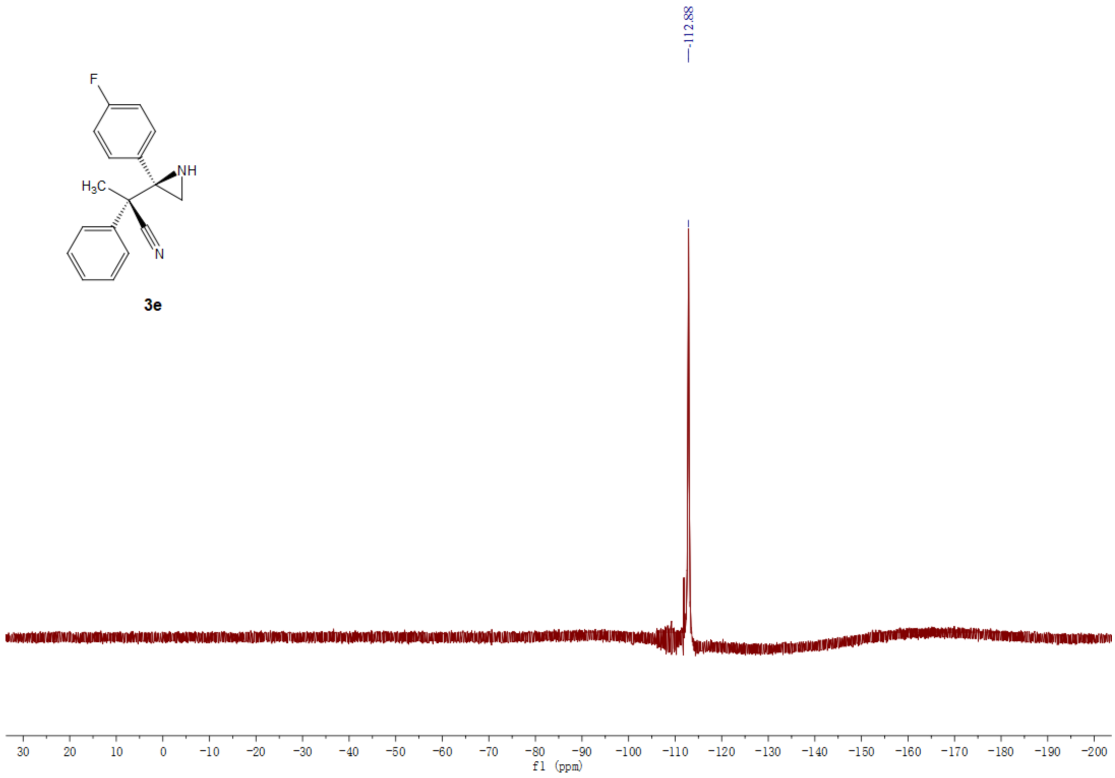

Supplementary Figure 70. ^{19}F NMR spectrum for compound **3e**

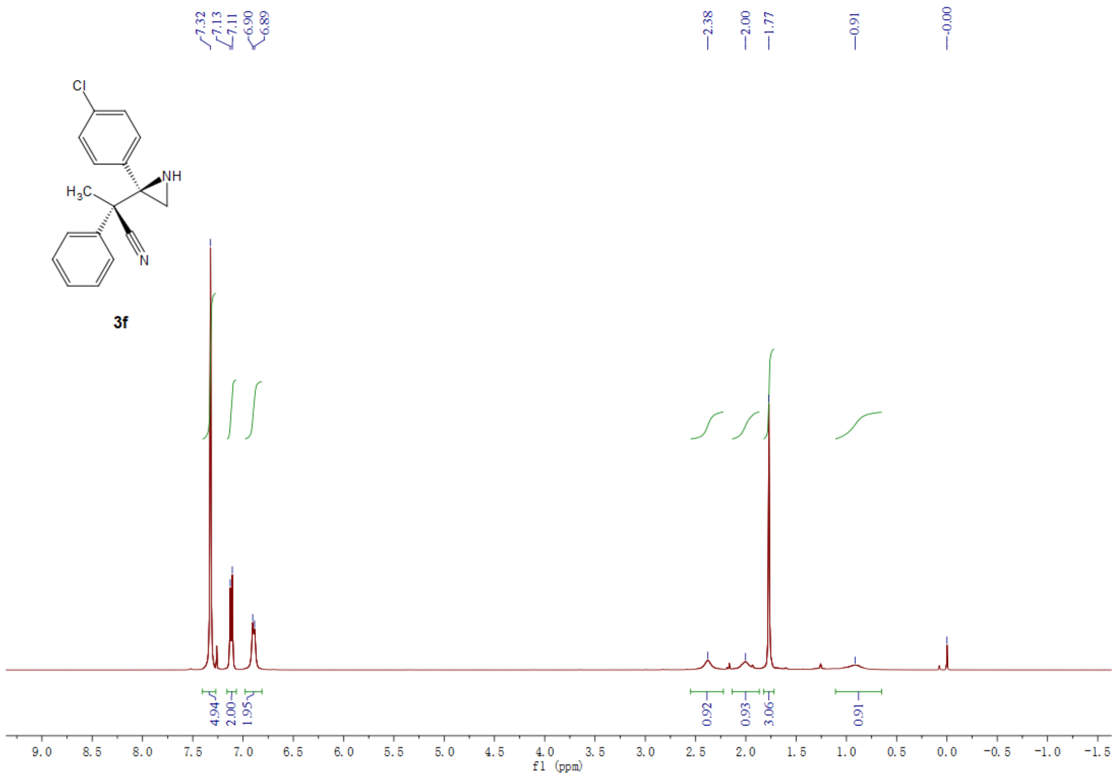

Supplementary Figure 71. ^{1}H NMR spectrum for compound **3f**

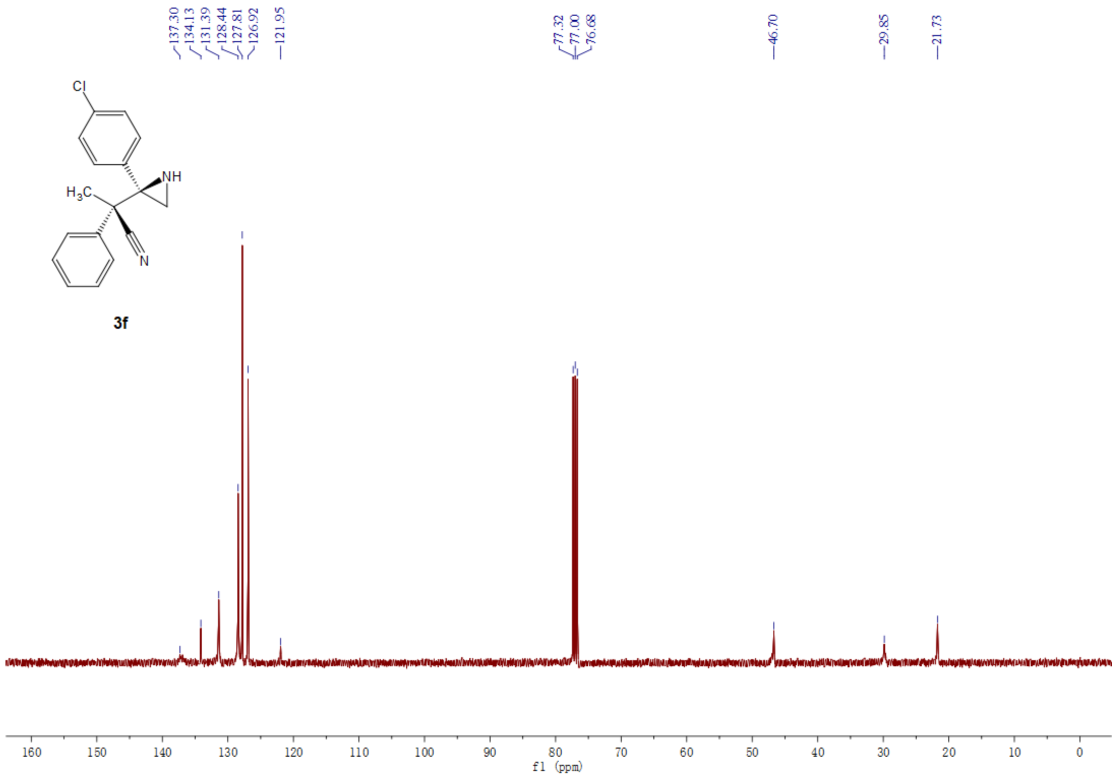

Supplementary Figure 72. ^{13}C NMR spectrum for compound **3f**

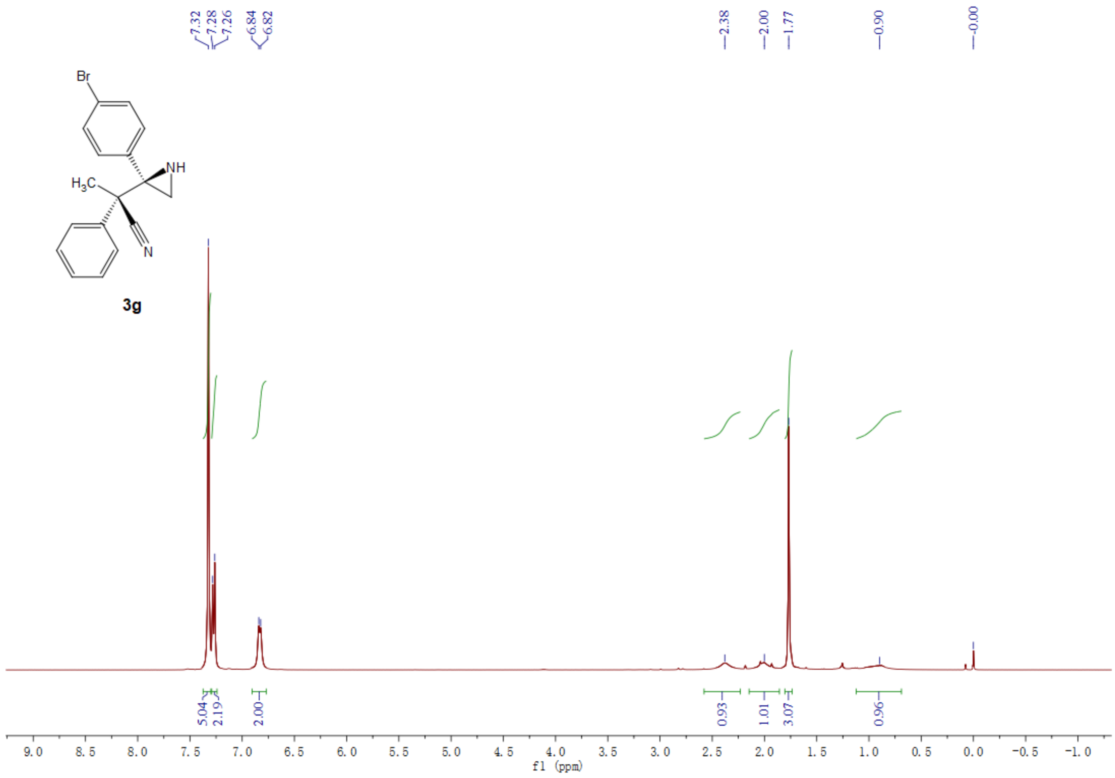

Supplementary Figure 73. ^{1}H NMR spectrum for compound **3g**

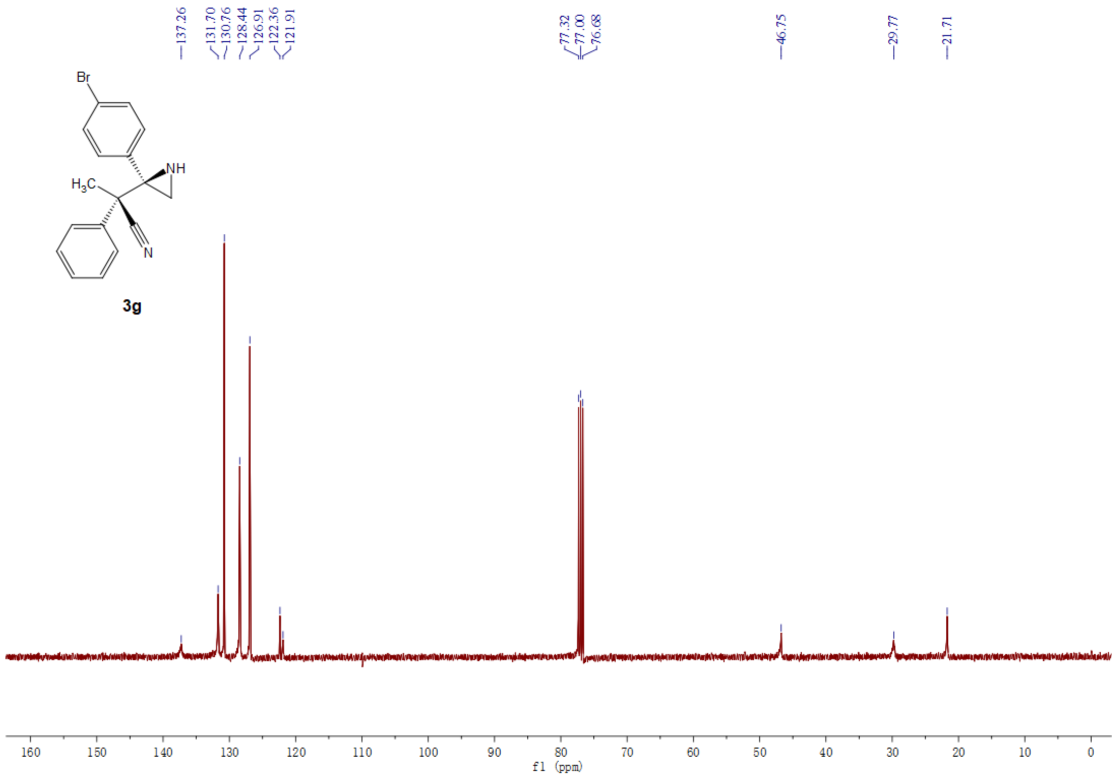

Supplementary Figure 74. ^{13}C NMR spectrum for compound **3g**

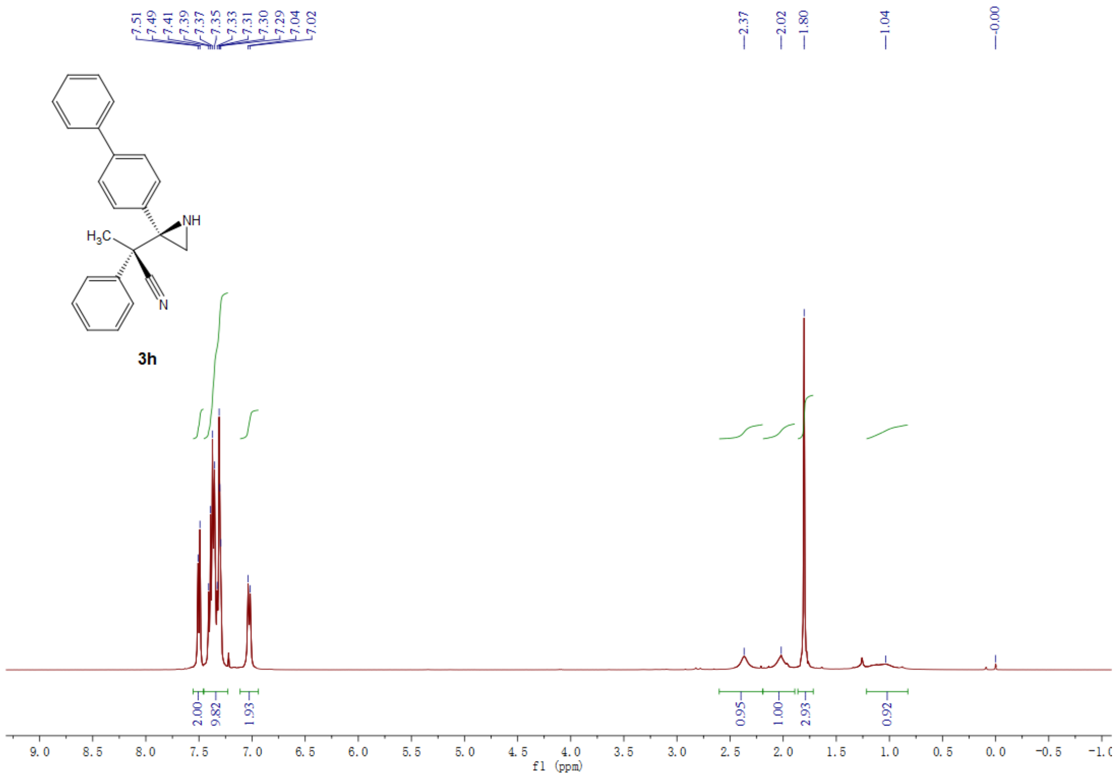

Supplementary Figure 75. ^1H NMR spectrum for compound **3h**

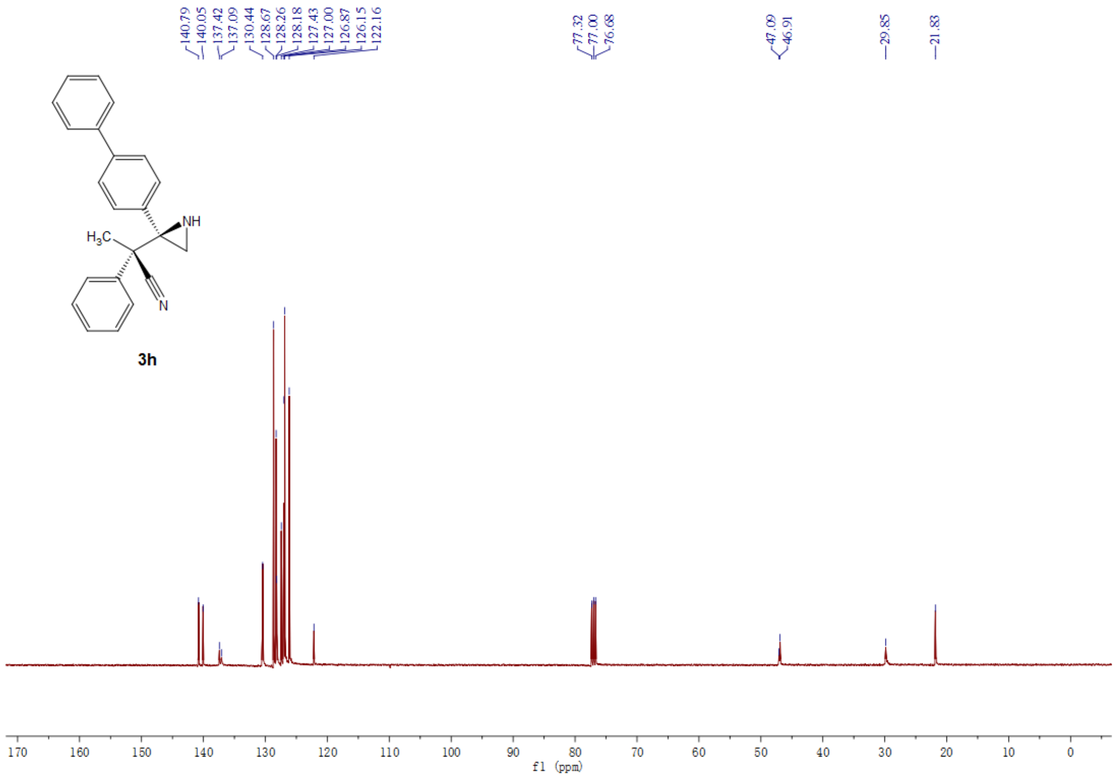

Supplementary Figure 76. ^{13}C NMR spectrum for compound **3h**

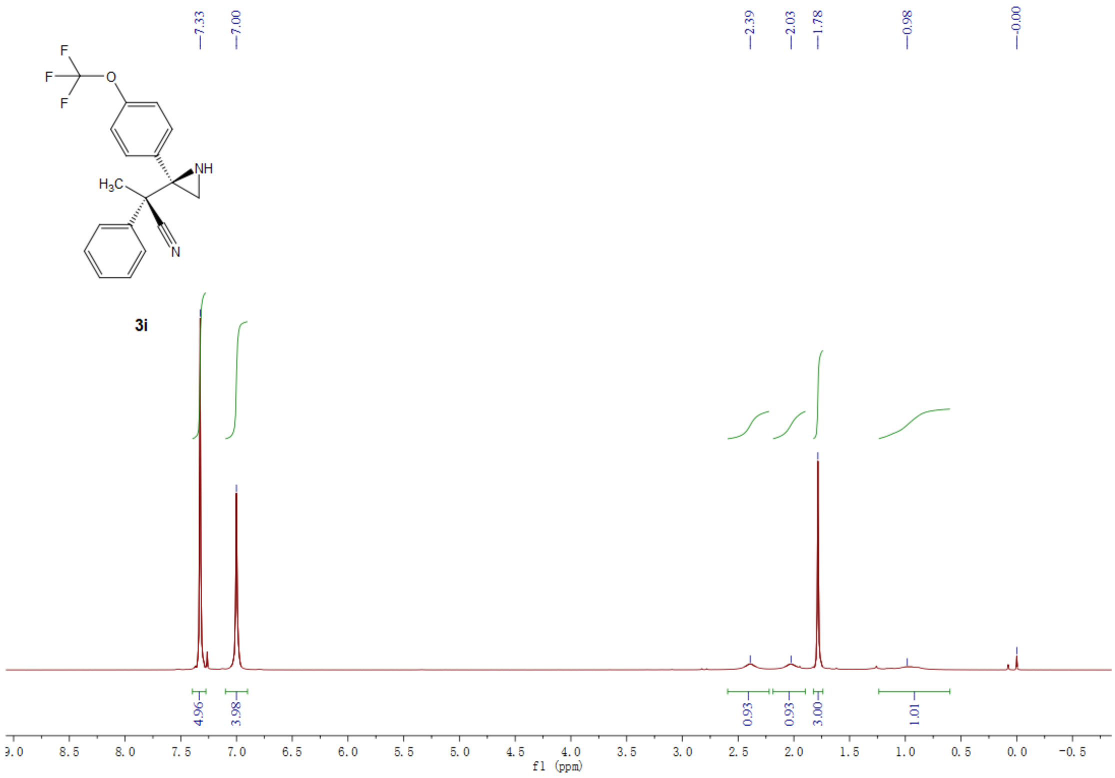

Supplementary Figure 77. ¹H NMR spectrum for compound **3i**

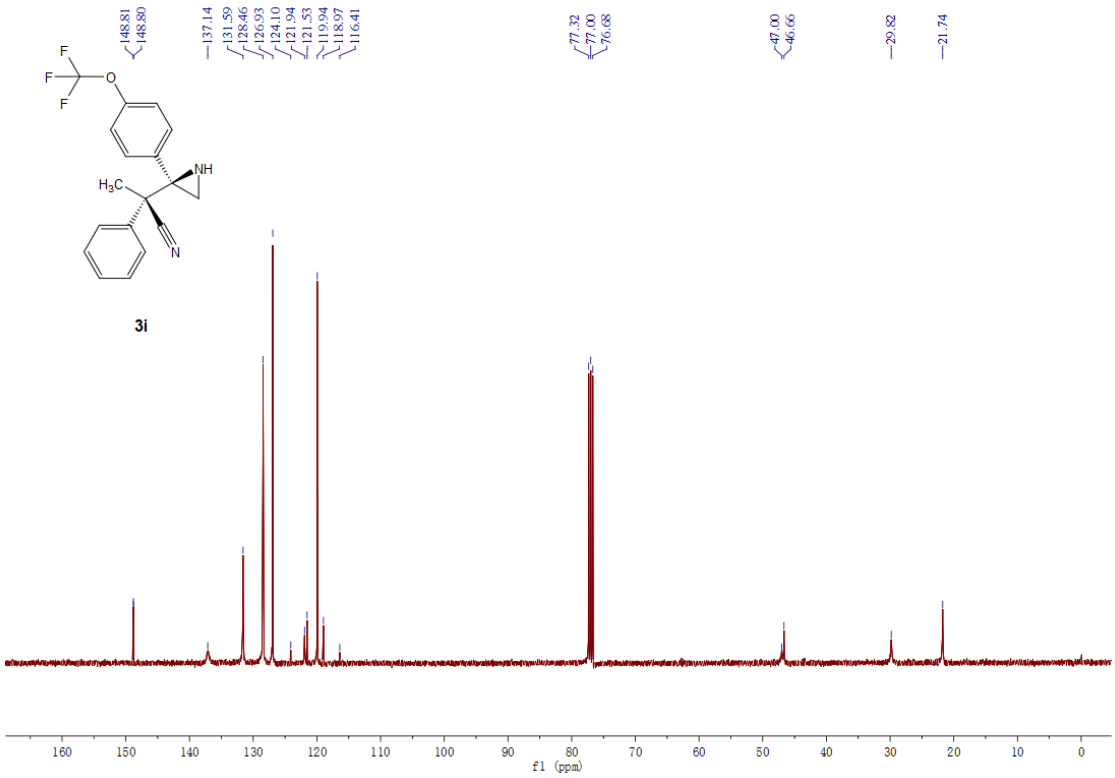

Supplementary Figure 78. ¹³C NMR spectrum for compound **3i**

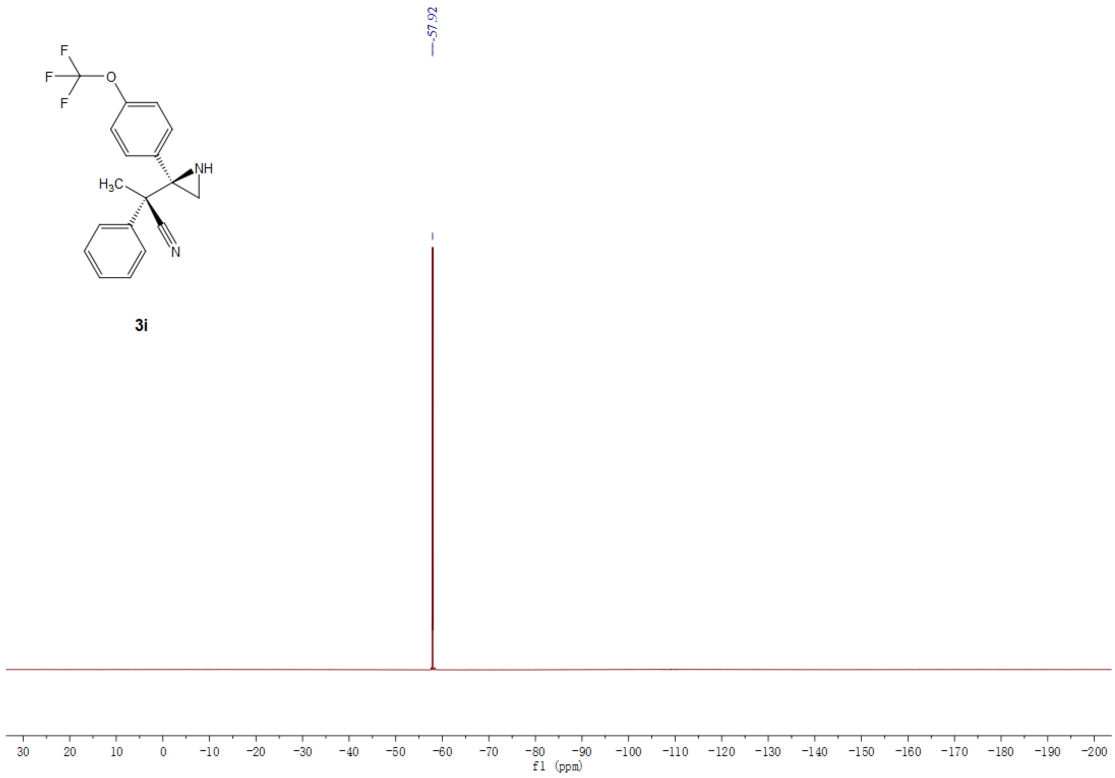

Supplementary Figure 79. ^{19}F NMR spectrum for compound **3i**

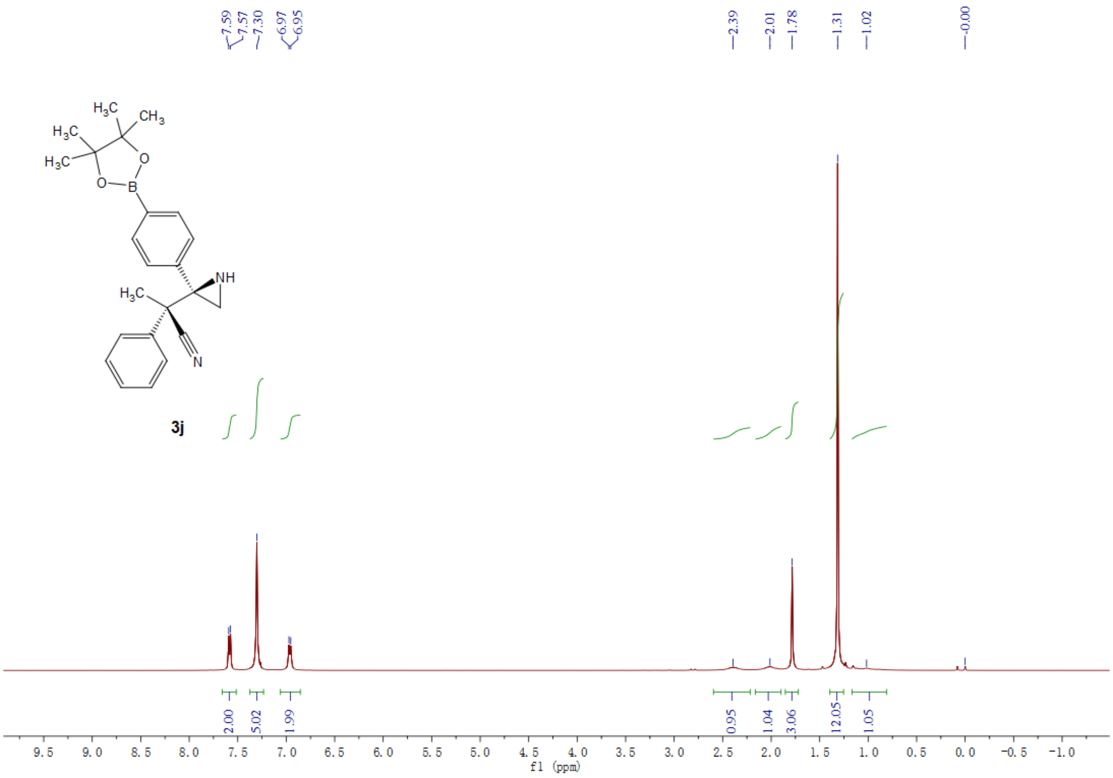

Supplementary Figure 80. ^{1}H NMR spectrum for compound **3j**

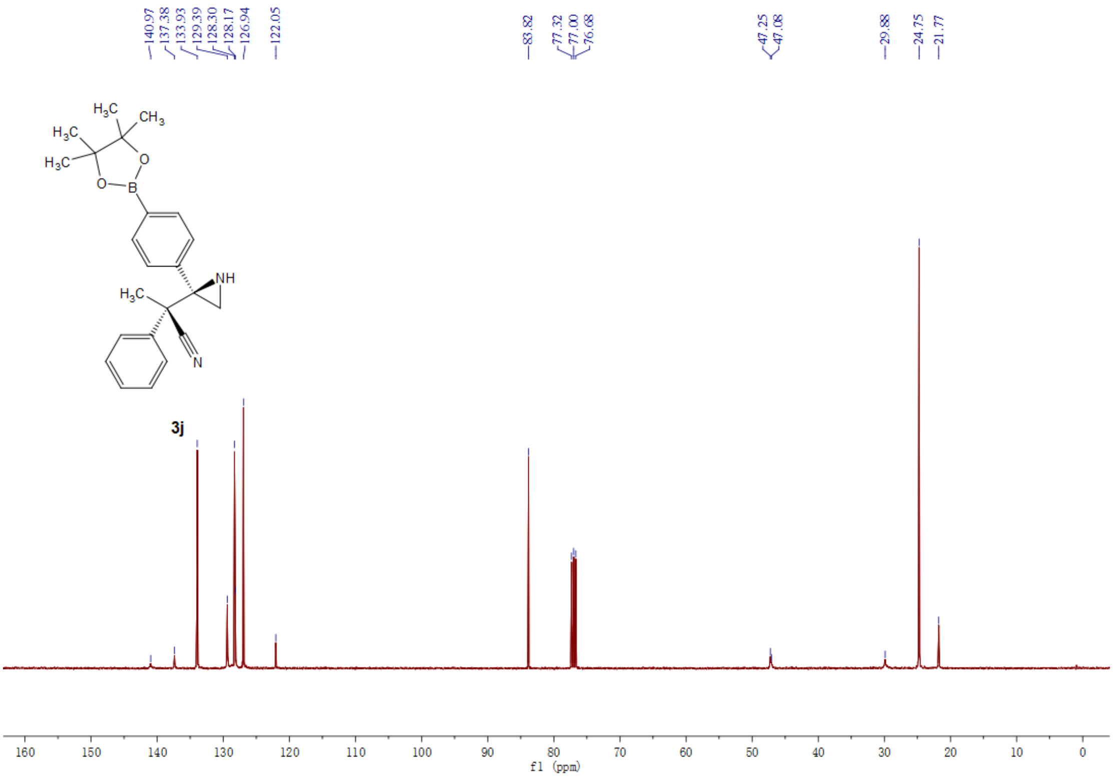

Supplementary Figure 81. ^{13}C NMR spectrum for compound **3j**

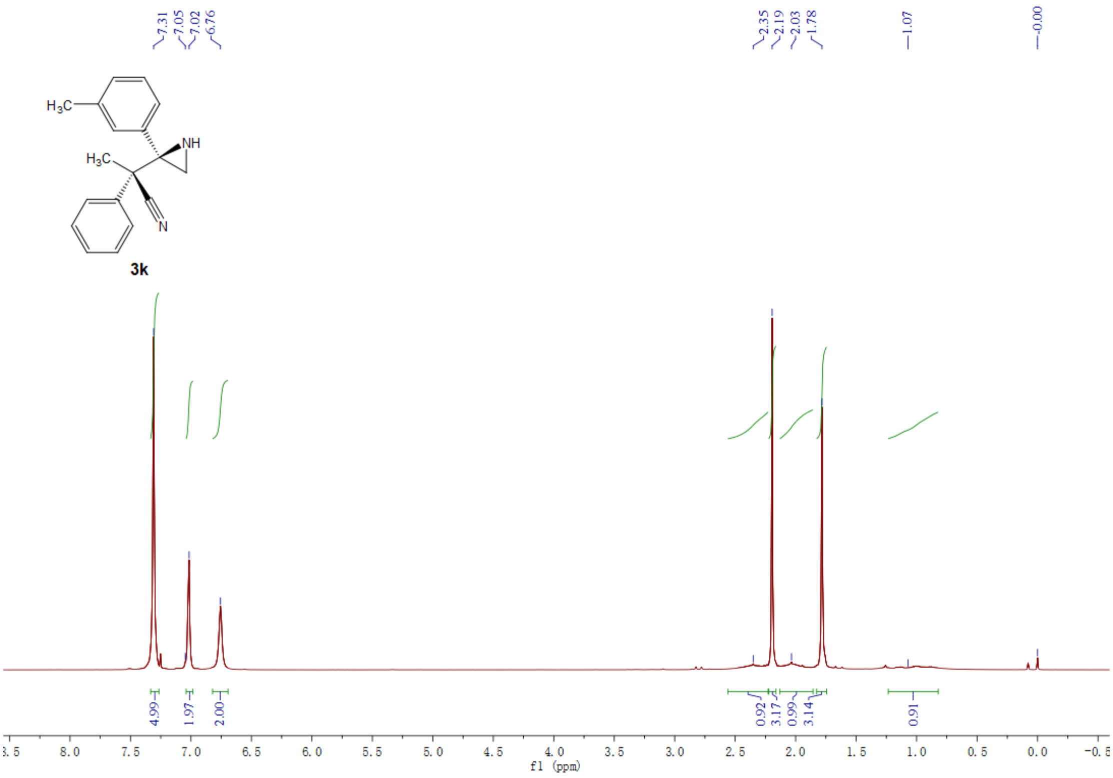

Supplementary Figure 82. ^{1}H NMR spectrum for compound **3k**

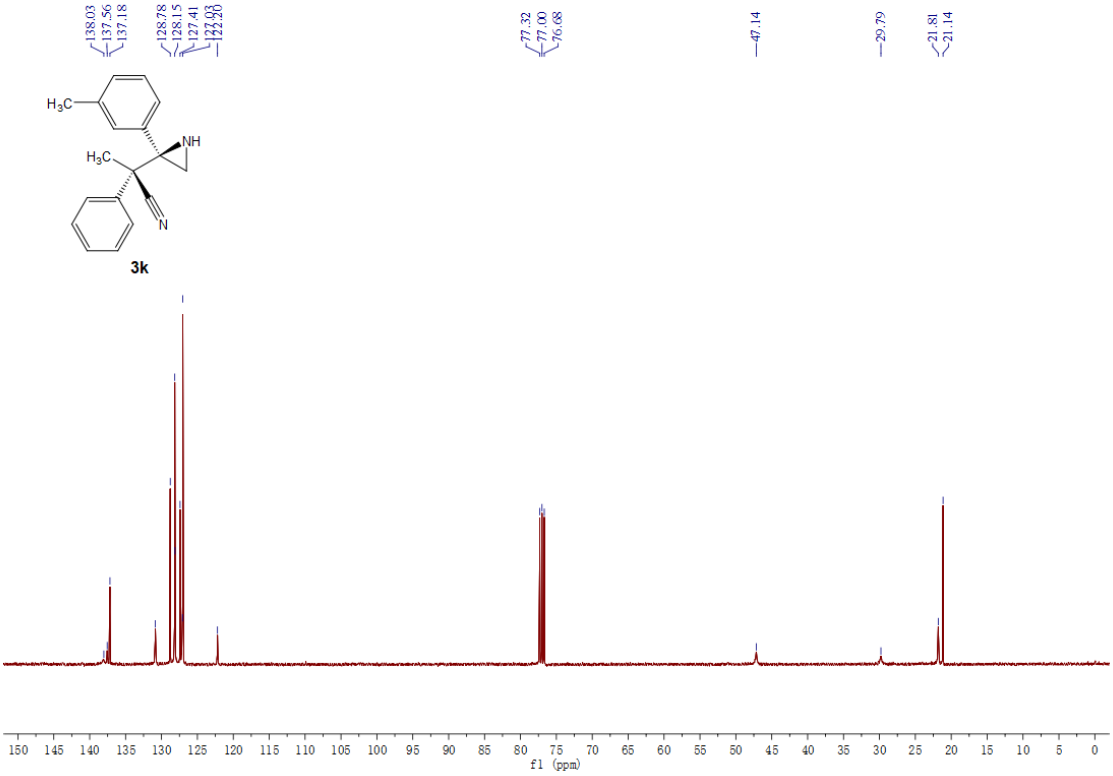

Supplementary Figure 83. ^{13}C NMR spectrum for compound **3k**

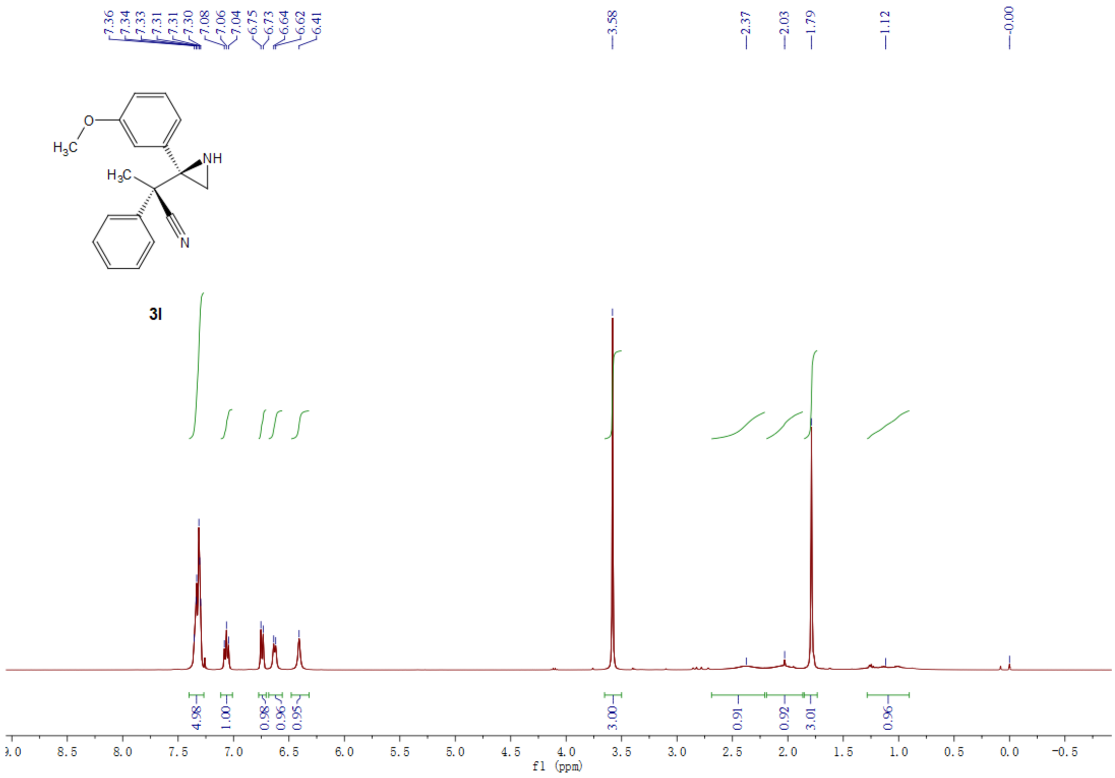

Supplementary Figure 84. ^{1}H NMR spectrum for compound **3l**

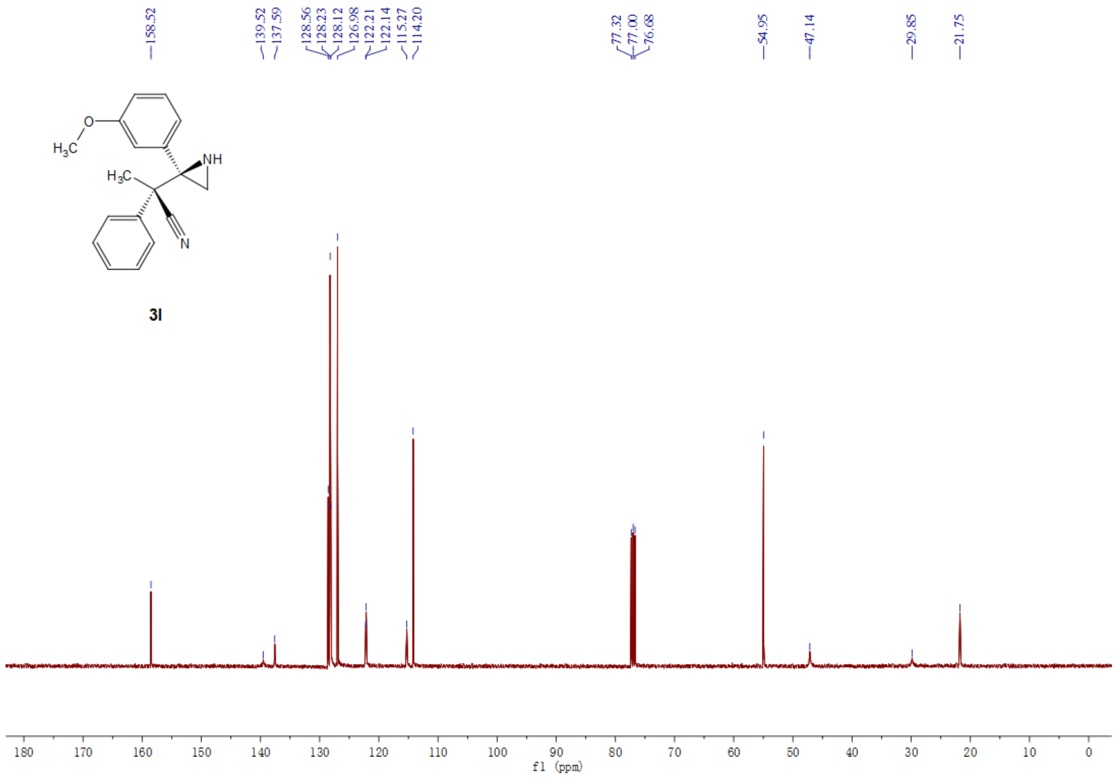

Supplementary Figure 85. ^{13}C NMR spectrum for compound **3l**

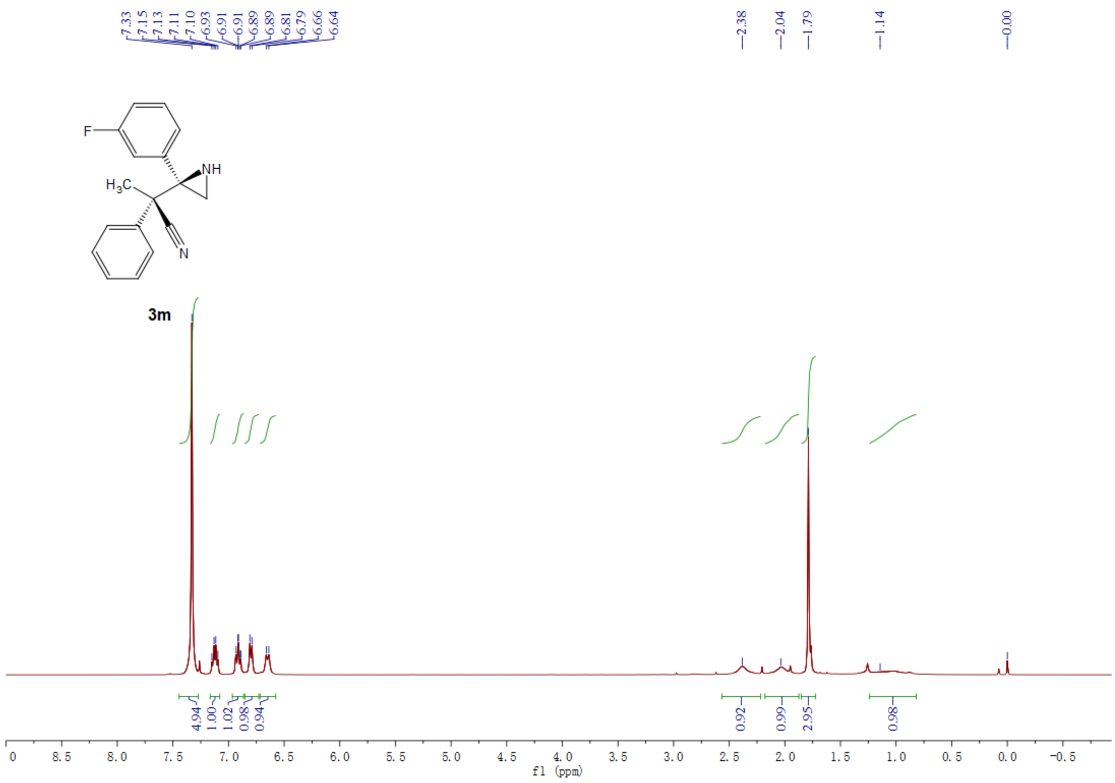

Supplementary Figure 86. ^{1}H NMR spectrum for compound **3m**

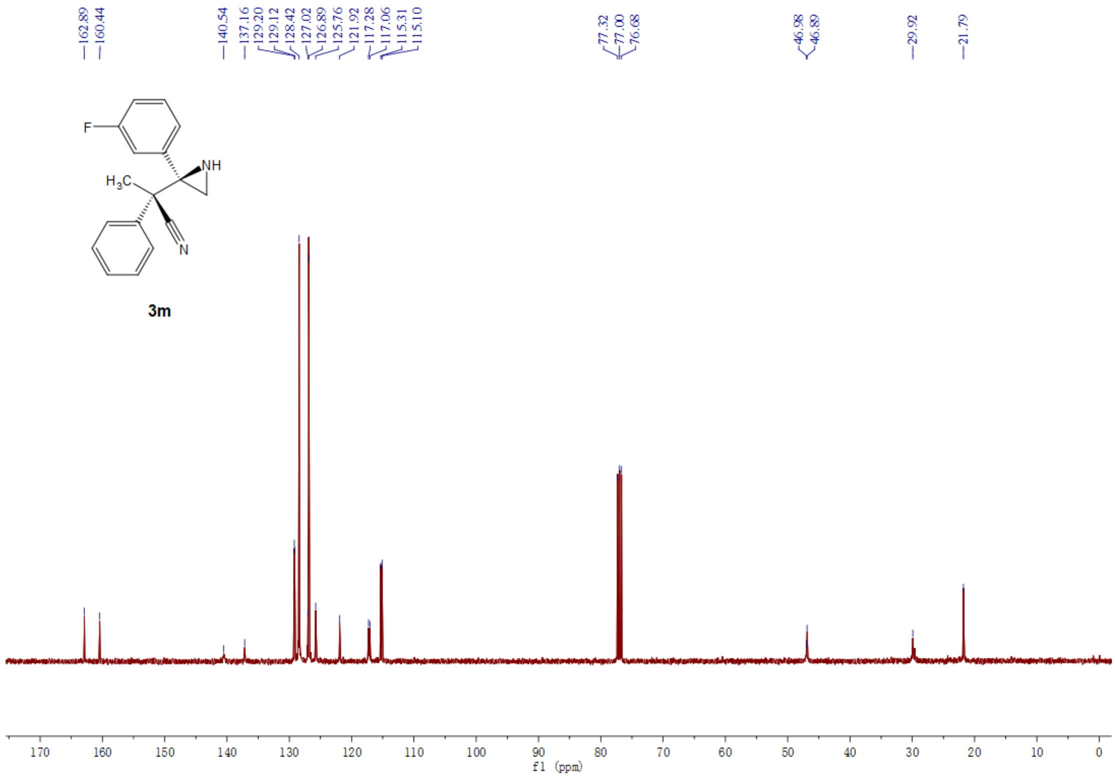

Supplementary Figure 87. ^{13}C NMR spectrum for compound **3m**

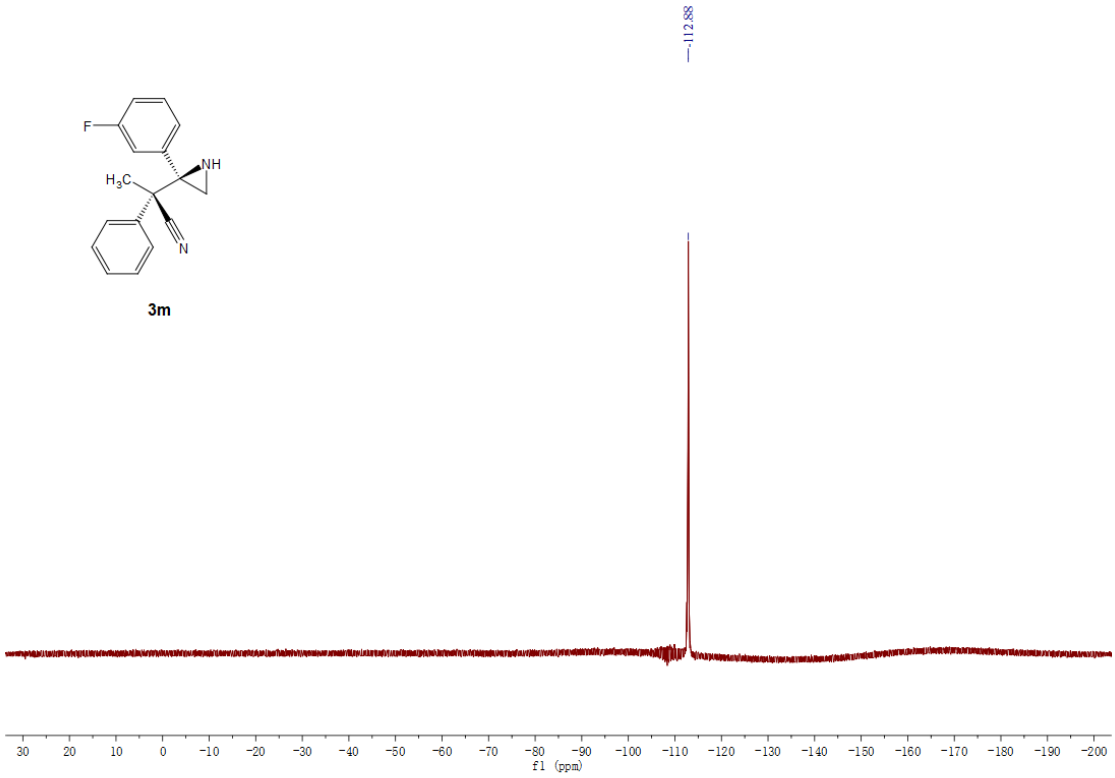

Supplementary Figure 88. ^{19}F NMR spectrum for compound **3m**

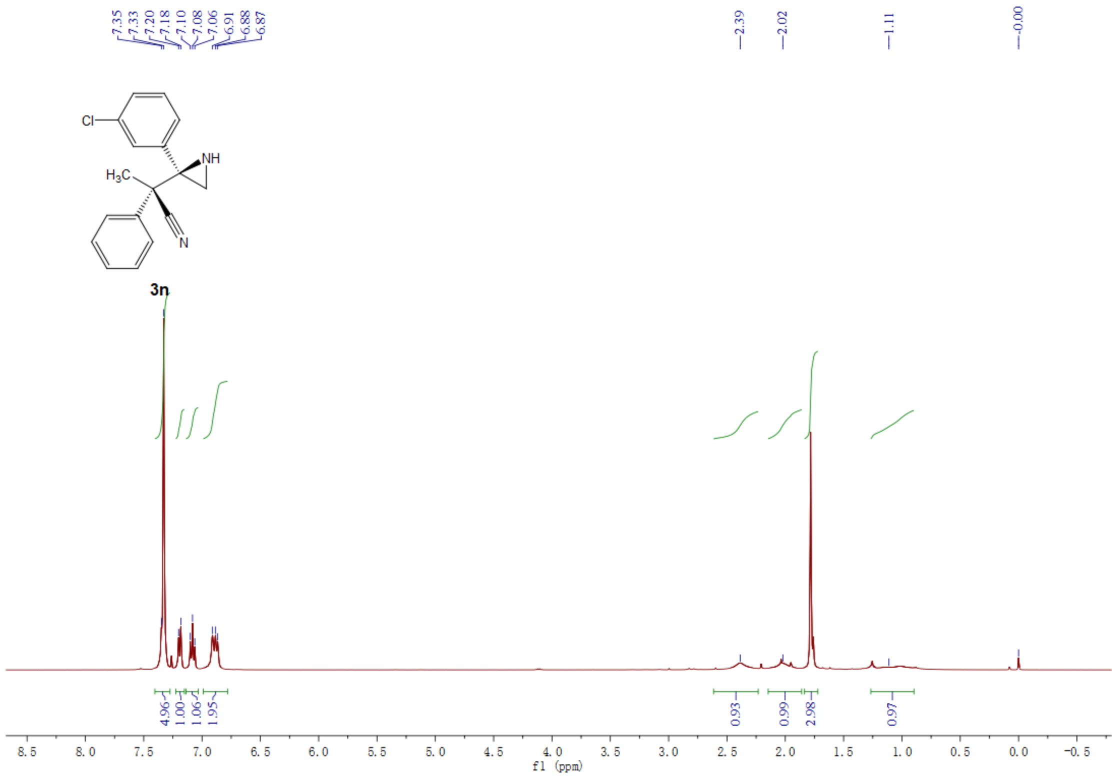

Supplementary Figure 89. ^{1}H NMR spectrum for compound **3n**

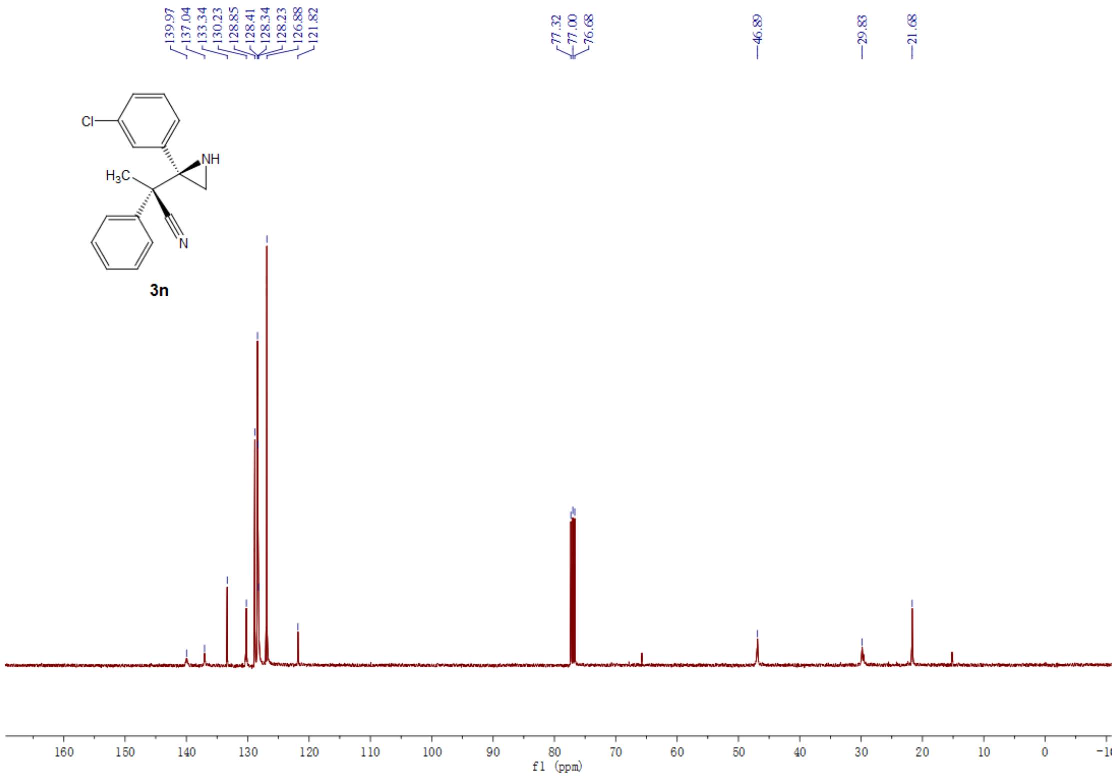

Supplementary Figure 90. ^{13}C NMR spectrum for compound **3n**

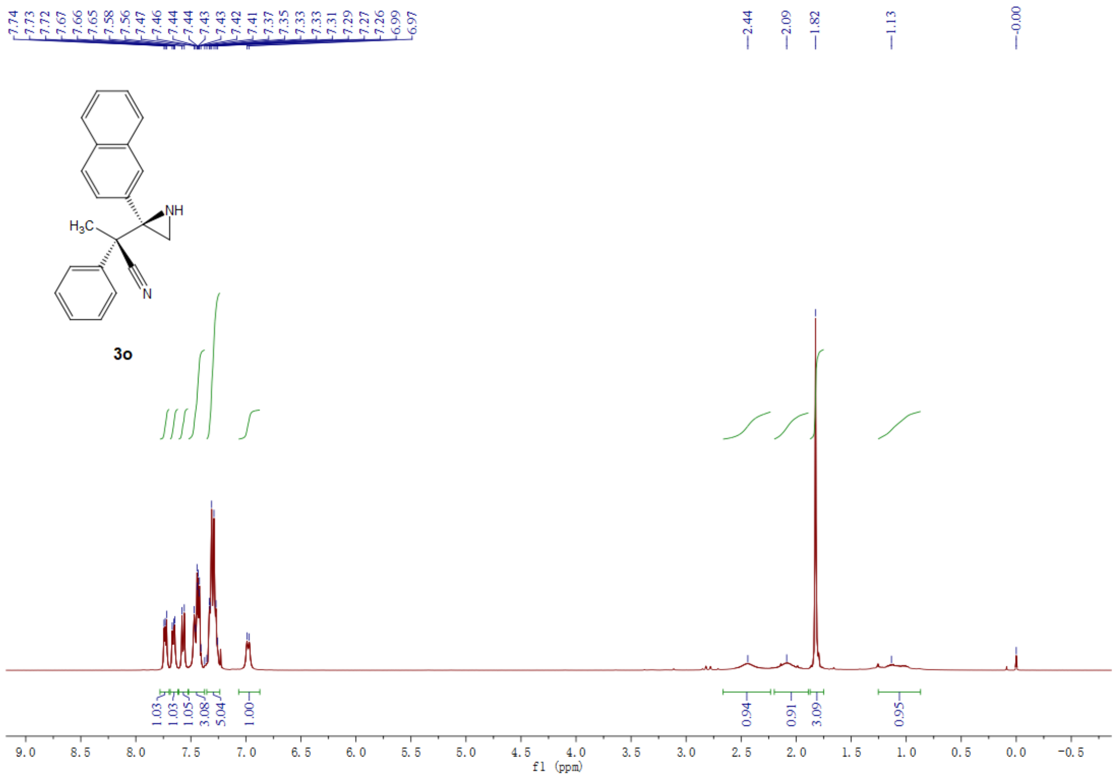

Supplementary Figure 91. ^{1}H NMR spectrum for compound **3o**

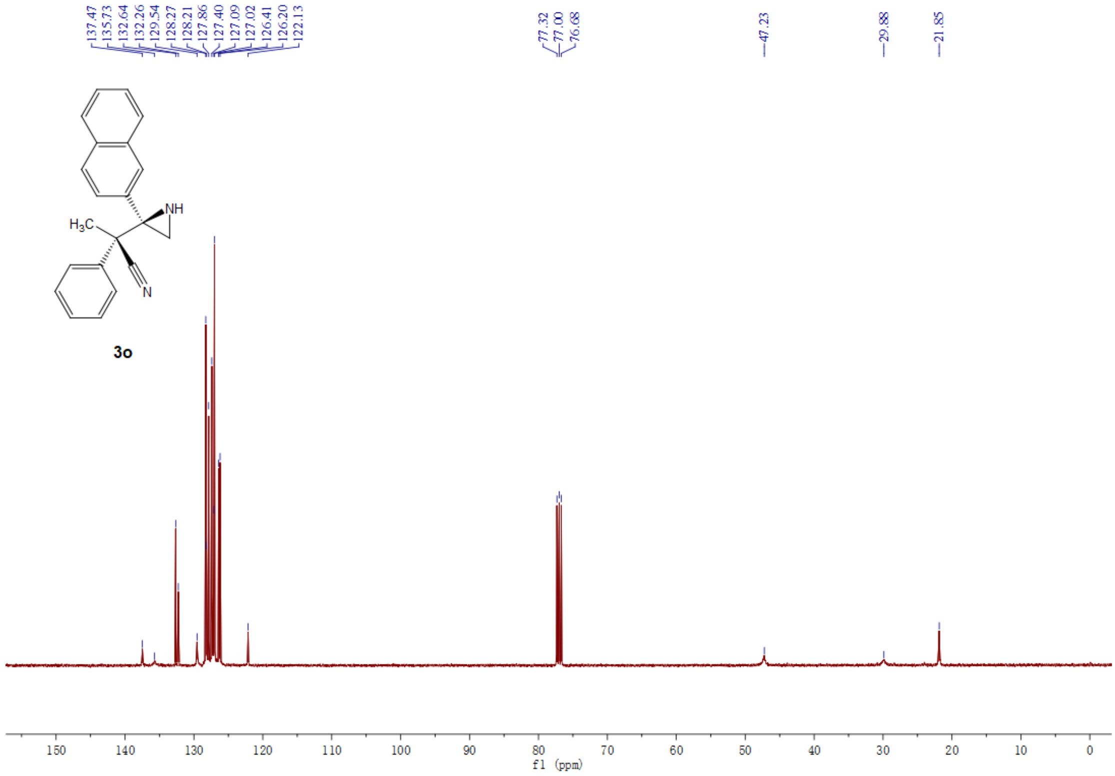

Supplementary Figure 92. ^{13}C NMR spectrum for compound **3o**

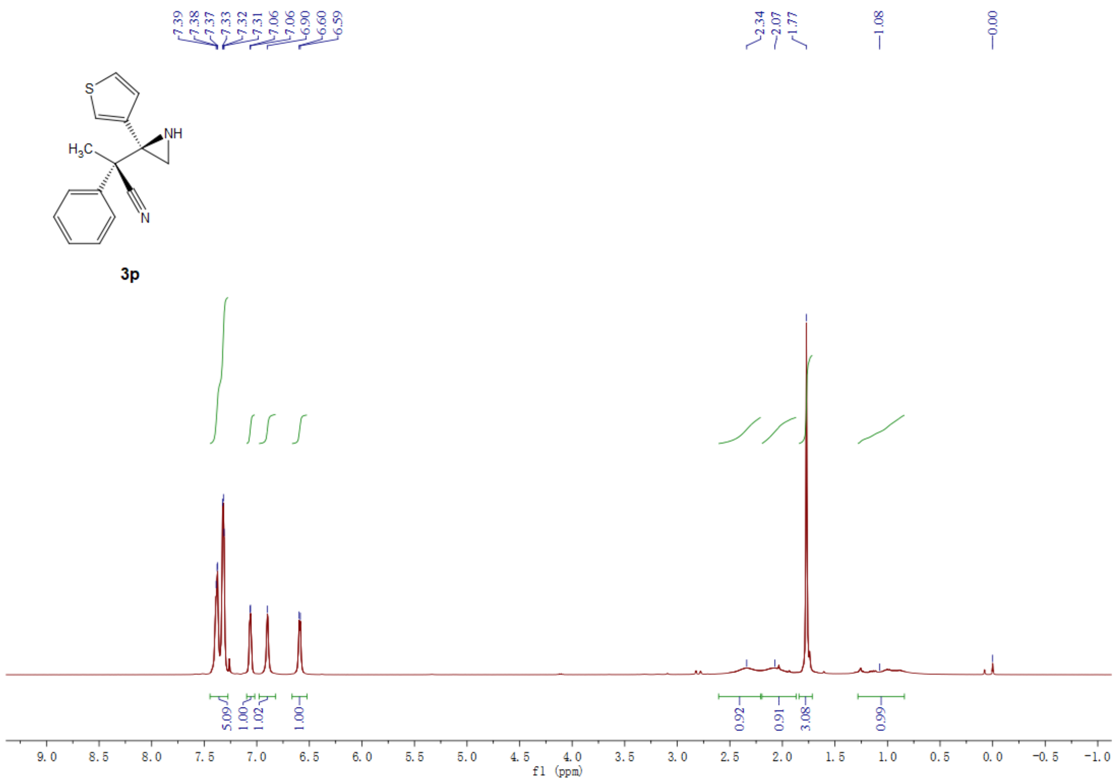

Supplementary Figure 93. ^{1}H NMR spectrum for compound **3p**

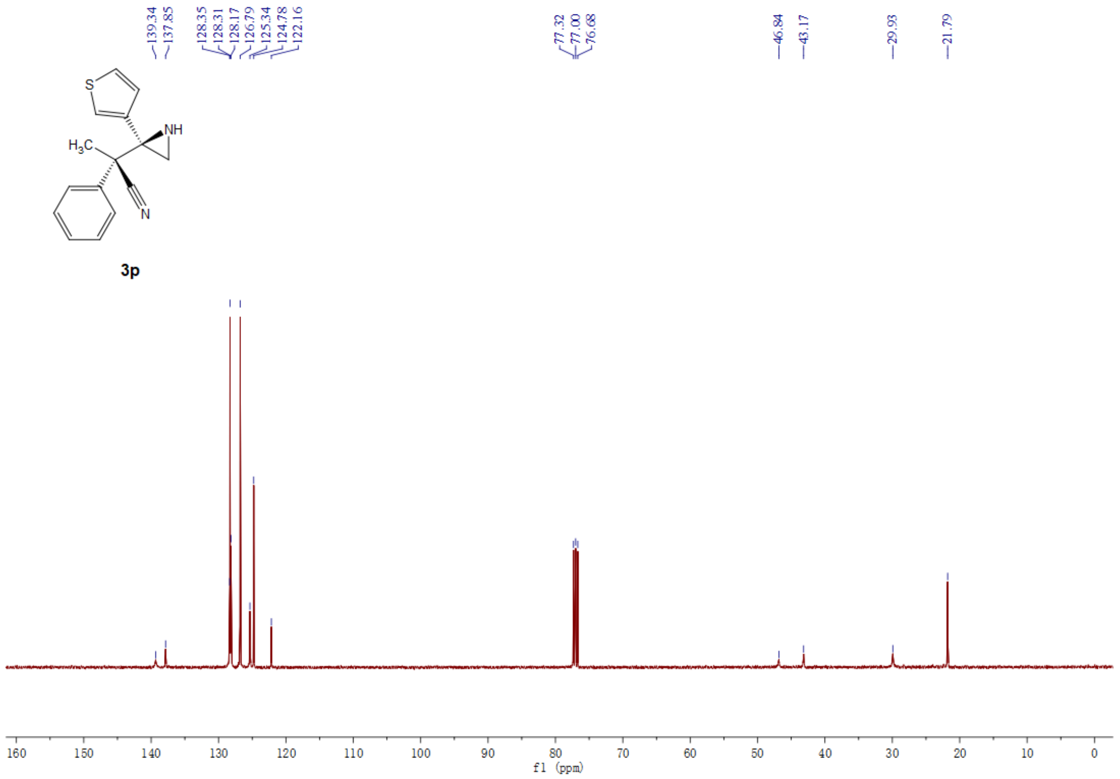

Supplementary Figure 94. ^{13}C NMR spectrum for compound **3p**

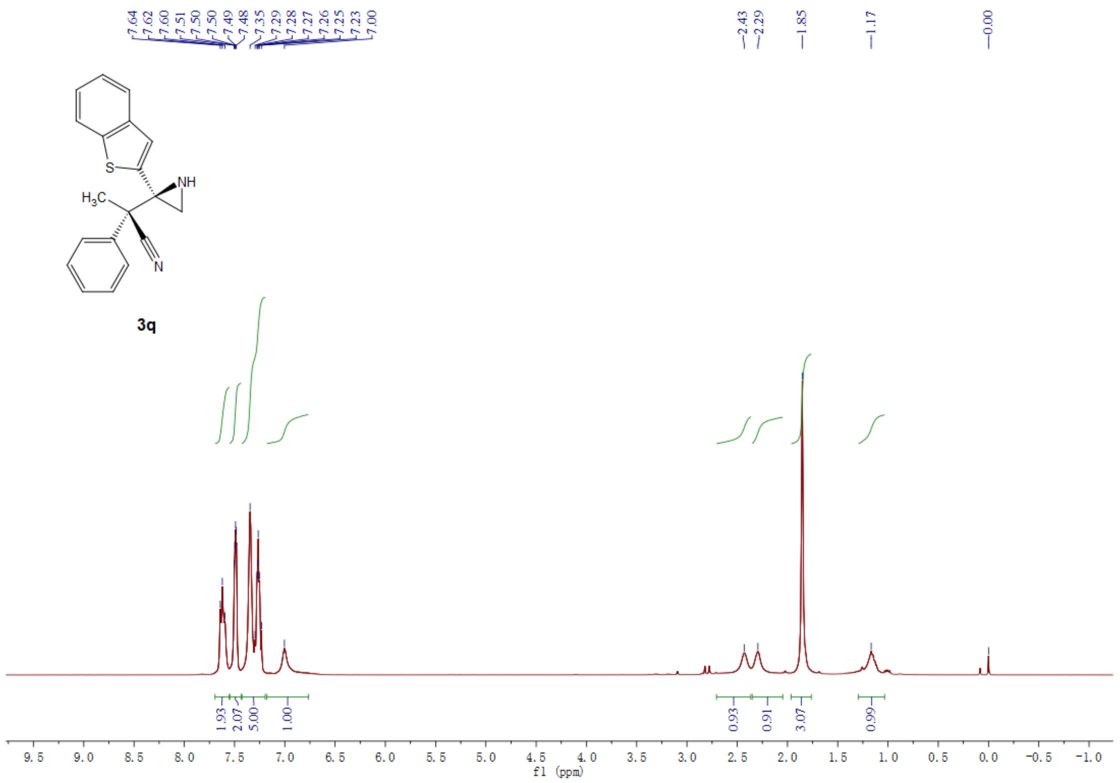

Supplementary Figure 95. ^{1}H NMR spectrum for compound **3q**

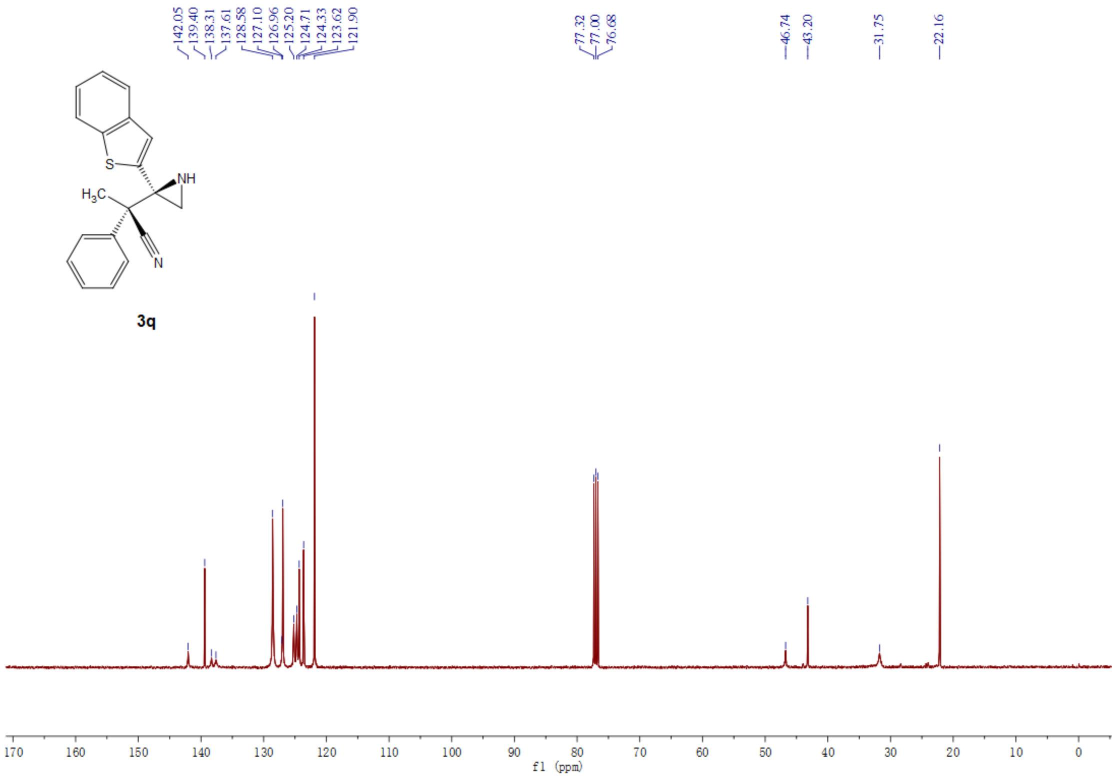

Supplementary Figure 96. ^{13}C NMR spectrum for compound **3q**

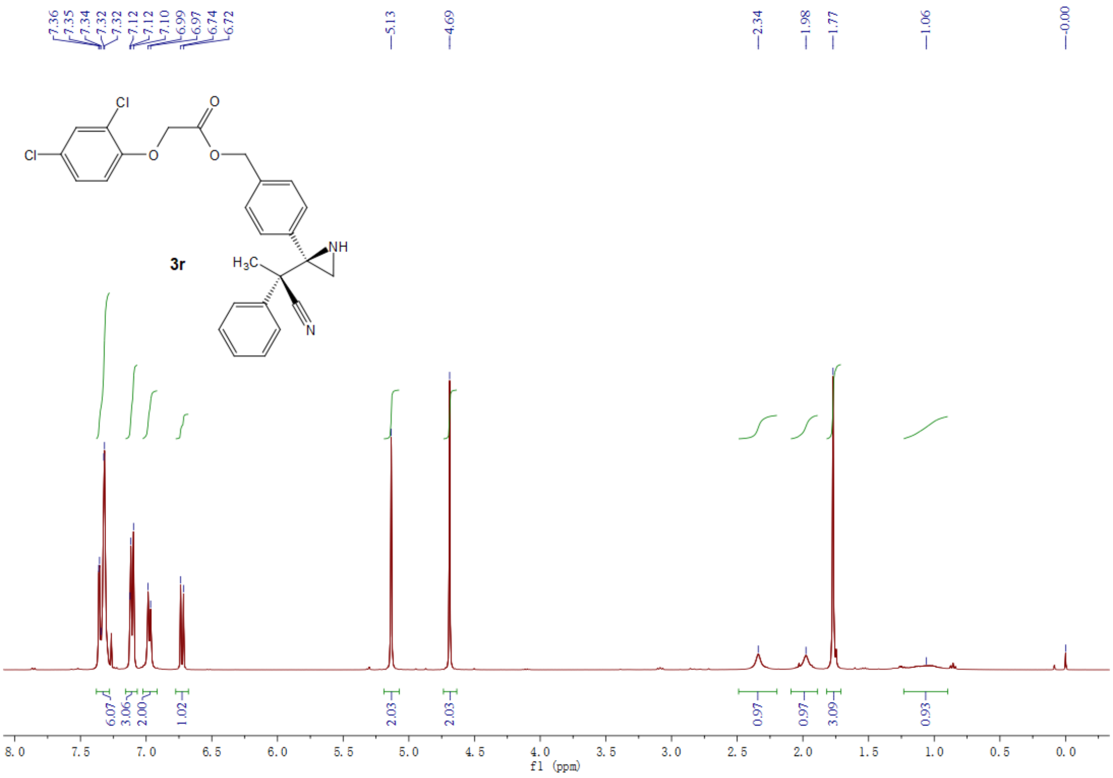

Supplementary Figure 97. ^{1}H NMR spectrum for compound **3r**

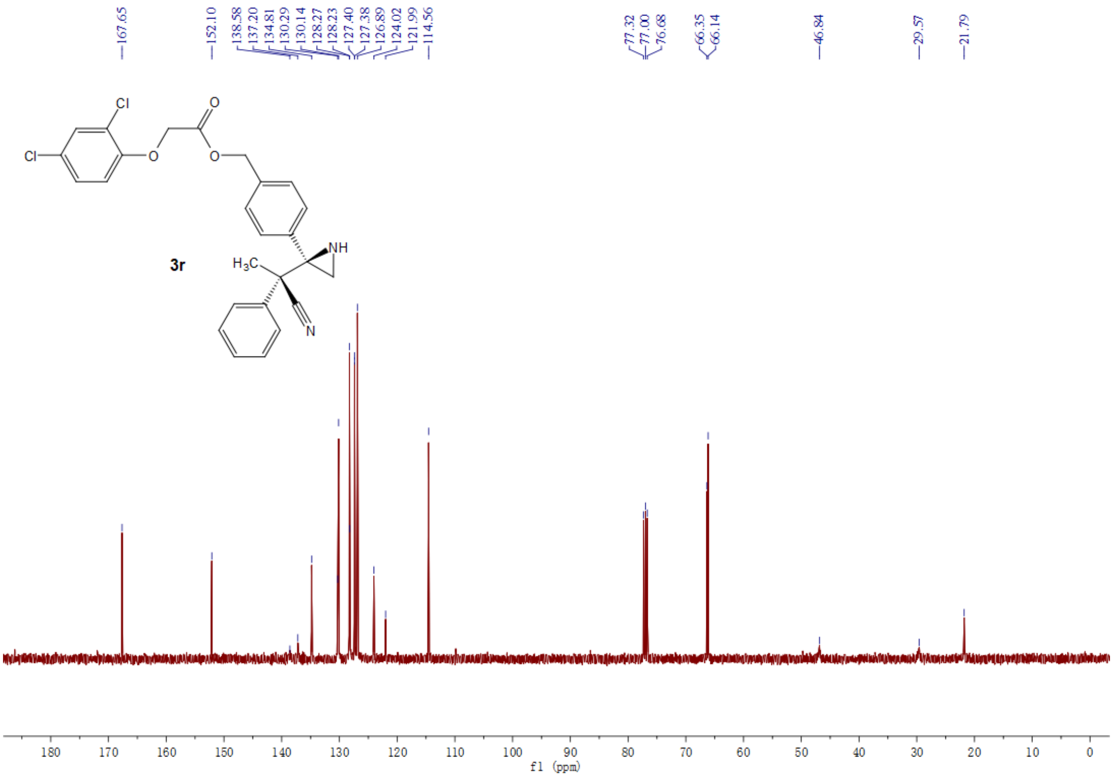

Supplementary Figure 98. ^{13}C NMR spectrum for compound **3r**

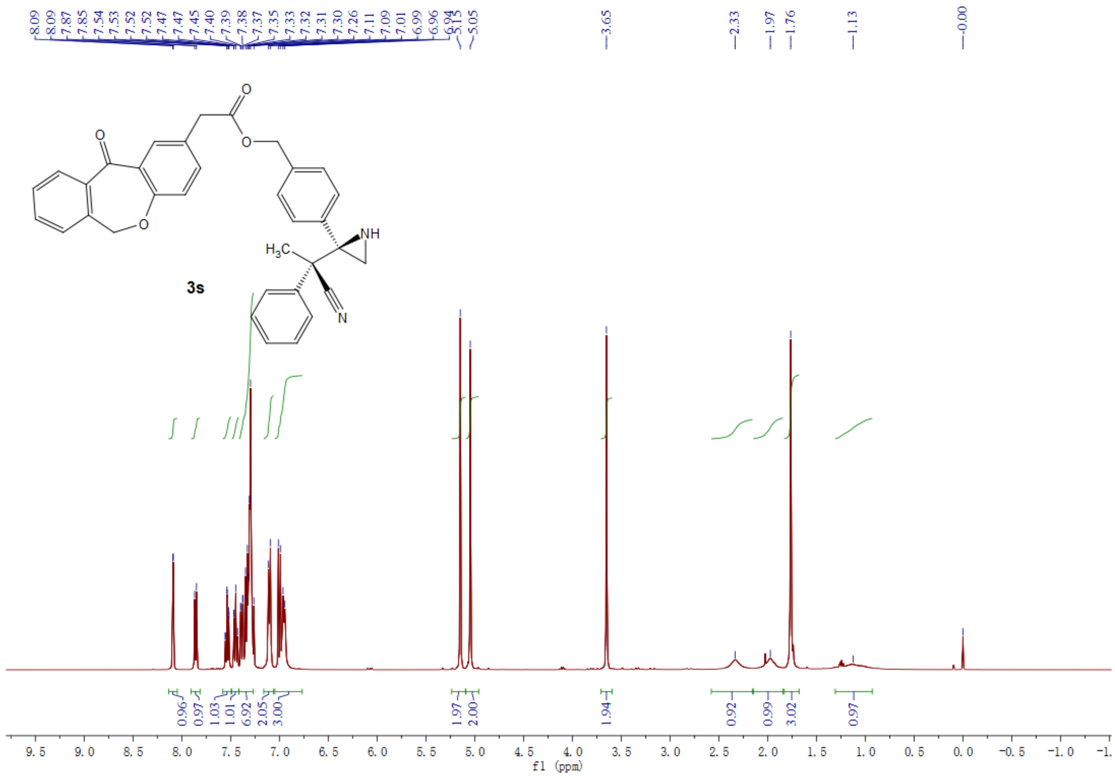

Supplementary Figure 99. ^{1}H NMR spectrum for compound **3s**

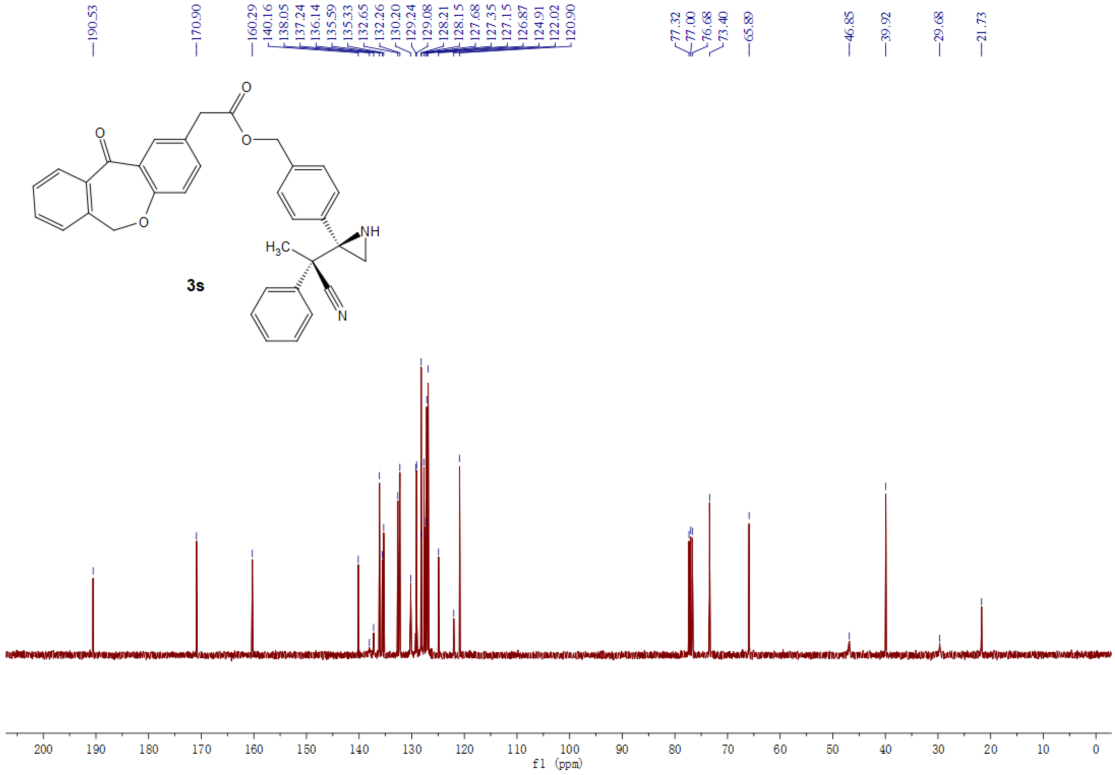

Supplementary Figure 100. ^{13}C NMR spectrum for compound **3s**

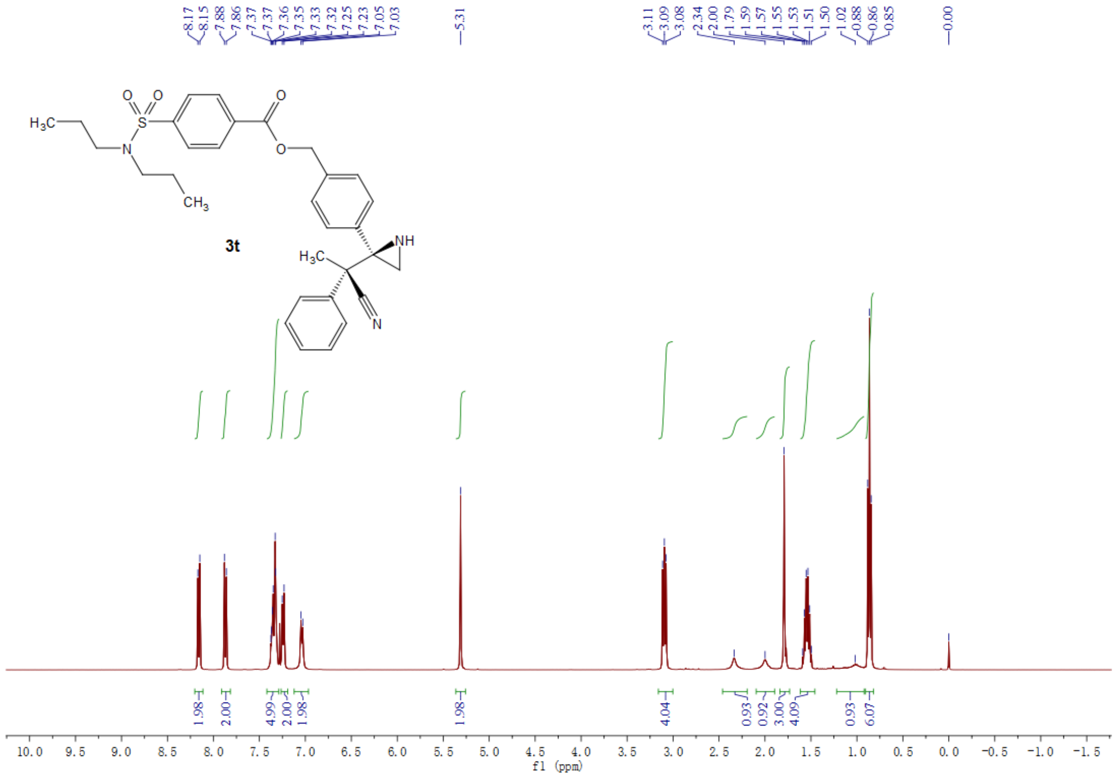

Supplementary Figure 101. ^{1}H NMR spectrum for compound **3t**

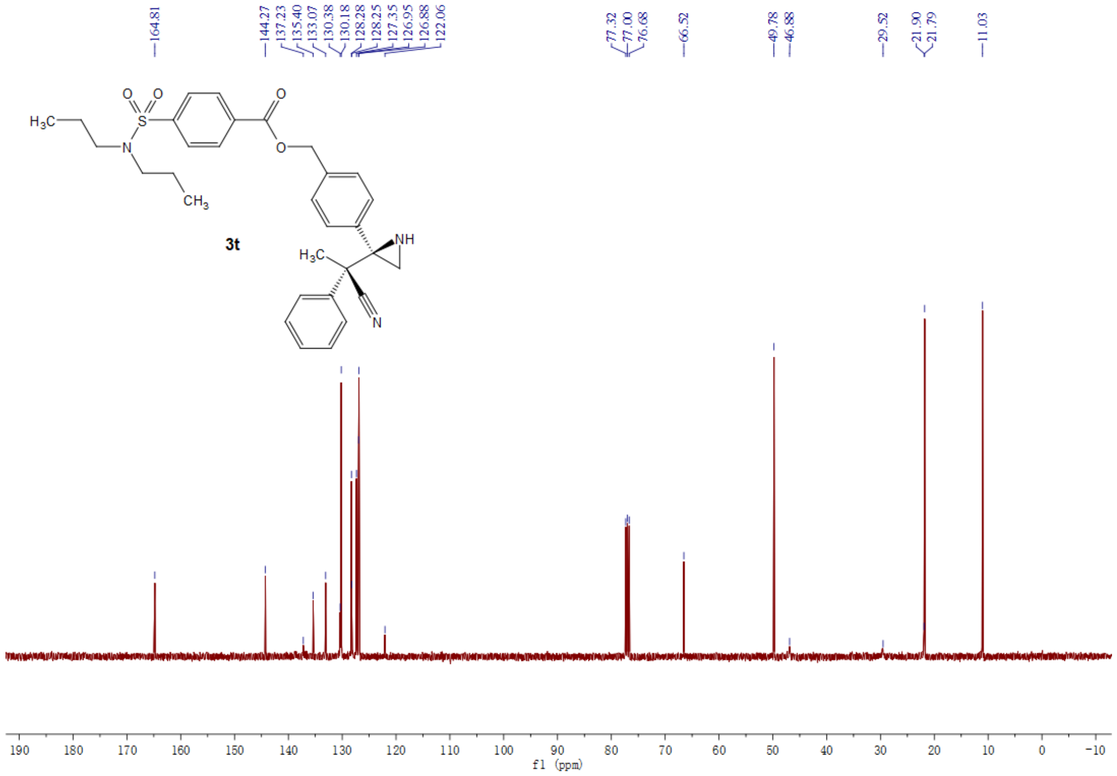

Supplementary Figure 102. ^{13}C NMR spectrum for compound **3t**

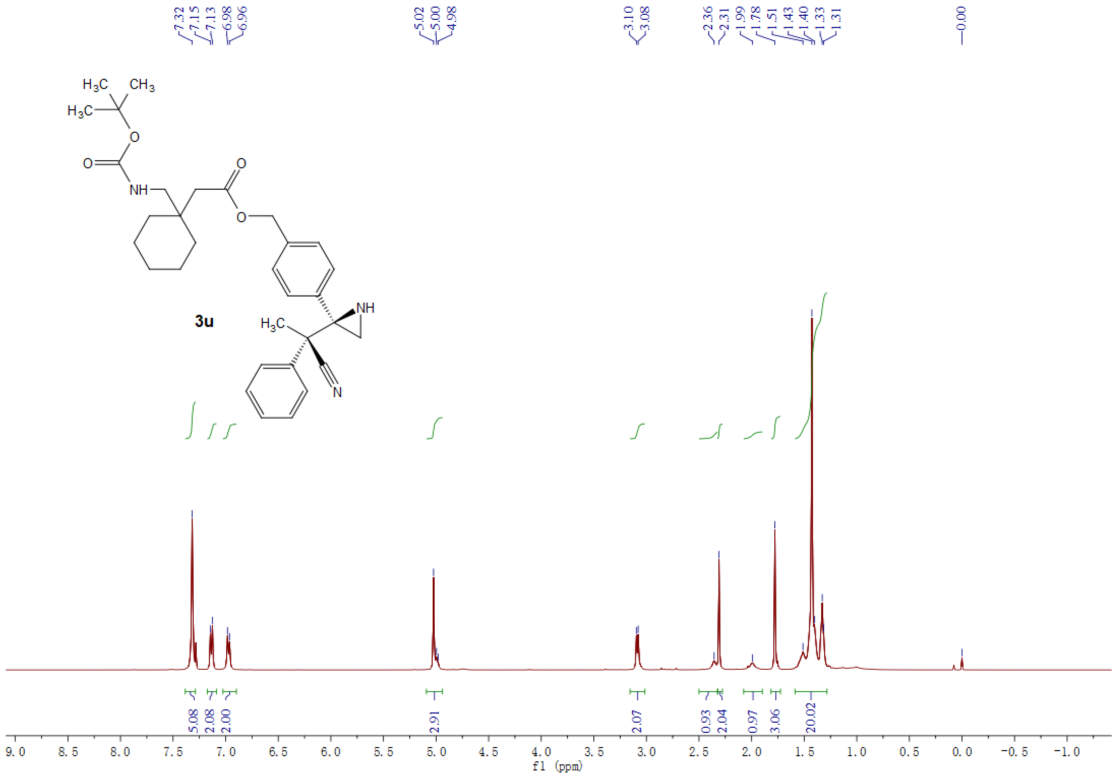

Supplementary Figure 103. ^{1}H NMR spectrum for compound **3u**

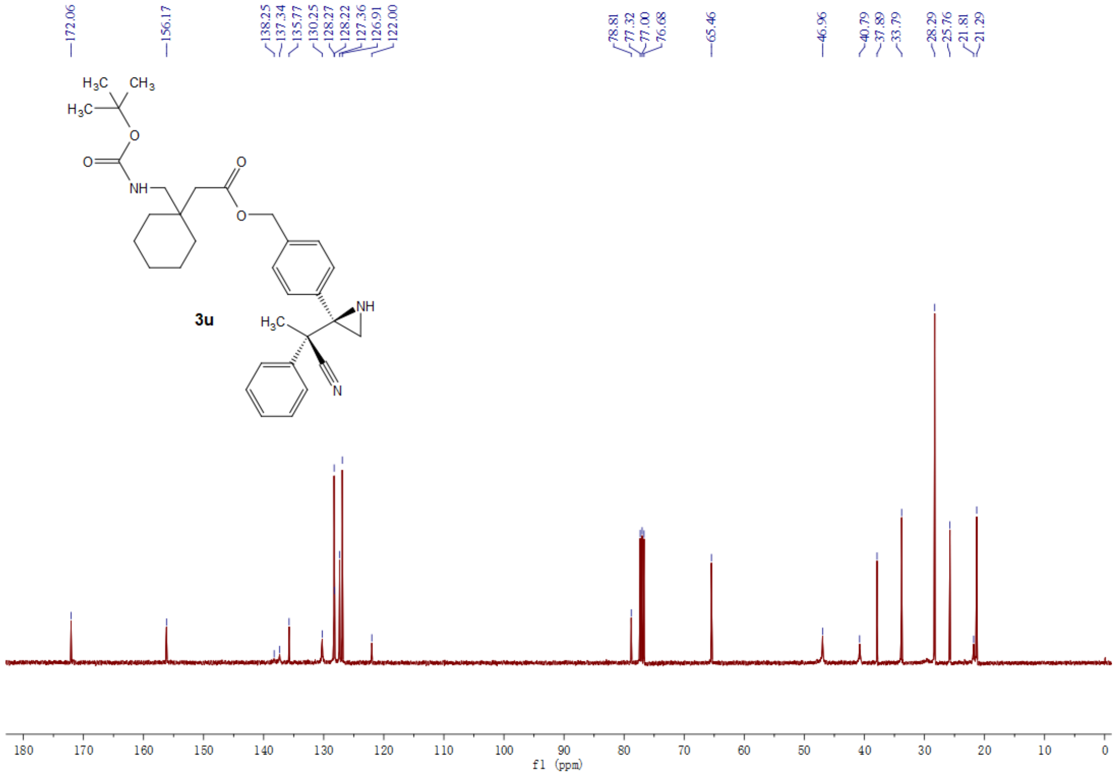

Supplementary Figure 104. ^{13}C NMR spectrum for compound **3u**

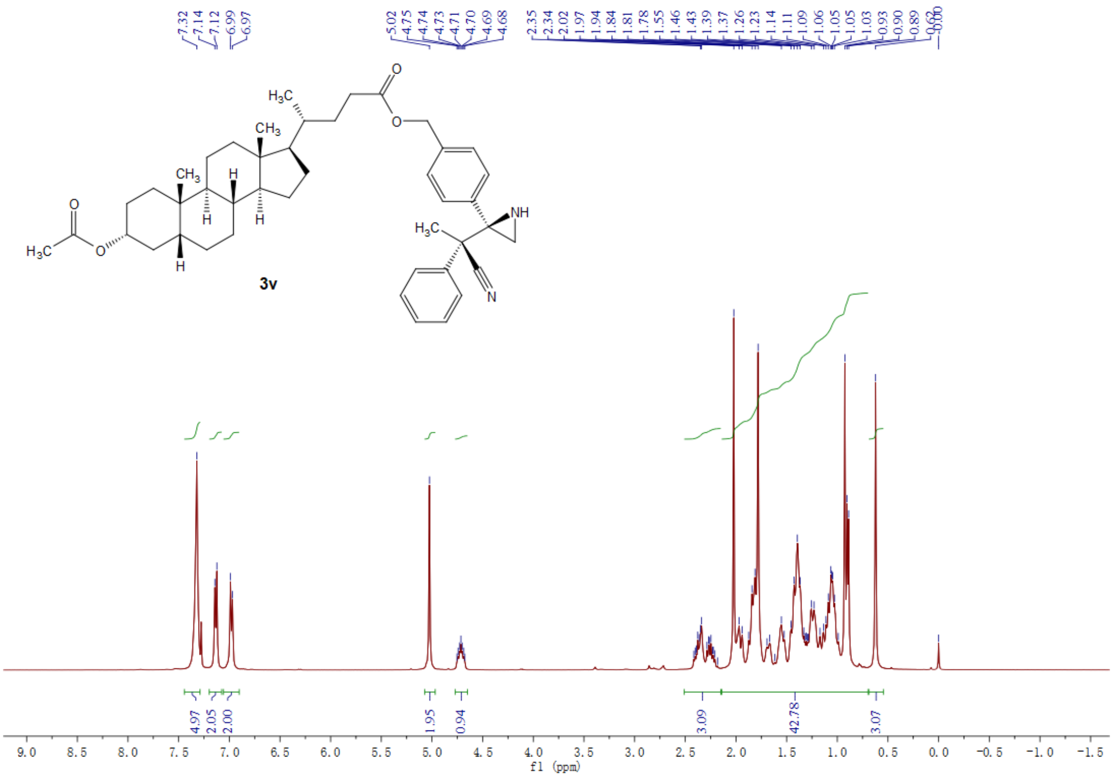

Supplementary Figure 105. ^{1}H NMR spectrum for compound **3v**

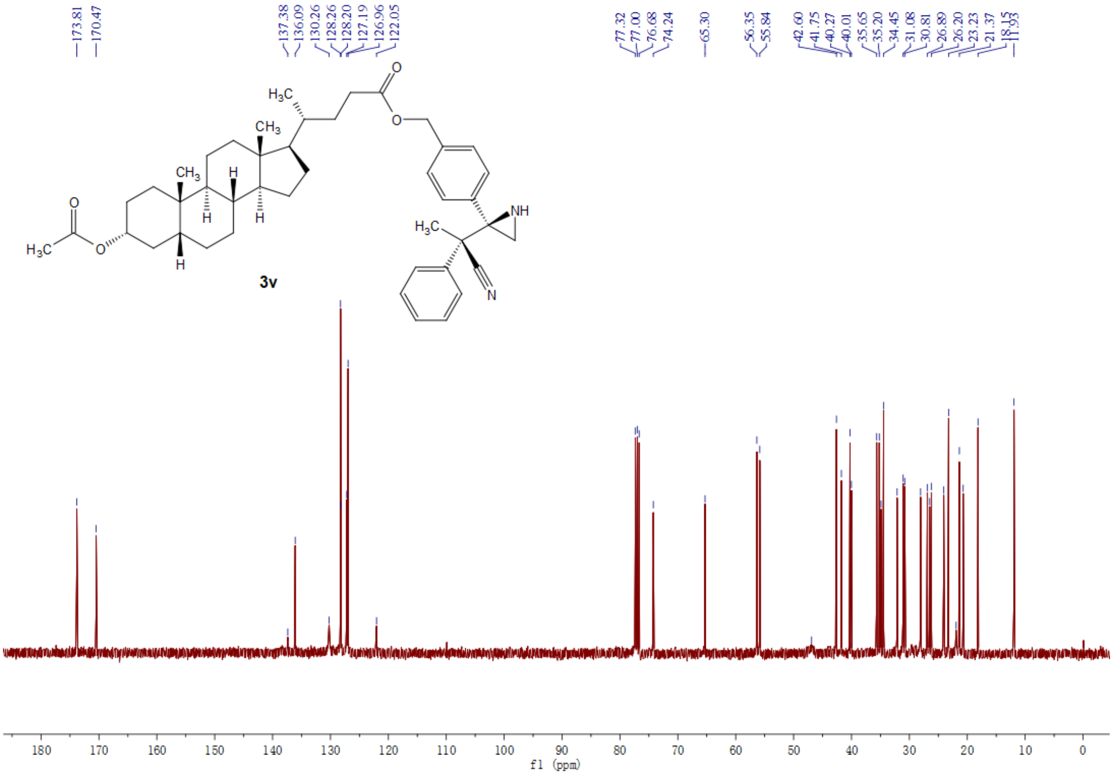

Supplementary Figure 106. ^{13}C NMR spectrum for compound **3v**

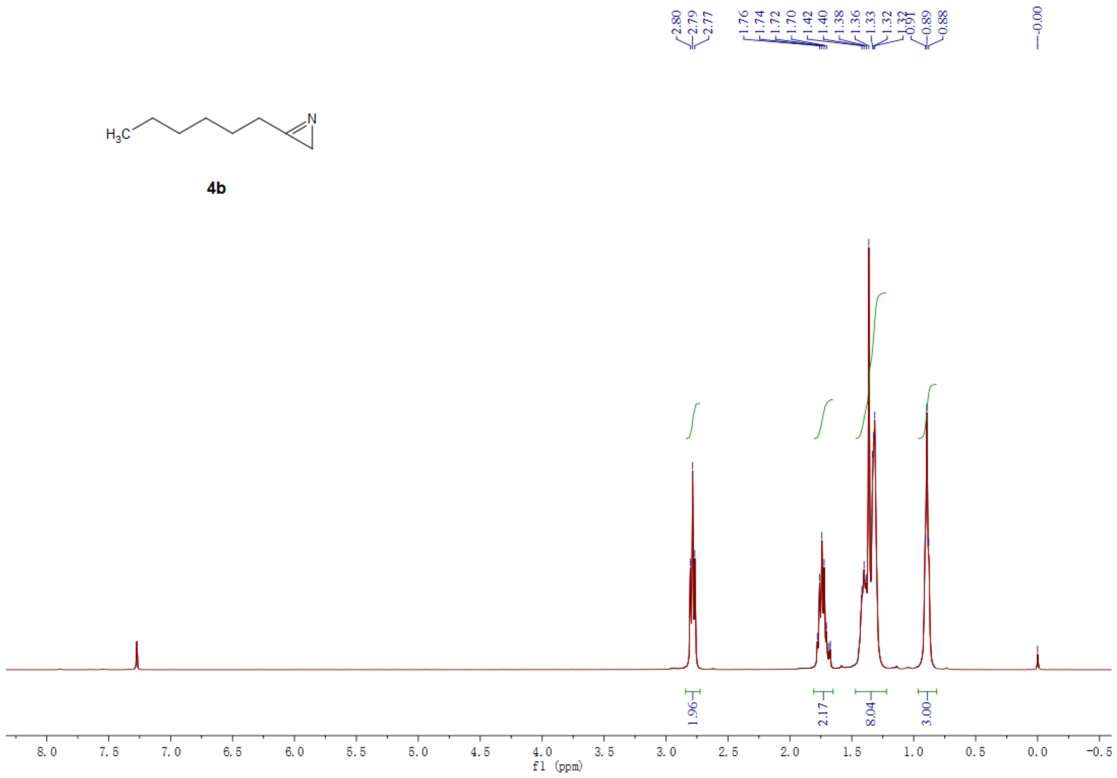

Supplementary Figure 107. ^{1}H NMR spectrum for compound **4b**

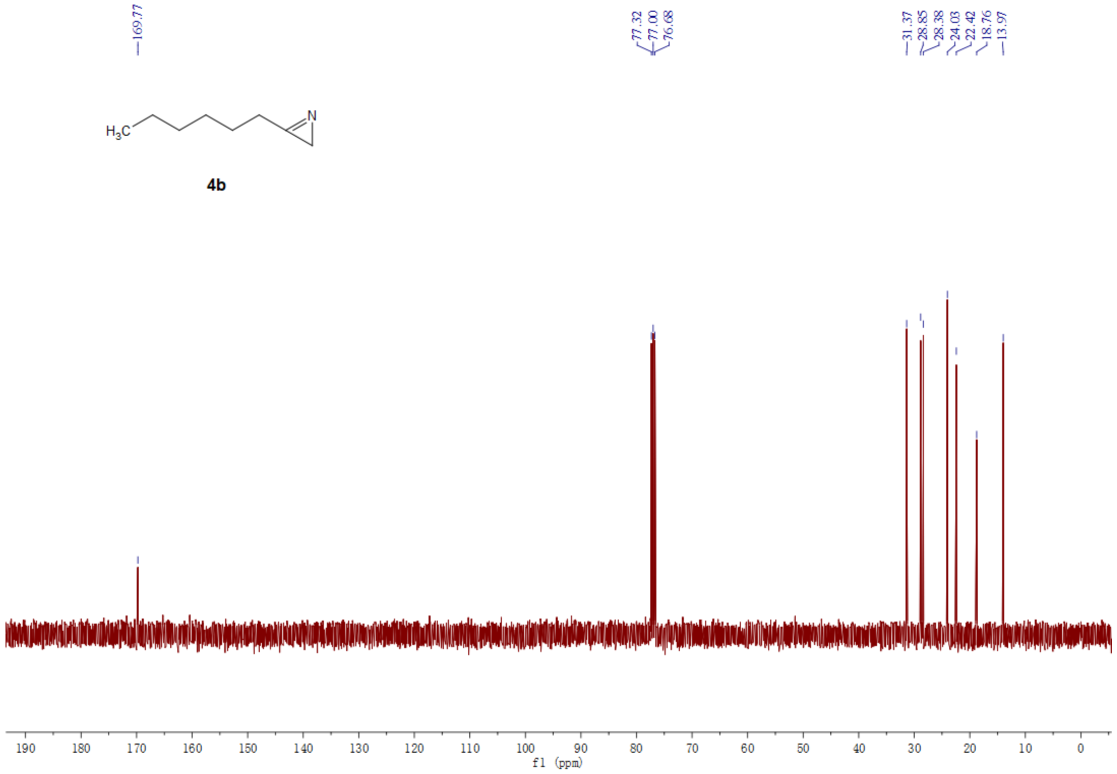

Supplementary Figure 108. ^{13}C NMR spectrum for compound **4b**

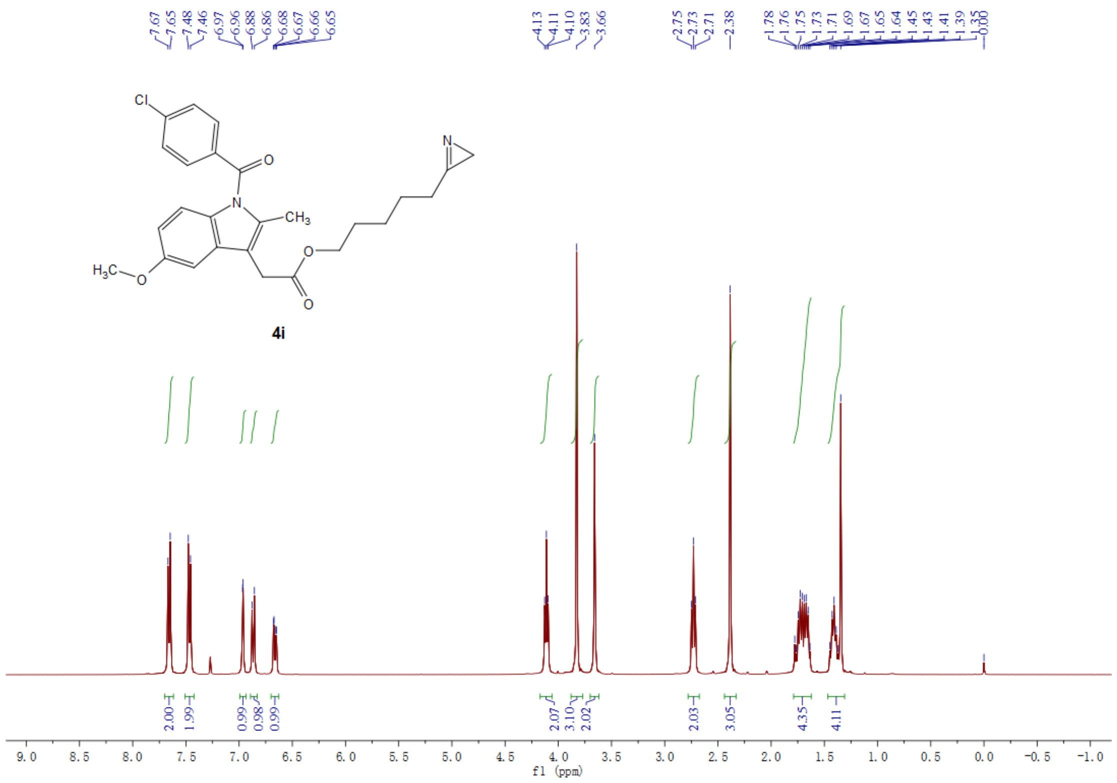

Supplementary Figure 109. ^{1}H NMR spectrum for compound **4i**

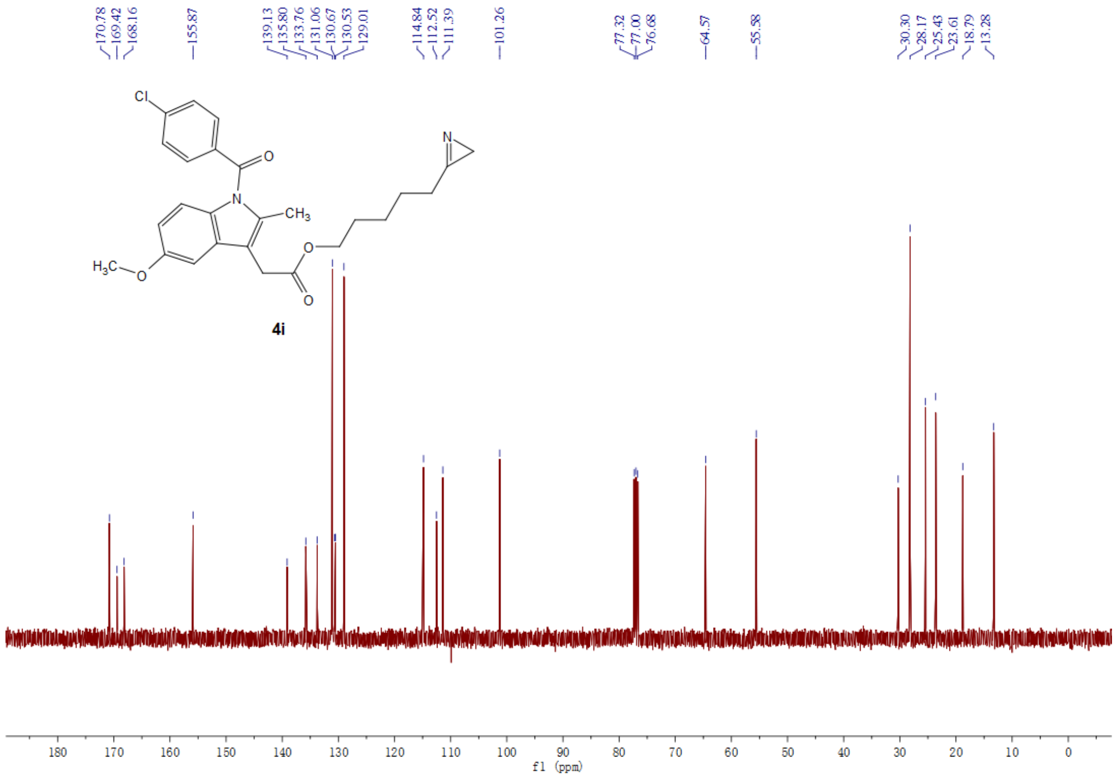

Supplementary Figure 110. ^{13}C NMR spectrum for compound **4i**

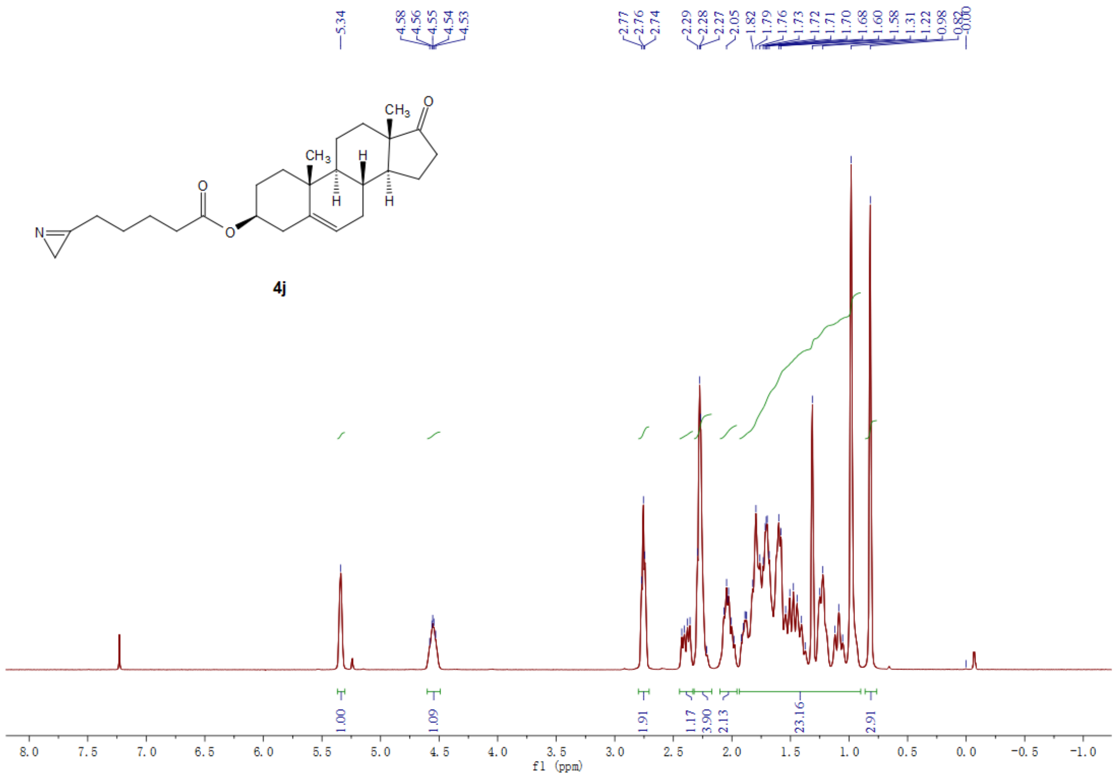

Supplementary Figure 111. ^{1}H NMR spectrum for compound **4j**

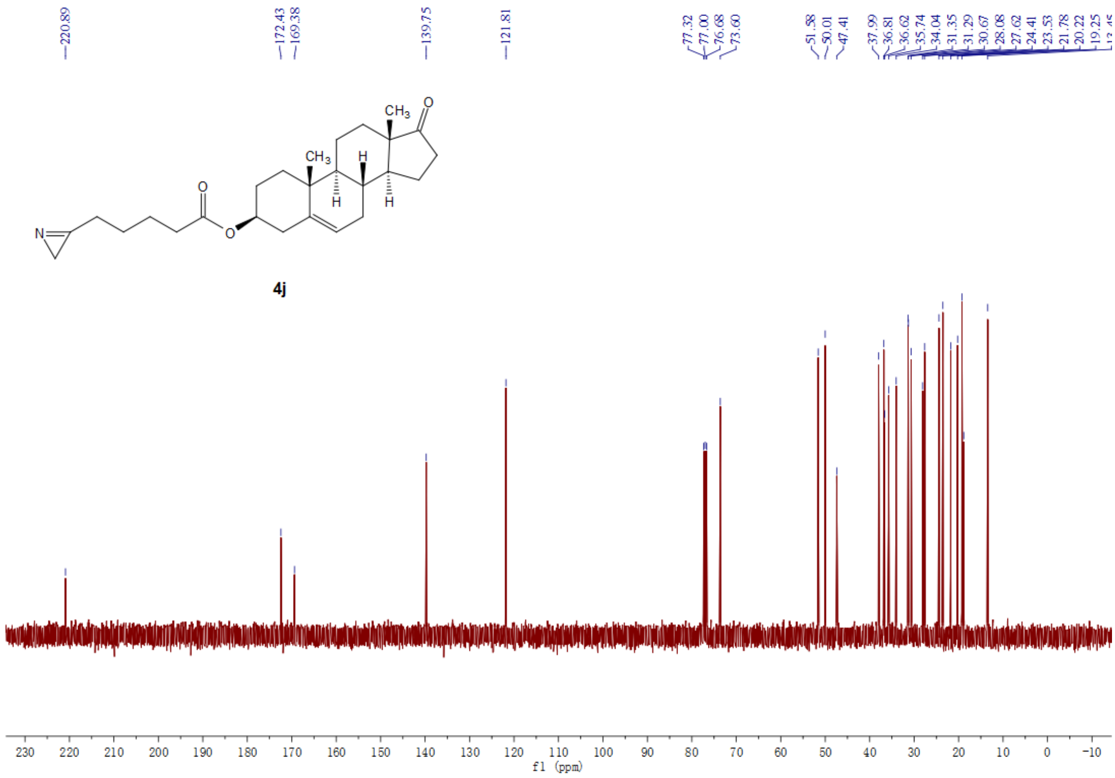

Supplementary Figure 112. ^{13}C NMR spectrum for compound **4j**

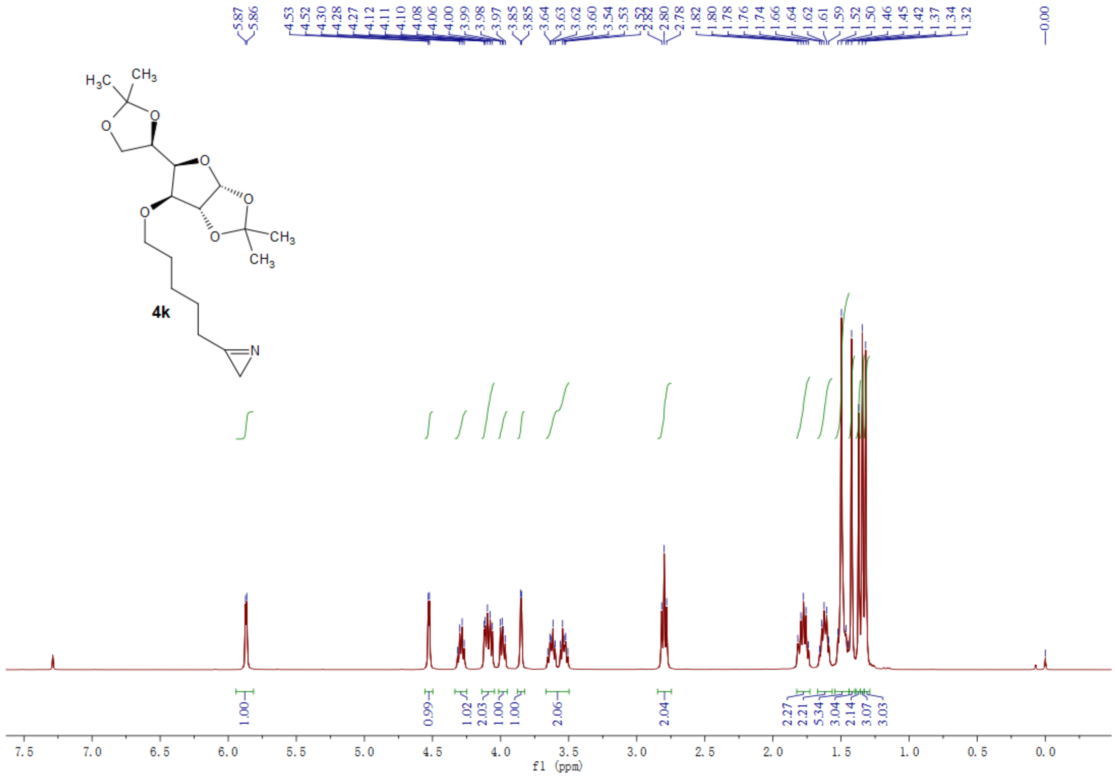

Supplementary Figure 113. ^{1}H NMR spectrum for compound **4k**

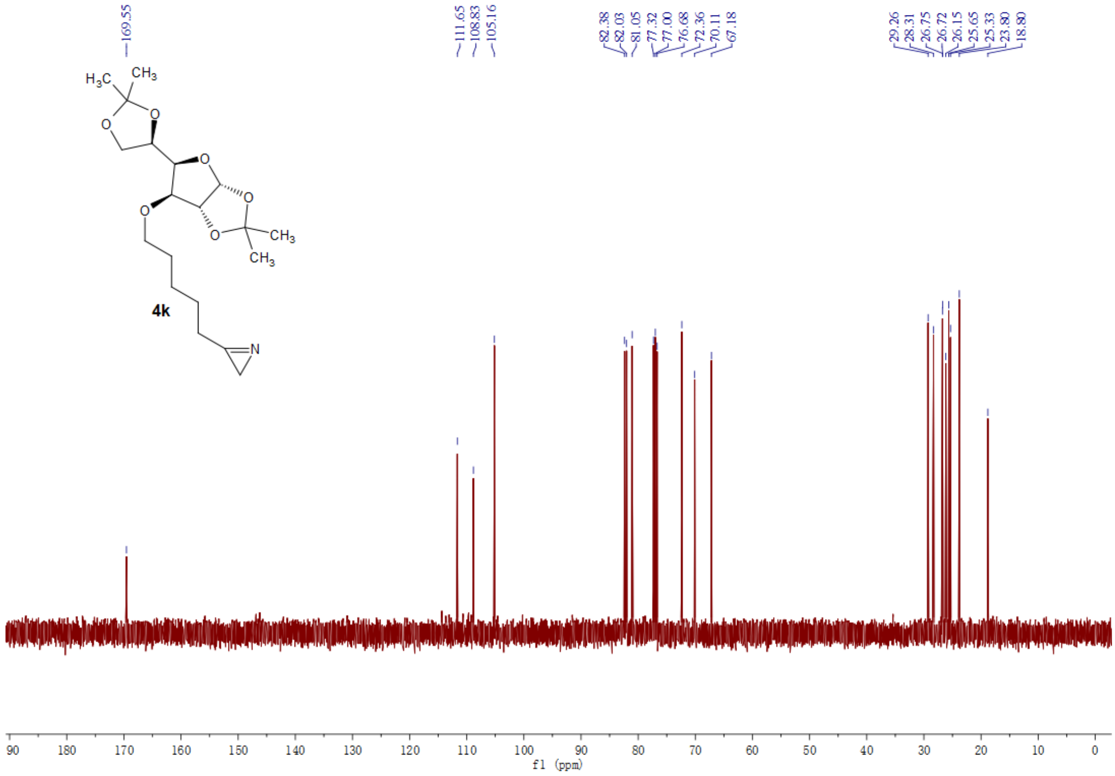

Supplementary Figure 114. ^{13}C NMR spectrum for compound **4k**

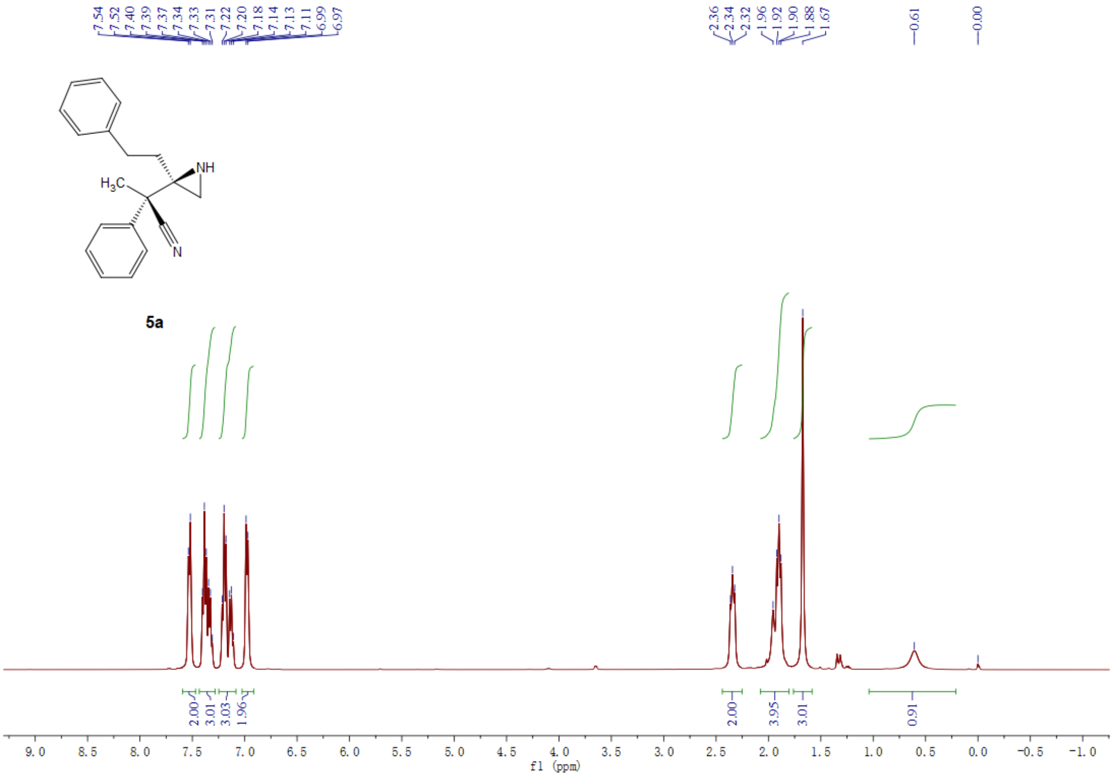

Supplementary Figure 115. ^{1}H NMR spectrum for compound **5a**

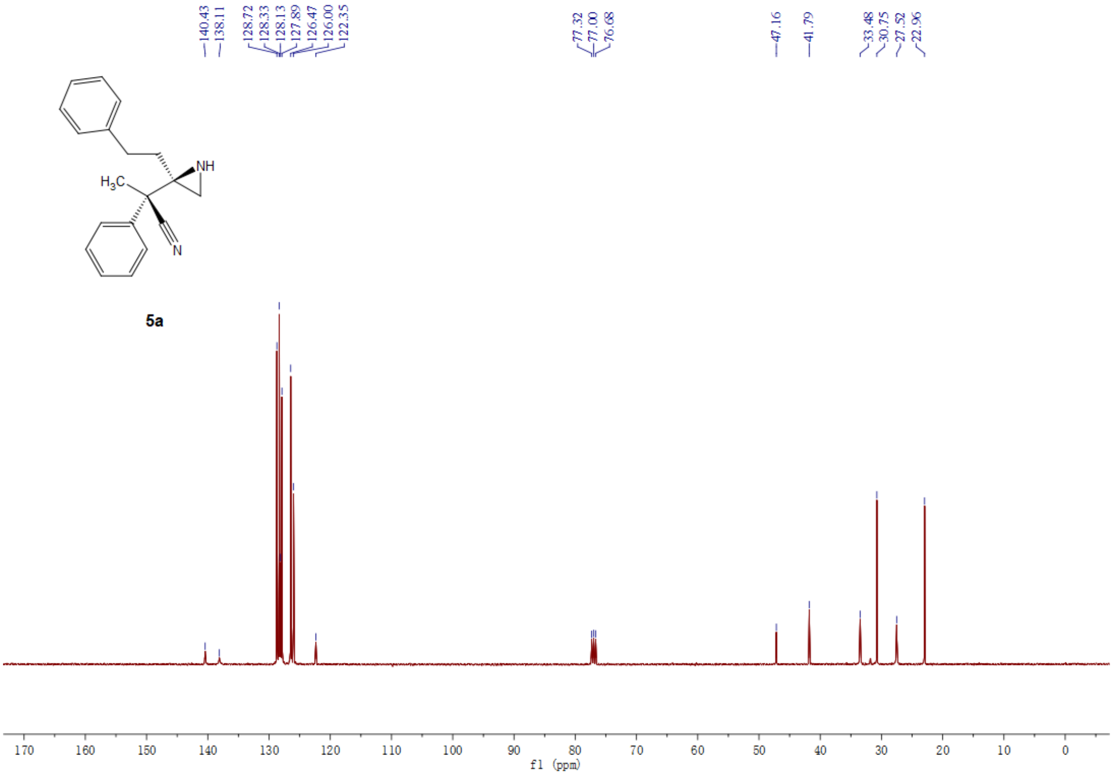

Supplementary Figure 116. ^{13}C NMR spectrum for compound **5a**

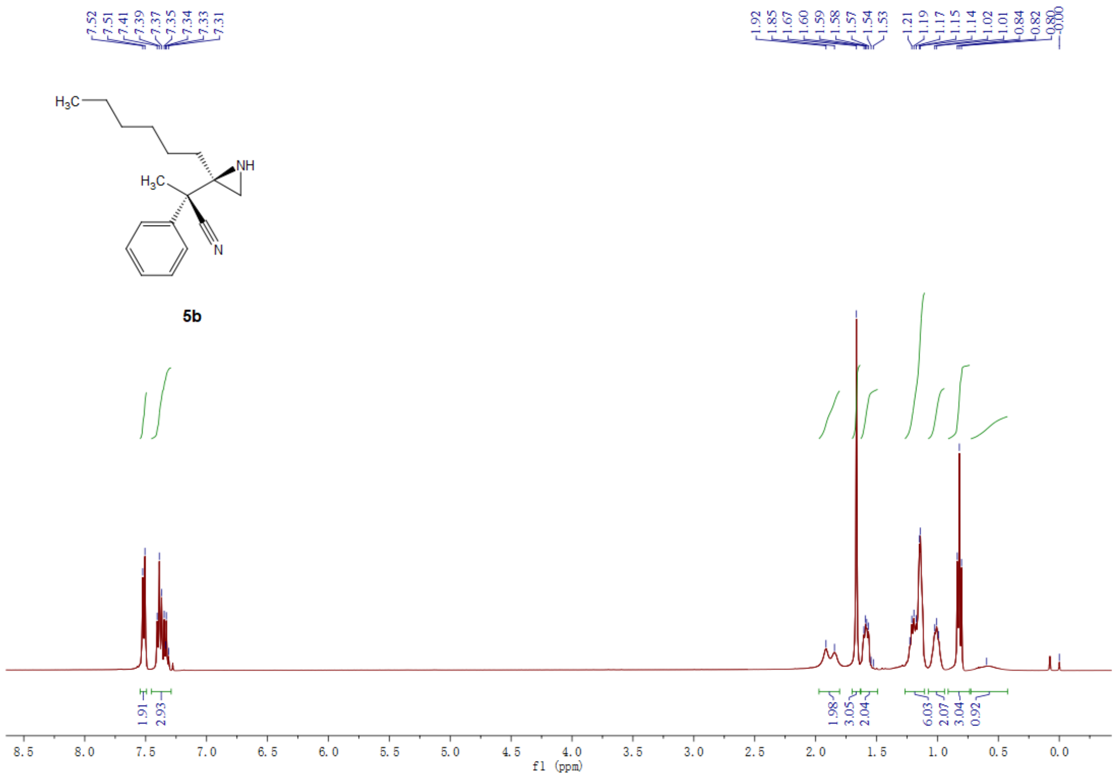

Supplementary Figure 117. ^{1}H NMR spectrum for compound **5b**

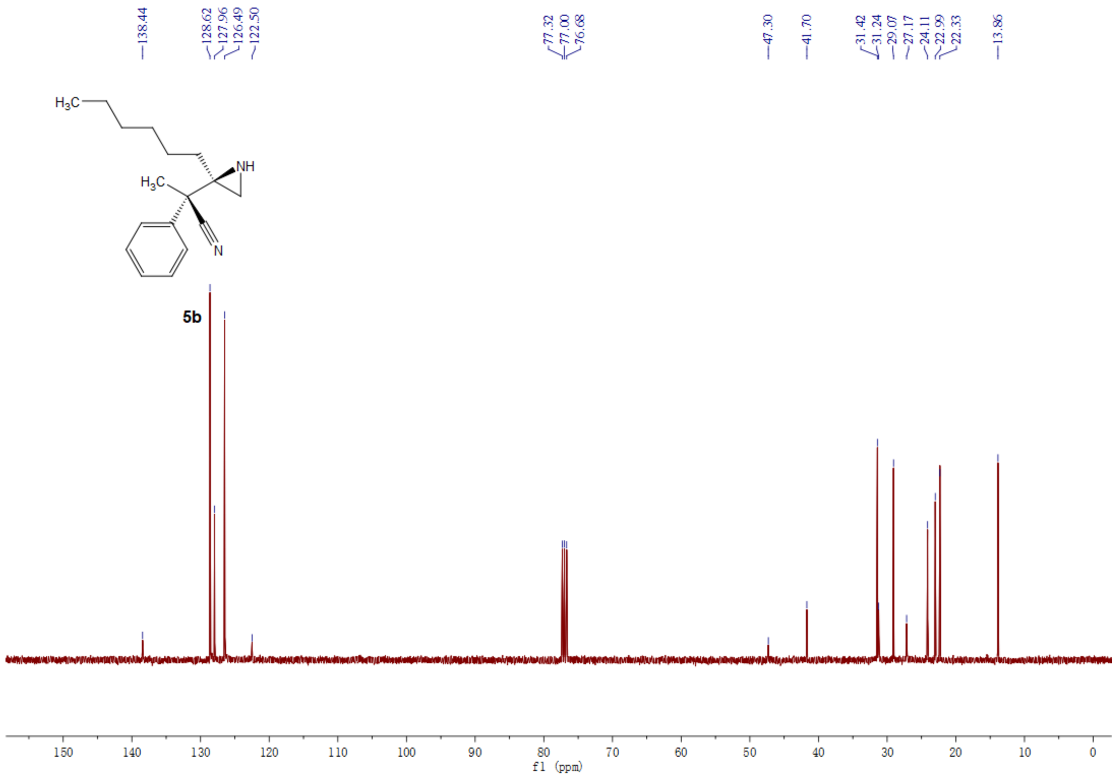

Supplementary Figure 118. ^{13}C NMR spectrum for compound **5b**

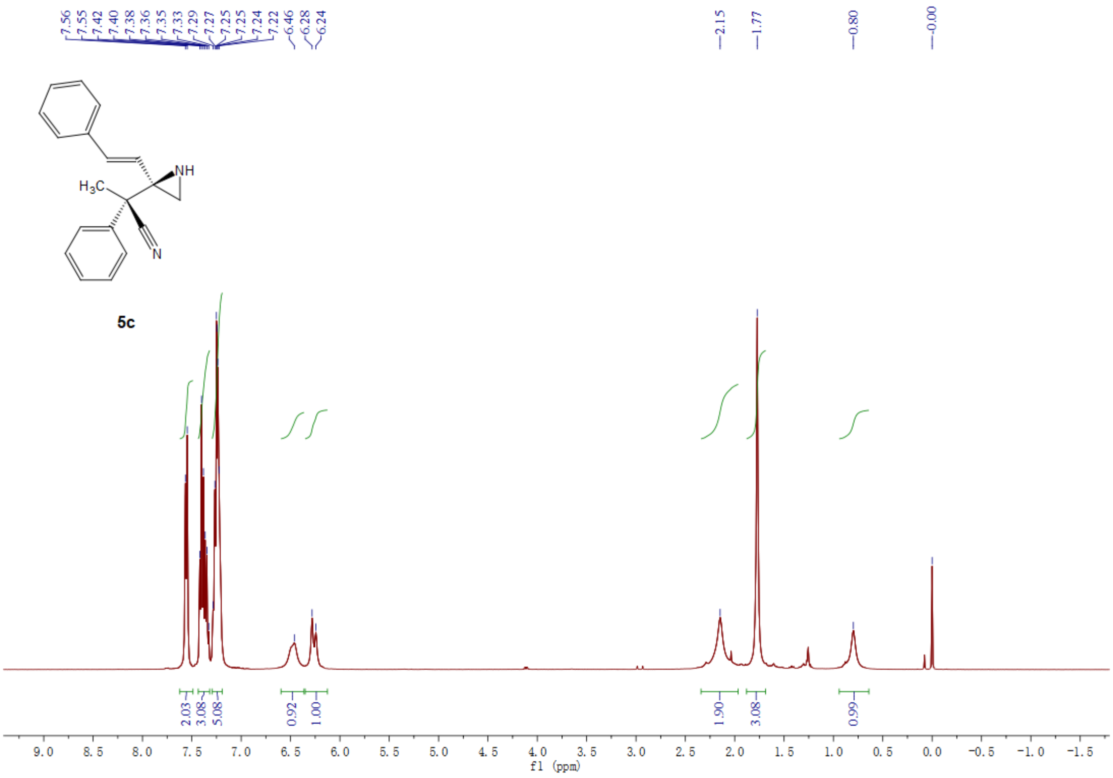

Supplementary Figure 119. ^{1}H NMR spectrum for compound **5c**

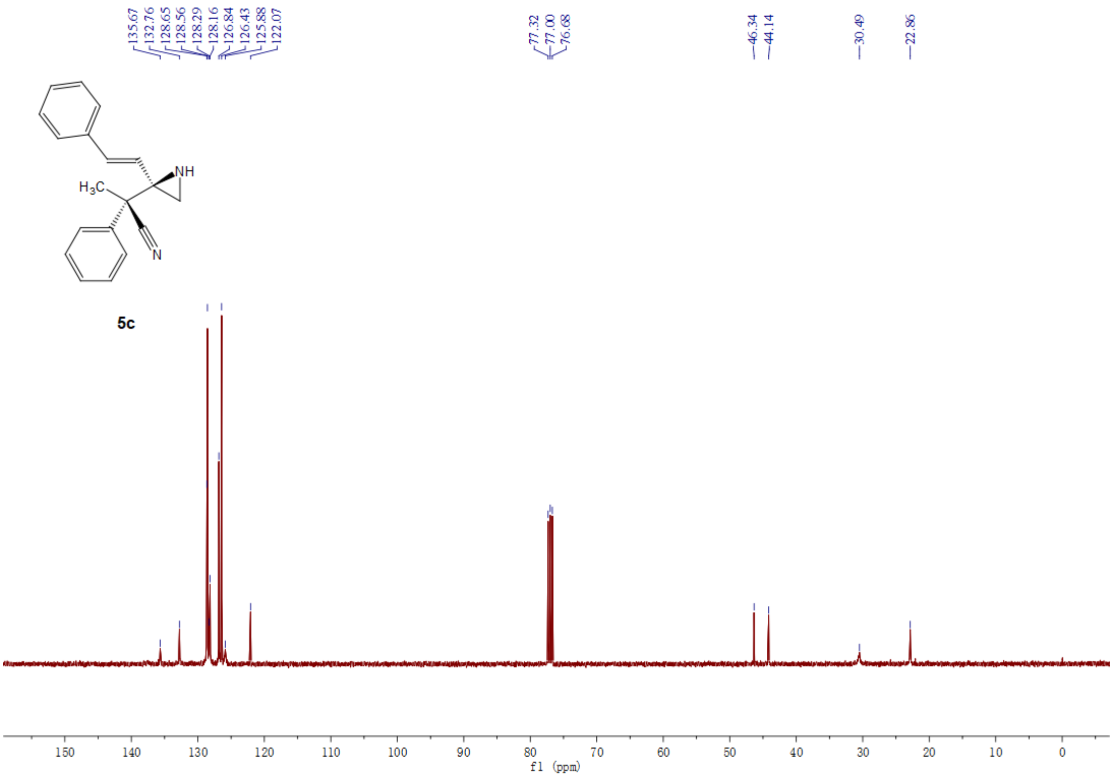

Supplementary Figure 120. ^{13}C NMR spectrum for compound **5c**

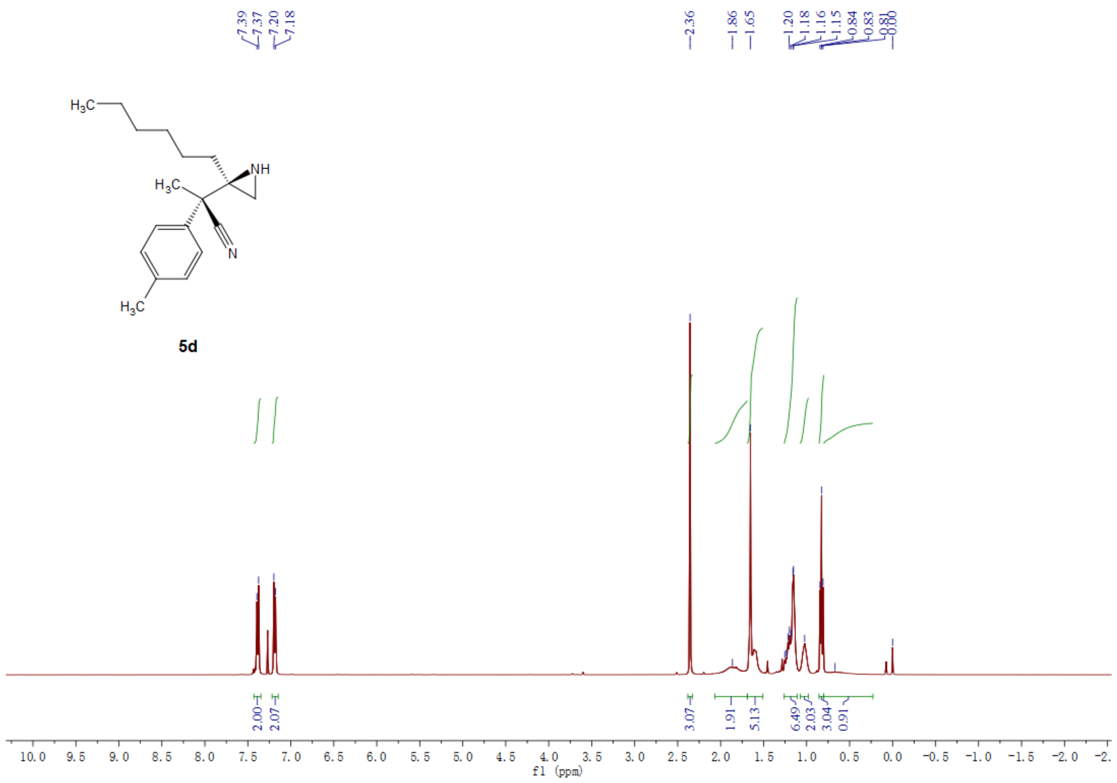

Supplementary Figure 121. ^{1}H NMR spectrum for compound **5d**

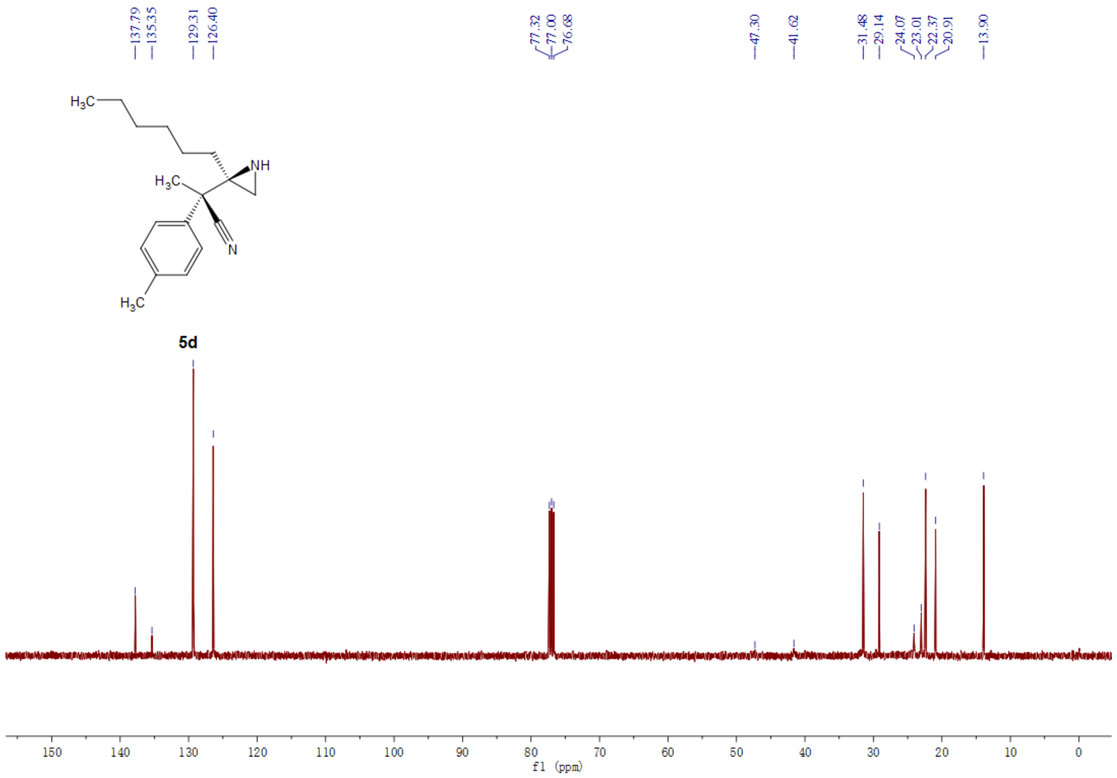

Supplementary Figure 122. ^{13}C NMR spectrum for compound **5d**

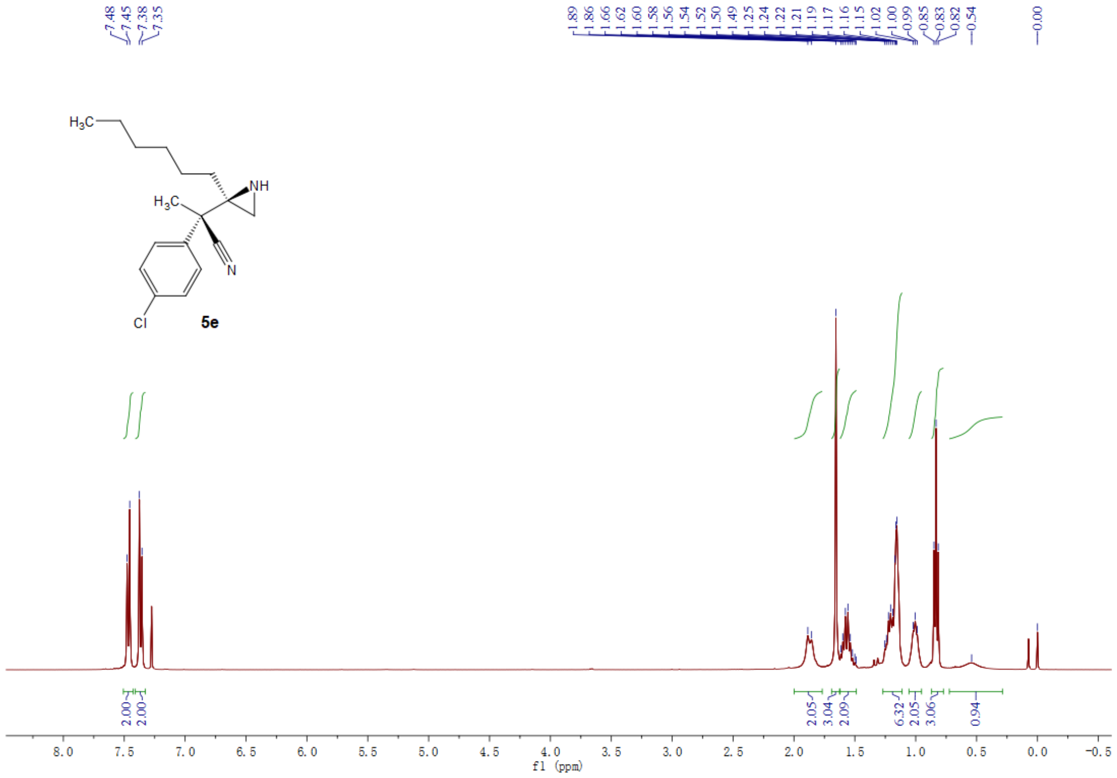

Supplementary Figure 123. ^{1}H NMR spectrum for compound **5e**

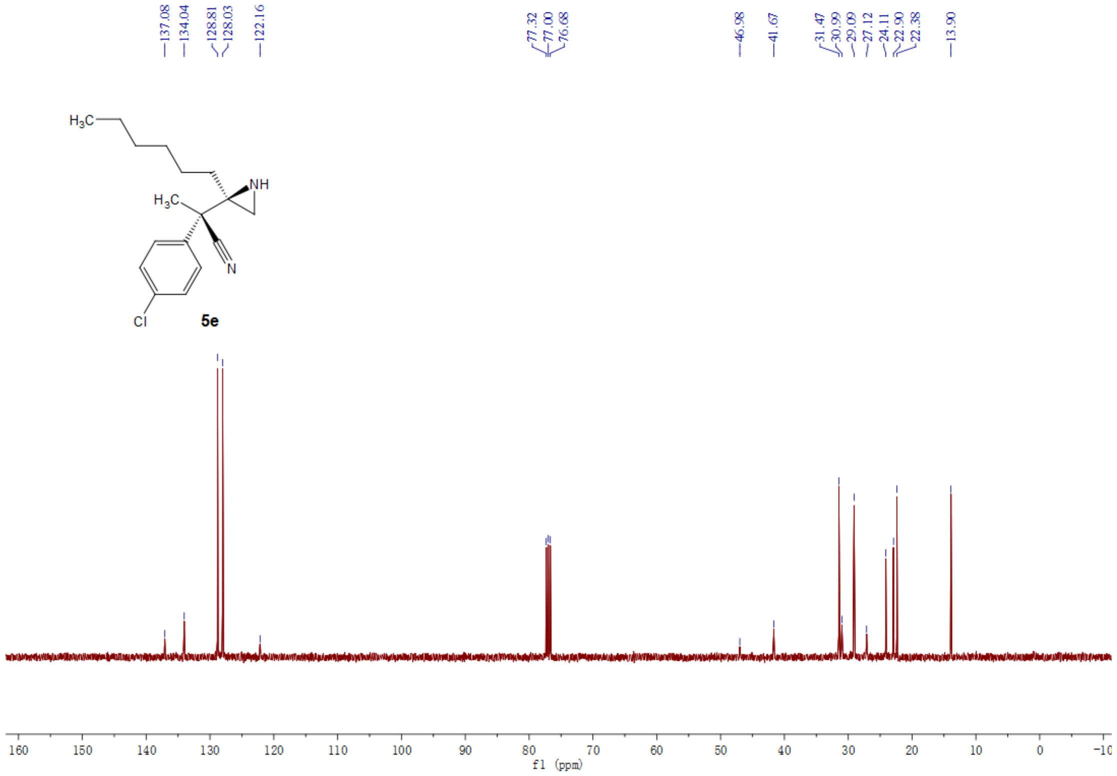

Supplementary Figure 124. ^{13}C NMR spectrum for compound **5e**

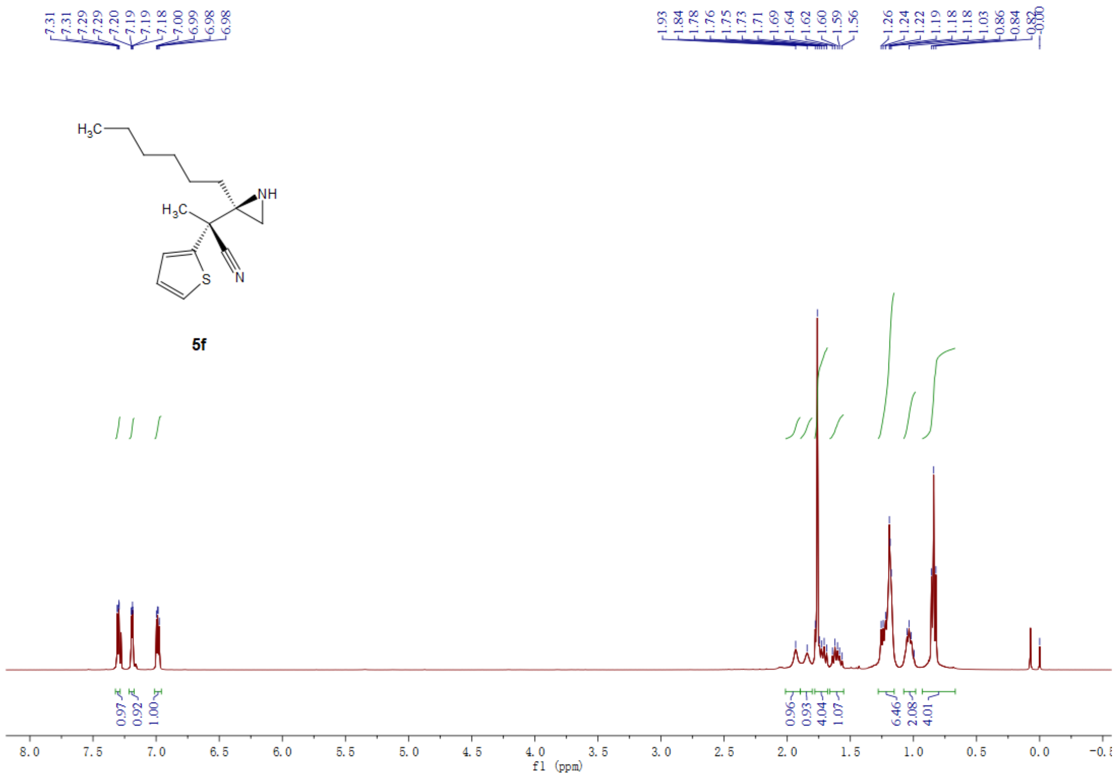

Supplementary Figure 125. ^{1}H NMR spectrum for compound **5f**

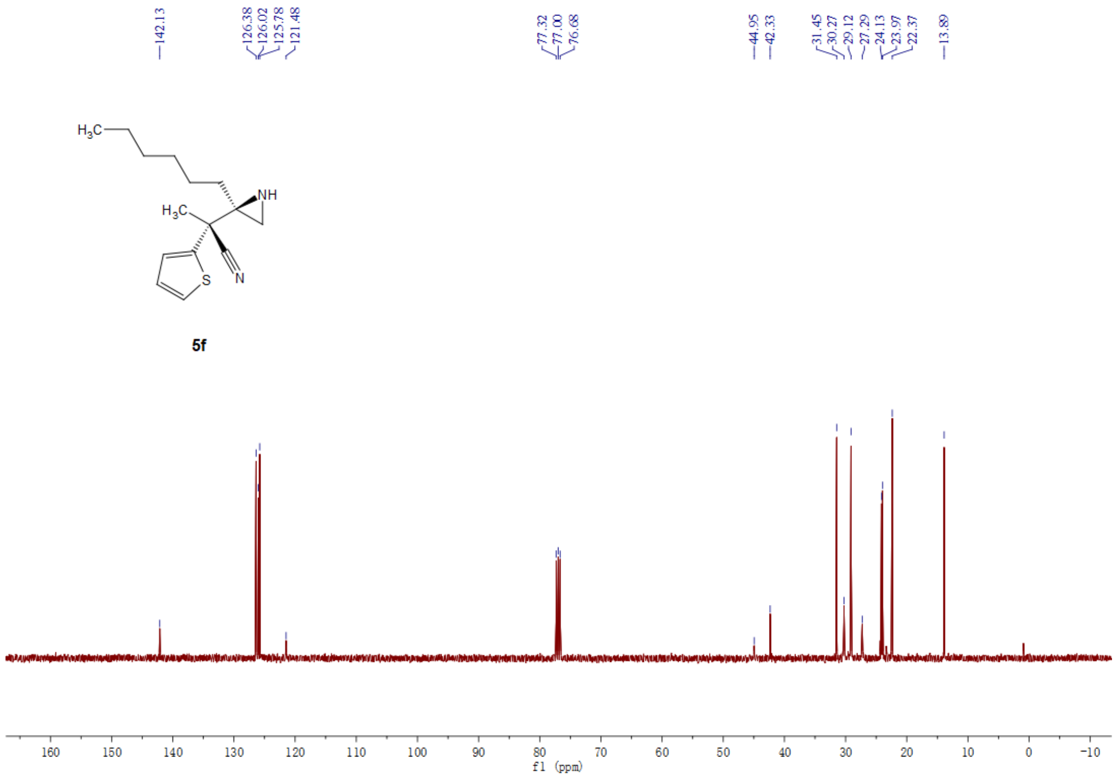

Supplementary Figure 126. ^{13}C NMR spectrum for compound **5f**

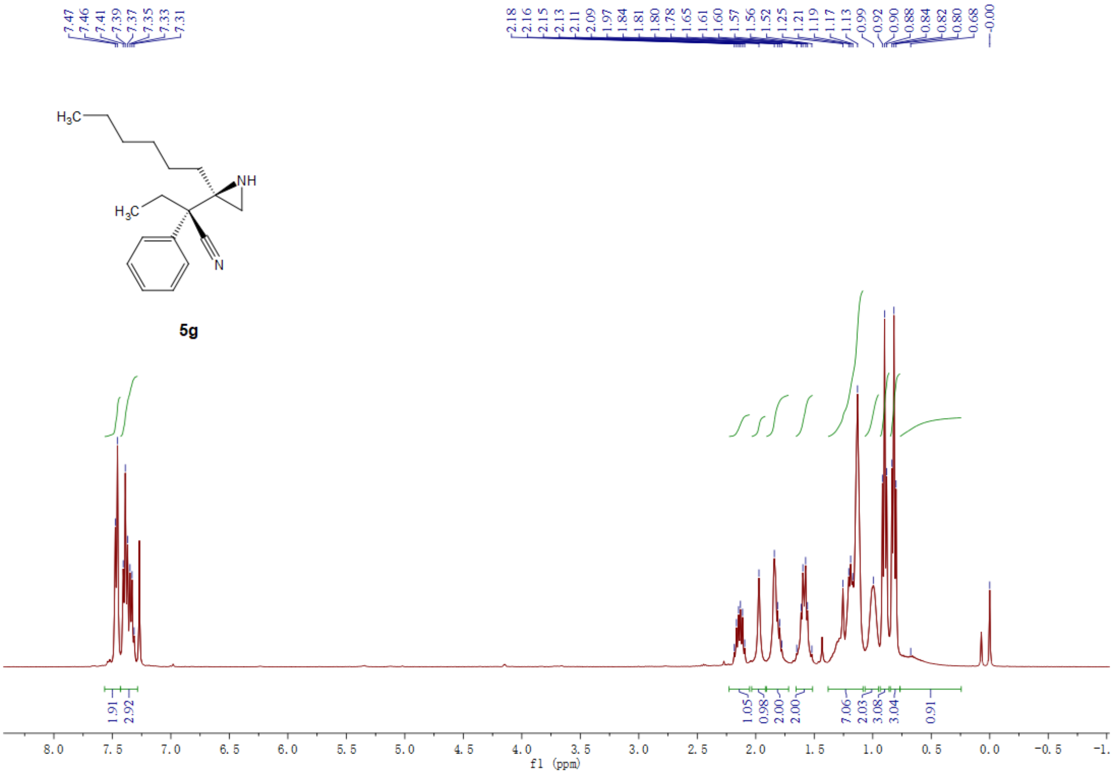

Supplementary Figure 127. ^{1}H NMR spectrum for compound **5g**

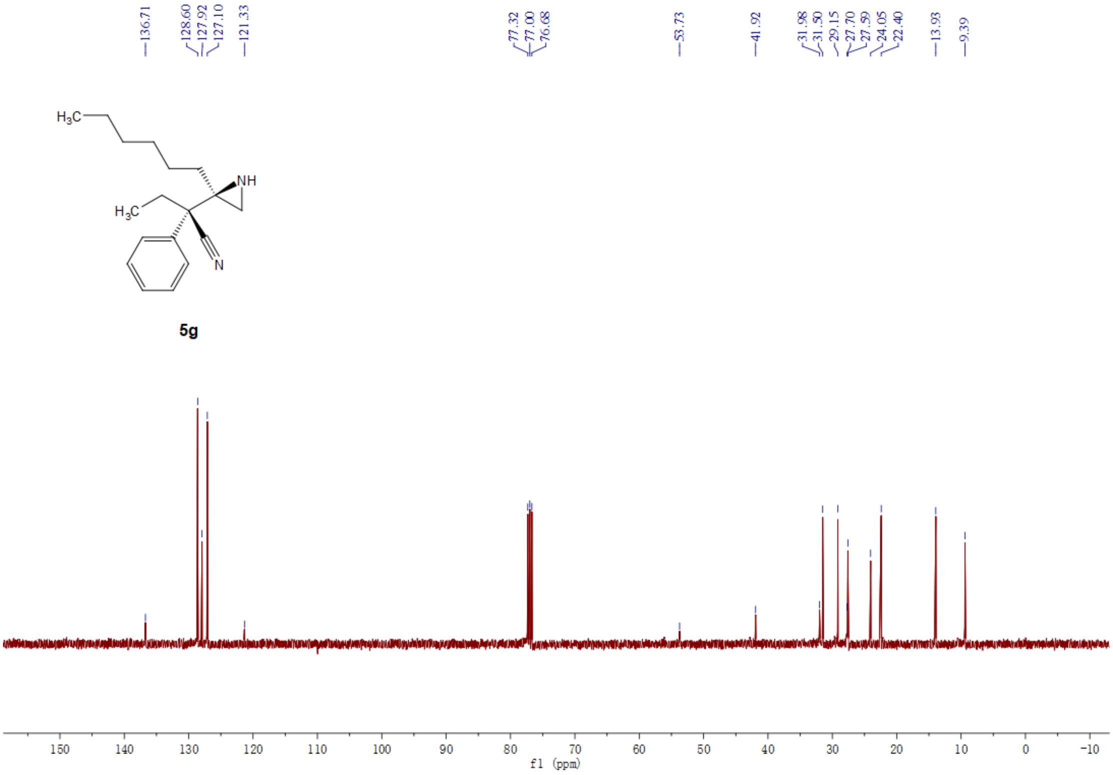

Supplementary Figure 128. ^{13}C NMR spectrum for compound **5g**

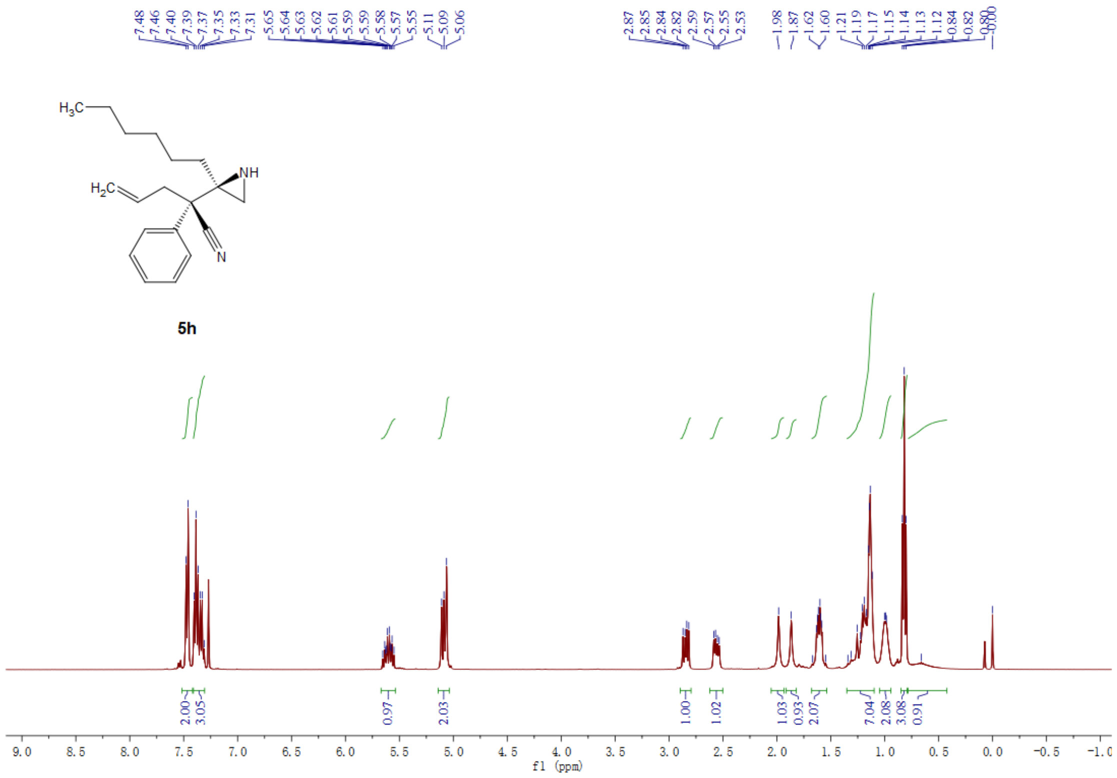

Supplementary Figure 129. ^{1}H NMR spectrum for compound **5h**

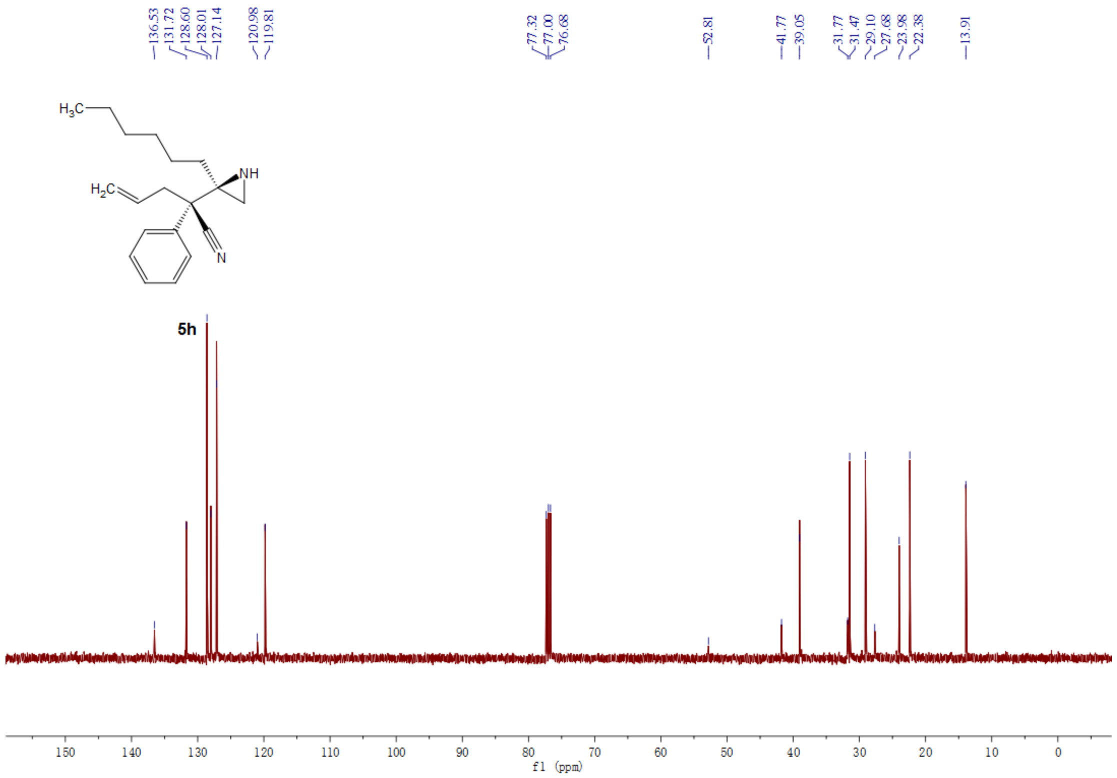

Supplementary Figure 130. ^{13}C NMR spectrum for compound **5h**

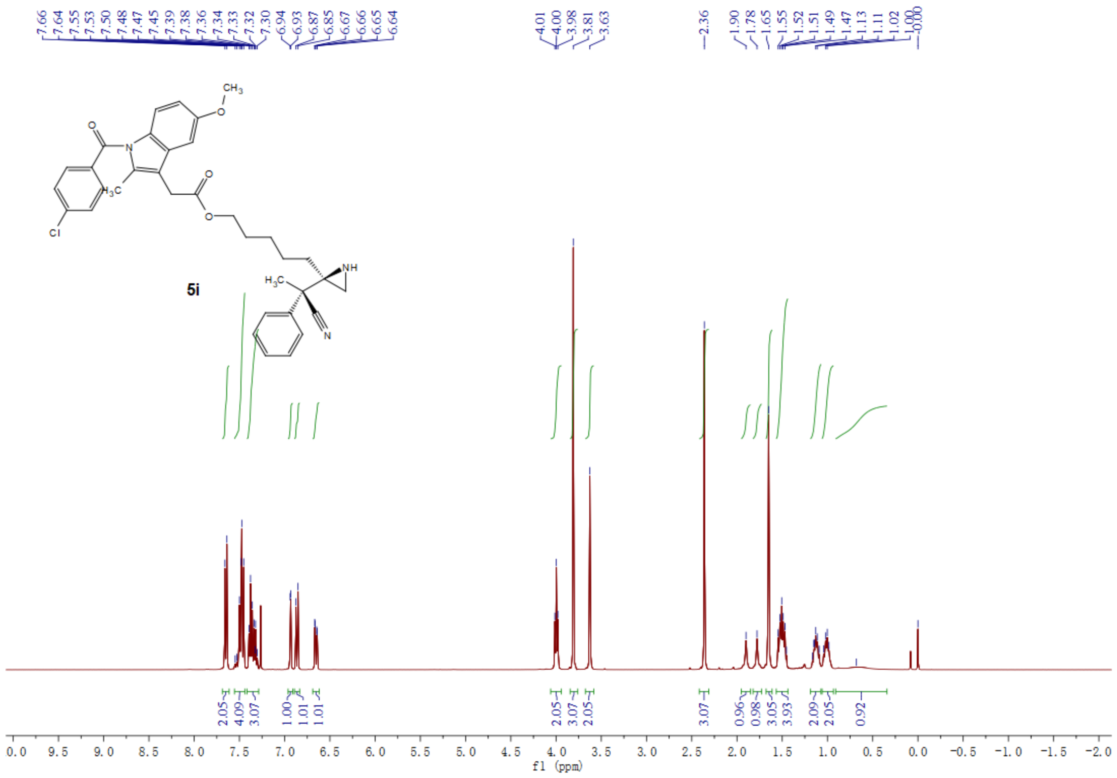

Supplementary Figure 131. ^{1}H NMR spectrum for compound **5i**

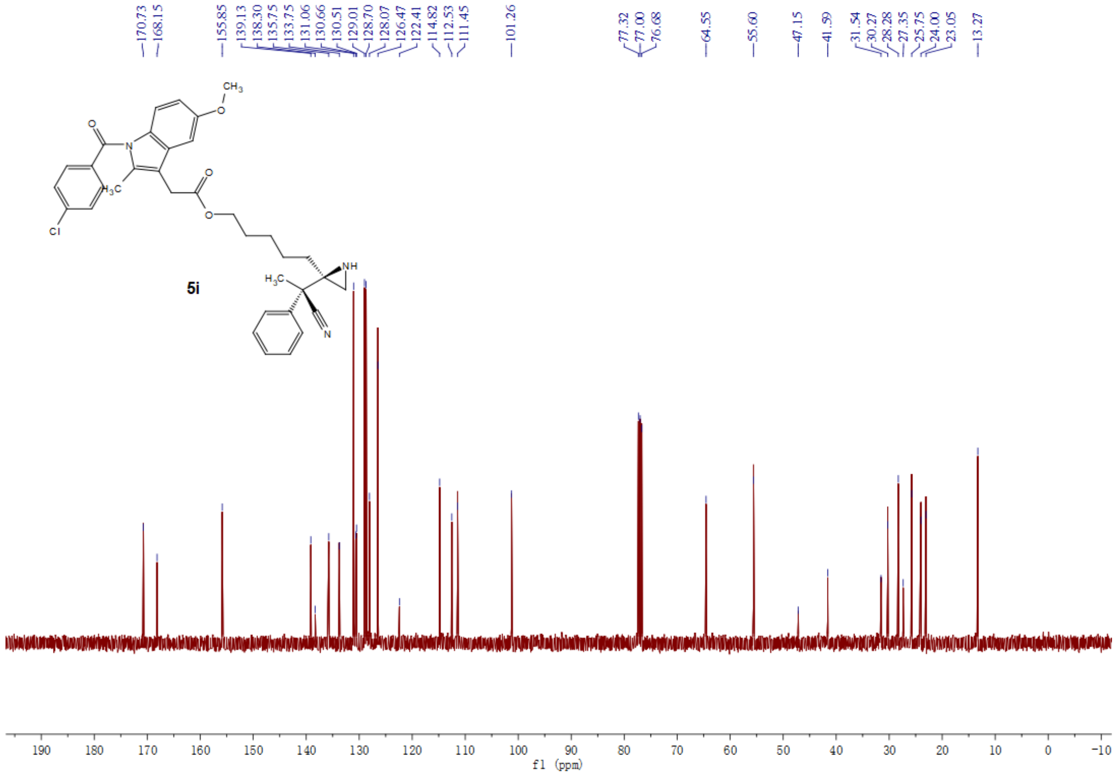

Supplementary Figure 132. ^{13}C NMR spectrum for compound **5i**

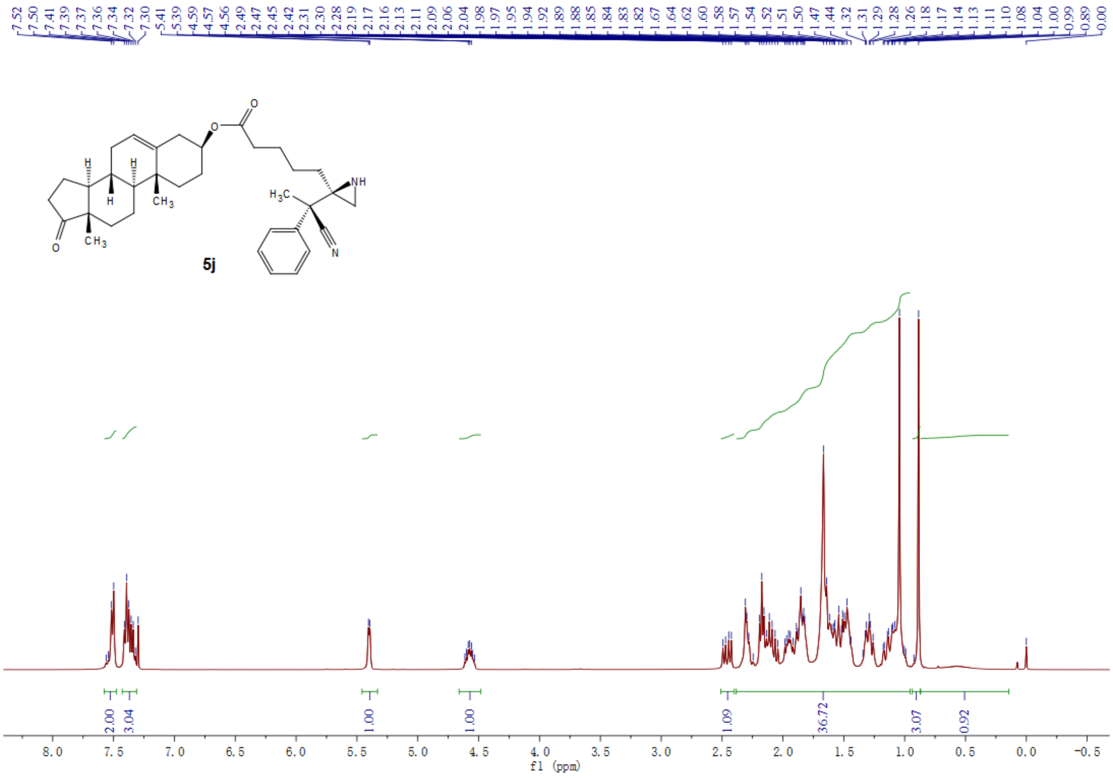

Supplementary Figure 133. ^{1}H NMR spectrum for compound **5j**

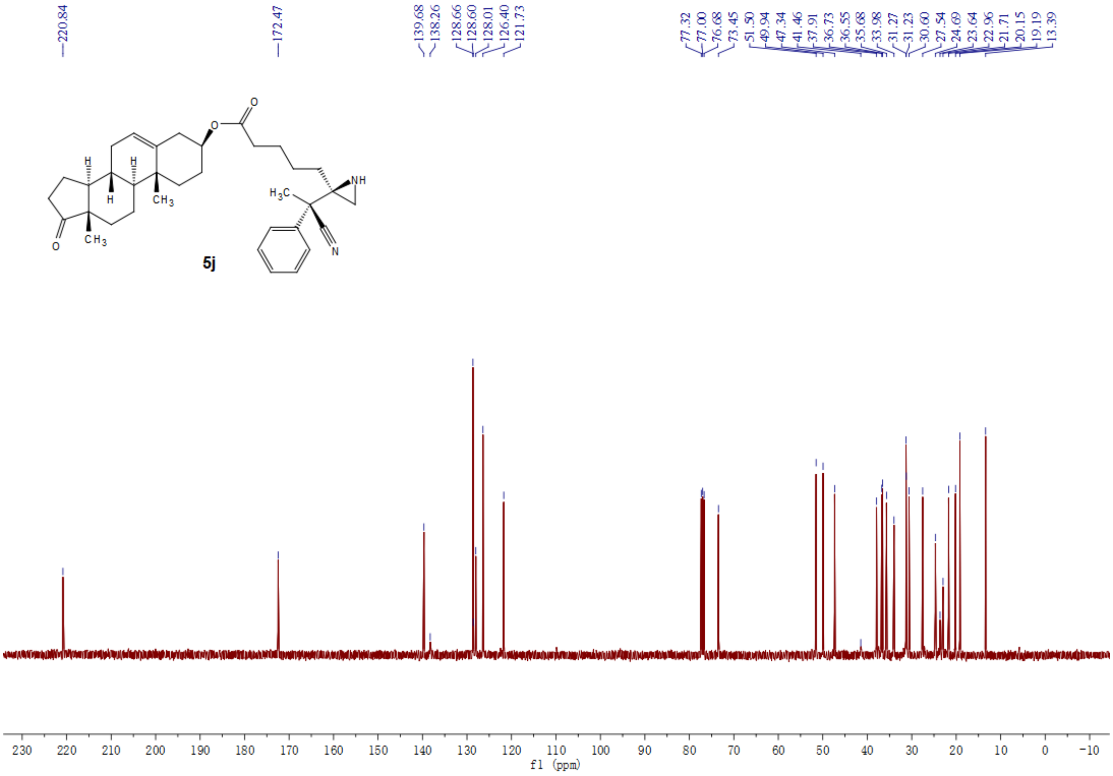

Supplementary Figure 134. ^{13}C NMR spectrum for compound **5j**

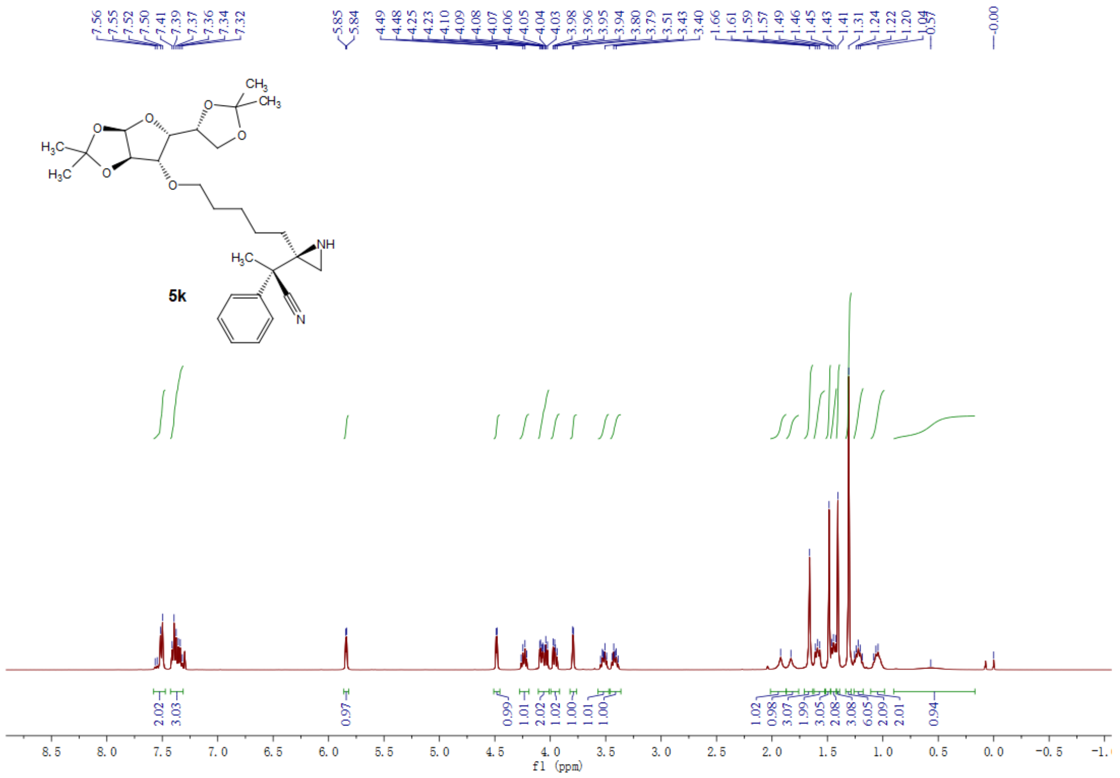

Supplementary Figure 135. ^{1}H NMR spectrum for compound **5k**

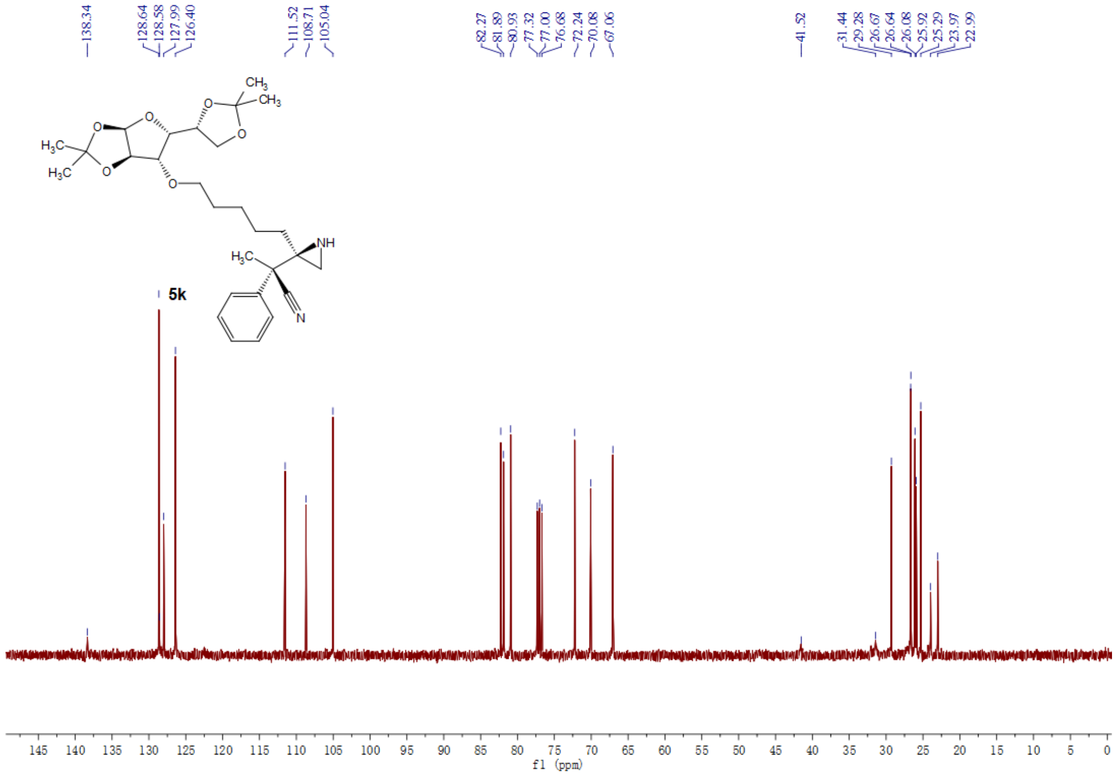

Supplementary Figure 136. ^{13}C NMR spectrum for compound **5k**

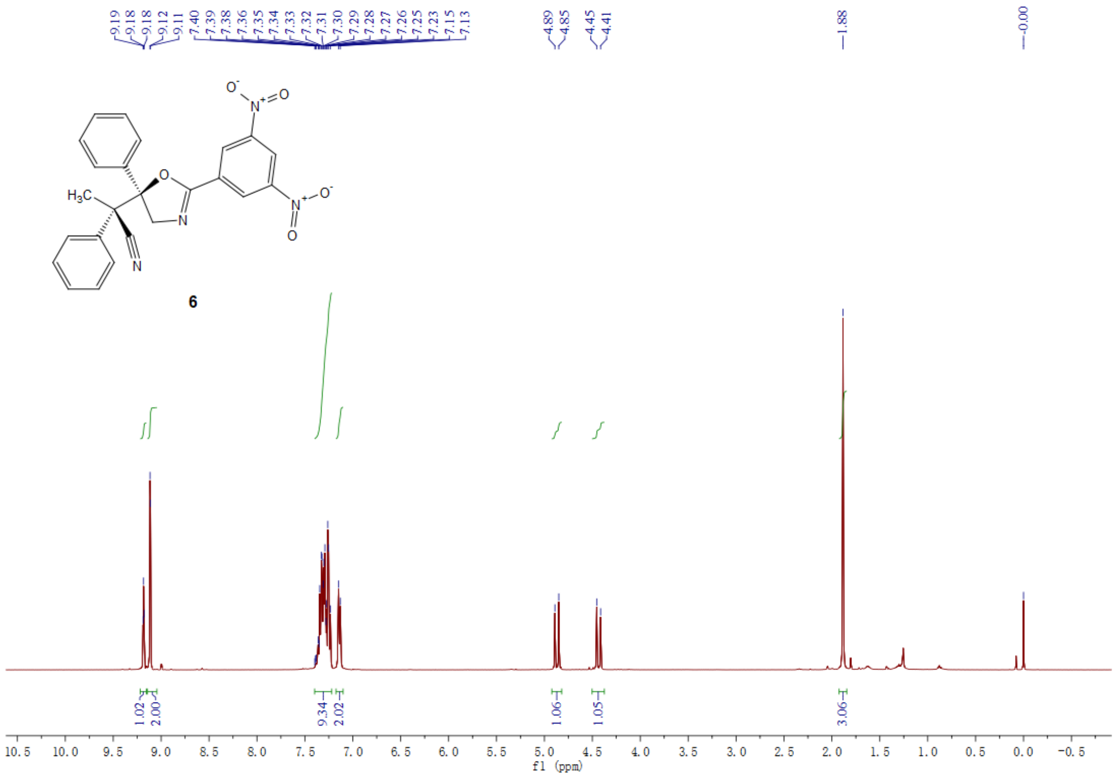

Supplementary Figure 137. ^{1}H NMR spectrum for compound **6**

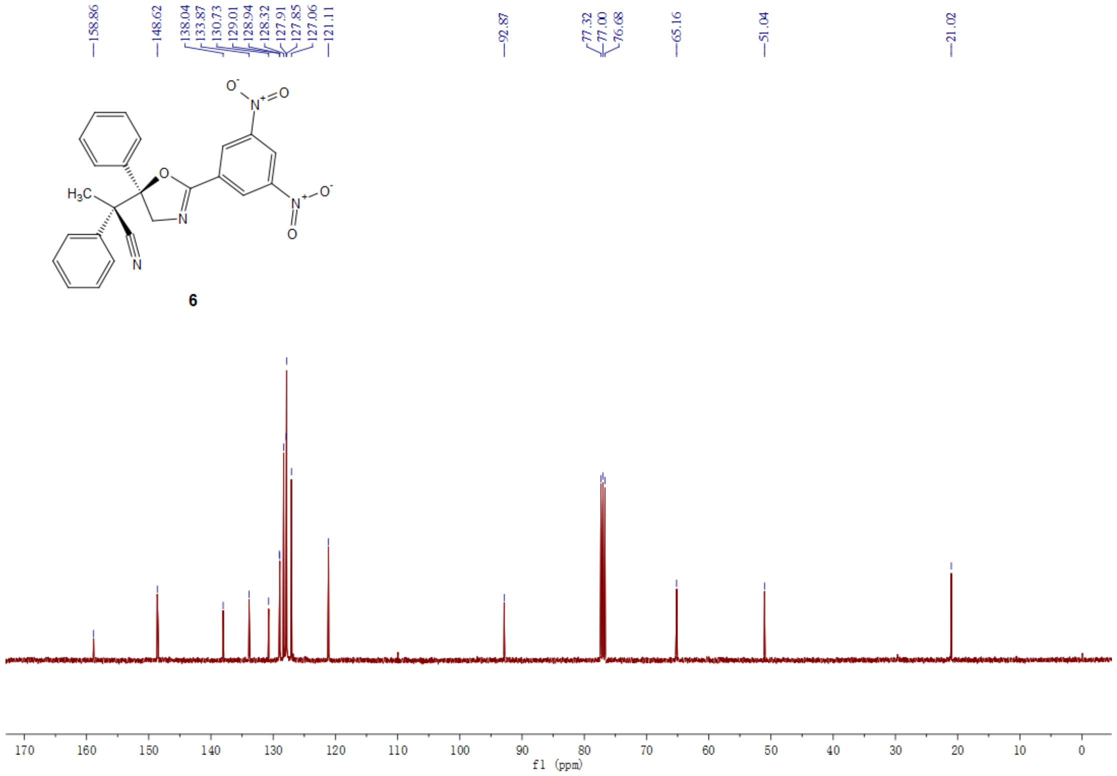

Supplementary Figure 138. ^{13}C NMR spectrum for compound **6**

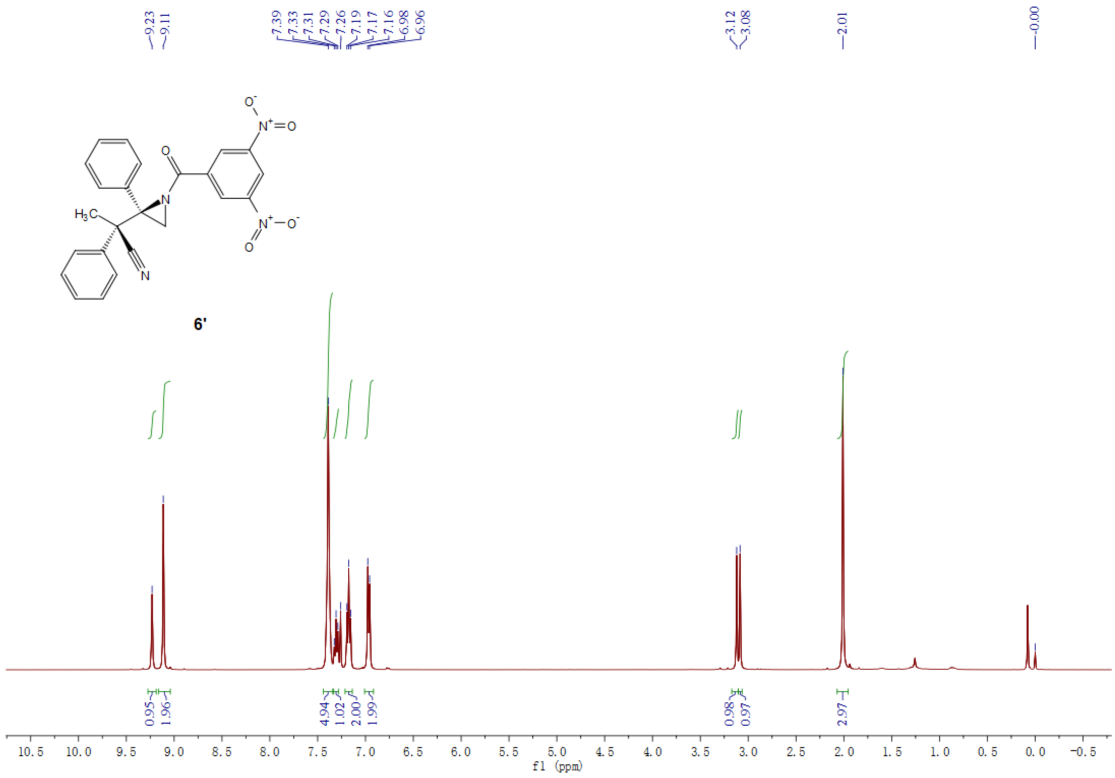

Supplementary Figure 139. ^{1}H NMR spectrum for compound **6'**

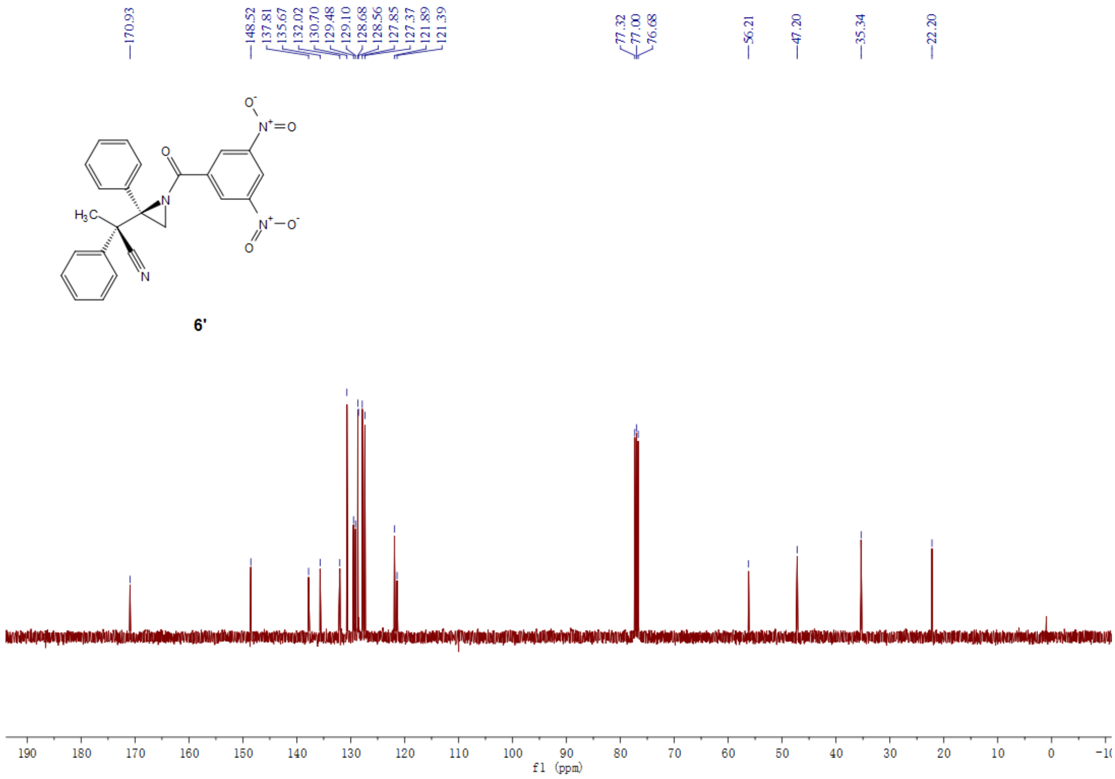

Supplementary Figure 140. ^{13}C NMR spectrum for compound **6'**

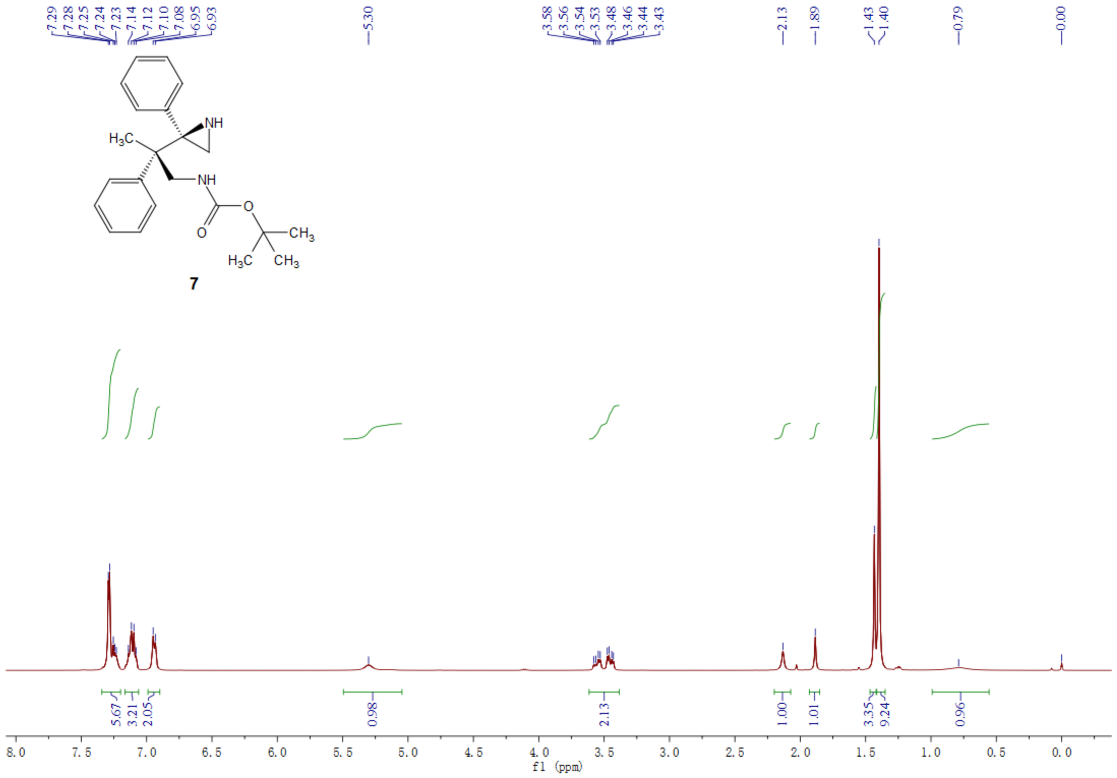

Supplementary Figure 141. ^{1}H NMR spectrum for compound **7**

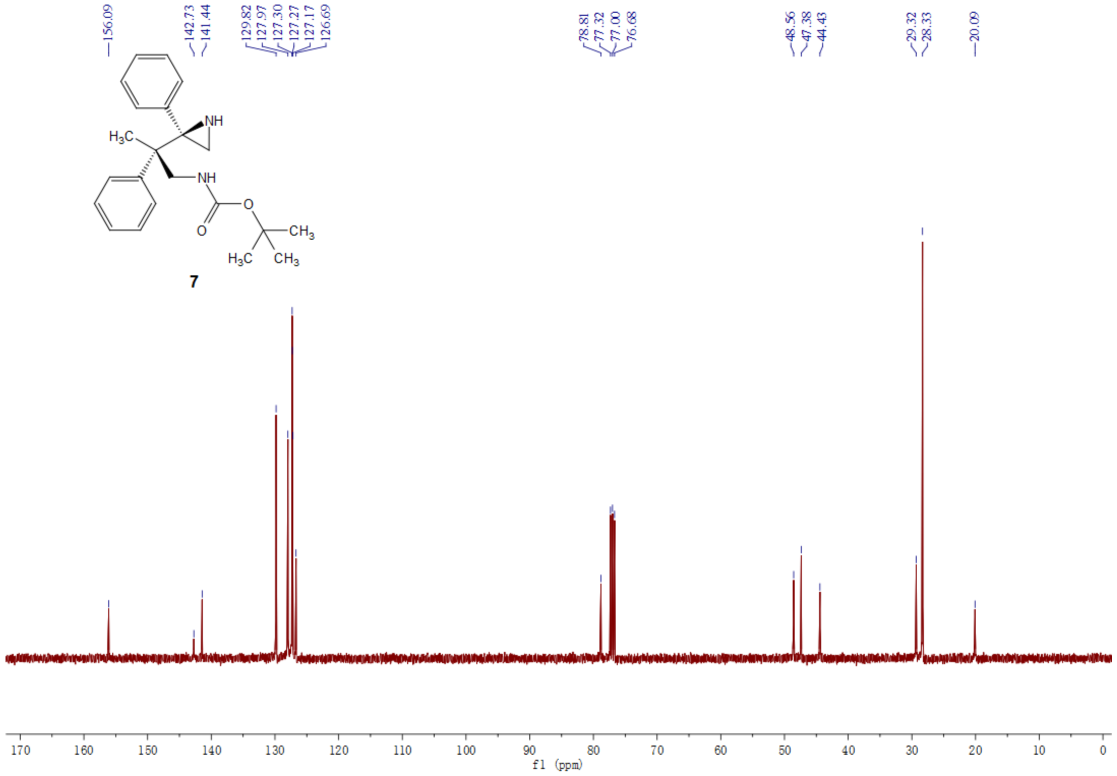

Supplementary Figure 142. ^{13}C NMR spectrum for compound **7**

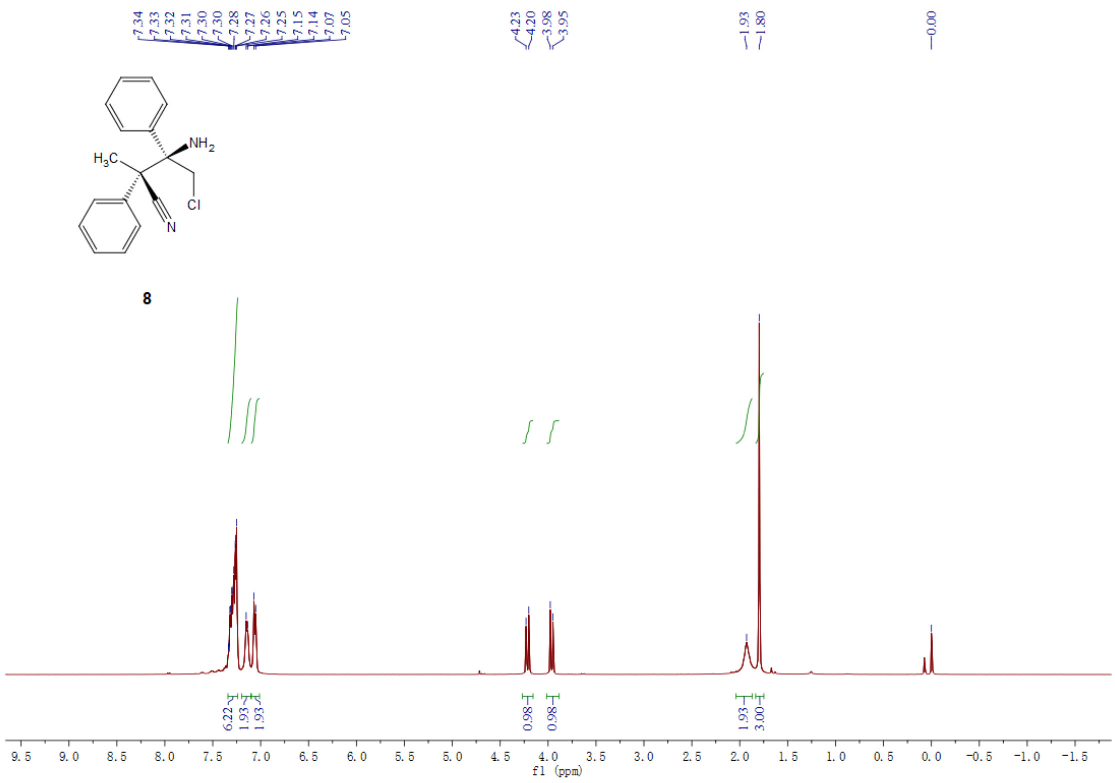

Supplementary Figure 143. ^{1}H NMR spectrum for compound **8**

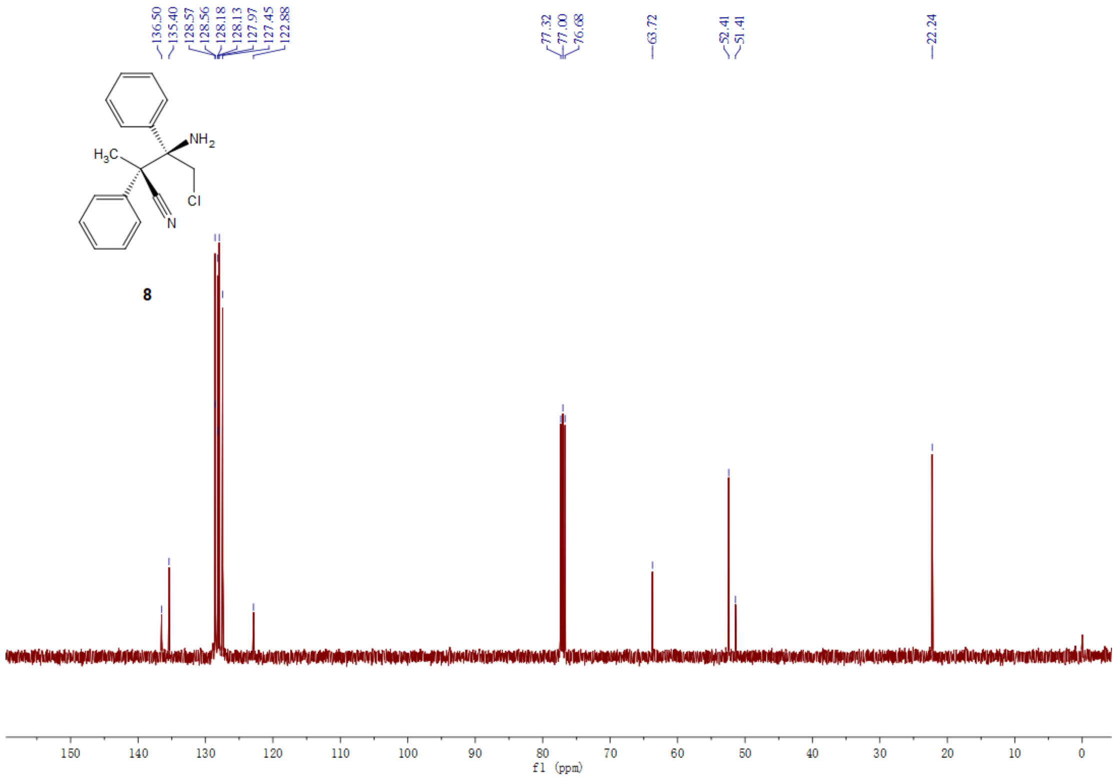

Supplementary Figure 144. ^{13}C NMR spectrum for compound **8**

Supplementary References

1. Yin, L., Kanai, M. & Shibasaki, M. Nucleophile generation via decarboxylation: asymmetric construction of contiguous trisubstituted and quaternary stereocenters through a Cu(I)-catalyzed decarboxylative Mannich-type reaction. *J. Am. Chem. Soc.* **131**, 9610-9611 (2009).
2. Nakamura, S. & Hayama, D. Enantioselective reaction of 2H-azirines with phosphite using chiral bis(imidazoline)/zinc(II) catalysts. *Angew. Chem. Int. Ed.* **56**, 8785-8789 (2017).
3. Peng, Q., Guo, D., Bie, J. & Wang, J. Catalytic enantioselective aza-benzoin reactions of aldehydes with 2H-azirines. *Angew. Chem. Int. Ed.* **57**, 3767-3771 (2018).
4. Liu, Z., Liao, P. & Bi, X. General silver-catalyzed hydroazidation of terminal alkynes by combining TMS-N_3 and H_2O: synthesis of vinyl azides. *Org. Lett.* **16**, 3668-3671 (2014).
5. Timén, Å. S., Risberg, E. & Somfai, P. Improved procedure for cyclization of vinyl azides into 3-substituted-2H-azirines. *Tetrahedron Lett.* **44**, 5339-5341 (2003).
